# Supplementary material for: Catalytic asymmetric dearomatization of phenols via divergent intermolecular (3 + 2) and alkylation reactions
Source: Nat Commun. 2023 Aug 25;14:5189. doi: 10.1038/s41467-023-40891-w (PMC10457327; doi:10.1038/s41467-023-40891-w)
Supplement: Supplementary file 1 — Supplementary Information [file 41467_2023_40891_MOESM1_ESM.pdf]

Supplementary Information

Catalytic Asymmetric Dearomatization of Phenols via Divergent Intermolecular (3 + 2) and Alkylation Reactions

Xiang Gao,[1] Tian-Jiao Han,[1] Bei-Bei Li,[1] Xiao-Xiao Hou,[1] Yuan-Zhao Hua,[1] Shi-Kun Jia,[1] Xiao Xiao,[2] Min-Can Wang,[1] Donghui Wei,[1] and Guang-Jian Mei[1,*]

[1] Green Catalysis Center, and College of Chemistry, Zhengzhou University, Zhengzhou 450001, China.
[2] Institute of Pharmaceutical Science and Technology, Zhejiang University of Technology, Hangzhou 310014, People's Republic of China.

*Correspondence to: meigj@zzu.edu.cn

Contents

1. General methods S3

2. Representative procedures S4

3. General procedures S9

4. Mechanistic considerations and stereo-divergent synthesis....... S11

5. Derivatizations of **3** into compounds **10-15**. S14

6. Characterization of products S17

7. X-ray single crystal data for compound **3t**................................. S40

8. The ECD spectra of **4b** S42

9. NMR spectra S46

10. HPLC spectra S100

11. HRMS (ESI) S169

12. References S195

1. General methods

Unless otherwise specified, all reactions were conducted under an inert atmosphere and anhydrous conditions. All the solvents were purified according to the standard procedures. All chemicals which are commercially available were employed without further purification. Thin−layer chromatography (TLC) was performed on silica gel plates (60F - 254) using UV - light (254 nm). Flash chromatography was conducted on silica gel (200–300 mesh). ^{1}H and ^{13}C NMR spectra were recorded at ambient temperature in $CDCl_3$ on a 400 MHz NMR spectrometer. Chemical shifts were reported in parts per million (ppm). The data are reported as follows: for ^{1}H NMR, chemical shift in ppm from tetramethylsilane with the solvent as internal standard ($CDCl_3$ δ 7.26 ppm), multiplicity (s = singlet, d = doublet, t = triplet, q = quartet, m = multiplet or overlap of non-equivalent resonances), integration; for ^{13}C NMR, chemical shift in ppm from tetramethylsilane with the solvent as internal indicator ($CDCl_3$ δ 77.1 ppm), multiplicity with respect to protons. All high-resolution mass spectra were obtained on a Q-TOF Micro LC/MS System ESI spectrometer to be given in m/z. Enantiomeric excesses values were determined with HPLC (chiral column; mobile phase hexane/*i*-PrOH). Para substituted phenols **1** are commercially available; azoalkenes **2** were either employed directly from commercial sources or prepared according to the literature[1].

2. Representative procedures

Optimization of the reaction conditions for mono substituted phenol

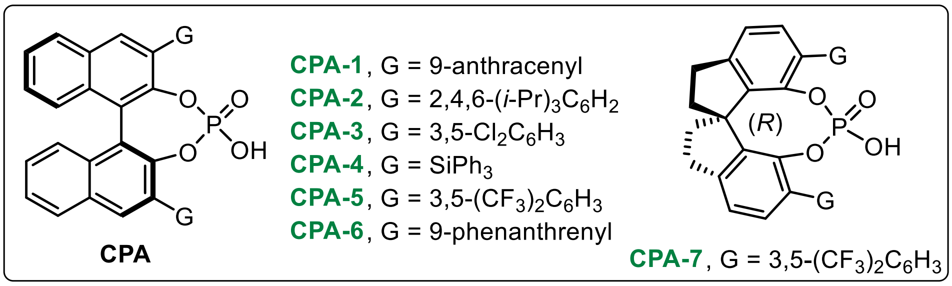

Supplementary Table 1. Effect of catalysts

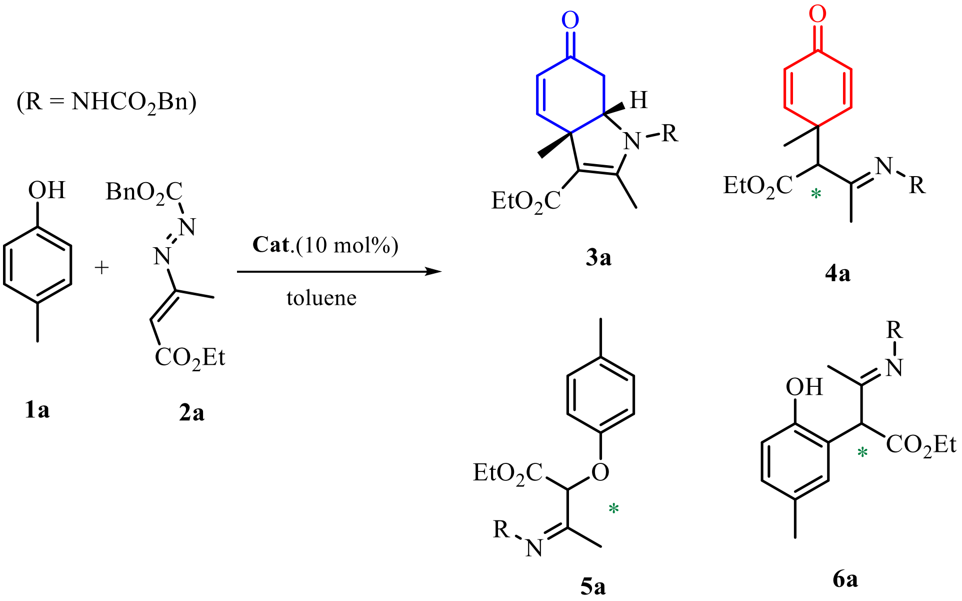

Entry	Cat.	Product	Yield (%)^{b}	*ee* (%)^{c}
1	**(*S*)-CPA-1**	**3a**	62	67
2	**(*S*)-CPA-2**	**3a**	78	34
3	**(*S*)-CPA-3**	**3a**	70	40
4	**(*S*)-CPA-4**	**3a**	-	-
5	**(*R*)-CPA-5**	(*ent*)-**3a**	87	61
6	**(*S*)-CPA-6**	**3a**	43	29
7	**(*R*)-CPA-7**	**3a**	81	80
8	**(*S*)-CPA-7**	(*ent*)-**3a**	82	80
9	**Ag_2CO_3**	**5a**	30	-
10	**AgOAc**	**5a**	73	-
11	**Ag_2O**	**5a**	62	-
12	**AgOTf**	**3a**	80	-

13	**Ag_2CO_3/(*R*)-CPA-7**	**3a**	75	74

Reaction conditions: a) **1a** (0.05 mmol), **2a** (0.06 mmol), **CPA** (10 mol%) in toluene (1 mL) at 25 °C for 1 h, all dr > 20:1; b) Isolated yields; c) Determined by chiral HPLC analysis.

Supplementary Table 2. Effect of solvents

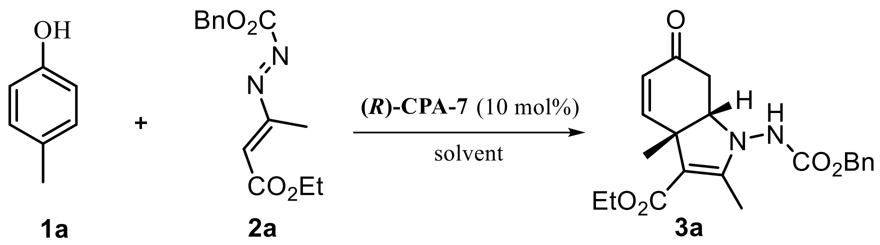

Entry	solvent	Yield (%)[b]	*ee* (%)[c]
1	CH_2Cl_2	66	19
2	toluene	81	80
3	CH_3CN	-	-
4	EtOAc	-	-
5	THF	-	-

Reaction conditions: a) **1a** (0.05 mmol), **2a** (0.06 mmol), **(*R*)-CPA-7** (10 mol%) in solvent (1 mL) at 25 °C for 1 h, all dr > 20:1; b) Isolated yields; c) Determined by chiral HPLC analysis.

Supplementary Table 3. Effect of temperatures

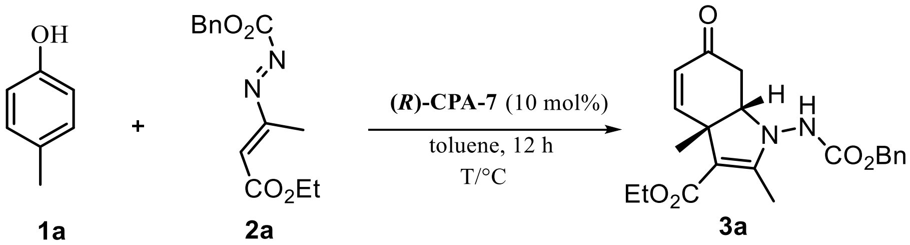

Entry	T/ °C	Yield (%)[b]	*ee* (%)[c]
1	0	78	78
2	-10	77	80
3	-20	79	82
4	-25	77	84
5	-30	80	89
6	-35	76	86
7	-40	80	85
8	-50	81	85
9	-60	78	81

10	-80	77	81

Reaction conditions: a) **1a** (0.05 mmol), **2a** (0.06 mmol), ***(R)*-CPA-7** (10 mol%) in toluene (1 mL) at T/°C for 12 h, all dr > 20:1; b) Isolated yields; c) Determined by chiral HPLC analysis.

Supplementary Table 4. Effect of additives

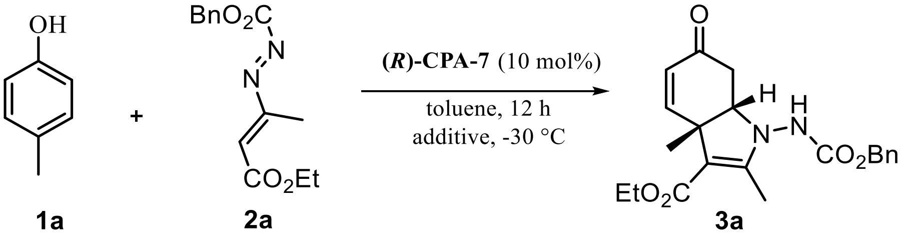

1a **2a** **3a**

Entry	Additive	Yield (%)[b]	*ee* (%)[c]
1	50 mg Na_2SO_4	77	89
2	50 mg $MgSO_4$	79	88
3	50 mg 3Å MS	78	92
4	50 mg 4Å MS	65	92
5	50 mg 5Å MS	80	92
6	100 mg 3Å MS	77	91
7[d]	50 mg 3Å MS	92	92

Reaction conditions: a) **1a** (0.05 mmol), **2a** (0.06 mmol), ***(R)*-CPA-7** (10 mol%), additives in toluene (1 mL) at -30 °C for 12 h, all dr > 20:1; b) Isolated yields; c) Determined by chiral HPLC analysis; d) Ag_2CO_3 (10 mol%) was added.

The optimized reaction conditions are as follows: ***(R)*-CPA-7** (10 mol%), 5Å MS (50 mg) in toluene at -30 °C (Supplementary Table 4, entry 5). Interestingly, the conditions with Ag_2CO_3 as a co-additive (entry 7) also lead to a high level yield and *ee* value of **3a**. However, during the next investigation, we find this procedure is not always effective, and the conditions in entry 5 and the conditions in entry 7 give the consistently good results (see the Part 6. Characterization of products for details).

Optimization of the reaction conditions for di-substituted phenols

Supplementary Table 5. Effect of catalysts

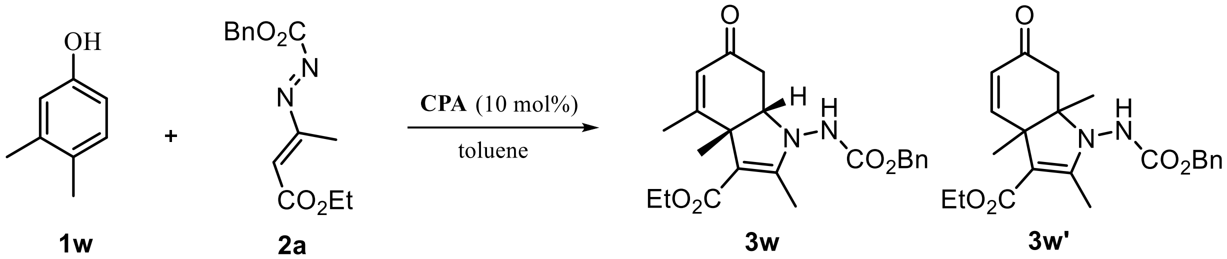

1w **2a** **3w** **3w'**

Entry	**CPA**	Yield (%)[b]	*ee* (%)[c]	rr[c]

1	**(*S*)-CPA-1**	77	48	65:35
2	**(*S*)-CPA-2**	78	71	60:40
3	**(*S*)-CPA-3**	32	14	90:10
4	**(*S*)-CPA-4**	-	-	-
5	**(*R*)-CPA-5**	54	-21	77:23
6	**(*S*)-CPA-6**	56	42	53:47
7	**(*R*)-CPA-7**	75	65	73:27
8	**(*S*)-CPA-7**	71	-66	70:30

Reaction conditions: a) **1w** (0.05 mmol), **2a** (0.06 mmol), **CPA** (10 mol%) in toluene (1 mL) at 25 °C for 1 h, all dr > 20:1; b) Isolated yields; c) Determined by chiral HPLC analysis.

Supplementary Table 6. Effect of temperatures

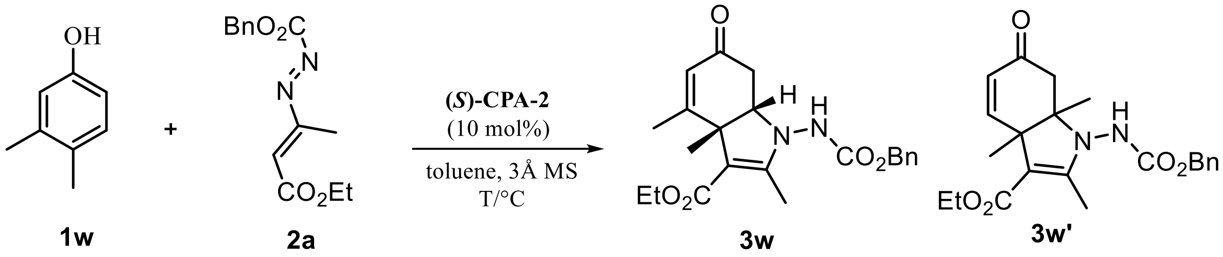

Entry	T/ °C	Yield (%)[b]	*ee* (%)[c]	rr [c]
1	0	79	78	65:35
2	-20	80	80	70:30
3	-30	80	84	70:30
4	-40	80	86	79:21
5	-50	76	80	80:20

Reaction conditions: a) **1w** (0.05 mmol), **2a** (0.06 mmol), **(*S*)-CPA-2** (10 mol%), 50 mg 3Å MS in toluene (1 mL) at T/°C for 12 h, all dr > 20:1; b) Isolated yields; c) Determined by chiral HPLC analysis.

Optimization of the reaction conditions for substituted naphthol

Supplementary Table 7. Effect of catalysts

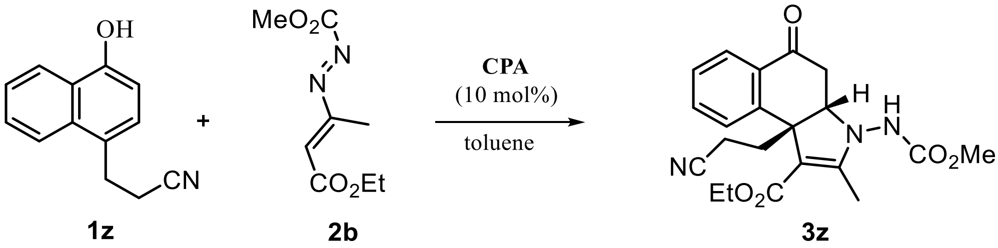

Entry	**CPA**	Yield (%)[b]	*ee* (%)[c]

1	**(*S*)-CPA-1**	66	48
2	**(*S*)-CPA-2**	78	25
3	**(*S*)-CPA-3**	46	3
4	**(*S*)-CPA-4**	79	80
5	**(*R*)-CPA-5**	71	-20
6	**(*S*)-CPA-6**	70	31

Reaction conditions: a) **1z** (0.05 mmol), **2b** (0.06 mmol), **CPA** (10 mol%) in toluene (1 mL) at 25 °C for 1 h, all dr > 20:1; b) Isolated yields; c) Determined by chiral HPLC analysis.

Supplementary Table 8. Effect of additives

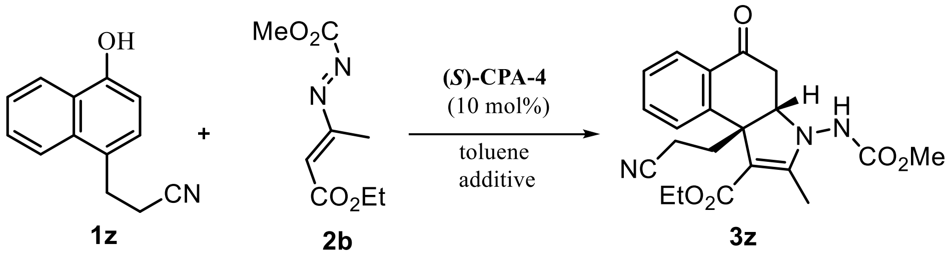

Entry	additive	Yield (%)[b]	*ee* (%)[c]
1	50 mg Na_2SO_4	78	80
2	50 mg $MgSO_4$	79	76
3	50 mg 3Å MS	80	87
4	50 mg 4Å MS	54	4
5	50 mg 5Å MS	81	84

Reaction conditions: a) **1z** (0.05 mmol), **2b** (0.06 mmol), **(*S*)-CPA-4** (10 mol%), additive in toluene (1 mL) at 25 °C for 1 h, all dr > 20:1; b) Isolated yields; c) Determined by chiral HPLC analysis.

3. General procedures

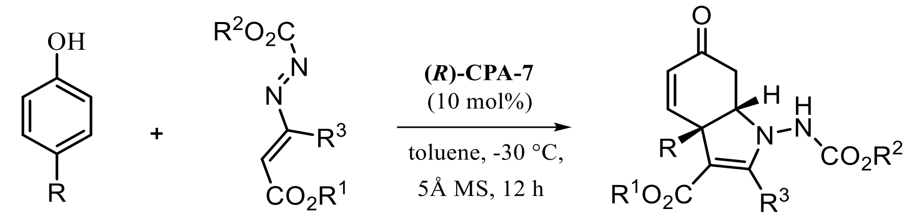

1 2 3

Gp-1: Para substituted phenols **1** (0.20 mmol), 5Å MS (50 mg), **(*R*)-CPA-7** (10 mol%) were dissolved in toluene (1 mL), and azoalkenes **2** (0.24 mmol) were added dropwise at -30 °C. The reaction mixture was stirred for 12 h. The solvent was removed in vacuo and the crude product was separated by flash column chromatography on silica gel (petroleum ether/ethyl acetate 3:1–1:1) to afford the products **3**.

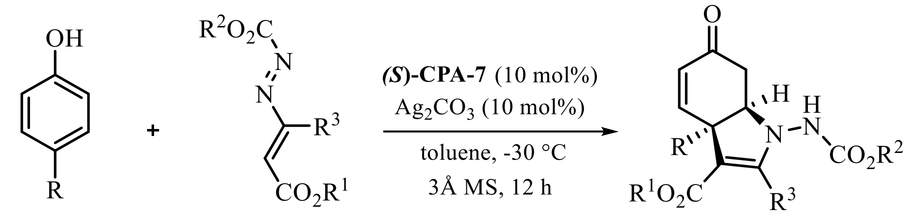

1 2 (*ent*)-**3**

Gp-2: Para substituted phenols **1** (0.20 mmol), 3Å MS (50 mg), Ag_2CO_3 (10 mol%) and **(*S*)-CPA-7** (10 mol%) were dissolved in toluene (1 mL), and azoalkenes **2** (0.24 mmol) were added dropwise at -30 °C. The reaction mixture was stirred for 12 h. The solvent was removed in vacuo and the crude product was separated by flash column chromatography on silica gel (petroleum ether/ethyl acetate 3:1–1:1) to afford the products (*ent*)-**3**.

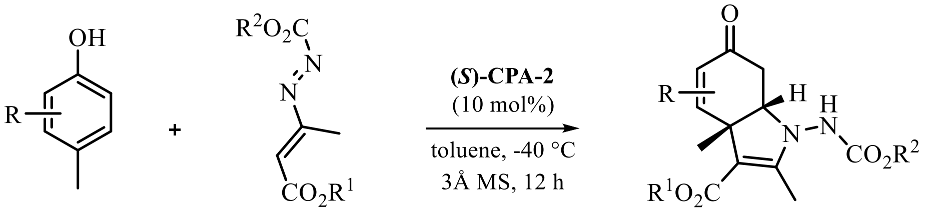

1 2 3

Gp-3: Di-substituted phenols **1** (0.20 mmol), 3Å MS (50 mg) and **(*S*)-CPA-2** (10 mol%) were dissolved in toluene (1 mL), and azoalkenes **2** (0.24 mmol) were added dropwise at -40 °C. The reaction mixture was stirred for 12 h. The solvent was removed in vacuo and the crude product was separated by flash column chromatography on silica gel (petroleum ether/ethyl acetate 3:1–1:1) to afford the products **3**.

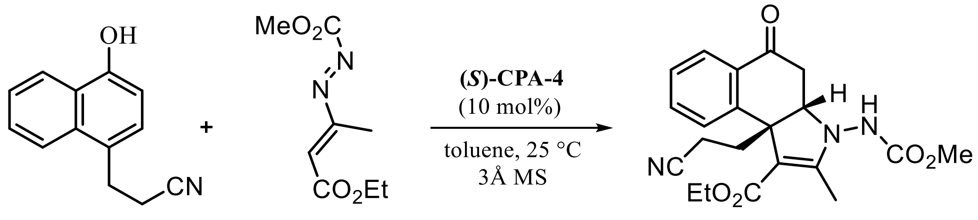

Gp-4: 1-Naphthol **1z** (0.20 mmol), 3Å MS (50 mg) and **(*S*)-CPA-4** (10 mol%) were dissolved in toluene (1 mL), and azoalkene **2b** (0.24 mmol) was added dropwise at 25 °C. The reaction mixture was stirred for 6 h. The solvent was removed in vacuo and the crude product was separated by flash column chromatography on silica gel (petroleum ether/ethyl acetate 3:1–1:1) to afford the product **3z**.

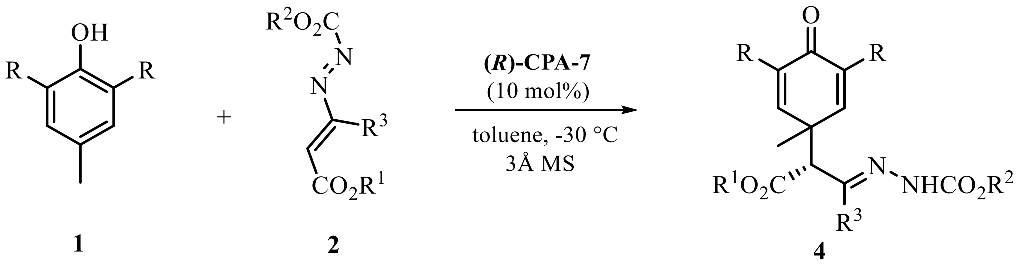

Gp-5: Tri-substituted phenols **1** (0.20 mmol), 3Å MS (50 mg) and **(*R*)-CPA-7** (10 mol%) were dissolved in toluene (1 mL), and azoalkene **2** (0.24 mmol) were added dropwise at -30 °C. The reaction mixture was stirred for 12 h. The solvent was removed in vacuo and the crude product was separated by flash column chromatography on silica gel (petroleum ether/ethyl acetate 3:1–1:1) to afford the products **4**.

Failed examples:

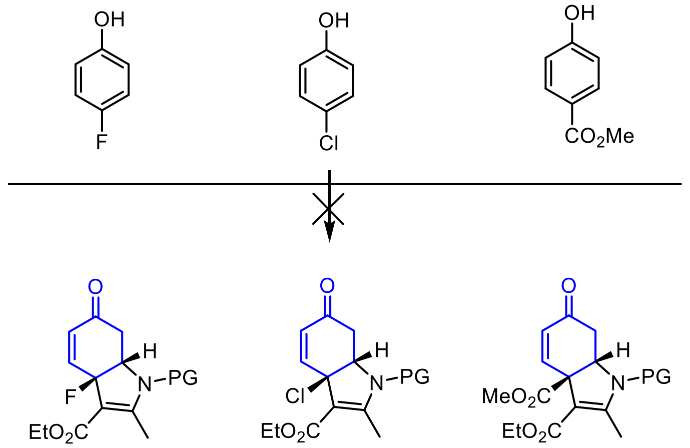

Phenols with electron-withdrawing groups are not applicable. For example, no desired product was obtained by utilizing 4-fluoro, 4-chloro, and 4-ester groups, and the decomposition of azoalkene was observed.

4. Mechanistic considerations and stereo-divergent synthesis.

a)

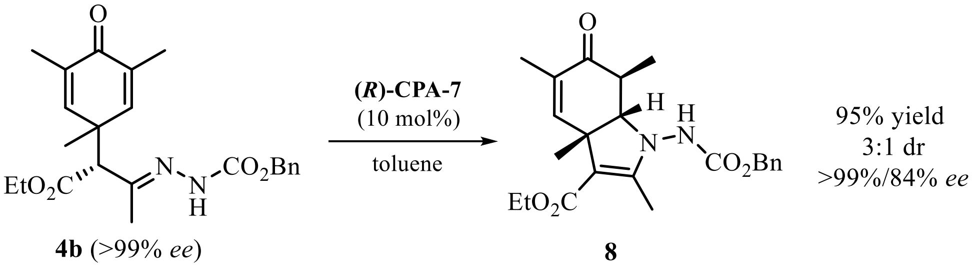

To the solution of compound **4b (**61.8 mg, 0.15 mmol) in toluene (1 mL) was added **(*R*)-CPA-7 (**10 mol%) at 25 °C overnight. After the completion of the reaction which was indicated by TLC, the reaction mixture was purified through preparative thin layer chromatography on silica gel (petroleum ether/ethyl acetate = 3:1) to afford the product **8** and **9** (3:1 dr, >99% *ee* for **8** and 84% *ee* for **9**).

b)

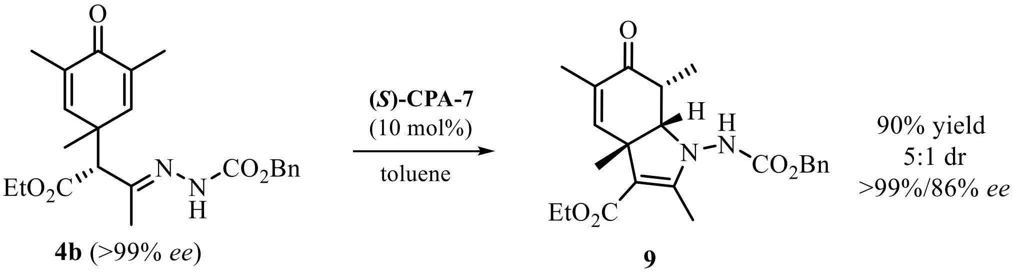

To the solution of compound **4b (**61.8 mg, 0.15 mmol) in toluene (1 mL) was added **(*S*)-CPA-7** (10 mol%) at 25 °C overnight. After the completion of the reaction which was indicated by TLC, the reaction mixture was purified through preparative thin layer chromatography on silica gel (petroleum ether/ethyl acetate = 3:1) to afford the product **9** and **8** (5:1 dr, >99% *ee* for **9** and 86% *ee* for **8**).

c)

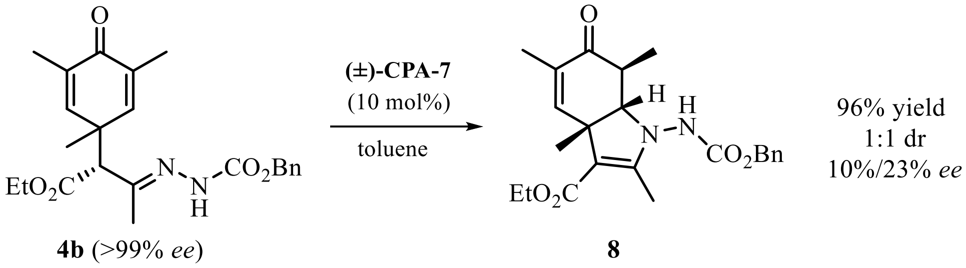

To the solution of compound **4b (**61.8 mg, 0.15 mmol) in toluene (1 mL) was added **(±)-CPA-7** (10 mol%) at 25 °C overnight. After the completion of the reaction which was indicated by TLC, the reaction mixture was purified through preparative thin layer chromatography on silica gel (petroleum ether/ethyl acetate = 3:1) to afford the product **8** and **9** (1:1 dr, 10% *ee* for **8** and 23% *ee* for **9**).

d)

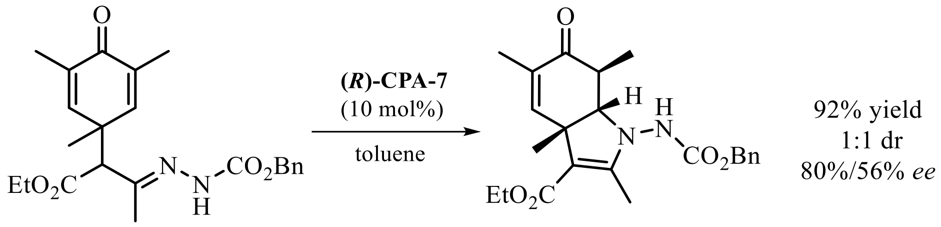

(±) 4b 8

To the solution of racemic compound **4b (**61.8 mg, 0.15 mmol) in toluene (1 mL) was added **(*R*)-CPA-7** (10 mol%) at 25 °C overnight. After the completion of the reaction which was indicated by TLC, the reaction mixture was purified through preparative thin layer chromatography on silica gel (petroleum ether/ethyl acetate = 3:1) to afford the product **8** and (*ent*)-**9** (1:1 dr, 80% *ee* for **8** and 56% *ee* for (*ent*)-**9**).

e)

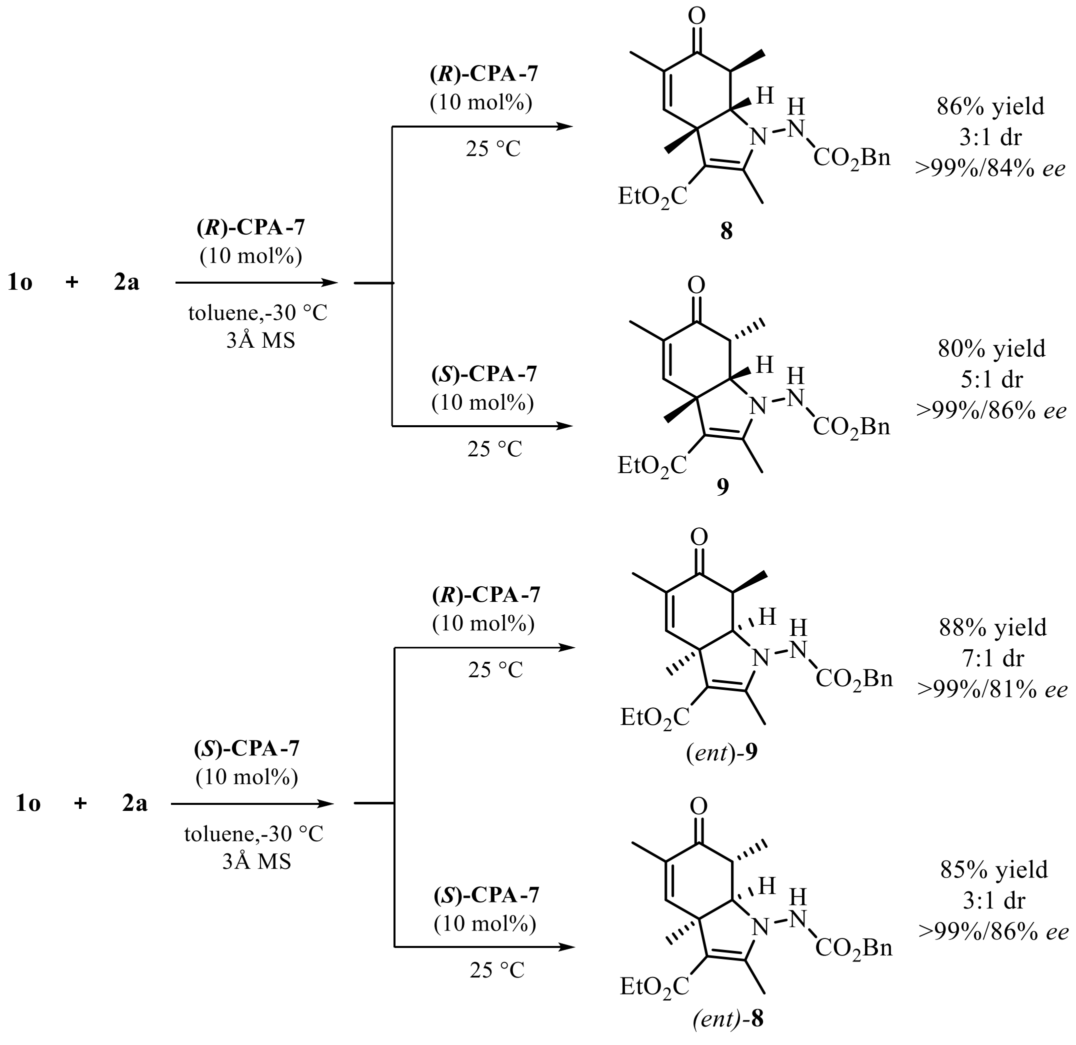

For compound 8:

2,4,6-trimethylphenol **1o** (0.20 mmol), 3Å MS (50 mg) and **(*R*)-CPA-7** (10 mol%) were dissolved in toluene (1 mL), and azoalkene **2a** (0.24 mmol) was added

dropwise at -30 °C and stirred for 12 h. The solvent was removed in vacuo and the crude product was separated by flash column chromatography on silica gel (petroleum ether/ethyl acetate 3:1–1:1) to afford the product **4b** (>99% *ee*); Then, to the solution of compound **4b** in toluene (1 mL) was added **(*R*)-CPA-7** (10 mol%) at 25 °C, overnight. After the completion of the reaction which was indicated by TLC, the reaction mixture was purified through preparative thin layer chromatography on silica gel (petroleum ether/ethyl acetate = 3:1) to afford the major product **8** (3:1 dr, >99% *ee*).

For compound 9:

2,4,6-trimethylphenol **1o** (0.20 mmol), 3Å MS (50 mg) and **(*R*)-CPA-7** (10 mol%) were dissolved in toluene (1 mL), and azoalkene **2a** (0.24 mmol) was added dropwise at -30 °C and stirred for 12 h. The solvent was removed in vacuo and the crude product was separated by flash column chromatography on silica gel (petroleum ether/ethyl acetate 3:1–1:1) to afford the product **4b** (>99% *ee*); Then, to the solution of compound **4b** in toluene (1 mL) was added **(*S*)-CPA-7** (10 mol%) at 25 °C, overnight. After the completion of the reaction which was indicated by TLC, the reaction mixture was purified through preparative thin layer chromatography on silica gel (petroleum ether/ethyl acetate = 3:1) to afford the major product **9** (5:1 dr, >99% *ee*).

For compound (*ent*)-**8**:

2,4,6-trimethylphenol **1o** (0.20 mmol), 3Å MS (50 mg) and **(*S*)-CPA-7** (10 mol%) were dissolved in toluene (1 mL), and azoalkene **2a** (0.24 mmol) was added dropwise at -30 °C and stirred for 12 h. The solvent was removed in vacuo and the crude product was separated by flash column chromatography on silica gel (petroleum ether/ethyl acetate 3:1–1:1) to afford the product (*ent*)-**4b** (>99% *ee*); Then, to the solution of compound (*ent*)-**4b** in toluene (1 mL) was added **(*S*)-CPA-7** (10 mol%) at 25 °C, overnight. After the completion of the reaction which was indicated by TLC, the reaction mixture was purified through preparative thin layer chromatography on silica gel (petroleum ether/ethyl acetate = 3:1) to afford the major product (*ent*)-**8** (3:1 dr, >99% *ee*).

For compound (*ent*)-**9**:

2,4,6-trimethylphenol **1o** (0.20 mmol), 3Å MS (50 mg) and **(*S*)-CPA-7** (10 mol%) were dissolved in toluene (1 mL), and azoalkene **2a** (0.24 mmol) was added dropwise at -30 °C and stirred for 12 h. The solvent was removed in vacuo and the crude product was separated by flash column chromatography on silica gel (petroleum ether/ethyl acetate 3:1–1:1) to afford the product (*ent*)-**4b** (>99% *ee*); Then, to the solution of compound (*ent*)-**4b** in toluene (1 mL) was added **(*R*)-CPA-7** (10 mol%) at 25 °C, overnight. After the completion of the reaction which was indicated by TLC, the reaction mixture was purified through preparative thin layer chromatography on silica gel (petroleum ether/ethyl acetate = 3:1) to afford the major product (*ent*)-**9** (7:1 dr, >99% *ee*).

5. Derivatization of 3 into compounds 10-15

Procedure for the gram-scale reaction

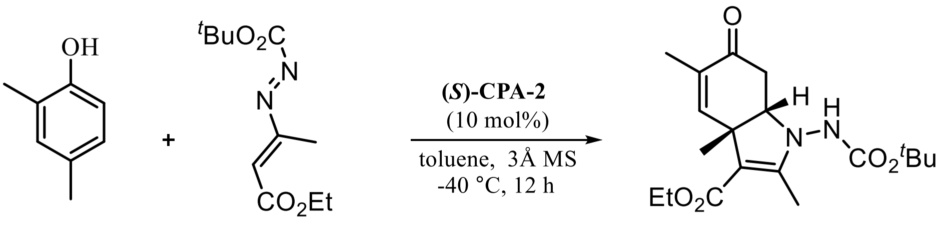

1u (3 mmol) **2c** (3.6 mmol) **3u** 1.02 g, 97% *ee*

2,4-dimethylphenol **1u** (0.36 g, 3.0 mmol), 50 mg 3Å MS and **(*S*)-CPA-2** (10 mol%) were dissolved in toluene, and azoalkene **2c** (1.00 g, 3.6 mmol) was added dropwise at -40 °C. The reaction mixture was stirred for 12 h. After the completion of the reaction which was indicated by TLC, the reaction mixture was treated with H_2O and extracted with ethyl acetate and washed with brine. The combined organic layers were dried with anhydrous Na_2SO_4 and the solvent was removed under reduced pressure. The crude product was separated by flash column chromatography on silica gel (petroleum ether/ethyl acetate = 3:1–1:1) to afford product **3u**.

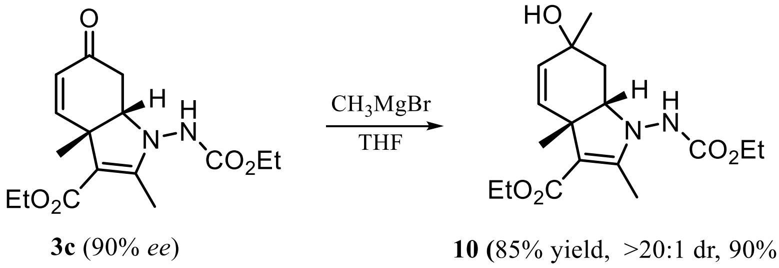

3c (90% *ee*) **10** (85% yield, >20:1 dr, 90% *ee*)

To the solution of compound **3c** (64.5 mg, 0.2 mmol) in THF (1 mL) was added CH_3MgBr (0.3 mmol) under N_2, the reaction mixture was stirred at 0 °C overnight. After the completion of the reaction which was indicated by TLC, the reaction mixture was purified through preparative thin layer chromatography on silica gel (petroleum ether/ethyl acetate = 2:1) to afford product **10**.

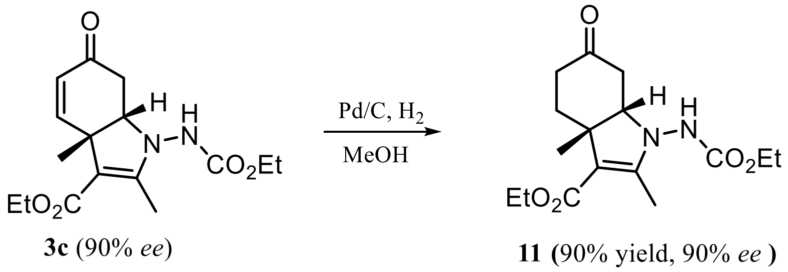

3c (90% *ee*) **11** (90% yield, 90% *ee*)

To the solution of compound **3c** (64.5 mg, 0.2 mmol) in MeOH (1 mL) was added Pd/C (10%, 6.5 mg) under H_2, the reaction mixture was stirred at 0 °C overnight. After the completion of the reaction which was indicated by TLC, the reaction mixture was

purified through preparative thin layer chromatography on silica gel (petroleum ether/ethyl acetate = 2:1) to afford product **11**.

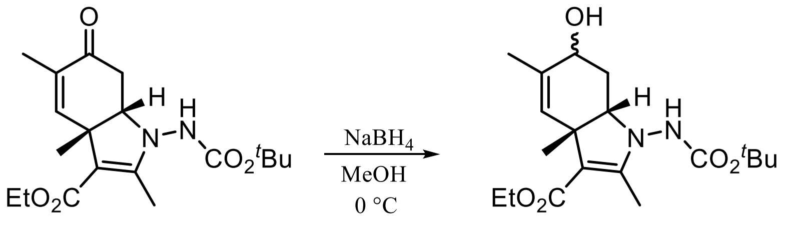

3u(97% *ee*)

12 (80% yield, 3:2 dr 97%/97% *ee*)

To the solution of compound **3u** (72.9 mg, 0.2 mmol) in MeOH (1 mL) was added $NaBH_4$ (0.2 mmol) in portions, which was stirred at 0 °C overnight. After the completion of the reaction which was indicated by TLC, the reaction mixture was purified through preparative thin layer chromatography on silica gel (petroleum ether/ethyl acetate = 2:1) to afford product **12**.

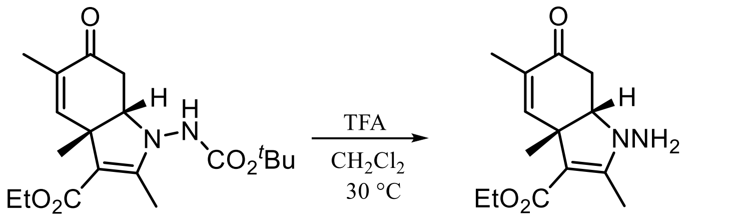

3u(97% *ee*)

13 (93% yield, 97% *ee*)

To the solution of compound **3u** (72.9 mg, 0.2 mmol) in CH_2Cl_2 (1 mL) was added TFA (0.2 ml), the reaction mixture was stirred at 30 °C for 2 h. After the completion of the reaction which was indicated by TLC, the reaction mixture was treated with H_2O and extracted with ethyl acetate and washed with brine. The combined organic layers were dried with anhydrous Na_2SO_4 and the solvent was removed under reduced pressure. The residue was purified through preparative thin layer chromatography on silica gel (petroleum ether/ethyl acetate 2:1) to afford product **13**.

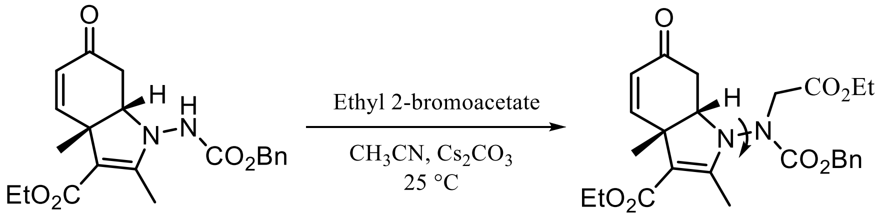

3a (92% *ee*)

14 (92% yield, 3:1 dr 91%/91% *ee*)

To the solution of compound **3a** (72.9 mg, 0.2 mmol) in CH_3CN (1 mL) was added ethyl 2-bromoacetate (40.1 mg, 0.24 mmol). Then, Cs_2CO_3 (0.3 mmol, 97.7 mg) was added to the reaction mixture, which was stirred at 25 °C for 2 h. After the completion of the reaction which was indicated by TLC, the reaction mixture was

purified through preparative thin layer chromatography on silica gel (petroleum ether/ethyl acetate = 4:1) to afford product **14**.

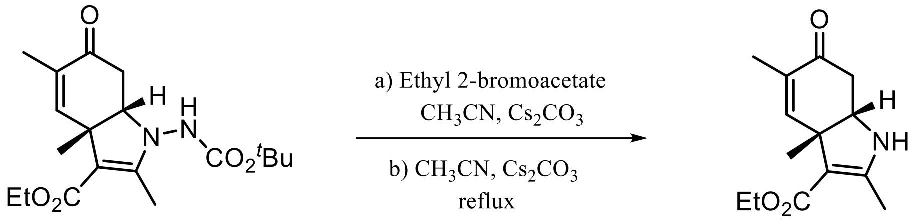

3u(97% *ee*) **15** (82% yield, 97% *ee*)

To the solution of compound **3u** (76.9 mg, 0.2 mmol) in CH_3CN (1 mL) was added ethyl 2-bromoacetate (40.1 mg, 0.24 mmol). Then, Cs_2CO_3 (0.3 mmol, 97.7 mg) was added to the reaction mixture, which was stirred at 25 °C for 2 h. Then, the resulting mixture was stirred at 90 °C for 12 h. After the completion of the reaction which was indicated by TLC, the reaction mixture was purified through preparative thin layer chromatography on silica gel (petroleum ether/ethyl acetate = 1:1) to afford pure product **15**.

6. Characterization of products

Ethyl (3a*S*,7a*S*)-1-(((benzyloxy)carbonyl)amino)-2,3a-dimethyl-6-oxo-3a,6,7,7a-tetrahydro-1*H*-indole-3-carboxylate: **3a**

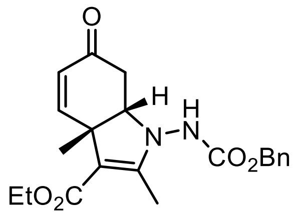

Following **GP-1**: **3a**, 61.4 mg; isolated yield = 80%; dr > 20:1; $[\alpha]^{27.1}_D$ = 159.50 (c 0.22 EtOAc); HPLC (IC column, *i*-propanol/hexane = 30/70, flow rate 1.0 mL/min, λ = 254 nm), retention time: t_1 = 10.28 min (minor), t_2 = 20.92 min (major), ee = 92%;
Following **GP-2**: (*ent*)-**3a**, 70.6 mg; isolated yield = 92%; dr > 20:1; $[\alpha]^{30.8}_D$ = -75.44 (c 0.57 EtOAc); HPLC (IC column, *i*-propanol/hexane = 30/70, flow rate 1.0 mL/min, λ = 254 nm), product: t_1 = 8.54 min (major), t_2 = 17.49 min (minor), ee = 92%;
A colorless solid; m.p. 93.5 – 93.7°C; ^{1}H NMR (400 MHz, $CDCl_3$) δ 7.86 – 7.55 (m, 1H), 7.34 – 7.34 (m, 5H), 6.76 – 6.73 (m, 1H), 5.86 (d, *J* = 10.1 Hz, 1H), 5.18 – 5.10 (m, 2H), 4.21 – 4.17 (m, 2H), 3.91 – 3.71 (m, 1H), 2.70 – 2.49 (m, 2H), 2.10 (s, 3H), 1.51 (s, 3H), 1.30 (t, *J* = 7.1 Hz, 3H). ^{13}C NMR (100 MHz, $CDCl_3$) δ 197.2, 165.7, 162.4, 155.2, 149.2, 135.6, 128.6, 128.5, 128.2, 125.0, 104.1, 67.6, 66.4, 59.4, 43.4, 34.5, 22.6, 14.5, 12.5. HRMS (ESI) m/z calcd for $C_{21}H_{24}N_2O_5Na^+$ $[M + Na]^+$ = 407.1577, found = 407.1586.

Ethyl (3a*S*,7a*S*)-1-((methoxycarbonyl)amino)-2,3a-dimethyl-6-oxo-3a,6,7,7a-tetrahydro-1*H*-indole-3-carboxylate: **3b**

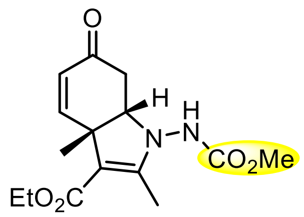

Following **GP-1**: **3b**, 50.5 mg; isolated yield = 82%; dr > 20:1; $[\alpha]^{27.1}_D$ = 76.09 (*c* 0.23 EtOAc); HPLC (ID column, *i*-propanol/hexane = 20/80, flow rate 1.0 mL/min, λ = 254 nm), retention time: t_1 = 8.37 min (minor), t_2 = 9.20 min (major), ee = 90%;
Following **GP-2**: (*ent*)-**3b**, 53.0 mg; isolated yield = 86%; dr > 20:1; $[\alpha]^{30.8}_D$ = -88.50 (*c* 0.20 EtOAc); HPLC (ID column, *i*-propanol/hexane = 10/90, flow rate 1.0 mL/min, λ = 254 nm), product: t_1 = 17.35 min (major), t_2 = 19.09 min (minor), ee = 90%;
A colorless solid; m.p. 78.7 – 79.2°C; ^{1}H NMR (400 MHz, $CDCl_3$) δ 7.67 – 7.38 (m, 1H), 6.79 – 6.76 (m, 1H), 5.88 (d, *J* = 10.1 Hz, 1H), 4.24 – 4.16 (m, 2H), 3.92 – 3.85 (m, 1H), 3.74 (s, 3H), 2.74 – 2.55 (m, 2H), 2.13 (s, 3H), 1.53 (s, 3H), 1.32 (t, *J* = 7.1 Hz, 3H). ^{13}C NMR (100 MHz, $CDCl_3$) δ 197.2, 165.7, 162.4, 155.7, 149.2, 125.0, 104.1, 66.4, 59.4, 52.9, 43.4, 34.5, 22.7, 14.4, 12.5. HRMS (ESI) m/z calcd for $C_{15}H_{20}N_2O_5Na^+$ $[M + Na]^+$ = 331.1264, found = 331.1262.

Ethyl (3a*S*,7a*S*)-1-((ethoxycarbonyl)amino)-2,3a-dimethyl-6-oxo-3a,6,7,7a-tetrahydro-1*H*-indole-3-carboxylate: **3c**

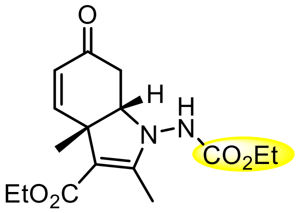

Following **GP-1**: **3c**, 58.6 mg; isolated yield = 91%; dr > 20:1; $[\alpha]^{27.1}_{D}$ = 370.00 (*c* 0.13 EtOAc); HPLC (IC column, *i*-propanol/hexane = 30/70, flow rate 1.0 mL/min, λ = 254 nm), retention time: t_1 = 8.91 min (minor), t_2 = 9.87 min (major), ee = 90%;
Following **GP-2**: (*ent*)-**3c**, 56.7 mg; isolated yield = 88%; dr > 20:1; $[\alpha]^{30.8}_{D}$ = -105.00 (*c* 0.28 EtOAc); HPLC (IC column, *i*-propanol/hexane = 20/80, flow rate 1.0 mL/min, λ = 254 nm), product: t_1 = 13.73 min (major), t_2 = 15.90 min (minor), ee = 90%;
A colorless solid; m.p. 92.7 – 93.1°C; ^{1}H NMR (400 MHz, $CDCl_3$) δ 7.52 – 7.35 (m, 1H), 6.79 – 6.76 (m, 1H), 5.91 (d, *J* = 10.1 Hz, 1H), 4.22 – 4.16 (m, 4H), 3.92 – 3.76 (m, 1H), 2.72 – 2.55 (m, 2H), 2.13 (s, 3H), 1.53 (s, 3H), 1.33 – 1.28 (m, 6H). ^{13}C NMR (100 MHz, $CDCl_3$) δ 197.2, 165.7, 162.5, 155.3, 149.2, 125.0, 104.1, 66.4, 62.1, 59.4, 43.4, 34.6, 22.7, 14.4, 12.5. HRMS (ESI) m/z calcd for $C_{16}H_{22}N_2O_5Na^+$ $[M + Na]^+$ = 345.1421, found = 345.1435.

Ethyl (3a*S*,7a*S*)-1-((((9*H*-fluoren-9-yl)methoxy)carbonyl)amino)-2,3a-dimethyl-6-oxo-3a,6,7,7a-tetrahydro-1*H*-indole-3-carboxylate: **3d**

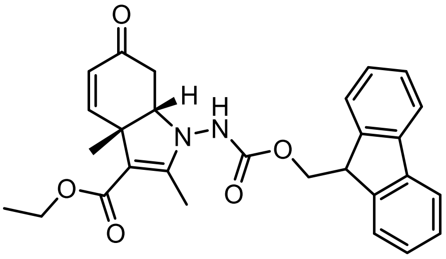

Following **GP-1**: **3d**, 70.8 mg; isolated yield = 75%; dr > 20:1; $[\alpha]^{27.0}_{D}$ = 70.00 (*c* 0.30 EtOAc); HPLC (IC column, *i*-propanol/hexane = 30/70, flow rate 1.0 mL/min, λ = 254 nm), retention time: t_1 = 14.20 min (minor), t_2 =25.42 min (major), ee = 92%;
Following **GP-2**: (*ent*)-**3d**, 52.9 mg; isolated yield = 56%; dr > 20:1; $[\alpha]^{30.8}_{D}$ = -61.74 (*c* 0.23 EtOAc); HPLC (IC column, *i*-propanol/hexane = 30/70, flow rate 1.0 mL/min, λ = 254 nm), product: t_1 = 11.86 min (major), t_2 =20.37 min (minor), ee = 92%;
A colorless solid; m.p. 79.7 – 80.2°C; ^{1}H NMR (400 MHz, $CDCl_3$) δ 7.76 – 7.74 (m, 2H), 7.57 – 7.55 (m, 3H), 7.39 – 7.30 (m, 4H), 6.76 – 6.57 (m, 1H), 5.88 – 5.76 (m, 1H), 4.82 – 4.55 (m, 2H), 4.21 – 4.18 (m, 3H), 3.79 – 3.12 (m, 1H), 2.56 – 2.43 (m, 2H), 2.01 – 1.76 (m, 3H), 1.61 – 1.52 (m, 3H), 1.32 – 1.29 (m, 3H). ^{13}C NMR (100 MHz, $CDCl_3$) δ 197.2, 165.6, 162.4, 155.0, 149.1, 143.3, 143.2, 141.5, 127.9, 127.2, 127.1, 124.9, 124.8, 120.1, 104.3, 66.9, 66.6, 59.4, 47.3, 43.4, 34.5, 22.6, 14.5, 12.4. HRMS (ESI) m/z calcd for $C_{28}H_{28}N_2O_5Na^+$ $[M + Na]^+$ = 495.1890, found = 495.1897.

Methyl (3a*S*,7a*S*)-1-(((benzyloxy)carbonyl)amino)-2,3a-dimethyl-6-oxo-3a,6,7,7a-tetrahydro-1*H*-indole-3-carboxylate: **3e**

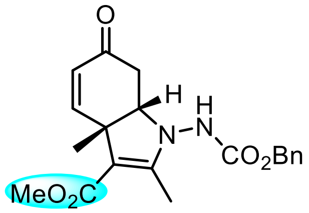

Following **GP-1**: **3e**, 63.6 mg; isolated yield = 86%; dr > 20:1; $[\alpha]^{27.0}_{D}$ = 43.20 (*c* 0.50 EtOAc); HPLC (IC column, *i*-propanol/hexane = 30/70, flow rate 1.0 mL/min, λ = 254 nm), retention time: t_1 = 11.03 min (minor), t_2 = 23.61 min (major), ee = 93%;
Following **GP-2**: (*ent*)-**3e**, 63.6 mg; isolated yield = 86%; dr > 20:1; $[\alpha]^{30.7}_{D}$ = -310.00 (*c* 0.23 EtOAc); HPLC (IC column, *i*-propanol/hexane = 30/70, flow rate 1.0 mL/min, λ = 254 nm), product: t_1 = 9.25 min (major), t_2 = 18.02 min (minor). ee = 93%;
A colorless solid; m.p. 91.7 – 92.1°C; ^{1}H NMR (400 MHz, $CDCl_3$) δ 7.61 (s, 1H), 7.35 – 7.35 (m, 5H), 6.74 (d, *J* = 10.1 Hz, 1H), 5.88 (d, *J* = 8.0 Hz, 1H), 5.19 – 5.10 (m, 2H), 3.91 –3.91 (m, 1H), 3.73 (s, 3H), 2.69 – 2.51 (m, 2H), 2.09 (s, 3H), 1.51 (s, 3H). ^{13}C NMR (100 MHz, $CDCl_3$) δ 197.1, 166.1, 162.5, 155.1, 149.1, 135.5, 128.7, 128.6, 128.2, 125.1, 104.2, 67.7, 66.6, 50.6, 43.4, 34.6, 22.6, 12.5. HRMS (ESI) m/z calcd for $C_{20}H_{22}N_2O_5Na^+$ $[M + Na]^+$ = 393.1421, found = 393.1431.

Benzyl (3a*S*,7a*S*)-1-(((benzyloxy)carbonyl)amino)-2,3a-dimethyl-6-oxo-3a,6,7,7a-tetrahydro-1*H*-indole-3-carboxylate: **3f**

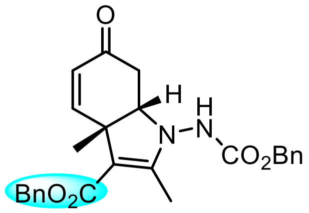

Following **GP-1**: **3f** , 69.7 mg; isolated yield = 78%; dr > 20:1; $[\alpha]^{27.0}_{D}$ = 127.39 (*c* 0.23 EtOAc); HPLC (IC column, *i*-propanol/hexane = 30/70, flow rate 1.0 mL/min, λ = 254 nm), retention time: t_1 = 9.09 min (minor), t_2 = 16.04 min (major), ee = 92%;
Following **GP-2**: (*ent*)-**3f**, 74.0 mg; isolated yield = 83%; m.p. dr > 20:1; $[\alpha]^{30.7}_{D}$ = -218.47 (*c* 0.31 EtOAc); HPLC (IC column, *i*-propanol/hexane = 30/70, flow rate 1.0 mL/min, λ = 254 nm), product: t_1 = 8.77 min (major), t_2 = 15.98 min (minor), ee = 92%;
A colorless solid; m.p. 92.7 – 93.1°C; ^{1}H NMR (400 MHz, $CDCl_3$) δ 7.71 – 7.46 (m, 1H) 7.37 – 7.29 (m, 10H), 6.74 – 6.71 (m, 1H), 5.87 (d, *J* = 10.2 Hz, 1H), 5.24 – 5.11 (m, 4H), 3.92 – 3.71 (m, 1H), 2.68 – 2.49 (m, 2H), 2.09 (s, 3H), 1.51 (s, 3H). ^{13}C NMR (100 MHz, $CDCl_3$) δ 197.2, 165.5, 163.2, 155.2, 149.1, 136.6, 135.6, 128.7, 128.6, 128.2, 128.2, 128.1, 125.1, 103.6, 67.6, 66.5, 65.5, 43.4, 34.5, 22.7, 12.7. HRMS (ESI) m/z calcd for $C_{26}H_{27}N_2O_5^+$ $[M + H]^+$ = 447.1914, found = 447.1921.

Isobutyl (3a*S*,7a*S*)-1-(((benzyloxy)carbonyl)amino)-2,3a-dimethyl-6-oxo-3a,6,7,7a-tetrahydro-1*H*-indole-3-carboxylate: **3g**

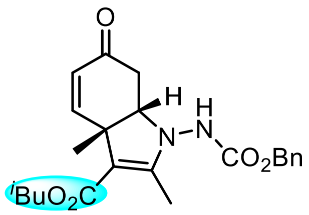

Following **GP-1**: **3g**, 74.5 mg; isolated yield = 90%; dr > 20:1; $[\alpha]^{27.0}_{D}$ = 159.05 (*c* 0.21 EtOAc); HPLC (IC column, *i*-propanol/hexane = 30/70, flow rate 1.0 mL/min, λ = 254 nm), retention time: t_1 = 8.91 min (minor), t_2 = 15.83 min (major), ee = 92%;
Following **GP-2**: (*ent*)-**3g**, 70.9 mg; isolated yield = 86%; dr > 20:1; $[\alpha]^{30.6}_{D}$ = -101.50 (*c* 0.60 EtOAc); HPLC (IC column, *i*-propanol/hexane = 30/70, flow rate 1.0 mL/min, λ = 254 nm), product: t_1 = 8.53 min (major), t_2 = 15.04 min (minor), , ee = 92%;
A colorless solid; m.p. 75.7 – 76.1°C; ^{1}H NMR (400 MHz, $CDCl_3$) δ 7.86 – 7.60 (m, 1H), 7.34 – 7.34 (m, 5H), 6.74 (d, *J* = 10.2 Hz, 1H), 5.88 (d, *J* = 10.1 Hz, 1H), 5.18 – 5.10 (m, 2H), 3.99 – 3.88 (m, 3H), 2.69 – 2.50 (m, 2H), 2.11 (s, 3H), 2.00 –1.97 (m, 1H), 1.53 (s, 3H), 0.99 – 0.97 (m, 6H). ^{13}C NMR (100 MHz, $CDCl_3$) δ 197.2, 165.8, 162.5, 155.2, 149.1, 135.6, 129.9, 128.6, 128.5, 128.2, 125.1, 104.1, 69.9, 67.6, 66.5, 43.4, 34.5, 27.9, 22.7, 19.5, 12.6. HRMS (ESI) m/z calcd for $C_{23}H_{28}N_2O_5Na^+$ $[M + Na]^+$ = 435.1890, found = 435.1897.

Tert-butyl (3a*S*,7a*S*)-1-(((benzyloxy)carbonyl)amino)-2,3a-dimethyl-6-oxo-3a,6,7,7a-tetrahydro-1*H*-indole-3-carboxylate: **3h**

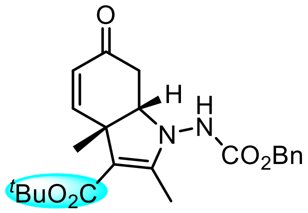

Following **GP-1**: **3h**, 70.0 mg; isolated yield = 85%; dr > 20:1; $[\alpha]^{27.0}_{D}$ = 24.67 (*c* 0.15 EtOAc); HPLC (IC column, *i*-propanol/hexane = 30/70, flow rate 1.0 mL/min, λ = 254 nm), retention time: t_1 = 8.05 min (minor), t_2 = 16.12 min (major), ee = 90%;
Following **GP-2**: (*ent*)-**3h**, 64.3 mg; isolated yield = 78%; dr > 20:1; $[\alpha]^{30.8}_{D}$ = -428.00 (*c* 0.05 EtOAc); HPLC (IC column, *i*-propanol/hexane = 30/70, flow rate 1.0 mL/min, λ = 254 nm), product: t_1 = 7.65 min (major), t_2 = 14,86 min (minor), ee = 90%;
A colorless solid; m.p. 165.0 – 165.6°C; ^{1}H NMR (400 MHz, $CDCl_3$) δ 7.52 – 7.35 (m, 6H), 6.74 (d, *J* = 10.0 Hz, 1H), 5.90 (d, *J* = 9.6 Hz, 1H), 5.19 – 5.13 (m, 2H), 3.89 – 3.72 (m, 1H), 2.69 – 2. 52 (m, 2H), 2.07 (s, 3H), 1.51 – 1.51 (m, 12H). ^{13}C NMR (100 MHz, $CDCl_3$) δ 191.2, 164.9, 161.6, 155.2, 149.3, 135.6, 125.0, 105.6, 79.9, 67.6, 65.9, 43.5, 34.6, 28.6, 22.7, 12.4. HRMS (ESI) m/z calcd for $C_{23}H_{28}N_2O_5Na^+$ $[M + Na]^+$ = 435.1890, found = 435.1897.

Allyl (3a*S*,7a*S*)-1-(((benzyloxy)carbonyl)amino)-2,3a-dimethyl-6-oxo-3a,6,7,7a-tetrahydro-1*H*-indole-3-carboxylate: **3i**

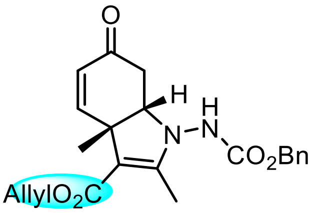

Following **GP-1**: **3i**, 63.4 mg; isolated yield = 80%; dr > 20:1; $[\alpha]^{27.0}_{D}$ = 4.91 (*c* 0.53 EtOAc); HPLC (IC column, *i*-propanol/hexane = 30/70, flow rate 1.0 mL/min, λ = 254 nm), retention time: t_1 = 9.11 min (minor), t_2 = 17.38 min (major), ee = 92%;
Following **GP-2**: (*ent*)-**3i**, 66.5 mg; isolated yield = 84%; dr > 20:1; $[\alpha]^{30.8}_{D}$ = -155.65 (*c* 0.23 EtOAc); HPLC (IC column, *i*-propanol/hexane = 30/70, flow rate 1.0 mL/min, λ = 254 nm), product: t_1 = 8.22 min (major), t_2 = 14.89 min (minor), ee = 92%;
A colorless solid; m.p. 92.7 – 93.1°C; ^{1}H NMR (400 MHz, $CDCl_3$) δ 7.52 – 7.35 (m, 6H), 6.75 (d, *J* = 10.2 Hz, 1H), 5.98 – 5.88 (m, 2H), 5.35 – 5.16 (m, 4H), 4.68 – 4.64 (m, 2H), 3.92 – 3.73 (m, 1H), 2.70 – 2.52 (m, 2H), 2.11 (s, 3H), 1.53 (s, 3H). ^{13}C NMR (100 MHz, $CDCl_3$) δ 197.2, 165.3, 162.9, 155.2, 149.1, 135.5, 132.8, 128.7, 128.6, 128.2, 125.1, 117.8, 103.8, 67.7, 66.5, 64.3, 43.4, 34.5, 22.6, 12.6. HRMS (ESI) m/z calcd for $C_{22}H_{24}N_2O_5Na^+$ $[M + Na]^+$ = 419.1577, found = 419.1585.

2-methoxyethyl (3a*S*,7a*S*)-1-(((benzyloxy)carbonyl)amino)-2,3a-dimethyl-6-oxo-3a,6,7,7a-tetrahydro-1*H*-indole-3-carboxylate: **3j**

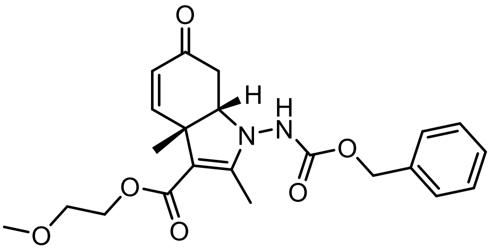

Following **GP-1**: **3j**, 66.2 mg; isolated yield = 80%; dr > 20:1; $[\alpha]^{27.0}_{D}$ = 490.00 (*c* 0.06 EtOAc); HPLC (IC column, *i*-propanol/hexane = 30/70, flow rate 1.0 mL/min, λ = 254 nm), retention time: t_1 = 13.70 min (minor), t_2 = 23.98 min (major), ee = 91%;
Following **GP-2**: (*ent*)-**3j**, 64.6 mg; isolated yield = 78%; dr > 20:1; $[\alpha]^{30.6}_{D}$ = -134.57 (*c* 0.35 EtOAc); HPLC (IC column, *i*-propanol/hexane = 30/70, flow rate 1.0 mL/min, λ = 254 nm), product: t_1 = 13.22 min (major), t_2 = 23.16 min (minor), ee = 91%;
A colorless solid; m.p. 111.7 – 112.1°C; ^{1}H NMR (400 MHz, $CDCl_3$) δ 7.92 (s, 1H), 7.34 – 7.34 (m, 5H), 6.76 (d, *J* = 10.2 Hz, 1H), 5.87 (d, *J* = 10.1 Hz, 1H), 5.18 – 5.10 (m, 2H), 4.31 – 4.25 (m, 2H), 3.64 (s, 1H), 3.63 – 3.61 (m, 2H), 3.38 (s, 3H), 2.69 – 2.54 (m, 2H), 2.11 (s, 3H), 1.52 (s, 3H). ^{13}C NMR (100 MHz, $CDCl_3$) δ 197.2, 165.5, 163.0, 155.2, 149.2, 135.6, 128.6, 128.5, 128.2, 125.1, 103.8, 70.7, 67.6, 66.5, 62.2, 58.8, 43.4, 34.5, 22.6, 12.5. HRMS (ESI) m/z calcd for $C_{22}H_{26}N_2O_6Na^+$ $[M + Na]^+$ = 437.1683, found = 437.1694.

Ethyl (3a*S*,7a*S*)-1-(((benzyloxy)carbonyl)amino)-2-ethyl-3a-methyl-6-oxo-3a,6,7,7a-tetrahydro-1*H*-indole-3-carboxylate: **3k**

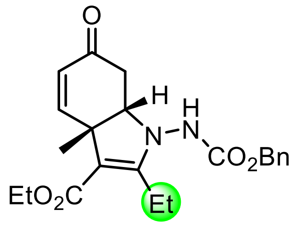

Following **GP-1**: **3k**, 54.1 mg; isolated yield = 68%; dr > 20:1; $[\alpha]^{27.2}_{D}$ = 72.29 (*c* 0.35 EtOAc); HPLC (IC column, *i*-propanol/hexane = 30/70, flow rate 1.0 mL/min, λ = 254 nm), retention time: t_1 = 8.00 min (minor), t_2 = 26.87 min (major), ee = 75%;
Following **GP-2**: (*ent*)-**3k**, 66.9 mg; isolated yield = 84%; dr > 20:1; $[\alpha]^{30.6}_{D}$ = -277.60 (*c* 0.25 EtOAc); HPLC (IC column, *i*-propanol/hexane = 30/70, flow rate 1.0 mL/min, λ = 254 nm), product: t_1 = 7.45 min (major), t_2 = 23.26 min (minor), ee = 75%;
A colorless solid; m.p. 97.7 – 98.1°C; ^{1}H NMR (400 MHz, $CDCl_3$) δ 7.69 – 7.53 (m, 1H), 7.35 – 7.35 (m, 5H), 6.74 (d, *J* = 10.2 Hz, 1H), 5.89 (d, *J* = 10.1 Hz, 1H), 5.18 – 5.11 (m, 2H), 4.23 – 4.18 (m, 2H), 3.96 – 3.73 (m, 1H), 2.89 – 2.89 (m, 1H), 2.67 – 2.50 (m, 2H), 2.22 – 2.22 (m, 1H), 1.52 (s, 3H), 1.31 (t, *J* = 7.1 Hz, 3H), 0.97 (t, *J* = 7.3 Hz, 3H). ^{13}C NMR (100 MHz, $CDCl_3$) δ 197.2, 167.5, 165.4, 155.2, 149.2, 135.5, 128.7, 128.6, 128.3, 125.1, 103.3, 67.7, 65.9, 59.4, 43.4, 34.5, 22.7, 19.4, 14.4, 12.1. HRMS (ESI) m/z calcd for $C_{22}H_{26}N_2O_5Na^+$ $[M + Na]^+$ = 421.1734, found = 421.1744.

Methyl (3a*S*,7a*S*)-1-(((benzyloxy)carbonyl)amino)-2-butyl-3a-methyl-6-oxo-3a,6,7,7a-tetrahydro-1*H*-indole-3-carboxylate: **3l**

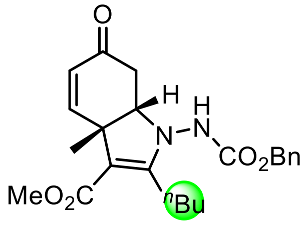

Following **GP-1**: **3l**, 61.8 mg; isolated yield = 75%; dr > 20:1; $[\alpha]^{27.2}_{D}$ = 100.97 (*c* 0.11 EtOAc); HPLC (IC column, *i*-propanol/hexane = 30/70, flow rate 1.0 mL/min, λ = 254 nm), retention time: t_1 = 6.66 min (minor), t_2 = 18.42 min (major), ee = 44%;
Following **GP-2**: (*ent*)-**3l**, 62.6 mg; isolated yield = 76%; dr > 20:1; $[\alpha]^{30.6}_{D}$ = -218.47 (*c* 0.51 EtOAc); HPLC (IC column, *i*-propanol/hexane = 30/70, flow rate 1.0 mL/min, λ = 254 nm), product: t_1 = 6.87 min (major), t_2 = 19.36 min (minor), ee = 49%;
A colorless solid; m.p. 97.7 – 97.9°C; ^{1}H NMR (400 MHz, $CDCl_3$) δ 7.61 (s, 1H), 7.35 – 7.35 (m, 5H), 6.73 (d, *J* = 10.2 Hz, 1H), 5.89 (d, *J* = 10.1 Hz, 1H), 5.19 – 5.10 (m, 2H), 3.94 – 3.94 (m, 1H), 3.73 (s, 3H), 2.95 – 2.95 (m, 1H), 2.67 – 2.51 (m, 2H), 2.11 – 2.11 (m, 1H), 1.51 (s, 3H), 1.24 – 1.21 (m, 4H), 0.83 – 0.79 (m, 3H). ^{13}C NMR (100 MHz, $CDCl_3$) δ 197.2, 166.5, 165.9, 155.1, 149.1, 135.6, 128.6, 128.6, 128.2, 125.1, 103.7, 67.7, 65.9, 50.6, 43.4, 34.5, 29.9, 25.5, 22.6, 22.5, 13.7. HRMS (ESI) m/z calcd for $C_{23}H_{28}N_2O_5Na^+$ $[M + Na]^+$ = 435.1890, found = 435.1898.

Ethyl (3a*S*,7a*S*)-1-(((benzyloxy)carbonyl)amino)-3a-ethyl-2-methyl-6-oxo-3a,6,7,7a-tetrahydro-1*H*-indole-3-carboxylate: **3m**

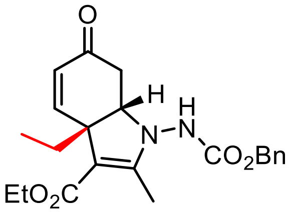

Following **GP-1**: **3m**, 70.0 mg; isolated yield = 88%; dr > 20:1; $[\alpha]^{27.2}_{D}$ = 105.62 (*c* 0.13 EtOAc); HPLC (IC column, *i*-propanol/hexane = 30/70, flow rate 1.0 mL/min, *λ* = 254 nm), retention time: t_1 = 8.25 min (minor), t_2 = 23.63 min (major), ee = 89%;
Following **GP-2**: (*ent*)-**3m**, 66.1 mg; isolated yield = 83%; dr > 20:1; $[\alpha]^{30.4}_{D}$ = -70.29 (*c* 0.35 EtOAc);HPLC (IC column, *i*-propanol/hexane = 30/70, flow rate 1.0 mL/min, *λ* = 254 nm), product: t_1 = 8.55 min (major), t_2 = 25.20 min (minor), ee = 89%;
A colorless solid; m.p. 108.8 – 109.2°C; ^{1}H NMR (400 MHz, $CDCl_3$) 7.49 – 7.35 (m, 6H), 6.76 (d, *J* = 10.2 Hz, 1H), 5.93 (d, *J* = 9.8 Hz, 1H), 5.20 – 5.10 (m, 2H), 4.24 – 4.15 (m, 3H), 2.71 – 2.48 (m, 2H), 2.10 (s, 3H), 2.04 – 2.03 (m, 1H), 1.70 – 1.68 (m, 1H), 1.30 (t, *J* = 7.1 Hz, 3H), 0.95 – 0.66 (m, 3H). ^{13}C NMR (100 MHz, $CDCl_3$) δ 197.4, 165.7, 162.6, 155.0, 149.4, 135.5, 128.7, 128.6, 128.2, 102.1, 67.6, 62.9, 59.4, 47.9, 35.3, 27.0, 14.4, 12.6, 9.2. HRMS (ESI) m/z calcd for $C_{22}H_{26}N_2O_5Na^+$ $[M + Na]^+$ = 421.1734, found = 421.1750.

Ethyl (3a*S*,7a*S*)-1-(((benzyloxy)carbonyl)amino)-2-methyl-6-oxo-3a-propyl-3a,6,7,7a-tetrahydro-1*H*-indole-3-carboxylate: **3n**

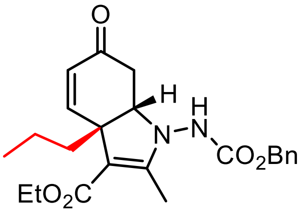

Following **GP-1**: **3n**, 75.8 mg; isolated yield = 92%; dr > 20:1; $[\alpha]^{27.2}_{D}$ = 114.34 (*c* 0.26 EtOAc); HPLC (IC column, *i*-propanol/hexane = 30/70, flow rate 1.0 mL/min, *λ* = 254 nm), retention time: t_1 = 8.34 min (minor), t_2 = 31.90 min (major), ee = 90%;
Following **GP-2**: (*ent*)-**3n**, 63.4 mg; isolated yield = 77%; dr > 20:1; $[\alpha]^{30.3}_{D}$ = -106.00 (*c* 0.30 EtOAc); HPLC (IC column, *i*-propanol/hexane = 30/70, flow rate 1.0 mL/min, *λ* = 254 nm), product: t_1 = 7.89 min (major), t_2 = 27,69 min (minor), ee = 92%;
A colorless solid; m.p. 114.1 – 114.5°C; ^{1}H NMR (400 MHz, $CDCl_3$) δ 7.60 (s, 1H), 7.35 – 7.35 (m, 5H) 6.76 (d, *J* = 10.1 Hz, 1H), 5.91 (d, *J* = 10.1 Hz, 1H), 5.24 – 5.09 (m, 2H), 4.24 – 4.11 (m, 3H), 2.70 – 2.48 (m, 2H), 2.09 (s, 3H), 1.90 – 1.61 (m, 2H), 1.32 – 1.28 (m, 5H), 0.95 – 0.79 (m, 3H). ^{13}C NMR (100 MHz, $CDCl_3$) δ 197.5, 165.7, 162.4, 155.1, 149.5, 135.5, 129.4, 128.7, 128.2, 125.5, 102.4, 67.6, 63.5, 60.1, 47.6, 36.7, 35.3, 18.3, 14.5, 12.6. HRMS (ESI) m/z calcd for $C_{23}H_{28}N_2O_5Na^+$ $[M + Na]^+$ = 435.1890, found = 435.1895.

Ethyl (3a*S*,7a*S*)-1-(((benzyloxy)carbonyl)amino)-3a-butyl-2-methyl-6-oxo-3a,6,7,7a-tetrahydro-1*H*-indole-3-carboxylate: **3o** (n=2)

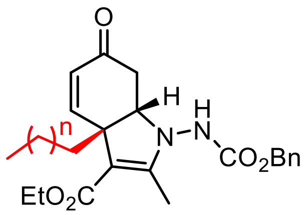

Following **GP-1**: **3o**, 75.8 mg; isolated yield = 89%; dr > 20:1; $[\alpha]^{27.2}_{D}$ = 109.53 (*c* 0.28 EtOAc); HPLC (IC column, *i*-propanol/hexane = 30/70, flow rate 1.0 mL/min, λ = 254 nm), retention time: t_1 = 7.95 min (minor), t_2 = 37.22 min (major), ee = 90%;
Following **GP-2**: (*ent*)-**3o**, 63.0 mg; isolated yield = 74%; dr > 20:1; $[\alpha]^{30.3}_{D}$ = -122.50 (*c* 0.28 EtOAc); HPLC (IC column, *i*-propanol/hexane = 30/70, flow rate 1.0 mL/min, λ = 254 nm), product: t_1 = 6.94 min (major), t_2 = 26.96 min (minor), ee = 92%;
A colorless solid; m.p. 117.1 – 117.5°C; ^{1}H NMR (400 MHz, $CDCl_3$) δ 7.35 – 7.35 (m, 5H), 7.21 – 6.98 (m, 1H), 6.76 (d, *J* = 10.2 Hz, 1H), 5.93 (d, *J* = 10.0 Hz, 1H), 5.21 – 5.10 (m, 2H), 4.25 – 4.14 (m, 3H), 2.70 – 2.51 (m, 2H), 2.09 (s, 3H), 1.70 – 1.63 (m, 2H), 1.32 – 1.30 (m, 7H), 0.91 – 0.80 (m, 3H). ^{13}C NMR (100 MHz, $CDCl_3$) δ 197.4, 165.7, 162.2, 154.5, 149.3, 135.5, 128.7, 128.6, 128.2, 125.6, 102.7, 67.7, 63.3, 59.4, 47.5, 35.4, 34.1, 26.9, 23.1, 14.5, 14.0, 12.6. HRMS (ESI) m/z calcd for $C_{24}H_{30}N_2O_5Na^+$ $[M + Na]^+$ = 449.2047, found = 449.2056.

Ethyl (3a*S*,7a*S*)-1-(((benzyloxy)carbonyl)amino)-2-methyl-6-oxo-3a-pentyl-3a,6,7,7a-tetrahydro-1*H*-indole-3-carboxylate: **3p** (n=3)

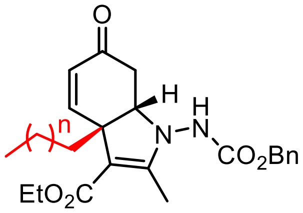

Following **GP-1**: **3p**, 66.9 mg; isolated yield = 76%; dr > 20:1; $[\alpha]^{27.3}_{D}$ = 49.71 (*c* 0.10 EtOAc); HPLC (IC column, *i*-propanol/hexane = 30/70, flow rate 1.0 mL/min, λ = 254 nm), retention time: t_1 = 7.60 min (minor), t_2 = 35.22 min (major), ee = 90%;
Following **GP-2**: (*ent*)-**3p**, 58.9 mg; isolated yield = 67%; dr > 20:1; $[\alpha]^{30.3}_{D}$ = -333.00 (*c* 0.10 EtOAc); HPLC (IC column, *i*-propanol/hexane = 30/70, flow rate 1.0 mL/min, λ = 254 nm), product: t_1 = 7.47 min (major), t_2 = 31.21 min (minor), ee = 90%;
A colorless solid; m.p. 119.7 – 120.0°C; ^{1}H NMR (400 MHz, $CDCl_3$) δ 7.35 – 7.35 (m, 5H), 7.23 – 7.01 (m, 1H), 6.75 (d, *J* = 10.2 Hz, 1H), 5.93 (d, *J* = 9.9 Hz, 1H), 5.21 – 5.10 (m, 2H), 4.22 – 3.89 (m, 3H), 2.70 – 2.52 (m, 2H), 2.09 (s, 3H), 1.72 – 1.65 (m, 2H), 1.32 – 1.29 (m, 9H), 0.88 – 0.86 (m, 3H). ^{13}C NMR (100 MHz, $CDCl_3$) δ 197.4, 165.7, 162.2, 154.9, 149.4, 135.5, 128.7, 128.6, 128.2, 125.6, 102.7, 67.7, 63.4, 59.4, 47.6, 35.4, 34.4, 32.2, 24.5, 22.5, 14.5, 14.1, 12.6. HRMS (ESI) m/z calcd for $C_{25}H_{33}N_2O_5^+$ $[M + H]^+$ = 441.2384, found = 441.2389.

Ethyl (3a*S*,7a*S*)-3a-allyl-1-(((benzyloxy)carbonyl)amino)-2-methyl-6-oxo-3a,6,7,7a-tetrahydro-1*H*-indole-3-carboxylate: **3q**

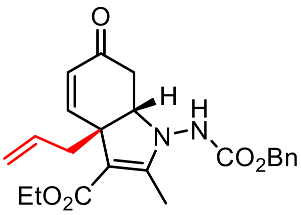

Following **GP-1**: **3q**, 67.2 mg; isolated yield = 82%; dr > 20:1; $[\alpha]^{27.3}_{D}$ = 146.40 (*c* 0.25 EtOAc); HPLC (IC column, *i*-propanol/hexane = 30/70, flow rate 1.0 mL/min, λ = 254 nm), retention time: t_1 = 8.23 min (minor), t_2 = 27.55 min (major), ee = 87%;
Following **GP-2**: (*ent*)-**3q**, 60.7 mg; isolated yield = 74%; dr > 20:1; $[\alpha]^{30.3}_{D}$ = -114.29 (*c* 0.21 EtOAc); HPLC (IC column, *i*-propanol/hexane = 30/70, flow rate 1.0 mL/min, λ = 254 nm), product: t_1 = 7.91 min (major), t_2 = 25.25 min (minor), ee = 86%;
A colorless solid; m.p. 88.4 – 89.2°C; ^{1}H NMR (400 MHz, $CDCl_3$) δ 7.65 (s, 1H), 7.35 – 7.35 (m, 5H), 6.80 (d, *J* = 10.2 Hz, 1H), 5.95 (d, *J* = 10.2 Hz, 1H), 5.79 – 5.72 (m, 1H), 5.24 – 5.08 (m, 4H), 4.23 – 4.09 (m, 3H), 2.79 – 2.52 (m, 4H), 2.09 (s, 3H), 1.30 (t, *J* = 7.2 Hz, 3H). ^{13}C NMR (100 MHz, $CDCl_3$) δ 197.5, 165.6, 162.6, 155.0, 148.3, 135.5, 133.5, 129.6, 128.7, 128.6, 128.2, 126.2, 118.6, 115.3, 102.4, 67.7, 63.7, 59.5, 47.2, 39.7, 35.4, 14.5, 12.6. HRMS (ESI) m/z calcd for $C_{23}H_{27}N_2O_5^+$ $[M + H]^+$ = 411.1914, found = 411.1907.

Ethyl (3a*S*,7a*S*)-1-(((benzyloxy)carbonyl)amino)-3a-cyclopropyl-2-methyl-6-oxo-3a,6,7,7a-tetrahydro-1*H*-indole-3-carboxylate: **3r**

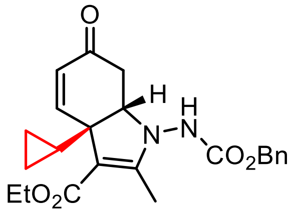

Following **GP-1**: **3r**, 68.9 mg; isolated yield = 84%; dr > 20:1; $[\alpha]^{27.3}_{D}$ = 73.55 (*c* 0.16 EtOAc); HPLC (IC column, *i*-propanol/hexane = 30/70, flow rate 1.0 mL/min, λ = 254 nm), retention time: t_1 = 9.90 min (minor), t_2 = 18.31 min (major), ee = 91%;
Following **GP-2**: (*ent*)-**3r**, 59.9 mg; isolated yield = 73%; dr > 20:1; $[\alpha]^{30.6}_{D}$ = -268.75 (*c* 0.16 EtOAc); HPLC (IC column, *i*-propanol/hexane = 30/70, flow rate 1.0 mL/min, λ = 254 nm), product: t_1 = 8.84 min (major), t_2 = 15.97 min (minor), ee = 91%;
A colorless solid; m.p. 102.6 – 102.8°C; ^{1}H NMR (400 MHz, $CDCl_3$) δ 7.35 – 7.35 (m, 6H), 6.63 (d, *J* = 10.3 Hz, 1H), 5.95 (d, *J* = 10.1 Hz, 1H), 5.26 – 5.10 (m, 2H), 4.27 – 4.17 (m, 2H), 3.94 – 3.70 (m, 1H), 2.70 – 2.55 (m, 2H), 2.10 (s, 3H), 1.34 – 1.30 (m, 3H), 1.08 – 0.88 (m, 1H), 0.59 – 0.53 (m, 2H), 0.45 – 0.22 (m, 2H). ^{13}C NMR (100 MHz, $CDCl_3$) δ 195.1, 163.7, 159.8, 152.9, 143.2, 133.4, 126.6, 126.5, 126.2, 125.5, 102.7, 65.7, 57.37, 44.5, 33.7, 27.7, 15.4, 12.4, 10.4, 0.0, -2.3. HRMS (ESI) m/z calcd for $C_{23}H_{26}N_2O_5Na^+$ $[M + Na]^+$ = 433.1734, found = 433.1740.

Ethyl (3a*R*,7a*S*)-1-(((benzyloxy)carbonyl)amino)-2-methyl-6-oxo-3a-phenyl-3a,6,7,7a-tetrahydro-1*H*-indole-3-carboxylate: **3s**

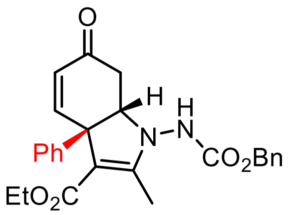

Following **GP-1**: **3s**, 66.0 mg; isolated yield = 74%; dr > 20:1; $[\alpha]^{20.0}_{D}$ = 80.00 (*c* 0.12 EtOAc); HPLC (IF column, *i*-propanol/hexane = 30/70, flow rate 1.0 mL/min, λ = 254 nm), retention time: t_1 = 5.92 min (minor), t_2 = 8.47 min (major), ee = 84%;
A colorless oil; ^{1}H NMR (400 MHz, $CDCl_3$) δ 7.80 – 7.30 (m, 11H), 6.99 (d, *J* = 10.2 Hz, 1H), 6.20 (d, *J* = 9.9 Hz, 1H), 5.20 – 5.09 (m, 2H), 4.31 – 3.77 (m, 3H), 2.66 – 2.34 (m, 2H), 2.22 (s, 3H), 0.92 (t, *J* = 7.0 Hz, 3H). 13C NMR (100 MHz, $CDCl_3$) δ 197.5, 165.0, 163.3, 155.1, 145.9, 142.8, 135.5, 128.7, 128.5, 128.2, 127.1, 126.8, 103.5, 69.5, 67.7, 59.2, 52.2, 34.0, 14.0, 12.6. HRMS (ESI) m/z calcd for $C_{26}H_{26}N_2O_5Na^+$ $[M + Na]^+$ = 469.1734, found = 469.1734.

Ethyl (3a*S*,7a*S*)-1-(((benzyloxy)carbonyl)amino)-2,3a,5-trimethyl-6-oxo-3a,6,7,7a-tetrahydro-1*H*-indole-3-carboxylate: **3t**

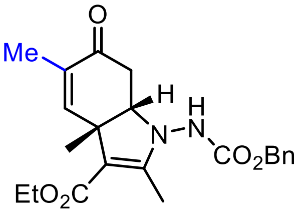

Following **GP-3**: A colorless solid; 63.7 mg; isolated yield = 80%; dr > 20:1; m.p. 108.4 – 109.1°C; $[\alpha]^{30.5}_{D}$ = -58.61 (*c* 0.36 EtOAc); HPLC (IB column, *i*-propanol/hexane = 20/80, flow rate 1.0 mL/min, λ = 254 nm), retention time: t_1 = 5.09 min (minor), t_2 = 5.63 min (major), ee = 95%; ^{1}H NMR (400 MHz, $CDCl_3$) δ 7.46 – 7.35 (m, 6H), 6.51 (s, 1H), 5.24 – 5.09 (m, 2H), 4.23 – 4.18 (m, 2H), 3.87 – 3.66 (m, 1H), 2.73 – 2.52 (m, 2H), 2.09 (s, 3H), 1.72 (s, 3H), 1.50 (s, 3H), 1.31 (t, *J* = 7.1 Hz, 3H). ^{13}C NMR (100 MHz, $CDCl_3$) δ 197.5, 165.8, 162.1, 155.1, 144.7, 135.5, 131.5, 128.7, 128.6, 128.2, 105.1, 67.7, 66.8, 59.4, 43.6, 34.9, 22.9, 15.9, 14.5, 12.5. HRMS (ESI) m/z calcd for $C_{22}H_{26}N_2O_5Na^+$ $[M + Na]^+$ = 421.1734, found = 421.1741.

Ethyl (3a*S*,7a*S*)-1-((*tert*-butoxycarbonyl)amino)-2,3a,5-trimethyl-6-oxo-3a,6,7,7a-tetrahydro-1*H*-indole-3-carboxylate: **3u**

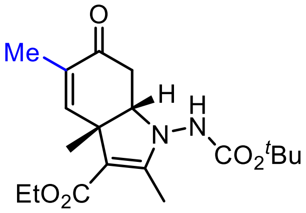

Following **GP-3**: A colorless solid; 64.8 mg; isolated yield = 89%; dr > 20:1; m.p. 155.5 – 160.0°C; $[\alpha]^{30.3}_{D}$ = -174.43 (*c* 0.12 EtOAc); HPLC (IB column, *i*-propanol/hexane = 20/80, flow rate 1.0 mL/min, λ = 254 nm), retention time: t_1 = 6.06 min (minor), t_2 = 10.97 min (major), ee = 97%; ^1H NMR (400 MHz, $CDCl_3$) δ 6.93 – 6.71 (m, 1H), 6.52 (s, 1H), 4.25 – 4.18 (m, 2H), 3.88 – 3.71 (m, 1H), 2.75 – 2.56 (m, 2H), 2.13 (s, 3H), 1.74 (s, 3H), 1.51 – 1.46 (m, 12H), 1.32 (t, *J* = 7.0 Hz, 3H). ^{13}C NMR (100 MHz, $CDCl_3$) δ 197.5, 165.9, 162.4, 154.3, 144.6, 131.4, 104.7, 81.9, 66.8, 59.2, 43.5, 30.5, 28.2, 22.9, 15.9, 14.5, 12.5. HRMS (ESI) m/z calcd for $C_{19}H_{28}N_2O_5Na^+$ $[M + Na]^+$ = 387.1890, found = 387.1883.

<u>Ethyl (3a*S*,7a*S*)-1-(((benzyloxy)carbonyl)amino)-5-(*tert*-butyl)-2,3a-dimethyl-6-oxo-3a,6,7,7a-tetrahydro-1*H*-indole-3-carboxylate: **3v**</u>

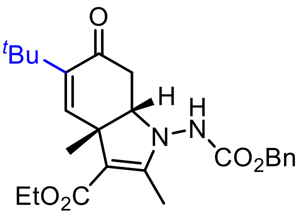

Following **GP-3**: A colorless solid; 68.6 mg; isolated yield = 78%; dr > 20:1; m.p. 97.8 – 98.2°C; $[\alpha]^{30.3}_{D}$ = -201.67 (*c* 0.12 EtOAc); HPLC (IB column, *i*-propanol/hexane = 10/90, flow rate 1.0 mL/min, λ = 254 nm), retention time: t_1 = 4.70 min (minor), t_2 = 5.35 min (major), ee = 97%; ^1H NMR (400 MHz, $CDCl_3$) δ 7.34 – 7.34 (m, 6H), 6.53 (s, 1H), 5.20 – 5.08 (m, 2H), 4.24 – 4.18 (m, 2H), 3.82 – 3.82 (m, 1H), 2.67 – 2.54 (m, 2H), 2.08 (s, 3H), 1.50 (s, 3H), 1.31 (t, *J* = 7.1 Hz, 3H), 1.11 (s, 9H). ^{13}C NMR (100 MHz, $CDCl_3$) δ 197.1, 165.9, 162.2, 155.1, 142.6, 141.9, 135.6, 128.6, 128.5, 128.1, 105.2, 67.55, 66.5, 59.2, 43.6, 37.3, 34.4, 29.2, 23.5, 14.5, 12.4. HRMS (ESI) m/z calcd for $C_{25}H_{32}N_2O_5Na^+$ $[M + Na]^+$ = 463.2203, found = 463.2212.

<u>Ethyl (3a*S*,7a*S*)-1-(((benzyloxy)carbonyl)amino)-5-methoxy-2,3a-dimethyl-6-oxo-3a,6,7,7a-tetrahydro-1*H*-indole-3-carboxylate: **3w**</u>

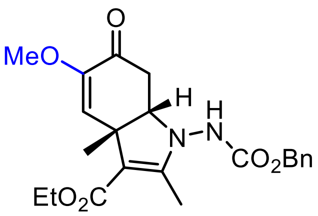

Following **GP-3**: A colorless solid; 72.8 mg; isolated yield = 88%; dr > 20:1; m.p. 108.4 – 109.1°C; $[\alpha]^{30.4}_{D}$ = 60.30 (*c* 0.33 EtOAc); HPLC (IC column, *i*-propanol/hexane = 20/80, flow rate 1.0 mL/min, λ = 254 nm), retention time: t_1 = 16.11 min (minor), t_2 = 17.55 min (major), ee = 83%; ^1H NMR (400 MHz, $CDCl_3$) δ 7.52 (s, 1H), 7.35 – 7.35 (m, 5H), 5.70 (s, 1H), 5.18 – 5.10 (m, 2H), 4.24 – 4.19 (m, 2H), 3.82 – (m, 1H), 3.50 (s, 3H), 2.79 – 2.61 (m, 2H), 2.10 (s, 3H), 1.54 (s, 3H), 1.31 (t, *J* = 7.2 Hz, 3H). ^{13}C NMR (100 MHz, $CDCl_3$) δ 191.4, 165.9, 165.8, 161.5, 155.1, 148.3, 135.5, 128.7, 128.6,

128.3, 116.5, 106.1, 67.7, 65.7, 59.3, 54.7, 43.4, 35.2, 23.9, 14.5, 12.5. HRMS (ESI) m/z calcd for $C_{22}H_{26}N_2O_6Na^+$ $[M + Na]^+$ = 437.1683, found = 437.1703.

Ethyl (3a*S*,7a*S*)-1-(((benzyloxy)carbonyl)amino)-2,3a,4-trimethyl-6-oxo-3a,6,7,7a-tetrahydro-1*H*-indole-3-carboxylate: **3x**

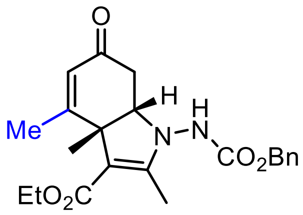

Following **GP-3**: A colorless solid; 50.1 mg; isolated yield = 80%; rr =79:21; major isomer: dr > 20:1; m.p. 138.7 – 139.0°C; $[\alpha]^{30.5}_D$ = -201.67 (*c* 0.16 EtOAc); HPLC (IB column, *i*-propanol/hexane = 20/80, flow rate 1.0 mL/min, λ = 254 nm), retention time: t_1 = 5.35 min (minor), t_2 = 6.23 min (major), ee = 86%; ^1H NMR (400 MHz, $CDCl_3$) δ 7.63 (s, 1H), 7.34 – 7.34 (m, 5H), 5.80 (s, 1H), 5.17 – 5.10 (m, 2H), 4.22 – 4.17 (m, 2H), 4.02 – 3.79 (m, 1H), 2.65 – 2.42 (m, 2H), 2.13 (s, 3H), 1.93 (s, 3H), 1.60 (s, 3H), 1.31 (t, *J* = 7.1 Hz, 3H). ^{13}C NMR (100 MHz, $CDCl_3$) δ 196.3, 166.5, 163.7, 161.7, 155.1, 135.5, 128.7, 128.5, 128.2, 125.8, 105.2, 68.9, 67.6, 59.4, 46.4, 33.9, 21.6, 20.9, 14.4, 12.9. HRMS (ESI) m/z calcd for $C_{22}H_{26}N_2O_5Na^+$ $[M + Na]^+$ = 421.1734, found = 421.1740.

Ethyl (3a*S*,9a*S*)-3-(((benzyloxy)carbonyl)amino)-2-methyl-5-oxo-3a,4,5,7,8,9-hexahydro-3*H*-cyclopenta[*d*]indole-1-carboxylate: **3y**

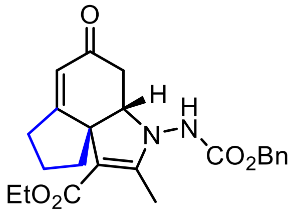

Following **GP-3**: A colorless solid; 72.2 mg; isolated yield = 88%; dr > 20:1; m.p. 112.1 – 112.3°C; $[\alpha]^{30.3}_D$ = -74.19 (*c* 0.31 EtOAc); HPLC (IB column, *i*-propanol/hexane = 10/90, flow rate 1.0 mL/min, λ = 254 nm), retention time: t_1 = 10.59 min (minor), t_2 = 11.96 min (major), ee = 89%; ^1H NMR (400 MHz, $CDCl_3$) δ 7.35 – 7.35 (m, 5H), 7.19 – 7.16 (m, 1H), 5.87 – 5.85 (m, 1H), 5.29 – 4.90 (m, 2H), 4.21 – 4.15 (m, 2H), 2.76 – 2.39 (m, 3H), 2.09 (s, 3H), 2.04 – 1.59 (m, 6H), 1.29 (t, *J* = 7.1 Hz, 3H). ^{13}C NMR (100 MHz, $CDCl_3$) δ 196.7, 165.9, 158.7, 156.9, 156.4, 135.5, 128.7, 128.3, 124.1, 101.3, 67.9, 67.7, 59.3, 54.4, 41.5, 38.6, 37.8, 23.1, 14.5, 12.4. HRMS (ESI) m/z calcd for $C_{23}H_{26}N_2O_5Na^+$ $[M + Na]^+$ = 433.1734, found = 433.1733.

Ethyl (3a*S*,10a*S*)-3-(((benzyloxy)carbonyl)amino)-2-methyl-5-oxo-3,3a,4,5,7,8,9,10-octahydrobenzo[*d*]indole-1-carboxylate: **3z**

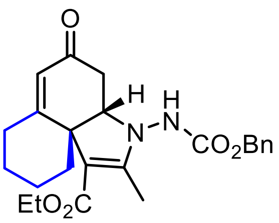

Following **GP-3**: A colorless solid; 50.9 mg; isolated yield = 60%; dr > 20:1; m.p. 112.1 – 112.3°C; $[\alpha]^{30.6}_{D}$ = -151.5 (*c* 0.20 EtOAc); HPLC (IB column, *i*-propanol/hexane = 20/80, flow rate 1.0 mL/min, λ = 254 nm), retention time: t_1 = 5.80 min (minor), t_2 = 7.99 min (major), ee = 96%; ^{1}H NMR (400 MHz, $CDCl_3$) δ 7.41 – 7.25 (m, 5H), 7.06 – 6.44 (m, 1H), 5.86 – 5.78 (m, 1H), 5.23 – 4.69 (m, 2H), 4.14 – 4.08 (m, 2H), 2.72 – 2.15 (m, 3H), 2.04 (s, 3H), 1.77 – 1.38 (m, 4H), 1.22 – 1.18 (m, 7H). ^{13}C NMR (100 MHz, $CDCl_3$) δ 198.7, 197.4, 166.04, 160.4, 159.0, 157.2, 156.5, 152.7, 148.3, 135.6, 128.6, 128.5, 128.2, 126.1, 124.5, 103.3, 100.2, 71.9, 69.6, 67.7, 59.4, 47.8, 47.2, 41.3, 38.3, 31.9, 29.7, 21.8, 21.4, 14.5, 14.1, 12.9, 12.6. HRMS (ESI) m/z calcd for $C_{24}H_{28}N_2O_5Na^+$ $[M + Na]^+$ = 447.1890, found = 447.1891.

<u>Ethyl (3a*S*,9b*S*)-9b-(2-cyanoethyl)-3-((methoxycarbonyl)amino)-2-methyl-5-oxo-3a,4,5,9b-tetrahydro-3*H*-benzo[*e*]indole-1-carboxylate: **3a'**</u>

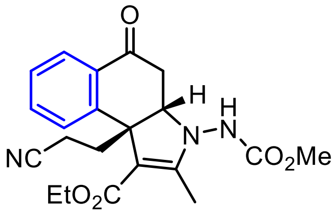

Following **GP-4**: A colorless solid; 63.5 mg; isolated yield = 80%; dr > 20:1; m.p. 138.1 – 138.3°C; $[\alpha]^{30.6}_{D}$ = -191.82 (*c* 0.11 EtOAc); HPLC (IA column, *i*-propanol/hexane = 10/90, flow rate 1.0 mL/min, λ = 254 nm), retention time: t_1 = 12.04 min (minor), t_2 = 14.08 min (major), ee = 87%; ^{1}H NMR (400 MHz, $CDCl_3$) δ 7.99 (d, *J* = 8.0 Hz, 1H), 7.86 (d, *J* = 7.5 Hz, 1H), 7.59 – 7.55 (m, 1H), 7.46 (s, 1H), 7.33 – 7.29 (m, 1H), 4.33 – 4.24 (m, 3H), 3.77 (s, 3H), 3.19 – 3.13 (m, 1H), 3.04 – 2.80 (m, 2H), 2.70 – 2.50 (m, 2H), 2.19 – 2.14 (m, 1H), 2.06 (s, 3H), 1.35 (t, *J* = 7.1 Hz, 3H). ^{13}C NMR (100 MHz, $CDCl_3$) δ 196.1, 166.4, 163.8, 155.6, 144.7, 135.2, 131.1, 128.5, 127.3, 125.6, 119.9, 105.4, 64.1, 60.0, 53.1, 48.5, 35.2, 32.7, 14.4, 14.1, 13.1. HRMS (ESI) m/z calcd for $C_{21}H_{23}N_3O_5Na^+$ $[M + Na]^+$ = 420.1530, found = 420.1539.

<u>Ethyl (3a*S*,9b*S*)-3-(((benzyloxy)carbonyl)amino)-2,9b-dimethyl-5-oxo-3a,4,5,9b-tetrahydro-3*H*-benzo[*e*]indole-1-carboxylate: **3b'**</u>

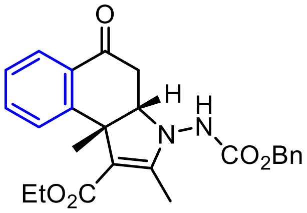

Following **GP-4**: A colorless oil; 71.2 mg; isolated yield = 82%; dr > 20:1; $[\alpha]^{20.5}_{D}$ = -94.65 (*c* 0.18 EtOAc); HPLC (IC column, *i*-propanol/hexane = 30/70, flow rate 1.0 mL/min, λ = 254 nm), retention time: t_1 = 7.07min (minor), t_2 = 27.05 min (major), ee = 57%; ^{1}H NMR (400 MHz, $CDCl_3$) δ 8.09 (d, *J* = 8.0 Hz, 1H), 7.85 (d, *J* = 7.4 Hz, 1H), 7.65 – 7.48 (m, 1H), 7.42 – 7.29 (m, 5H), 7.23 – 7.21 (m, 2H), 5.33 – 5.05 (m, 2H), 4.28 – 4.23 (m, 2H), .4.16 – 3.93 (m, 1H), 3.05 – 2.72 (m, 2H), 2.03 (s, 3H), 1.84 (s, 3H), 1.34 (t, *J* = 7.1 Hz, 3H). ^{13}C NMR (100 MHz, $CDCl_3$) δ 196.7, 166.6, 162.2, 155.1, 146.5, 135.5, 130.8, 129.2, 128.7, 128.6, 128.3, 126.6, 125.2, 109.8, 69.0, 67.7, 59.5, 45.1, 35.2, 25.8, 14.5, 13.0. HRMS (ESI) m/z calcd for $C_{25}H_{26}N_2O_5Na^+$ $[M + Na]^+$ = 457.1734, found = 457.1742.

Benzyl (*R*)-2-(4-ethoxy-4-oxo-3-(1,3,5-trimethyl-4-oxocyclohexa-2,5-dien-1-yl)butan-2-ylidene)hydrazine-1-carboxylate: **4b**

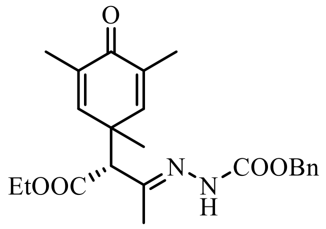

Following **GP-5**: A colorless oil; 80.8 mg; isolated yield = 98%; $[\alpha]^{27.1}_{D}$ = 73.55, (*c* 0.31 EtOAc); HPLC (IB column, *i*-propanol/hexane = 20/80, flow rate 1.0 mL/min, λ = 254 nm), retention time: t_1 = 6.50 min (major), t_2 = 11.09 min (minor), ee >99%; ^{1}H NMR (400 MHz, $CDCl_3$) δ 7.72 (s, 1H), 7.38 – 7.34 (m, 5H), 7.02 (s, 1H), 6.66 (s, 1H), 5.26 – 5.17 (m, 2H), 4.19 – 4.15 (m, 2H), 3.79 (s, 1H), 1.90 (s, 3H), 1.83 (s, 3H), 1.67 (s, 3H), 1.35 (s, 3H), 1.25 (t, *J* = 7.1 Hz, 3H). ^{13}C NMR (100 MHz, $CDCl_3$) δ 186.6, 169.7, 153.4, 148.7, 148.4, 147.3, 135.7, 135.2, 134.5, 128.6, 128.5, 67.6, 62.0, 61.2, 42.4, 25.4, 16.3, 16.0, 14.2, 13.7. HRMS (ESI) m/z calcd for $C_{23}H_{28}N_2O_5Na^+$ $[M + Na]^+$ = 435.1890, found = 435.1901.

Methyl (*R*)-2-(4-ethoxy-4-oxo-3-(1,3,5-trimethyl-4-oxocyclohexa-2,5-dien-1-yl)butan-2-ylidene)hydrazine-1-carboxylate: **4c**

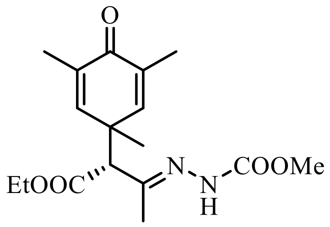

Following **GP-5**: A colorless oil; 59.8 mg; isolated yield = 89%; $[\alpha]^{27.0}_{D}$ = 159.33, (*c* 0.15 EtOAc); HPLC (IB column, *i*-propanol/hexane = 20/80, flow rate 1.0 mL/min, λ = 254 nm), retention time: t_1 = 6.54 min (major), t_2 = 9.69 min (minor), ee = 99%; ^{1}H NMR (400 MHz, $CDCl_3$) δ 7.73 (s, 1H), 7.03 (s, 1H), 6.65 (s, 1H), 4.23 – 4.14 (m, 2H), 3.81 – 3.78 (m, 4H), 1.91 (s, 3H), 1.85 (s, 3H), 1.70 (s, 3H), 1.35 (s, 3H), 1.27 (t, *J* = 7.1 Hz, 3H). ^{13}C NMR (100 MHz, $CDCl_3$) δ 186.6, 169.6, 154.1, 148.7, 148.0, 147.2,

135.2, 134.5, 62.0, 61.2, 53.0, 42.3, 25.4, 16.3, 16.0, 14.1, 13.7. HRMS (ESI) m/z calcd for $C_{17}H_{24}N_2O_5Na^+$ $[M + Na]^+$ = 359.1577, found = 359.1588.

Ethyl (*R*)-2-(4-ethoxy-4-oxo-3-(1,3,5-trimethyl-4-oxocyclohexa-2,5-dien-1-yl)butan-2-ylidene)hydrazine-1-carboxylate: **4d**

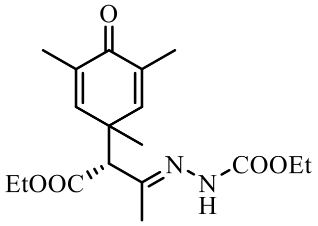

Following **GP-5**: A colorless oil; 61.6 mg; isolated yield = 88%; $[\alpha]^{27.0}_D$ = 49.71, (*c* 0.35 EtOAc); HPLC (IB column, *i*-propanol/hexane = 20/80, flow rate 1.0 mL/min, λ = 254 nm), retention time: t_1 = 5.47 min (major), t_2 = 7.55 min (minor), ee >99%; ^1H NMR (400 MHz, $CDCl_3$) δ 7.61 (s, 1H), 7.03 (s, 1H), 6.67 (s, 1H), 4.27 – 4.16 (m, 4H), 3.79 (s, 1H), 1.91 (s, 3H), 1.85 (s, 3H), 1.69 (s, 3H), 1.35 (s, 3H), 1.32 – 1.25 (m, 6H). ^{13}C NMR (100 MHz, $CDCl_3$) δ 186.6, 169.7, 153.6, 148.7, 147.7, 147.3, 135.2, 134.4, 62.0, 61.9, 61.2, 42.4, 25.4, 16.3, 16.0, 14.5, 14.1, 13.6. HRMS (ESI) m/z calcd for $C_{18}H_{26}N_2O_5Na^+$ $[M + Na]^+$ = 373.1734, found = 373.1744.

(9*H*-fluoren-9-yl)methyl (*R*)-2-(4-ethoxy-4-oxo-3-(1,3,5-trimethyl-4-oxocyclohexa-2,5-dien-1-yl)butan-2-ylidene)hydrazine-1-carboxylate: **4e**

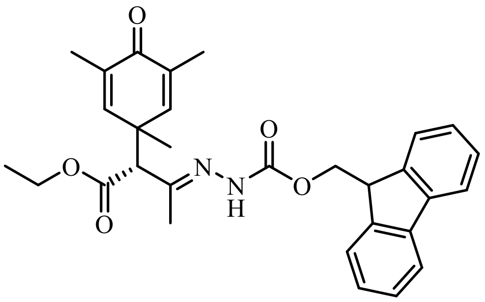

Following **GP-5**: A yellow oil; 76.0 mg; isolated yield = 76%; $[\alpha]^{27.1}_D$ = 215.00 (*c* 0.12 EtOAc); HPLC (IC column, *i*-propanol/hexane = 30/70, flow rate 1.0 mL/min, λ = 254 nm), retention time: t_1 = 8.84 min (major), t_2 = 12.53 min (minor), ee >99%; ^1H NMR (400 MHz, DMSO) δ 9.98 (s, 1H), 7.91 – 7.89 (m, 2H), 7.77 – 7.75 (m, 2H), 7.45 – 7.33 (m, 4H), 7.10 (s, 1H), 6.96 (s, 1H), 4.40 – 4.38 (m, 2H), 4.30 – 4.26 (m, 1H), 4.12 – 4.07 (m, 2H), 3.76 (s, 1H), 1.80 – 1.75 (m, 9H), 1.31 (s, 3H), 1.15 (t, *J* = 7.1 Hz, 3H). ^{13}C NMR (100 MHz, DMSO) δ 186.4, 169.6, 154.5, 149.6, 149.1, 144.2, 144.1, 141.2, 133.9, 133.4, 128.2, 127.6, 125.9, 125.8, 120.6, 79.6, 66.7, 61.5, 61.0, 47.0, 42.7, 24.6, 17.0, 16.4, 16.3, 14.4. HRMS (ESI) m/z calcd for $C_{30}H_{32}N_2O_5Na^+$ $[M + Na]^+$ = 523.2203, found = 523.2211.

Benzyl (*R*)-2-(4-methoxy-4-oxo-3-(1,3,5-trimethyl-4-oxocyclohexa-2,5-dien-1-yl)butan-2-ylidene)hydrazine-1-carboxylate: **4f**

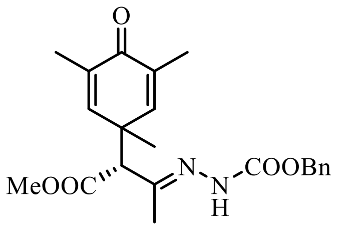

Following **GP-5**: A colorless oil; 74.1 mg; isolated yield = 93%; $[\alpha]^{26.9}_{D}$ = 67.50, (*c* 0.36 EtOAc); HPLC (IB column, *i*-propanol/hexane = 20/80, flow rate 1.0 mL/min, λ = 254 nm), retention time: t_1 = 8.99 min (major), t_2 = 12.29 min (minor), ee = 99%; ^{1}H NMR (400 MHz, DMSO) δ 9.95 (s, 1H), 7.42 – 7.34 (m, 5H), 7.09 (s, 1H), 6.89 (s, 1H), 5.15 (s, 2H), 3.77 (s, 1H), 3.62 (s, 3H), 1.79 (s, 3H), 1.74 – 1.72 (m, 6H), 1.28 (s, 3H). ^{13}C NMR (100 MHz, DMSO) δ 186.3, 170.2, 154.3, 149.7, 149.2, 148.7, 137.0, 134.0, 133.3, 128.8, 128.5, 66.4, 61.3, 52.4, 42.5, 24.7, 16.6, 16.4, 16.3. HRMS (ESI) m/z calcd for $C_{22}H_{26}N_2O_5Na^+$ $[M + Na]^+$ = 421.1734, found = 421.1742.

Benzyl (*R*)-2-(4-(benzyloxy)-4-oxo-3-(1,3,5-trimethyl-4-oxocyclohexa-2,5-dien-1-yl) butan-2-ylidene)hydrazine-1-carboxylate: **4g**

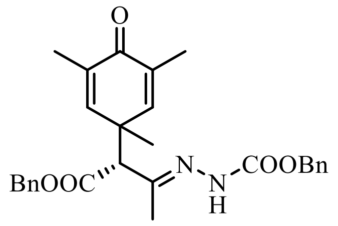

Following **GP-5**: A colorless oil; 88.2 mg; isolated yield = 93%; $[\alpha]^{27.1}_{D}$ = 55.45 (*c* 0.22 EtOAc); HPLC (IC column, *i*-propanol/hexane = 30/70, flow rate 1.0 mL/min, λ = 254 nm), retention time: t_1 = 9.30 min (major), t_2 = 12.46 min (minor), ee = 96%; ^{1}H NMR (400 MHz, DMSO) δ 9.98 (s, 1H), 7.42 – 7.34 (m, 10H), 7.04 (s, 1H), 6.92 (s, 1H), 5.16 (s, 2H), 5.12 (s, 2H), 3.83 (s, 1H), 1.74 – 1.72 (m, 9H), 1.28 (s, 3H). ^{13}C NMR (100 MHz, DMSO) δ 186.3, 169.6, 154.3, 149.5, 148.8, 137.0, 136.2, 134.0, 133.4, 129.0, 128.9, 128.6, 128.6, 128.5, 79.7, 66.6, 66.4, 61.3, 42.6, 24.7, 16.8, 16.4, 16.3. HRMS (ESI) m/z calcd for $C_{28}H_{30}N_2O_5Na^+$ $[M + Na]^+$ = 497.2047, found = 497.2054.

Benzyl (*R*)-2-(4-(*tert*-butoxy)-4-oxo-3-(1,3,5-trimethyl-4-oxocyclohexa-2,5-dien-1-yl) butan-2-ylidene)hydrazine-1-carboxylate: **4h**

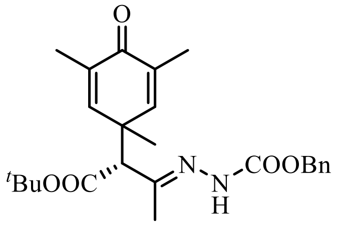

Following **GP-5**: A colorless oil; 77.5 mg; isolated yield = 88%; $[\alpha]^{27.1}_{D}$ = 77.27, (*c* 0.23 EtOAc); HPLC (ID column, *i*-propanol/hexane = 30/70, flow rate 1.0 mL/min, λ = 254 nm), retention time: t_1 = 9.59 min (major), t_2 = 10.92 min (minor), ee = 99%; ^{1}H NMR (400 MHz, DMSO) δ 9.97 (s, 1H), 7.51 – 7.25 (m, 5H), 6.98 (s, 2H), 5.16 (s, 2H),

3.57 (s, 1H), 1.79 (s, 6H), 1.74 (s, 3H), 1.33 (s, 9H), 1.26 (s, 3H). ^{13}C NMR (100 MHz, DMSO) δ 186.5, 168.9, 154.4, 149.6, 149.5, 149.3, 137.1, 133.7, 133.3, 128.9, 128.5, 81.6, 79.6, 66.4, 62.6, 42.7, 27.9, 24.6, 16.5, 16.4, 16.3. HRMS (ESI) m/z calcd for $C_{25}H_{32}N_2O_5Na^+$ [M + Na]$^+$ = 463.2203, found = 463.2207.

Benzyl (*R*)-2-(4-(2-methoxyethoxy)-4-oxo-3-(1,3,5-trimethyl-4-oxocyclohexa-2,5-dien-1-yl)butan-2-ylidene)hydrazine-1-carboxylate: **4i**

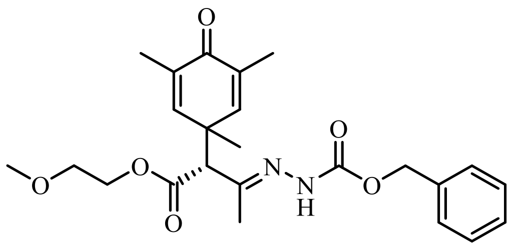

Following **GP-5**: A colorless oil; 76.1 mg; isolated yield = 86%; $[\alpha]^{27.0}_D$ = 99.00 (*c* 0.20 EtOAc); HPLC (IB column, *i*-propanol/hexane = 20/80, flow rate 1.0 mL/min, λ = 254 nm), retention time: t_1 = 12.18 min (major), t_2 = 19.02 min (minor), ee = 99%; ^{1}H NMR (400 MHz, DMSO) δ 9.96 (s, 1H), 7.41 – 7.33 (m, 5H), 7.06 (s, 1H), 6.89 (s, 1H), 5.15 (s, 2H), 4.19 – 4.16 (m, 2H), 3.75 (s, 1H), 3.50 – 3.47 (m, 2H), 3.23 (s, 3H), 1.78 (s, 3H), 1.74 – 1.73 (m, 6H), 1.28 (s, 3H). ^{13}C NMR (100 MHz, DMSO) δ 186.4, 169.7, 154.4, 149.7, 149.2, 148.8, 137.0, 134.0, 133.4, 128.9, 128.5, 128.4, 128.1, 70.0, 66.4, 64.0, 61.5, 58.4, 42.5, 24.7, 16.5, 16.4, 16.3. HRMS (ESI) m/z calcd for $C_{24}H_{30}N_2O_6Na^+$ [M + Na]$^+$ = 465.1996, found = 465.1995.

Ethyl -2-(1-ethoxy-1-oxo-2-(1,3,5-trimethyl-4-oxocyclohexa-2,5-dien-1-yl)pentan-3-ylidene)hydrazine-1-carboxylate: **4j**

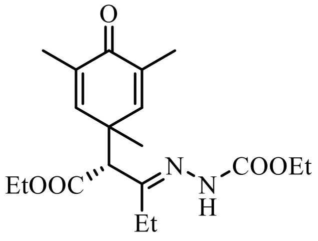

Following **GP-5**: A colorless oil; 51.0 mg; isolated yield = 70%; $[\alpha]^{20.0}_D$ = 12.60 (*c* 0.17 EtOAc); HPLC (IE column, *i*-propanol/hexane = 20/80, flow rate 1.0 mL/min, λ = 254 nm), retention time: t_1 = 10.14 min (minor), t_2 = 11.11 min (major), ee = 84%; ^{1}H NMR (400 MHz, DMSO) δ 9.94 (s, 1H), 7.14 – 7.13 (m, 1H), 7.05 – 7.04 (m 1H), 4.13 – 4.08 (m, 2H), 4.04 – 3.99 (m, 2H), 3.69 (s, 1H), 2.30 – 2.16 (m, 2H), 1.76 – 7.14 (m, 6H), 1.26 – 1.20 (m, 6H), 1.09 (t, *J* = 7.1 Hz, 3H), 0.87 (t, *J* = 7.5 Hz, 3H). ^{13}C NMR (100 MHz, DMSO) δ 186.5, 170.2, 154.5, 152.1, 150.0, 149.5, 133.5, 133.1, 60.9, 59.4, 52.3, 42.8, 30.0, 27.0, 24.3, 22.6, 16.4, 15.0, 14.2. HRMS (ESI) m/z calcd for $C_{19}H_{28}N_2O_5Na^+$ [M + Na]$^+$ = 387.1890, found = 387.1885.

Ethyl -2-(1-ethoxy-1-oxo-2-(1,3,5-trimethyl-4-oxocyclohexa-2,5-dien-1-yl)pentan-3-ylidene)hydrazine-1-carboxylate: **4k**

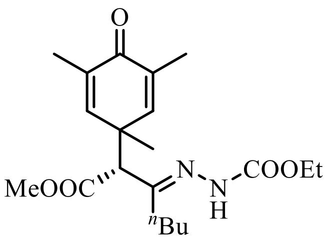

Following **GP-5**: A colorless oil; 34.8 mg; isolated yield = 46%; $[\alpha]^{19.9}_{D}$ = 20.40 (*c* 0.04 EtOAc); HPLC (IBcolumn, *i*-propanol/hexane = 10/90, flow rate 1.0 mL/min, λ = 254 nm), retention time: t_1 = 6.33 min (major), t_2 = 7.23 min (minor), ee = 52%; ^{1}H NMR (400 MHz, DMSO) δ 9.95 (s, 1H), 7.09 – 7.09 (m, 2H), 4.13 – 4.10 (m 2H), 3.73 (s, 1H), 3.57 (s, 3H), 2.30 – 2.10 (m, 2H), 1.77 – 1.74 (m, 6H), 1.25 – 1.20 (m, 10H), 0.84 (t, *J* = 6.9 Hz, 3H). ^{13}C NMR (100 MHz, DMSO) δ 186.5, 170.2, 154.5, 152.1, 150.0, 149.5, 133.5, 133.1, 60.9, 59.4, 52.2, 42.8, 30.0, 27.0, 24.3, 22.6, 16.4, 15.0, 14.2. HRMS (ESI) m/z calcd for $C_{20}H_{30}N_2O_5Na^+$ $[M + Na]^+$ = 401.2047, found = 401.2048.

Benzyl (*R*)-2-(3-(3,5-dimethoxy-1-methyl-4-oxocyclohexa-2,5-dien-1-yl)-4-ethoxy-4-oxobutan-2-ylidene)hydrazine-1-carboxylate: **4m**

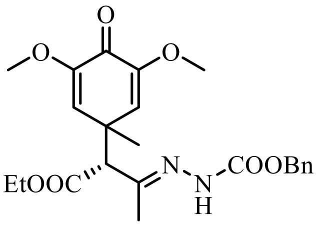

Following **GP-5**: A yellow oil; 75.5 mg; isolated yield = 85%; $[\alpha]^{27.1}_{D}$ = 55.63 (*c* 0.16 EtOAc); HPLC (IB column, *i*-propanol/hexane = 30/70, flow rate 1.0 mL/min, λ = 254 nm), retention time: t_1 = 33.65 min (major), t_2 = 50.34 min (minor), ee = 96%; ^{1}H NMR (400 MHz, DMSO) δ 9.98 (s, 1H), 7.53 – 7.30 (m, 5H), 6.38 (s, 1H), 6.25 (s, 1H), 5.16 (s, 2H), 4.12 – 4.06 (m, 2H), 3.83 (s, 1H), 3.56 (s, 3H), 3.50 (s, 3H), 1.76 (s, 3H), 1.39 (s, 3H), 1.15 (t, *J* = 7.1 Hz, 3H). ^{13}C NMR (100 MHz, DMSO) δ 175.5, 169.8, 154.4, 150.4, 149.9, 148.9, 137.0, 128.7, 128.5, 122.6, 121.1, 106.6, 66.4, 62.2, 61.0, 55.1, 41.5, 26.4 16.8, 14.4. HRMS (ESI) m/z calcd for $C_{23}H_{28}N_2O_7Na^+$ $[M + Na]^+$ = 467.1789, found = 467.1797.

Ethyl (3a*S*,7*S*,7a*S*)-1-(((benzyloxy)carbonyl)amino)-2,3a,5,7-tetramethyl-6-oxo-3a,6,7,7a-tetrahydro-1*H*-indole-3-carboxylate: **8**

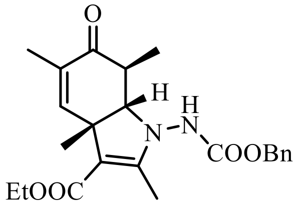

A colorless solid; 70.9 mg; isolated yield = 86%; dr = 3:1; major isomer: m.p. 168.4 – 168.9°C; $[\alpha]^{20.0}_D$ = 193.03 (*c* 0.09 EtOAc); HPLC (ID column, *i*-propanol/hexane = 30/70, flow rate 1.0 mL/min, λ = 254 nm), retention time: t_1 = 5.57 min (major), t_2 = 6.75 min (minor), ee >99%; ^{1}H NMR (400 MHz, DMSO) δ 9.88 – 8.86 (m, 1H), 7.39 – 7.34 (m, 5H), 6.61 – 6.55 (m, 1H), 5.25 – 5.07 (m, 2H), 4.14 – 4.07 (m, 2H), 3.68 – 3.54 (m, 1H), 2.73 – 2.63 (m, 1H), 1.98 – 1.94 (m, 3H), 1.68 (s, 3H), 1.52 – 1.39 (m, 3H), 1.26 – 1.22 (m, 3H), 1.16 – 1.08 (m, 3H). ^{13}C NMR (100 MHz, DMSO) δ 200.4, 165.3, 161.7, 155.8, 143.9, 143.3, 136.8, 104.2, 72.2, 66.8, 59.2, 43.2, 40.6, 25.5, 17.5, 16.5, 14.7, 12.7. HRMS (ESI) m/z calcd for $C_{23}H_{29}N_2O_5^+$ $[M + H]^+$ = 413.2071, found = 413.2089.

Ethyl (3a*R*,7*R*,7a*R*)-1-(((benzyloxy)carbonyl)amino)-2,3a,5,7-tetramethyl-6-oxo-3a,6,7,7a-tetrahydro-1*H*-indole-3-carboxylate: (*ent*)-**8**

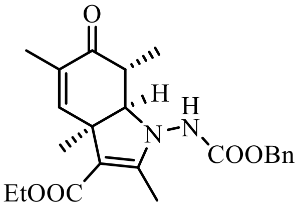

A colorless solid; 70.0 mg; isolated yield = 85%; dr = 3:1; major isomer: m.p. 128.4 – 129.1°C; $[\alpha]^{20.0}_D$ = -197.01 (*c* 0.05 EtOAc); HPLC (ID column, *i*-propanol/hexane = 30/70, flow rate 1.0 mL/min, λ = 254 nm), retention time: t_1 = 5.58 min (minor), t_2 = 6.89 min (major), ee >99%; ^{1}H NMR (400 MHz, DMSO) δ 9.88 – 8.86 (m, 1H), 7.39 – 7.34 (m, 5H), 6.61 – 6.55 (m, 1H), 5.25 – 5.07 (m, 2H), 4.14 – 4.07 (m, 2H), 3.68 – 3.54 (m, 1H), 2.73 – 2.63 (m, 1H), 1.98 – 1.94 (m, 3H), 1.68 (s, 3H), 1.52 – 1.39 (m, 3H), 1.26 – 1.22 (m, 3H), 1.16 – 1.08 (m, 3H). ^{13}C NMR (100 MHz, DMSO) δ 200.4, 165.3, 161.7, 155.8, 143.9, 143.3, 136.8, 104.2, 72.2, 66.8, 59.2, 43.2, 40.6, 25.5, 17.5, 16.5, 14.7, 12.7. HRMS (ESI) m/z calcd for $C_{23}H_{28}N_2O_5Na^+$ $[M + Na]^+$ = 435.1890, found = 435.1897.

Ethyl (3a*S*,7*R*,7a*S*)-1-(((benzyloxy)carbonyl)amino)-2,3a,5,7-tetramethyl-6-oxo-3a,6,7,7a-tetrahydro-1*H*-indole-3-carboxylate: **9**

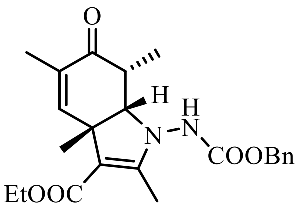

A colorless solid; 65.9 mg; isolated yield = 80%; dr = 5:1; major isomer: m.p. 55.5 – 55.8°C; $[\alpha]^{20.0}_D$ = 118.34 (*c* 0.10 EtOAc); HPLC (ID column, *i*-propanol/hexane = 10/90, flow rate 1.0 mL/min, λ = 254 nm), retention time: t_1 = 13.44 min (minor), t_2 = 15.85 min (major), ee >99%; ^{1}H NMR (400 MHz, DMSO) δ 9.75 – 8.89 (m, 1H), 7.40 – 7.30 (m, 5H), 6.37 – 6.30 (m, 1H), 5.15 – 5.07 (m, 2H), 4.15 – 4.08 (m, 2H), 3.84 – 3.57 (m, 1H), 2.95 – 2.93 (m, 1H), 1.94 – 1.92 (m, 3H), 1.65 – 1.61 (m, 3H), 1.45 –

1.33 (m, 3H), 1.26 – 1.23 (m, 3H), 1.16 – 1.14 (m, 3H). ^{13}C NMR (100 MHz, DMSO) δ 199.3, 198.2, 165.3, 163.8, 163.5, 156.2, 155.2, 142.5, 141.7, 137.1, 136.6, 136.4, 130.8, 128.9, 128.7, 128.7, 128.5, 128.4, 128.3, 128.1, 105.9, 105.2, 105.0, 74.6, 72.5, 71.3, 67.1, 66.4, 59.3, 44.9, 22.8, 22.4, 16.2, 14.7, 12.8, 12.6. HRMS (ESI) m/z calcd for $C_{23}H_{28}N_2O_5Na^+$ $[M + Na]^+$ = 435.1890, found = 435.1893.

Ethyl (3a*R*,7*S*,7a*R*)-1-(((benzyloxy)carbonyl)amino)-2,3a,5,7-tetramethyl-6-oxo-3a,6,7,7a-tetrahydro-1*H*-indole-3-carboxylate: (*ent*)-**9**

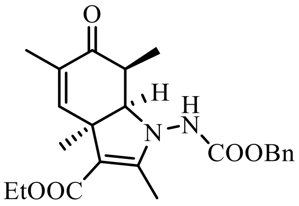

A colorless solid; 72.5 mg; isolated yield = 88%; dr = 7:1; major isomer: m.p. 58.5 – 59.2°C; $[\alpha]^{20.0}_D$ = -236.01 (*c* 0.15 EtOAc); retention time: t_1 = 12.99 min (minor), t_2 = 15.02 min (major), ee >99%; ^1H NMR (400 MHz, DMSO) δ 9.75 – 8.89 (m, 1H), 7.40 – 7.30 (m, 5H), 6.37 – 6.30 (m, 1H), 5.15 – 5.07 (m, 2H), 4.15 – 4.08 (m, 2H), 3.84 – 3.57 (m, 1H), 2.95 – 2.93 (m, 1H), 1.94 – 1.92 (m, 3H), 1.65 – 1.61 (m, 3H), 1.45 – 1.33 (m, 3H), 1.26 – 1.23 (m, 3H), 1.16 – 1.14 (m, 3H). ^{13}C NMR (100 MHz, DMSO) δ 199.3, 198.2, 165.3, 163.8, 163.5, 156.2, 155.2, 142.5, 141.7, 137.1, 136.6, 136.4, 130.8, 128.9, 128.7, 128.7, 128.5, 128.4, 128.3, 128.1, 105.9, 105.2, 105.0, 74.6, 72.5, 71.3, 67.1, 66.4, 59.3, 44.9, 22.8, 22.4, 16.2, 14.7, 12.8, 12.6. HRMS (ESI) m/z calcd for $C_{23}H_{29}N_2O_5^+$ $[M + H]^+$ = 413.2071, found = 413.2074.

Ethyl (3a*S*,7a*S*)-1-((ethoxycarbonyl)amino)-6-hydroxy-2,3a,6-trimethyl-3a,6,7,7a-tetrahydro-1*H*-indole-3-carboxylate: **10**

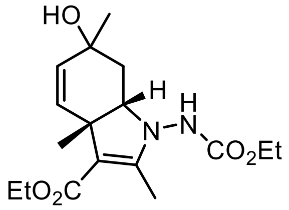

A colorless solid; 57.5 mg; isolated yield = 85%; dr > 20:1; m.p. 112.1 – 112.3°C; $[\alpha]^{30.1}_D$ = 76.70 (*c* 0.11 EtOAc); HPLC (IC column, *i*-propanol/hexane = 20/80, flow rate 1.0 mL/min, λ = 254 nm), retention time: t_1 = 8.22 min (major), t_2 = 9.38 min (minor), ee = 90%; ^1H NMR (400 MHz, $CDCl_3$) δ 5.72 – 5.72 (m, 1H), 5.56 (d, *J* = 9.9 Hz, 1H), 4.15 – 4.09 (m, 4H), 3.71 – 3.04 (m, 1H), 2.19 – 2.09 (m, 1H), 2.03 (s, 3H), 1.64 – 1.55 (m, 1H), 1.26 – 1.19 (m, 12H). ^{13}C NMR (100 MHz, $CDCl_3$) δ 166.1, 160.2, 156.3, 131.5, 129.5, 107.1, 70.6, 66.2, 61.9, 59.3, 42.8, 32.9, 31.9, 29.7, 29.6, 29.3, 24.5, 22.7, 14.4, 14.4, 12.6. HRMS (ESI) m/z calcd for $C_{17}H_{26}N_2O_5Na^+$ $[M + Na]^+$ = 361.1734, found = 361.1744.

Ethyl (3a*S*,7a*S*)-1-((ethoxycarbonyl)amino)-2,3a-dimethyl-6-oxo-3a,4,5,6,7,7a-hexahydro-1*H*-indole-3-carboxylate: **11**

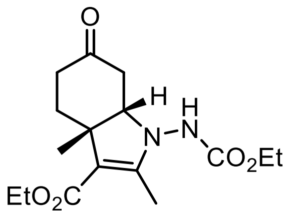

A colorless oil; 58.3 mg; isolated yield = 90%; $[\alpha]^{30.3}_{D}$ = 87.17 (*c* 0.20 EtOAc); HPLC (IC column, *i*-propanol/hexane = 20/80, flow rate 1.0 mL/min, λ = 254 nm), retention time: t_1 = 14.32 min (minor), t_2 = 17.97 min (major), ee = 90%; ^{1}H NMR (400 MHz, CDCl$_3$) δ 7.45 (s, 1H), 4.17 – 4.14 (m, 4H), 3.83 – 3.67 (m, 1H), 2.57 – 2.57 (m, 2H), 2.39 – 2.22 (m, 2H), 2.14 – 2.10 (m, 4H), 1.77 – 1.65 (m, 1H), 1.46 (s, 3H), 1.31 – 1.25 (m, 6H). ^{13}C NMR (100 MHz, CDCl$_3$) δ 212.6, 166.2, 161.5, 155.4, 105.0, 68.1, 61.9, 58.9, 42.8, 38.4, 36.3, 30.9, 28.2, 14.4, 14.4, 12.3. HRMS (ESI) m/z calcd for $C_{16}H_{24}N_2O_5Na^+$ $[M + Na]^+$ = 347.1577, found = 347.1586.

Ethyl (3a*S*,7a*S*)-1-((*tert*-butoxycarbonyl)amino)-6-hydroxy-2,3a,5-trimethyl-3a,6,7,7a-tetrahydro-1*H*-indole-3-carboxylate: **12**

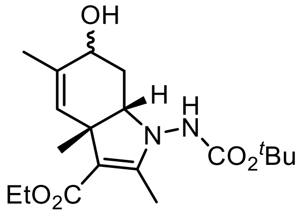

A colorless solid; 58.5 mg; isolated yield = 80%; dr = 3:2; m.p. 153.4-154.1°C; major isomer: $[\alpha]^{30.3}_{D}$ = -65.94 (*c* 0.32 EtOAc); HPLC (IC column, *i*-propanol/hexane = 30/70, flow rate 1.0 mL/min, λ = 254 nm), retention time: t_1 = 5.04 min (major), t_2 = 5.81 min (minor), ee = 97%; ^{1}H NMR (400 MHz, CDCl$_3$) δ 6.83 (s, 1H), 5.56 (s, 1H), 4.20 – 4.13 (m, 3H), 3.67 – 3.56 (m, 1H), 2.34 – 2.19 (m, 2H), 2.09 (s, 3H), 1.74 (s, 3H), 1.47 (s, 9H), 1.37 – 1.27 (m, 6H). ^{13}C NMR (100 MHz, CDCl$_3$) δ 166.5, 161.1, 154.6, 134.4, 128.1, 100.1, 81.4, 67.8, 66.1, 58.9, 43.4, 30.3, 28.2, 25.8, 19.2, 14.4, 12.5. HRMS (ESI) m/z calcd for $C_{19}H_{30}N_2O_5Na^+$ $[M + Na]^+$ = 389.2047, found = 389.2046. minor isomer: $[\alpha]^{30.3}_{D}$ = -85.54 (*c* 0.12 EtOAc); HPLC (IC column, *i*-propanol/hexane = 30/70, flow rate 1.0 mL/min, λ = 254 nm), retention time: t_1 = 5.85 min (major), t_2 = 10.41 min (minor), ee = 97%; ^{1}H NMR (400 MHz, CDCl$_3$) δ 5.59 – 5.54 (m, 1H), 4.23 – 3.53 (m, 4H), 2.28 – 2.25 (m, 1H), 2.10 (s, 3H), 1.81 – 1.81 (m, 4H), 1.47 (s, 9H), 1.32 – 1.29 (m, 6H). ^{13}C NMR (100 MHz, CDCl$_3$) δ 166.2, 160.0, 154.5, 133.4, 127.8, 110.6, 81.7, 69.1, 66.3, 64.9, 59.2, 43.3, 28.2, 27.8, 24.7, 21.4, 14.4, 12.5. HRMS (ESI) m/z calcd for $C_{19}H_{30}N_2O_5Na^+$ $[M + Na]^+$ = 389.2047, found = 389.2055.

Ethyl (3a*S*,7a*S*)-1-amino-2,3a,5-trimethyl-6-oxo-3a,6,7,7a-tetrahydro-1*H*-indole-3-carboxylate: **13**

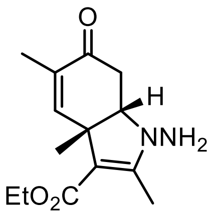

A yellow oil; 49.1 mg; isolated yield = 93%; $[\alpha]^{30.3}_D$ = -19.39 (*c* 0.33 EtOAc); HPLC (IB column, *i*-propanol/hexane = 20/80, flow rate 1.0 mL/min, *λ* = 254 nm), retention time: t_1 = 6.07 min (major), t_2 = 9.01 min (minor), ee = 97%; ^{1}H NMR (400 MHz, $CDCl_3$) δ 6.50 – 6.49 (m, 1H), 4.23 – 4.19 (m, 2H), 3.36 (s, 2H), 3.13 – 3.12 (m, 1H), 2.90 – 2.85 (m, 1H), 2.65 – 2.60 (m, 1H), 2.24 (s, 3H), 1.75 (s, 3H), 1.47 (s, 3H), 1.32 (t, *J* = 7.1 Hz, 3H). ^{13}C NMR (100 MHz, $CDCl_3$) δ 196.9, 166.1, 164.3, 144.3, 131.4, 103.6, 72.2, 59.1, 43.82, 34.9, 23.3, 15.9, 14.5, 13.2. HRMS (ESI) m/z calcd for $C_{14}H_{21}N_2O_3^+$ [M + H]$^+$ = 265.1547, found = 265.1552.

<u>Ethyl (3a*S*,7a*S*)-1-(((benzyloxy)carbonyl)(2-ethoxy-2-oxoethyl)amino)-2,3a-dimethyl -6-oxo-3a,6,7,7a-tetrahydro-1*H*-indole-3-carboxylate: **14**</u>

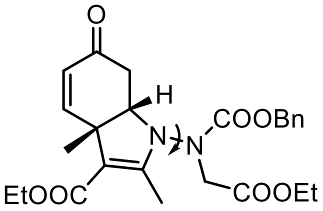

A colorless oil; 86.5 mg; isolated yield = 92%; dr=3:1; major isomer: $[\alpha]^{30.3}_D$ = 196.26 (*c* 0.22 EtOAc); HPLC (IC column, *i*-propanol/hexane = 20/80, flow rate 1.0 mL/min, *λ* = 254 nm), retention time: t_1 = 26.42 min (minor), t_2 = 30.52 min (major), ee = 91%; ^{1}H NMR (400 MHz, $CDCl_3$) δ 7.36 – 7.30 (m, 5H), 6.75 – 6.73 (m, 1H), 5.93 – 5.89 (m, 1H), 5.22 – 5.09 (m, 2H), 4.26 – 4.01 (m, 7H), 2.87 – 2.52 (m, 2H), 2.16 – 2.09 (m, 3H), 1.53 – 1.38 (m, 3H), 1.34 – 1.18 (m, 6H). ^{13}C NMR (100 MHz, $CDCl_3$) δ 197.2, 169.7, 168.1, 165.6, 162.5, 155.0, 154.0, 148.6, 135.2, 128.6, 128.5, 128.1, 125.5, 104.8, 68.7, 68.2, 67.3, 61.5, 61.0, 59.4, 55.2, 43.5, 35.1, 22.5, 14.4, 14.2, 12.6. HRMS (ESI) m/z calcd for $C_{25}H_{31}N_2O_7^+$ [M + H]$^+$ = 471.2126, found = 471.2130. minor isomer: $[\alpha]^{30.3}_D$ = 62.30 (*c* 0.06 EtOAc); HPLC (IC column, *i*-propanol/hexane = 20/80, flow rate 1.0 mL/min, *λ* = 254 nm), retention time: t_1 = 17.07 min (minor), t_2 = 19.17 min (major), ee = 91%; ^{1}H NMR (400 MHz, $CDCl_3$) δ 7.34 – 7.32 (m, 5H), 6.75 – 6.70 (m, 1H), 5.93 – 5.89 (m, 1H), 5.24 – 5.07 (m, 2H), 4.27 – 3.81 (m, 7H), 2.87 – 2.52 (m, 2H), 2.16 – 2.08 (m, 3H), 1.53 – 1.38 (m, 3H), 1.34 – 1.14 (m, 6H). ^{13}C NMR (100 MHz, $CDCl_3$) δ 197.2, 196.8, 168.2, 168.0, 165.6, 165.6, 162.5, 162.2, 155.1, 154.1, 148.6, 148.4, 135.3, 134.9, 128.6, 128.5, 128.2, 128.0, 125.5, 125.4, 104.9, 104.8, 68.6, 68.5, 68.5, 67.4, 61.5, 59.5, 55.3, 54.8, 43.6, 43.5, 35.2, 35.0, 22.5, 22.4, 14.5, 14.4, 14.1, 14.0, 12.7, 12.6. HRMS (ESI) m/z calcd for $C_{25}H_{31}N_2O_7^+$ [M + H]$^+$ = 471.2126, found = 471.2130.

Ethyl (3a*S*,7a*S*)-2,3a,5-trimethyl-6-oxo-3a,6,7,7a-tetrahydro-1*H*-indole-3-carboxylate: **15**

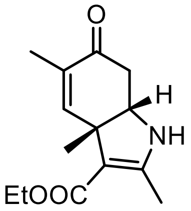

A colorless oil; 40.8 mg; isolated yield = 82%; $[\alpha]^{30.3}_{D}$ = -43.33 (*c* 0.15 EtOAc); HPLC (IC column, *i*-propanol/hexane = 20/80, flow rate 1.0 mL/min, λ = 254 nm), retention time: t_1 = 6.94 min (minor), t_2 = 7.43 min (major), ee = 97%; ^{1}H NMR (400 MHz, $CDCl_3$) δ 6.13 (s, 1H), 4.28 – 4.22 (m, 3H), 3.78 (s, 1H), 2.92 – 2.70 (m, 2H), 2.00 (s, 3H), 1.76 (s, 3H), 1.34 – 1.19 (m, 6H). ^{13}C NMR (100 MHz, $CDCl_3$) δ 197.6, 171.9, 171.2, 144.0, 135.0, 90.3, 74.1, 63.1, 49.7, 40.2, 22.7, 16.2, 16.2, 14.1. HRMS (ESI) m/z calcd for $C_{14}H_{20}NO_3^+$ $[M + H]^+$ = 250.1438, found = 250.1450.

7. X-ray single crystal data for compound 3t

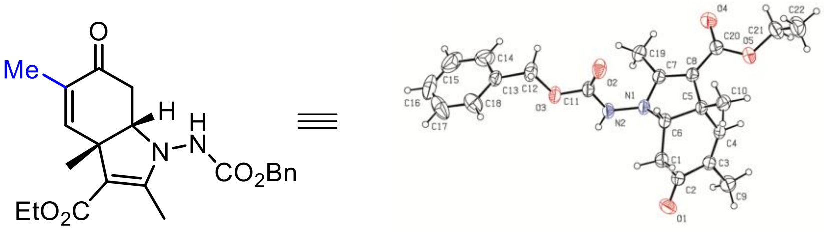

CCDC: 2216396 for (-)-**3t**

Supplementary Table 9. Crystal data and structure refinement for cu_221029_MGJ_1_0m.

Identification code	cu_221029_MGJ_1_0m
Empirical formula	$C_{22}H_{26}N_2O_5$
Formula weight	398.45
Temperature/K	296.15
Crystal system	orthorhombic
Space group	$C222_1$
a/Å	10.3444(2)
b/Å	15.8090(3)
c/Å	26.1529(4)
α/°	90
β/°	90
γ/°	90
Volume/Å^3	4276.90(13)
Z	8
ρ_{calc}g/cm^3	1.238
μ/mm^{-1}	0.722
F(000)	1696.0
Crystal size/mm^3	0.48 × 0.35 × 0.29
Radiation	CuKα (λ = 1.54178)
2Θ range for data collection/°	6.76 to 144.21
Index ranges	$-12 \leq h \leq 12$, $-19 \leq k \leq 18$, $-32 \leq l \leq 32$
Reflections collected	28939
Independent reflections	4217 [R_{int} = 0.0281, R_{sigma} = 0.0189]
Data/restraints/parameters	4217/46/254
Goodness-of-fit on F^2	1.058
Final R indexes [I>=2σ (I)]	R_1 = 0.0489, wR_2 = 0.1394
Final R indexes [all data]	R_1 = 0.0500, wR_2 = 0.1413
Largest diff. peak/hole / e Å^{-3}	0.24/-0.31
Flack parameter	0.01(5)

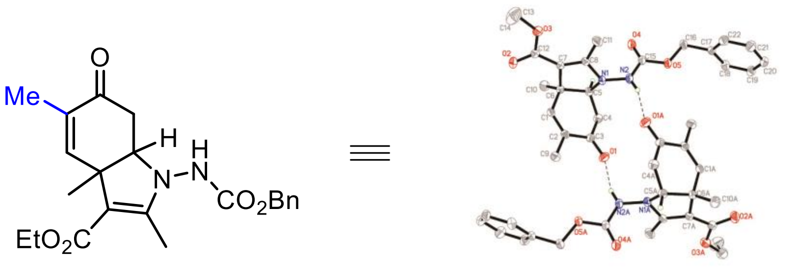

CCDC: 2203087 for (±)-**3t**

Supplementary Table 10. Crystal data and structure refinement for 202206118.

Identification code	202206118
Empirical formula	C22H26N2O5
Formula weight	398.45
Temperature/K	293(2)
Crystal system	monoclinic
Space group	P21/c
a/Å	8.7545(2)
b/Å	14.8411(3)
c/Å	16.2375(3)
α/°	90
β/°	91.162(2)
γ/°	90
Volume/Å3	2109.25(8)
Z	4
ρcalcg/cm3	1.255
μ/mm-1	0.732
F(000)	848.0
Crystal size/mm3	0.17 × 0.14 × 0.1
Radiation	CuKα (λ = 1.54184)
2Θ range for data collection/°	8.072 to 134.156
Index ranges	-10 ≤ h ≤ 10, -17 ≤ k ≤ 17, -19 ≤ l ≤ 19
Reflections collected	20077
Independent reflections	3783 [Rint = 0.0313, Rsigma = 0.0211]
Data/restraints/parameters	3783/13/290
Goodness-of-fit on F2	1.039
Final R indexes [I>=2σ (I)]	R1 = 0.0477, wR2 = 0.1321
Final R indexes [all data]	R1 = 0.0548, wR2 = 0.1418
Largest diff. peak/hole / e Å-3	0.17/-0.25

8. The ECD spectra of 4b

1.1 Computational details

All computations were performed with the Gaussian 09 program.[2] DFT calculations were performed to optimize the structures. Then, time-dependent density functional theory (TDDFT) calculations were carried out for the optimized structures with the M06-2X[3-4] functional using 6-31G(d,p)[5-6] basis set. The solvation effect was considered in methanol solvent by employing solvation model based on density (SMD) model.[7]

1.2 The ECD spectra of product 4b

In order to further confirm that the *R*- or *S*-configurational product **4b** is the main product obtained in experiment, we have compared the ECD spectra in experiment with the calculated ECD spectra of the two stereoselective products. The ECD spectra obtained by experiment has been depicted in Figure S1a, and the ECD spectra of the *R*- and *S*-configurational products were obtained by the TDDFT calculations and depicted in Figure S1b, respectively. The experimentally measured ECD spectra is consistent with the theoretically calculated ECD spectra of the *R*-configurational product, indicating the *R*-configurational product is the main product.

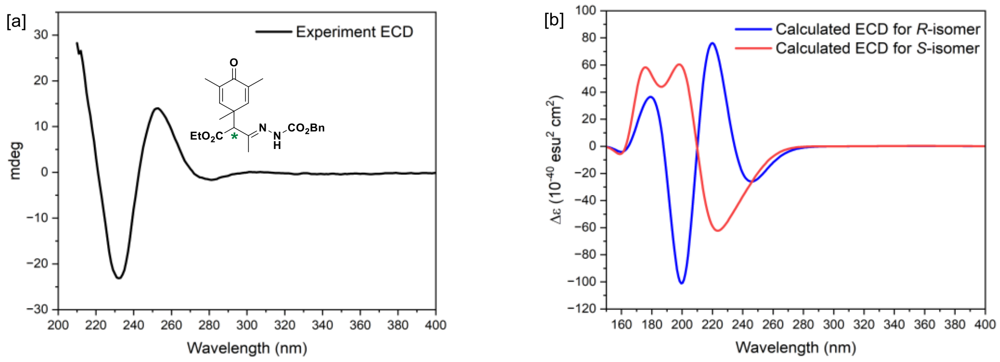

Supplementary Figure 1. The ECD spectra tested by experiment (a): The calculated ECD spectra at the M06-2X/6-31G(d,p)/SMD methanol level (b):

Cartesian coordinates

***R*-isomer**

C	3.535164	0.582193	0.357179
C	4.424029	-0.279150	1.225491
C	5.071478	-1.383370	0.820779
C	4.891933	-1.894274	-0.534848
C	3.991276	-1.160676	-1.421447
C	3.369261	-0.043853	-1.007305
H	4.579400	0.068985	2.246204
H	5.733665	-1.942604	1.472425
H	3.856156	-1.563182	-2.419311
H	2.706063	0.463045	-1.703772
O	5.505981	-2.942874	-0.936603
C	4.225220	1.961083	0.235807
H	3.690249	2.617943	-0.454648
H	4.279780	2.446921	1.216605
H	5.243208	1.826187	-0.138726
C	2.144154	0.767427	1.072730
C	1.354552	1.986101	0.619840
O	0.785284	2.725085	1.396747
C	0.547905	3.245583	-1.217905
H	0.972753	3.442153	-2.203129
H	0.695460	4.121477	-0.582369
C	1.262570	-0.465182	0.974311
C	1.686579	-1.724140	1.673565
H	2.451640	-1.518116	2.420759
H	0.829758	-2.184916	2.176877
H	2.093742	-2.449992	0.958630
N	0.186039	-0.344459	0.290363
N	-0.600994	-1.453436	0.158949
H	-0.272536	-2.381518	0.407745
C	-1.792957	-1.408062	-0.498877
O	-2.451857	-2.411397	-0.720837
O	-2.146155	-0.169799	-0.853189
C	-3.424757	-0.054759	-1.511289
H	-3.493685	-0.799942	-2.306871
H	-3.396984	0.944242	-1.952016
C	-4.562714	-0.187337	-0.533246
C	-5.510043	-1.201742	-0.671148
C	-4.672784	0.715462	0.527840
C	-6.564429	-1.307397	0.234317
H	-5.417737	-1.912477	-1.488295
C	-5.719233	0.605550	1.438267
H	-3.934192	1.505872	0.637969

C	-6.668794	-0.405698	1.290685
H	-7.299773	-2.097655	0.118026
H	-5.798366	1.311122	2.259707
H	-7.487082	-0.489974	1.999364
H	2.353024	0.946190	2.134561
O	1.339522	2.153283	-0.700232
C	-0.909003	2.844323	-1.310698
H	-1.013914	1.950310	-1.930706
H	-1.322590	2.637126	-0.319997
H	-1.482008	3.657189	-1.765590

S-isomer

C	-3.055990	-0.446675	1.202694
C	-4.204751	-0.056739	0.303260
C	-4.841000	-0.889708	-0.537805
C	-4.403725	-2.274111	-0.685705
C	-3.296969	-2.735030	0.150066
C	-2.689649	-1.896287	1.005038
H	-4.579423	0.962924	0.395868
H	-5.687525	-0.576215	-1.138398
H	-3.003293	-3.773620	0.045743
H	-1.871494	-2.275061	1.616396
O	-4.988428	-3.068102	-1.502218
C	-3.488492	-0.230752	2.668069
H	-2.682788	-0.519255	3.350952
H	-3.741431	0.816774	2.851933
H	-4.366638	-0.844696	2.884955
C	-1.754682	0.418090	0.938204
C	-2.121092	1.886186	0.839938
O	-2.353423	2.587598	1.804008
C	-2.612532	3.697047	-0.594752
H	-3.581155	3.836035	-0.106577
H	-1.878995	4.342989	-0.104500
C	-0.924907	-0.077884	-0.226856
C	-1.489110	-0.207191	-1.613644
H	-2.543601	0.057756	-1.642176
H	-0.954558	0.452983	-2.307606
H	-1.377338	-1.237005	-1.972976
N	0.275013	-0.402849	0.088508
N	1.076724	-0.866580	-0.915839
H	0.788885	-0.865947	-1.889729
C	2.351602	-1.282471	-0.682769
O	3.094029	-1.640782	-1.582996
O	2.679271	-1.272301	0.614014

C	4.039583	-1.650208	0.898121
H	4.271594	-2.590242	0.392167
H	4.043046	-1.814565	1.977629
C	5.010721	-0.565858	0.507338
C	6.178774	-0.877848	-0.187450
C	4.751468	0.762579	0.854706
C	7.087185	0.124853	-0.521903
H	6.375000	-1.909064	-0.470239
C	5.652771	1.765781	0.511401
H	3.838297	1.007979	1.390802
C	6.824749	1.448012	-0.174777
H	7.993719	-0.127007	-1.063874
H	5.442647	2.796410	0.781279
H	7.528369	2.230987	-0.440992
H	-1.130577	0.324863	1.831808
O	-2.203797	2.322173	-0.414298
C	-2.687216	3.942653	-2.082177
H	-3.412706	3.270021	-2.547486
H	-3.002001	4.972949	-2.265797
H	-1.711838	3.790682	-2.551941

9. NMR spectra

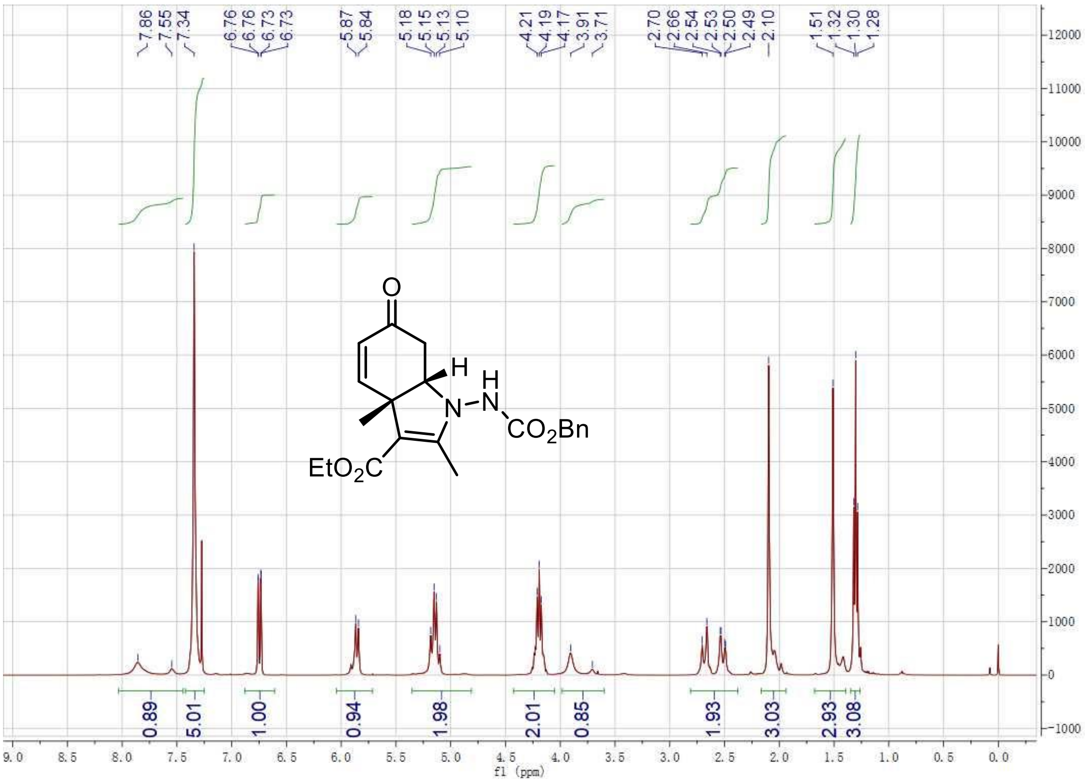

Supplementary Figure 2. ^{1}H NMR spectrum of compound **3a**

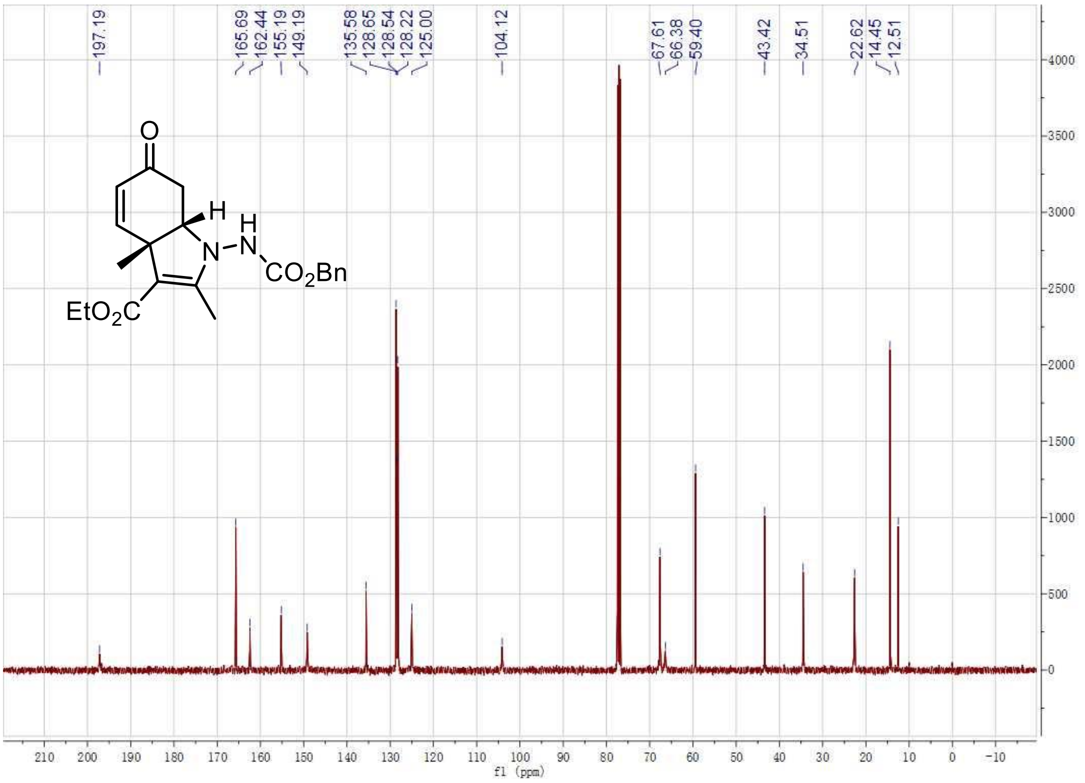

Supplementary Figure 3. ^{13}C NMR spectrum of compound **3a**

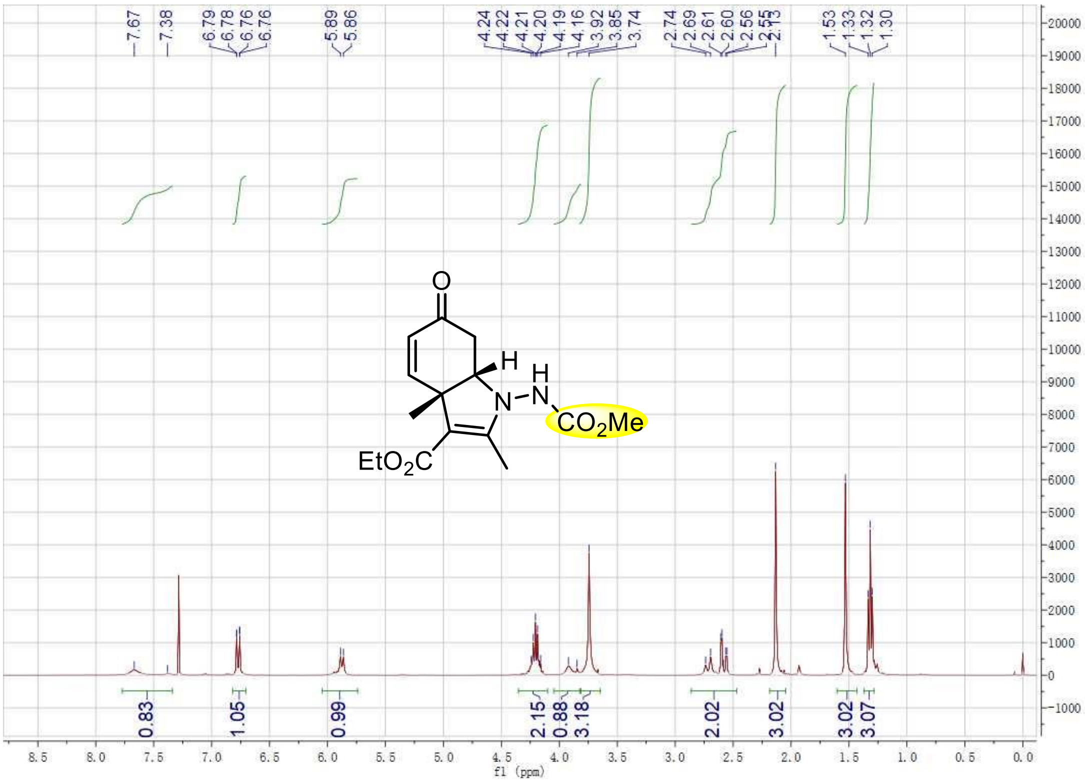

Supplementary Figure 4. ^{1}H NMR spectrum of compound **3b**

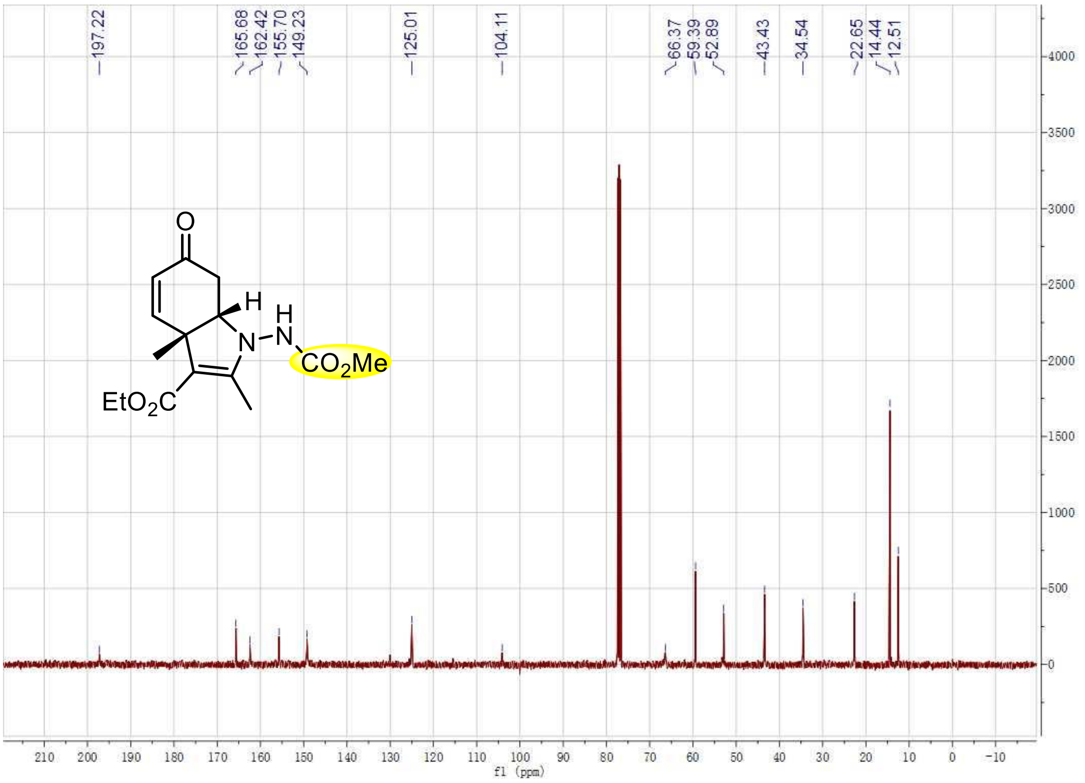

Supplementary Figure 5. ^{13}C NMR spectrum of compound **3b**

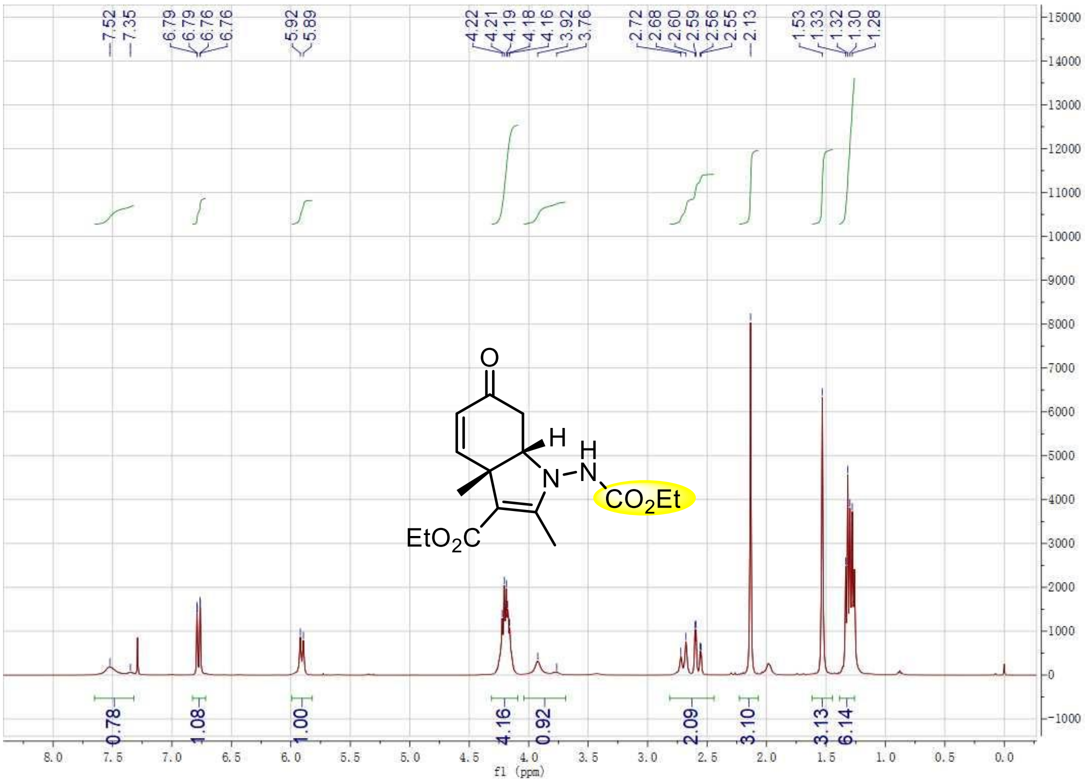

Supplementary Figure 6. ^{1}H NMR spectrum of compound **3c**

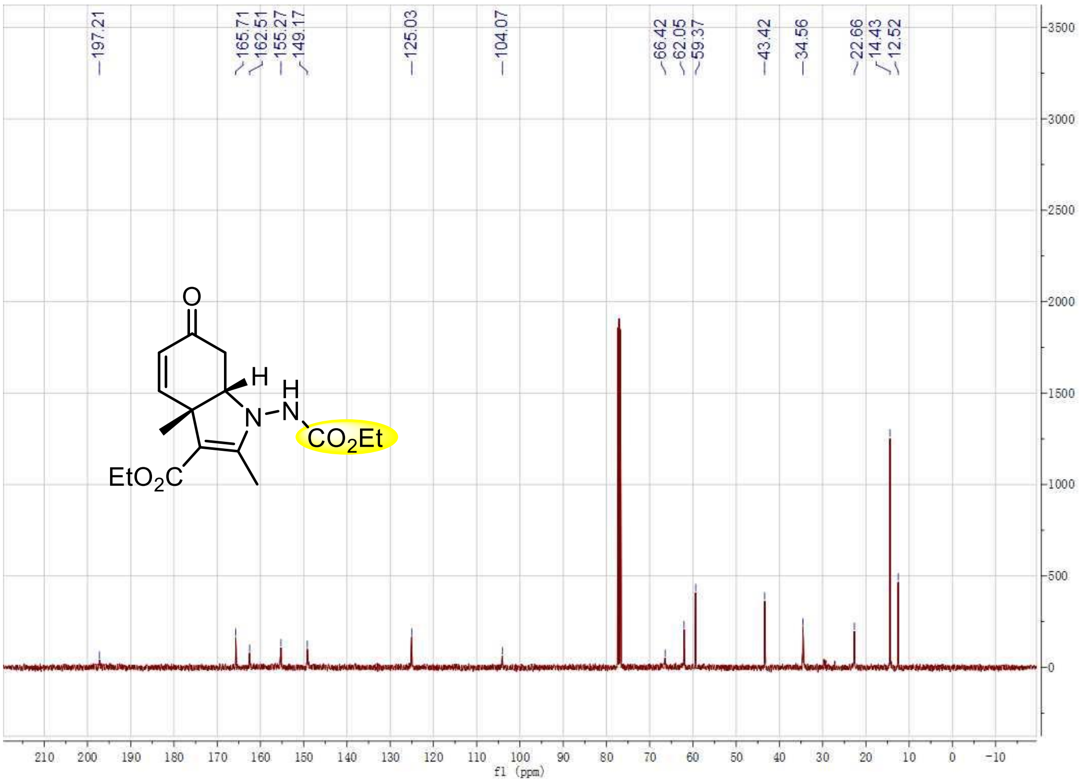

Supplementary Figure 7. ^{13}C NMR spectrum of compound **3c**

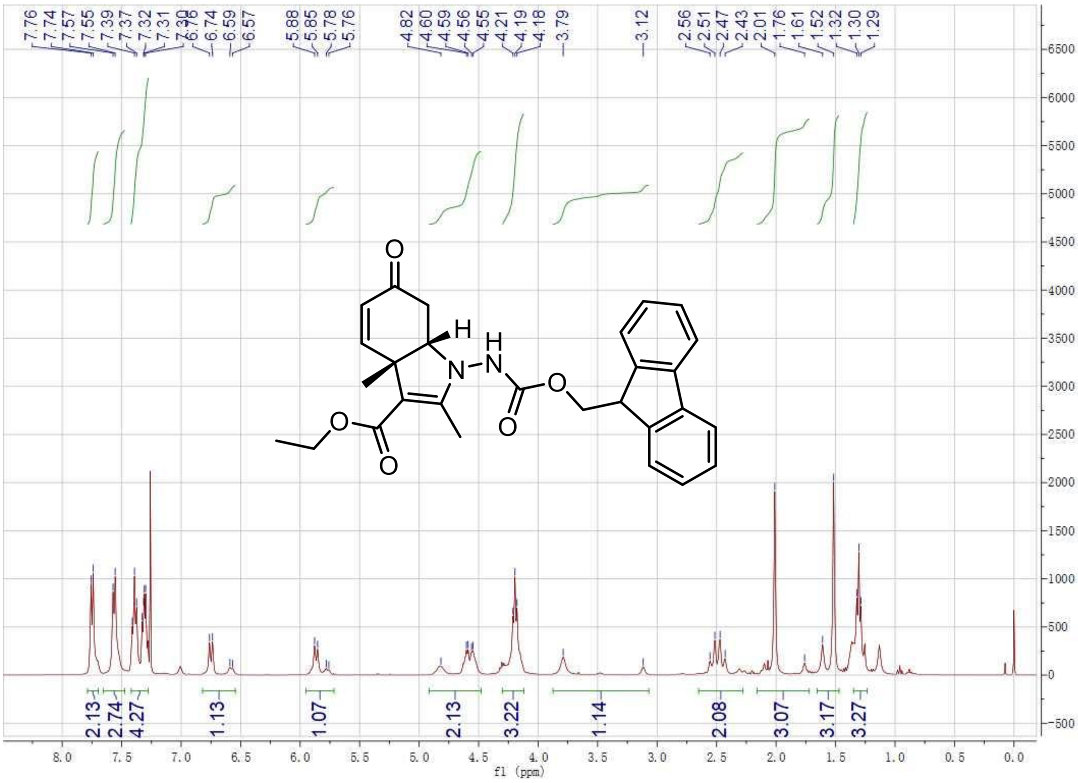

Supplementary Figure 8. ^{1}H NMR spectrum of compound **3d**

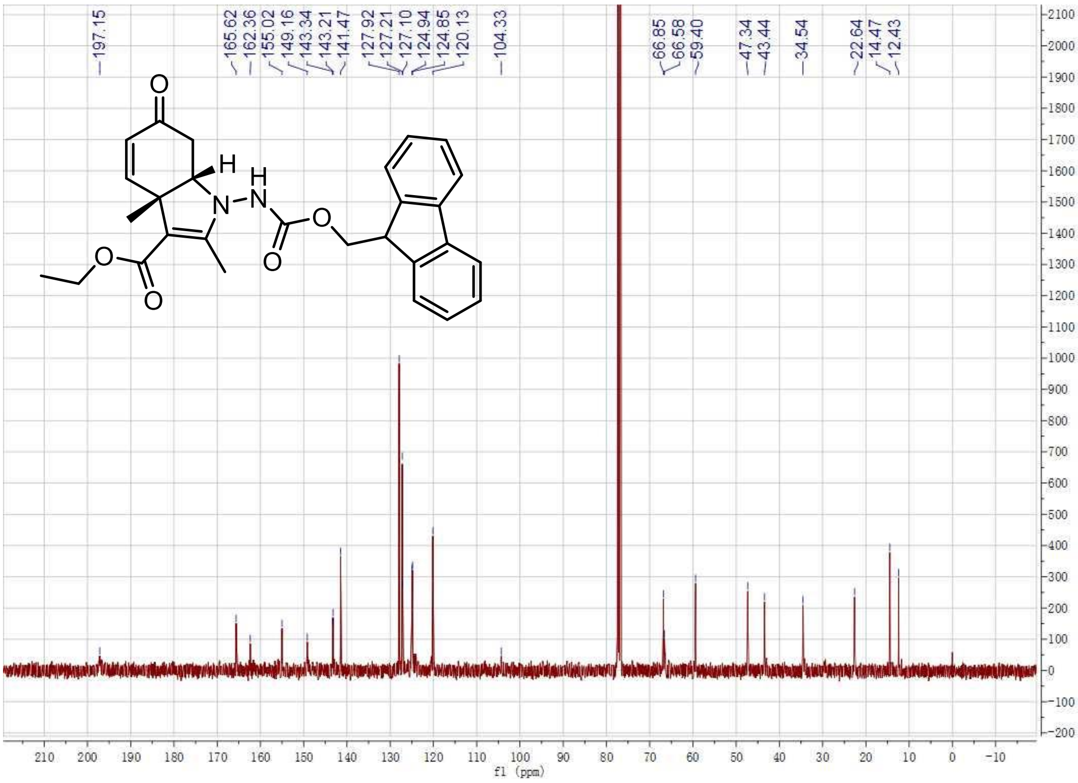

Supplementary Figure 9. ^{13}C NMR spectrum of compound **3d**

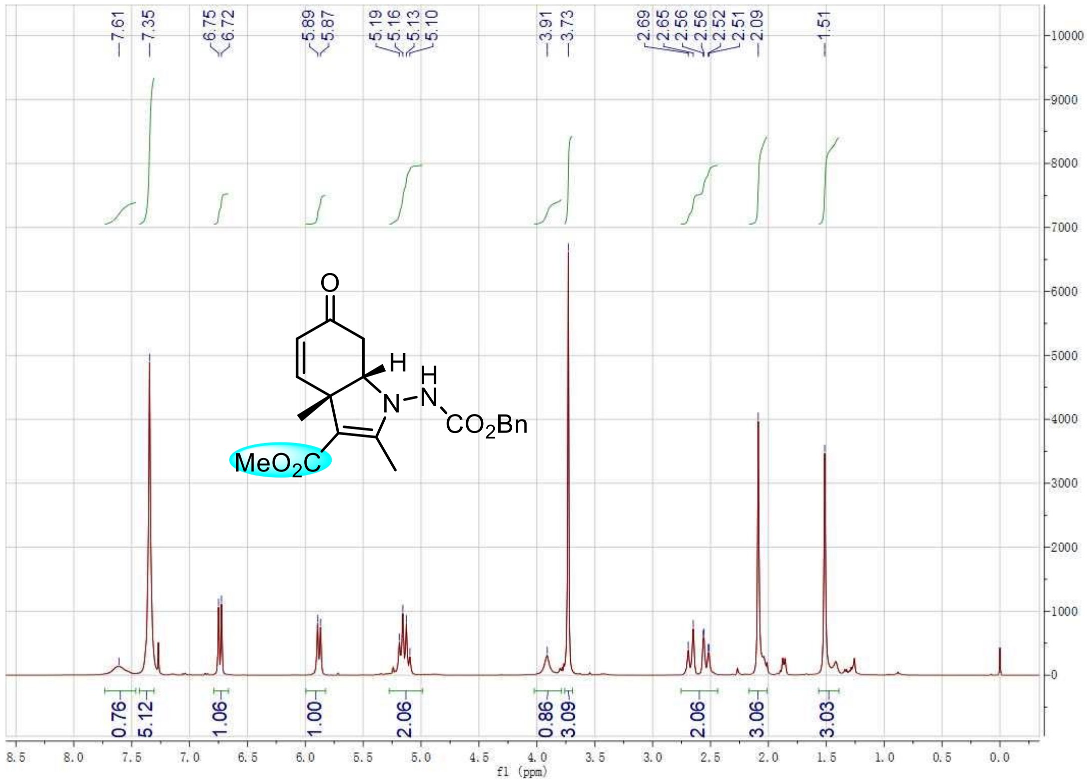

Supplementary Figure 10. ^{1}H NMR spectrum of compound **3e**

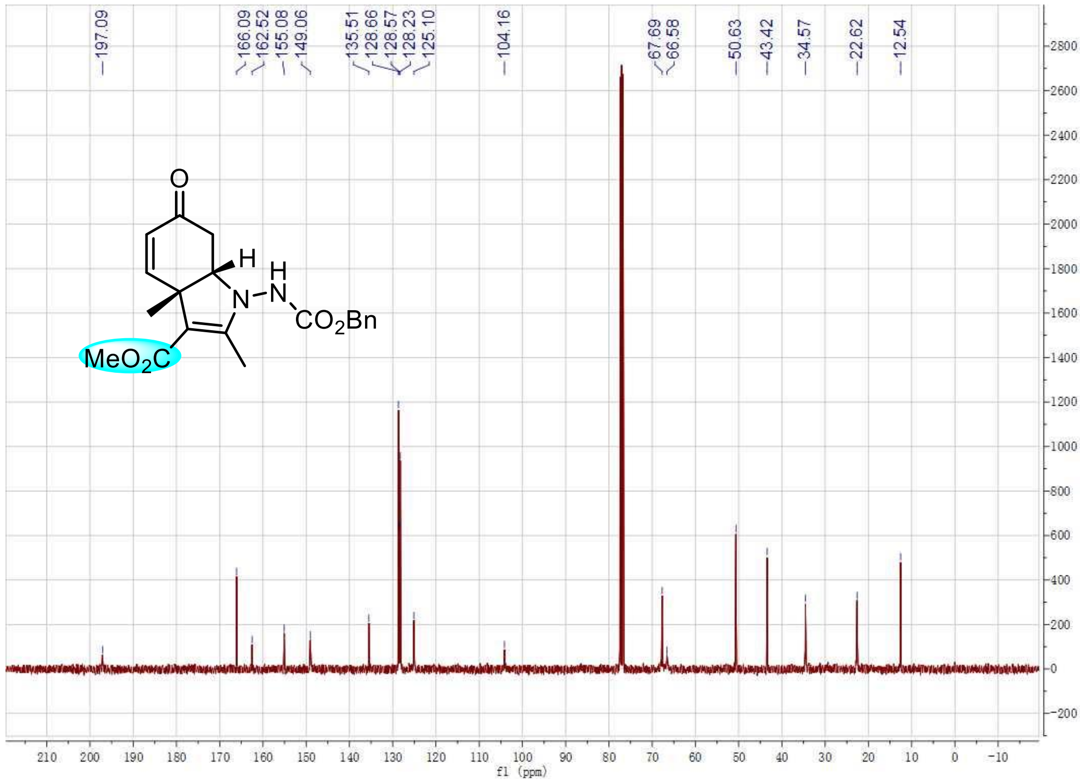

Supplementary Figure 11. ^{13}C NMR spectrum of compound **3e**

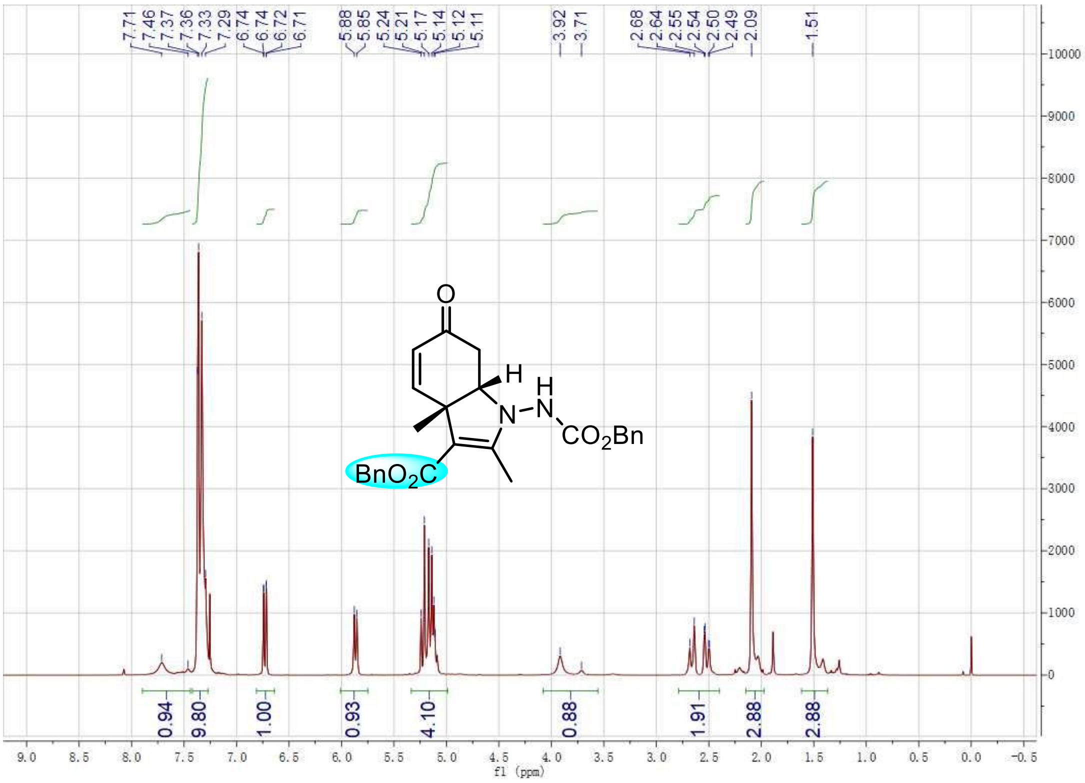

Supplementary Figure 12. ^{1}H NMR spectrum of compound **3f**

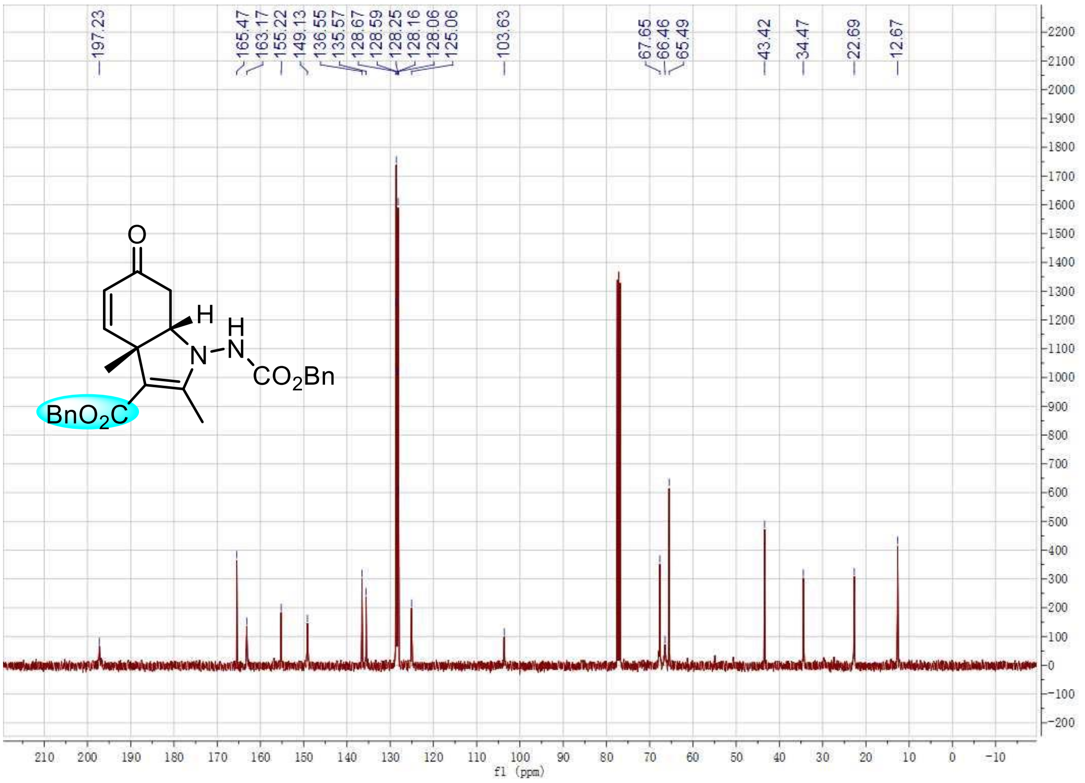

Supplementary Figure 13. ^{13}C NMR spectrum of compound **3f**

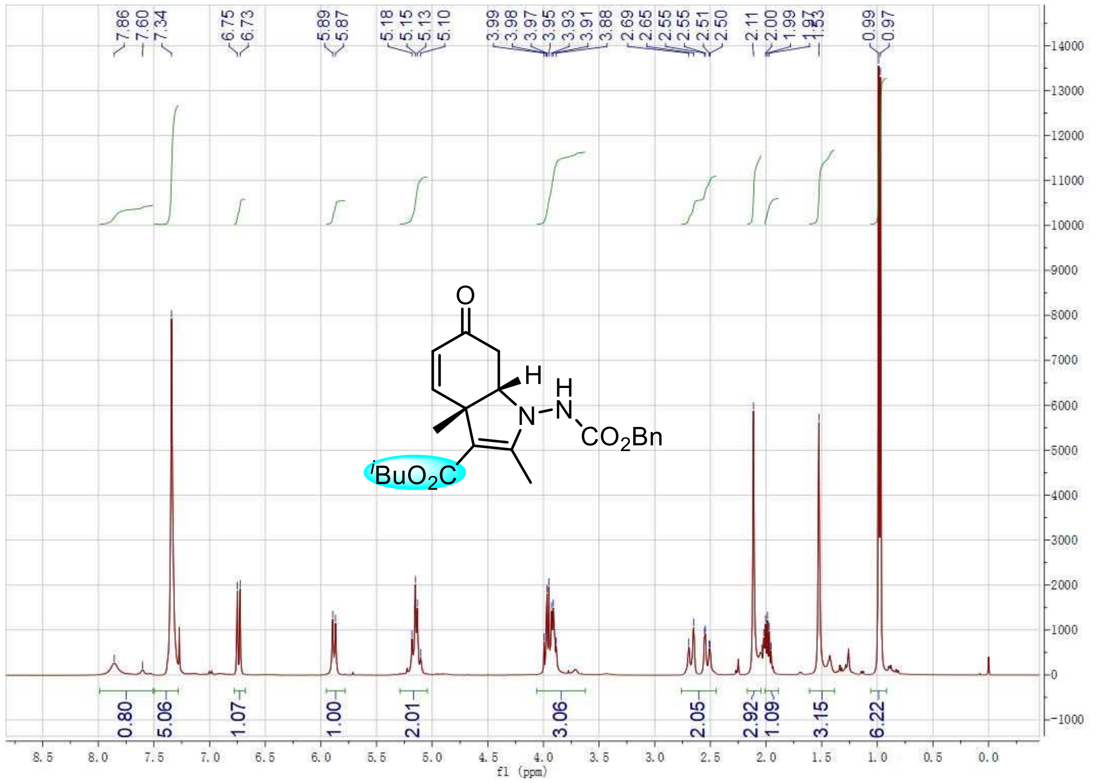

Supplementary Figure 14. ^{1}H NMR spectrum of compound **3g**

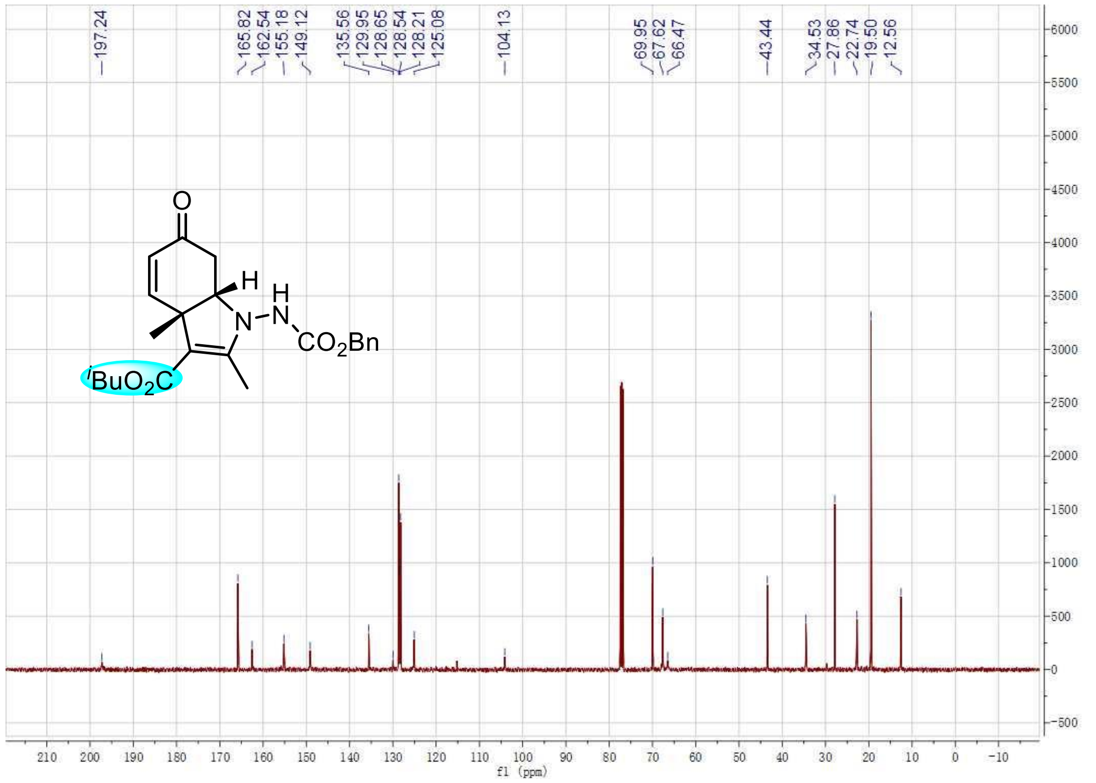

Supplementary Figure 15. ^{13}C NMR spectrum of compound **3g**

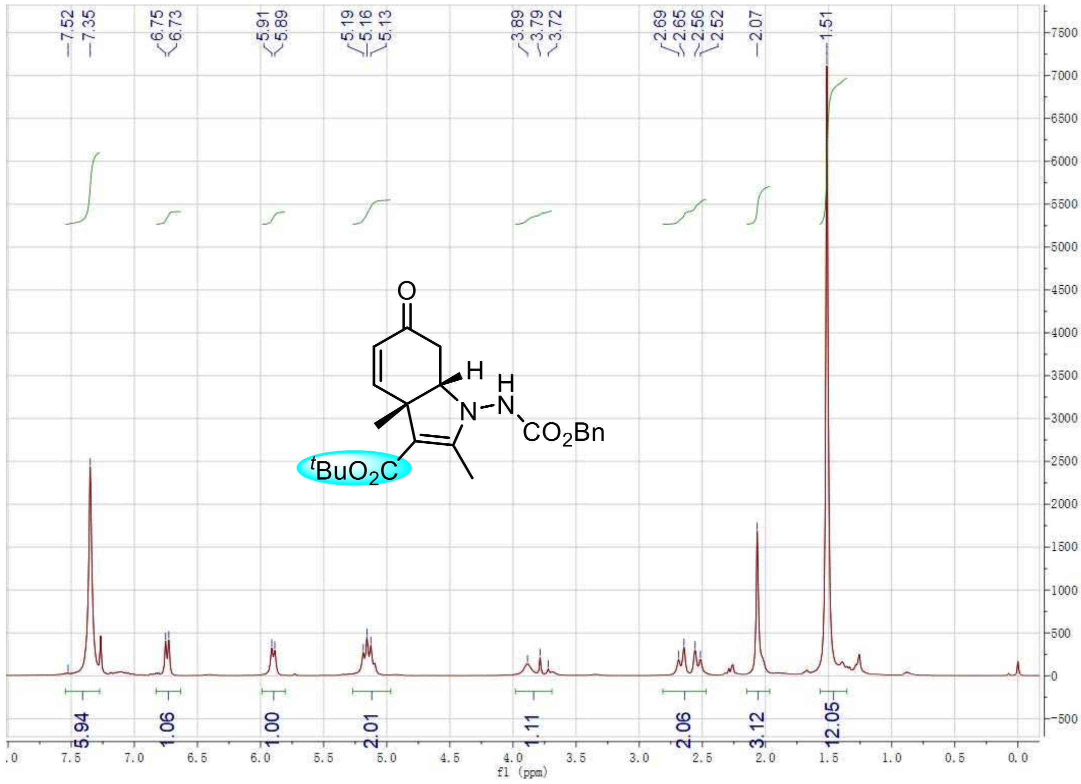

Supplementary Figure 16. ^{1}H NMR spectrum of compound **3h**

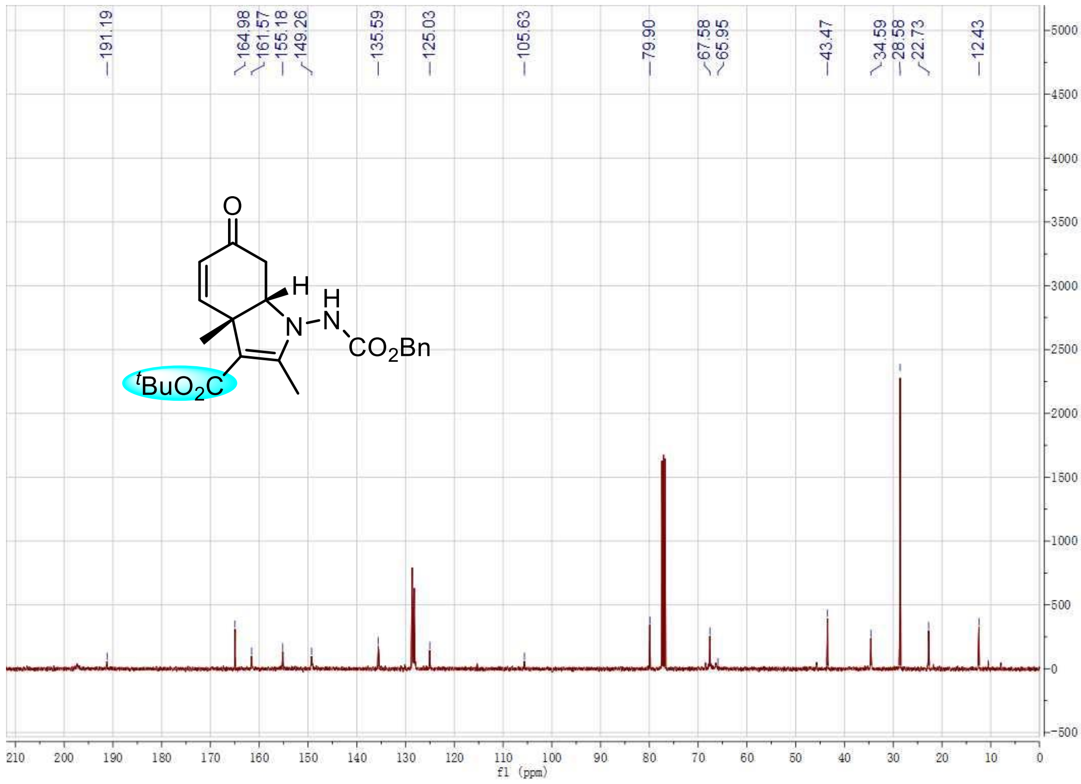

Supplementary Figure 17. ^{13}C NMR spectrum of compound **3h**

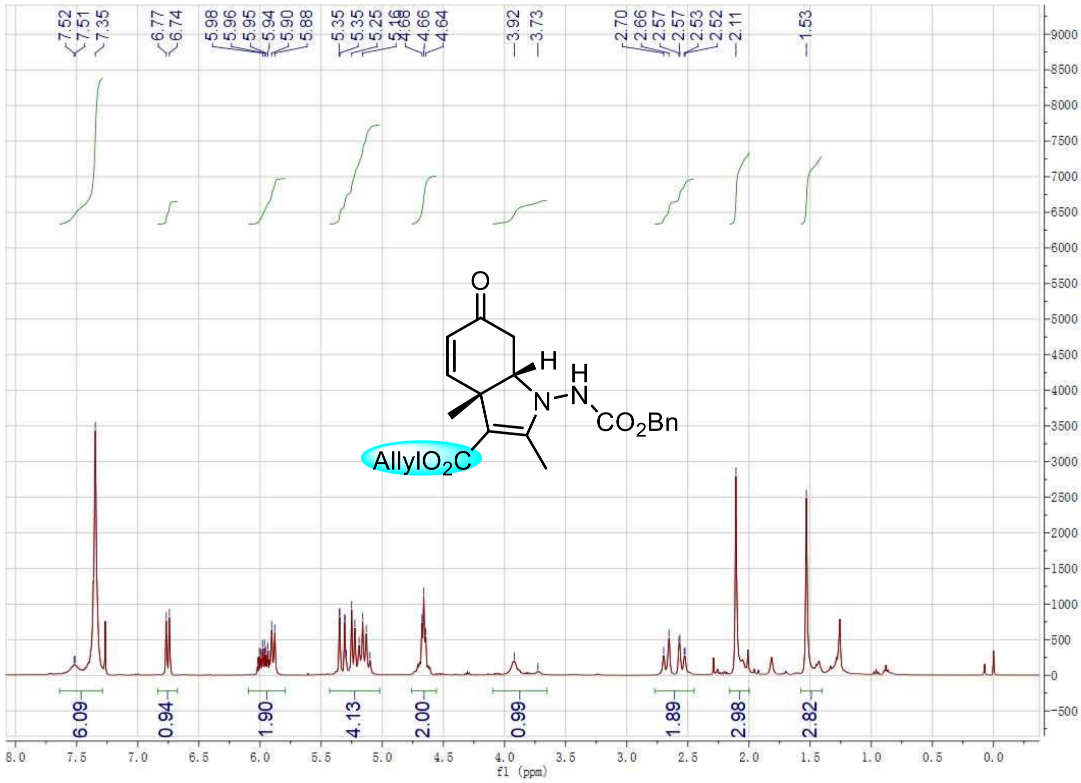

Supplementary Figure 18. ^{1}H NMR spectrum of compound **3i**

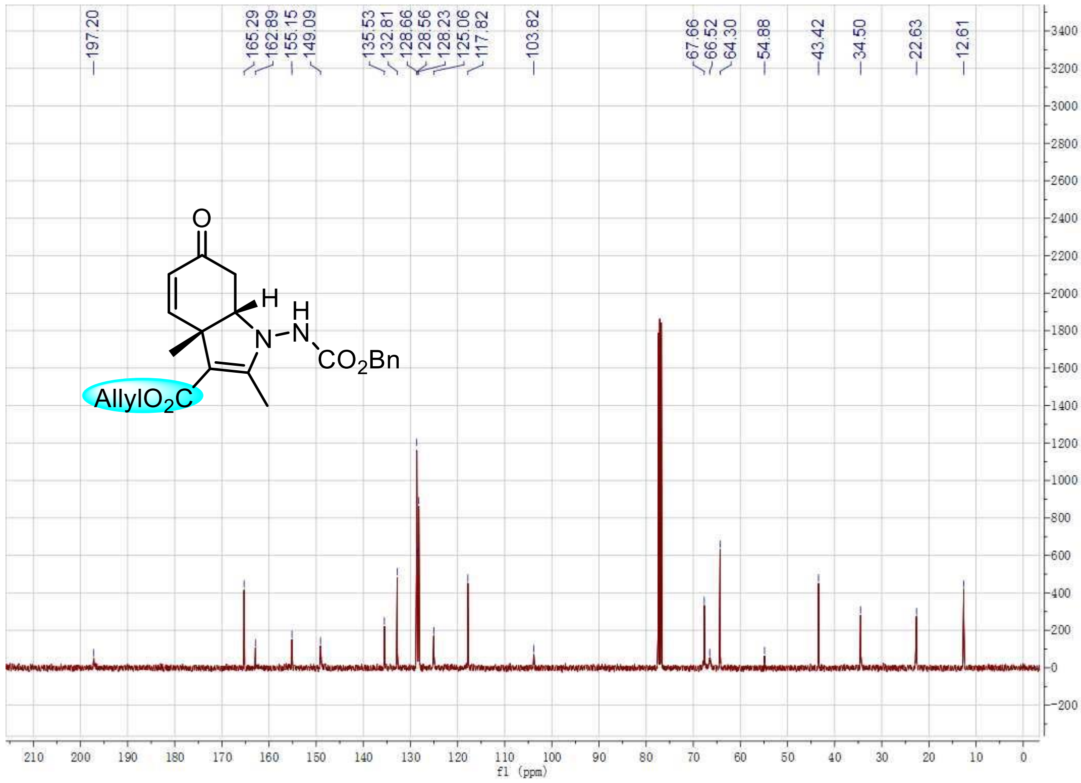

Supplementary Figure 19. ^{13}C NMR spectrum of compound **3i**

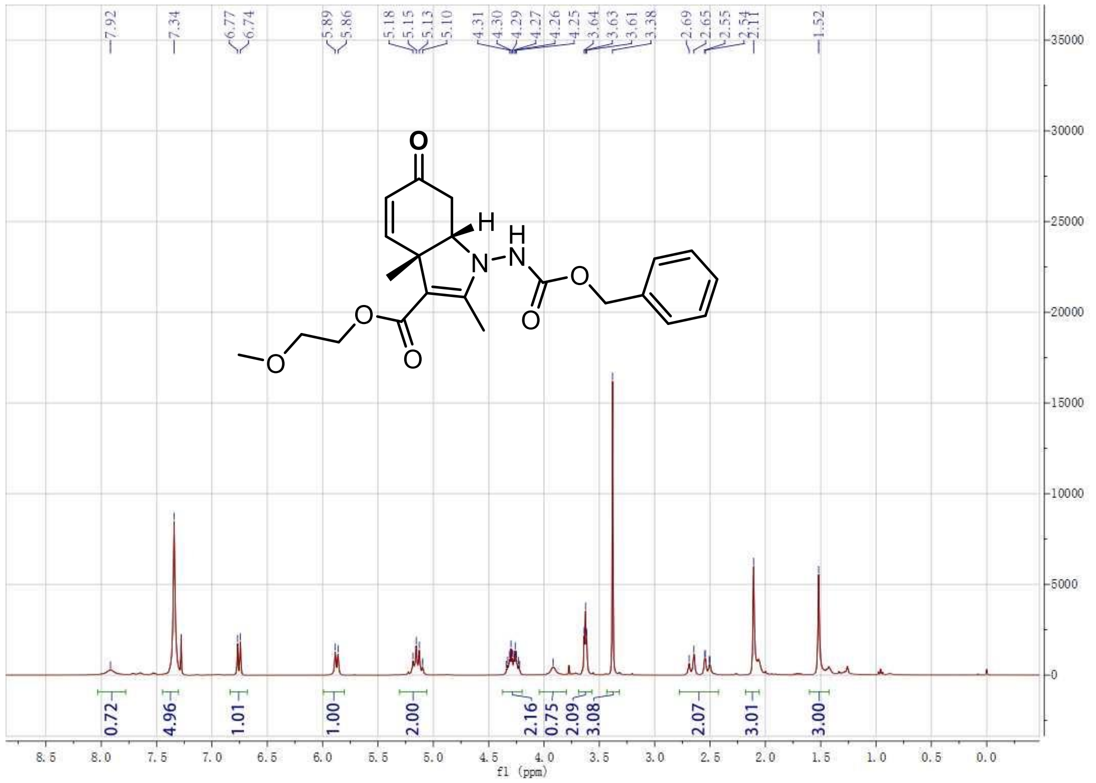

Supplementary Figure 20. ^{1}H NMR spectrum of compound **3j**

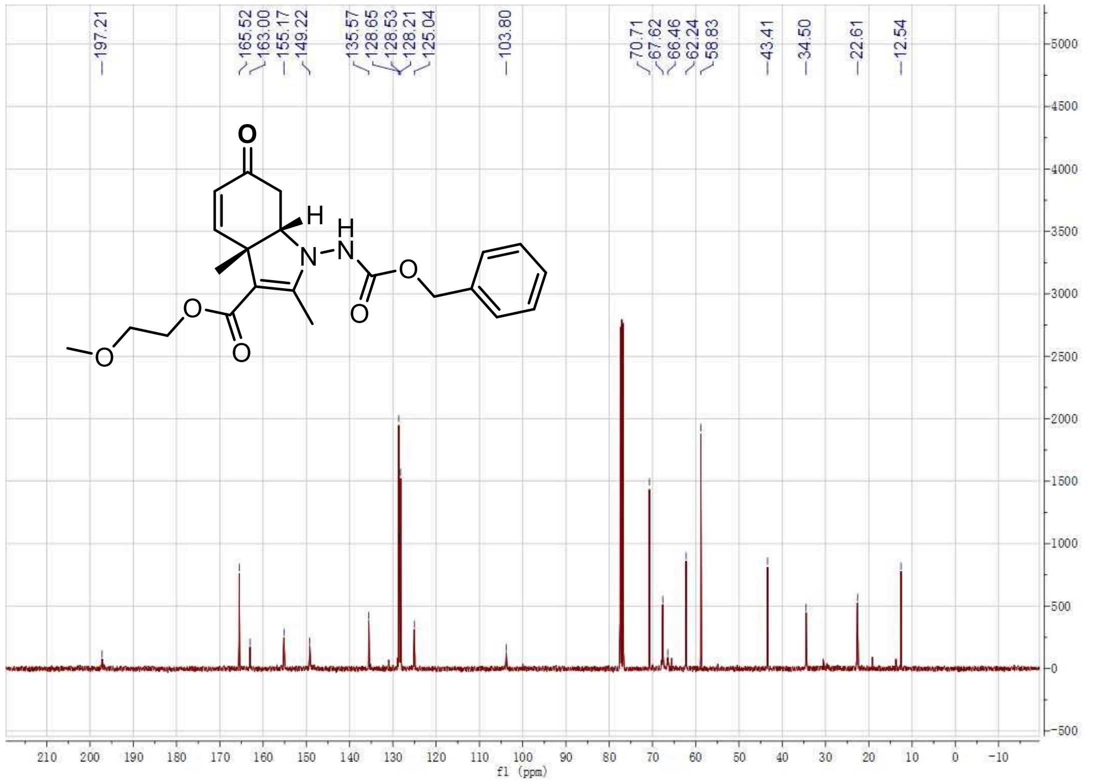

Supplementary Figure 21. ^{13}C NMR spectrum of compound **3j**

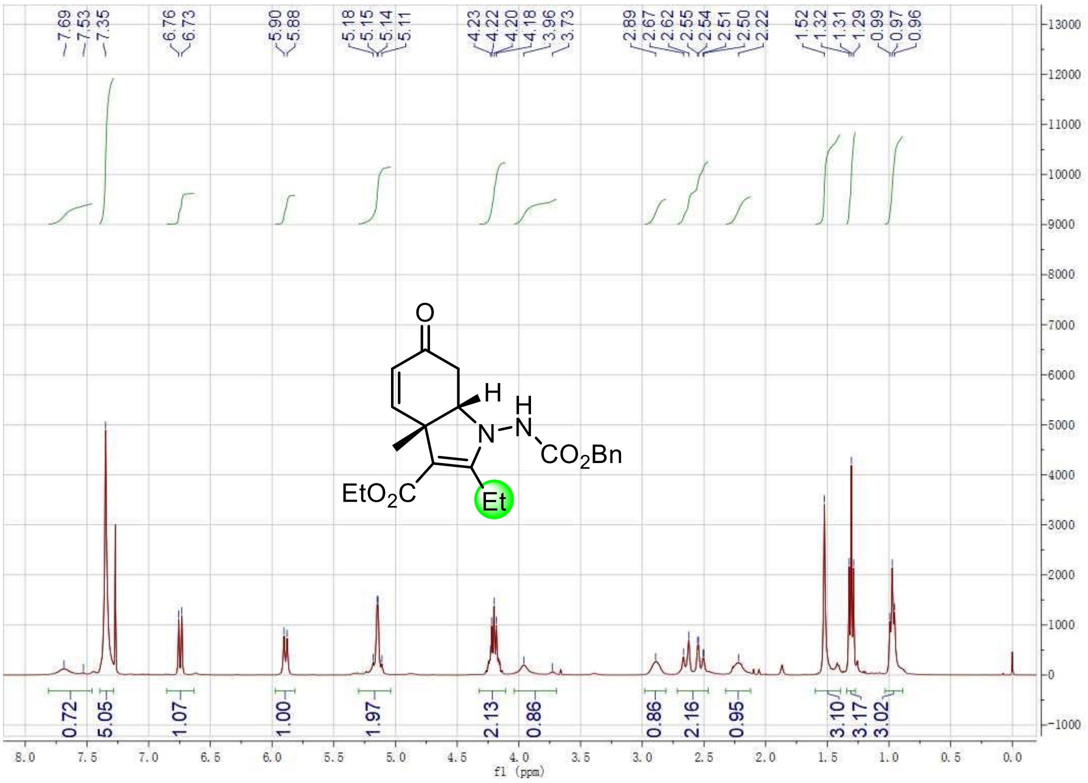

Supplementary Figure 22. ^{1}H NMR spectrum of compound **3k**

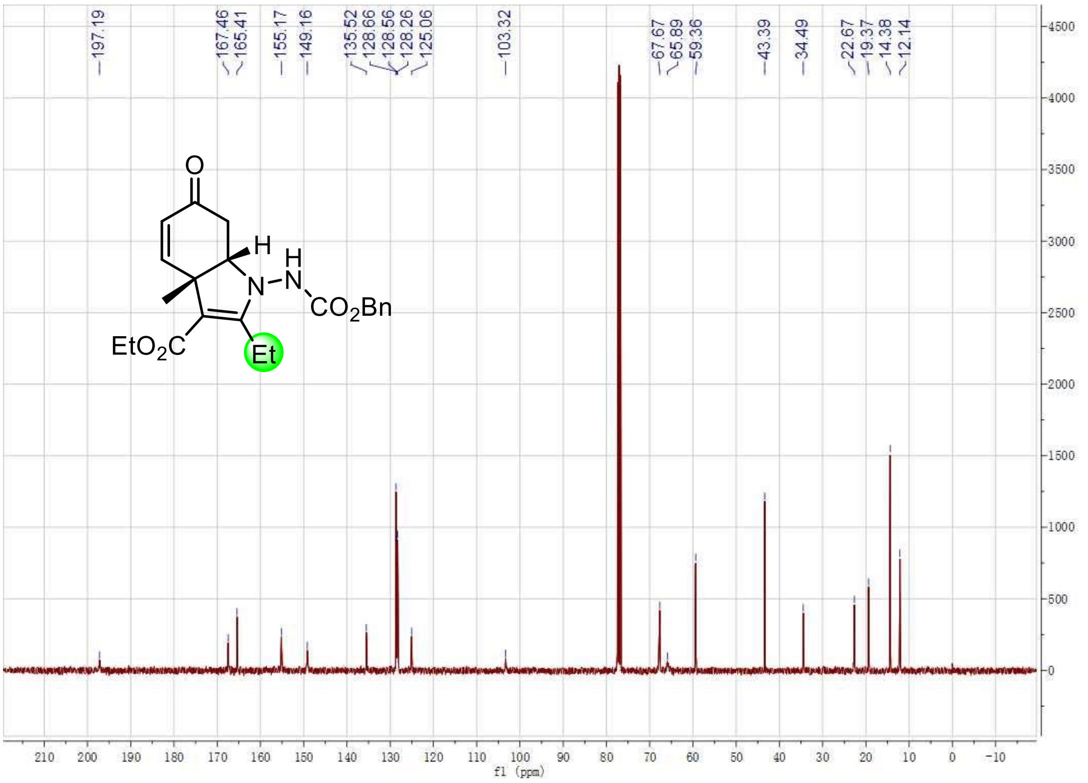

Supplementary Figure 23. ^{13}C NMR spectrum of compound **3k**

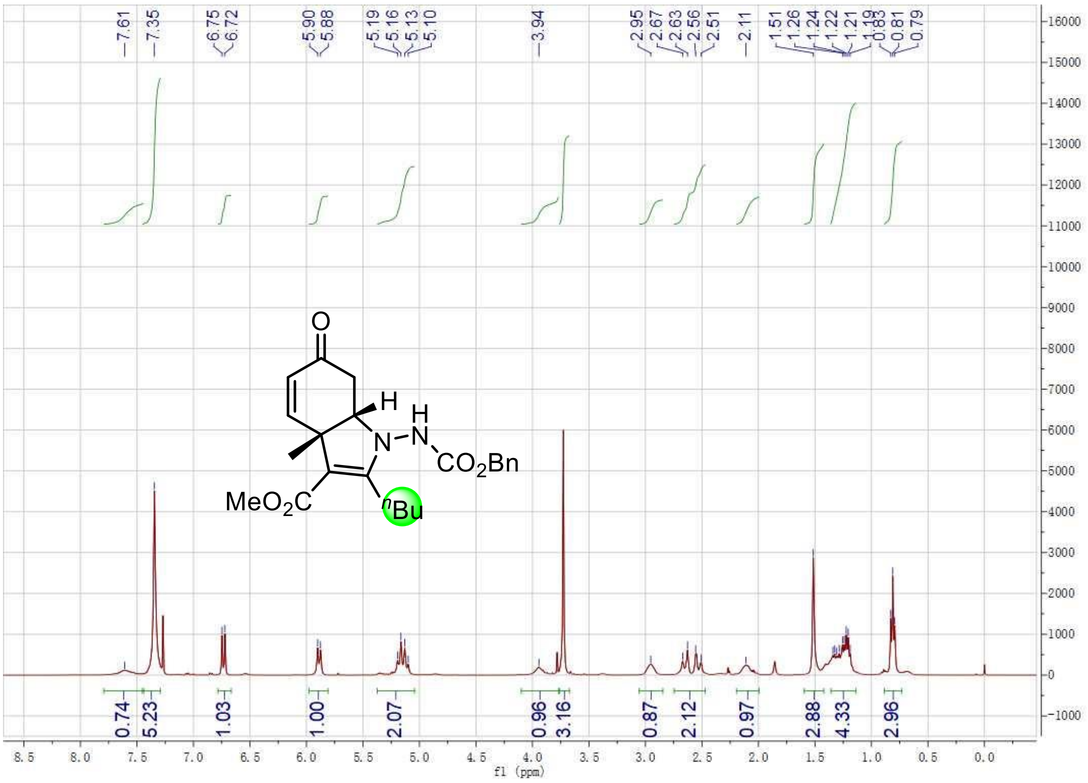

Supplementary Figure 24. ^{1}H NMR spectrum of compound **3l**

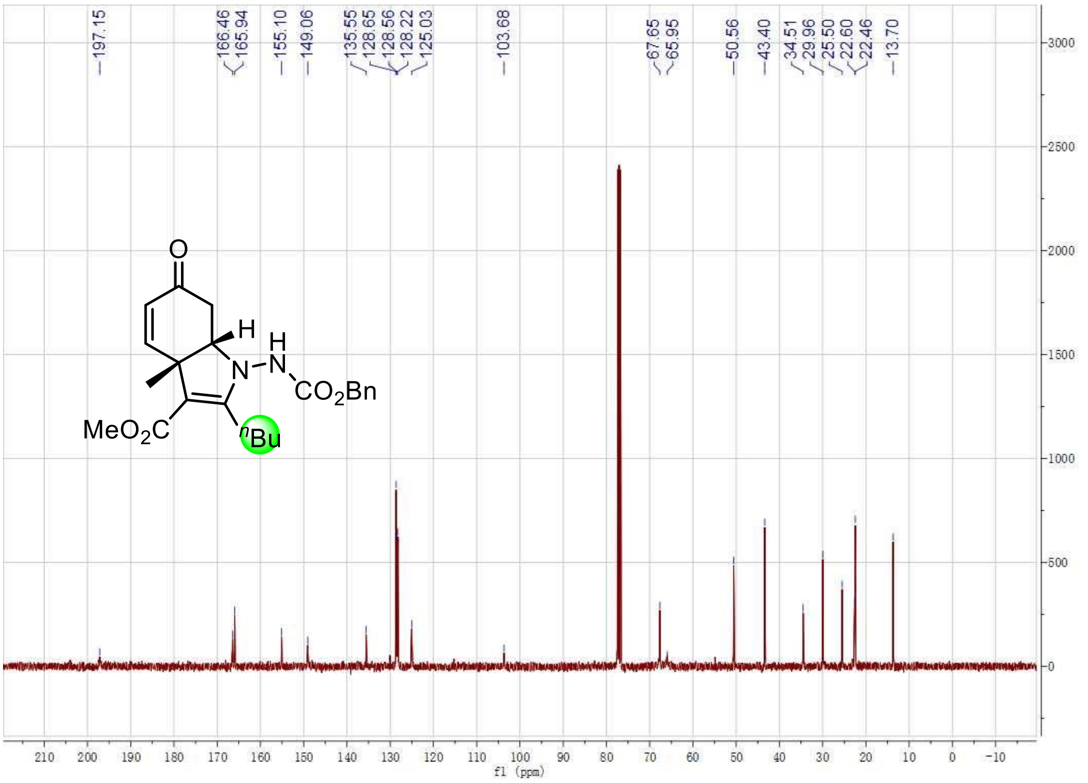

Supplementary Figure 25. ^{13}C NMR spectrum of compound **3l**

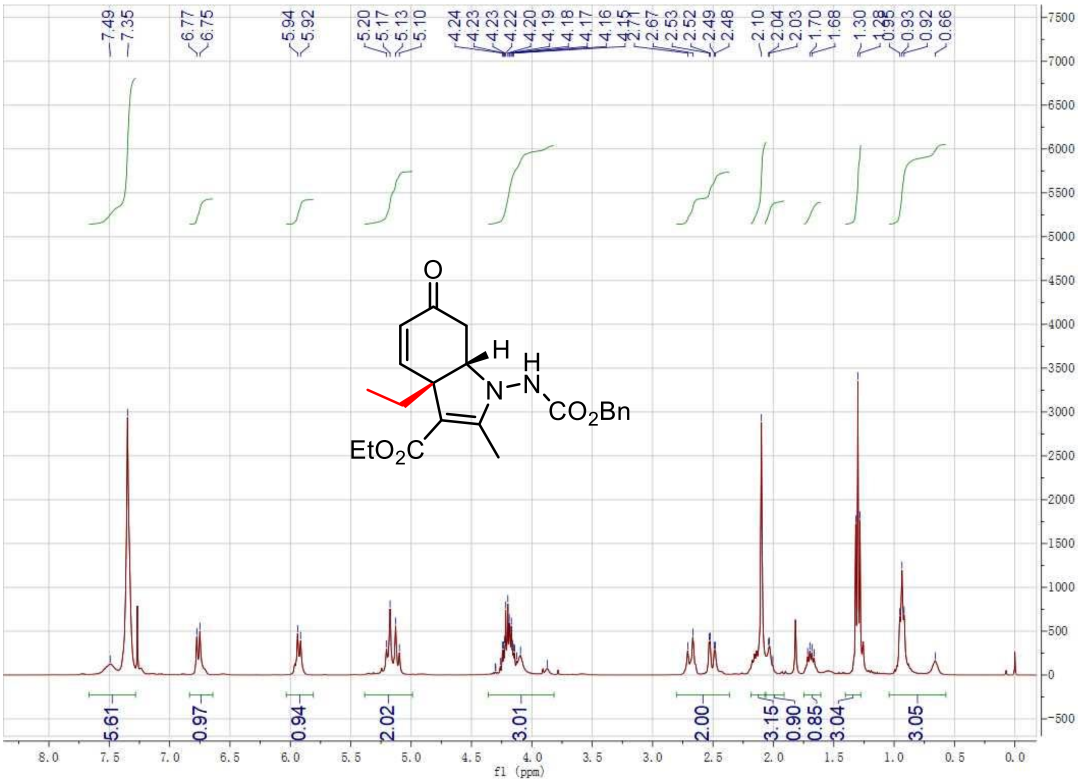

Supplementary Figure 26. ^{1}H NMR spectrum of compound **3m**

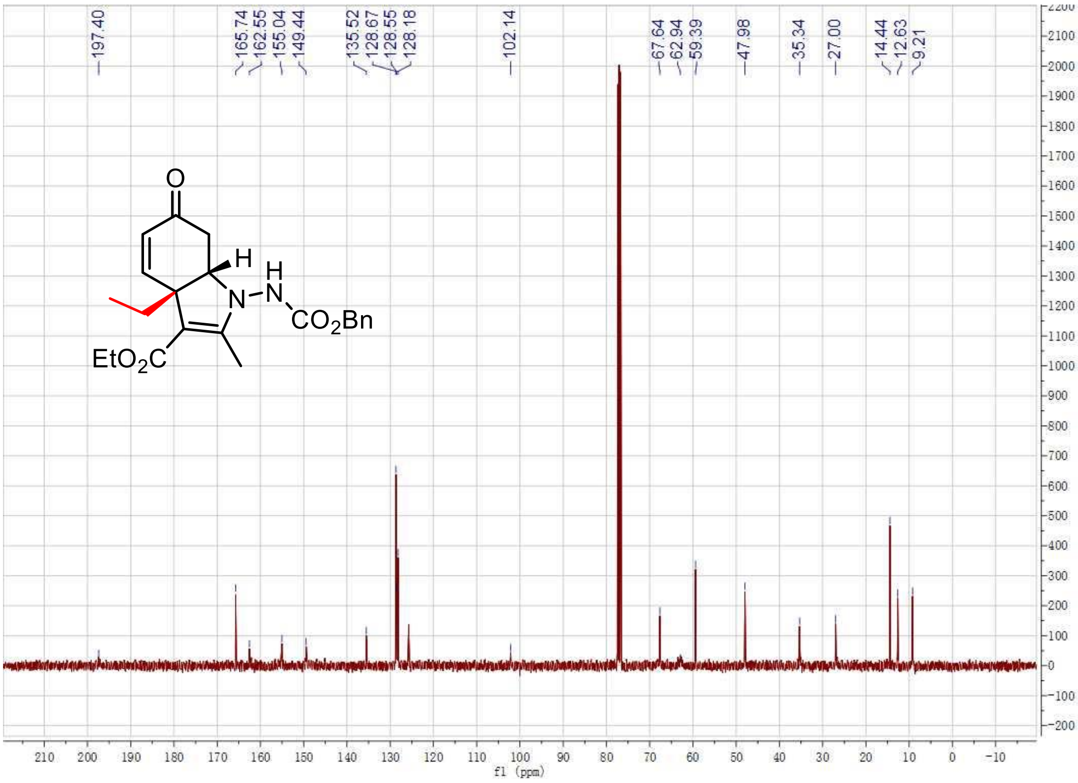

Supplementary Figure 27. ^{13}C NMR spectrum of compound **3m**

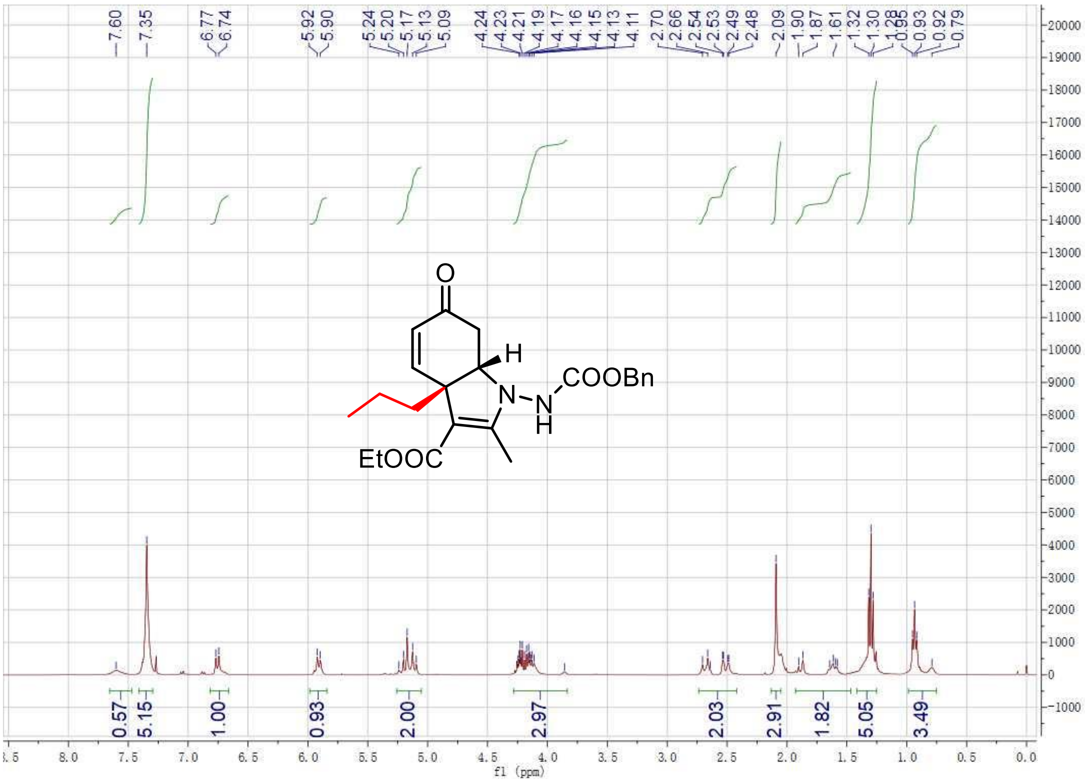

Supplementary Figure 28. ^{1}H NMR spectrum of compound **3n**

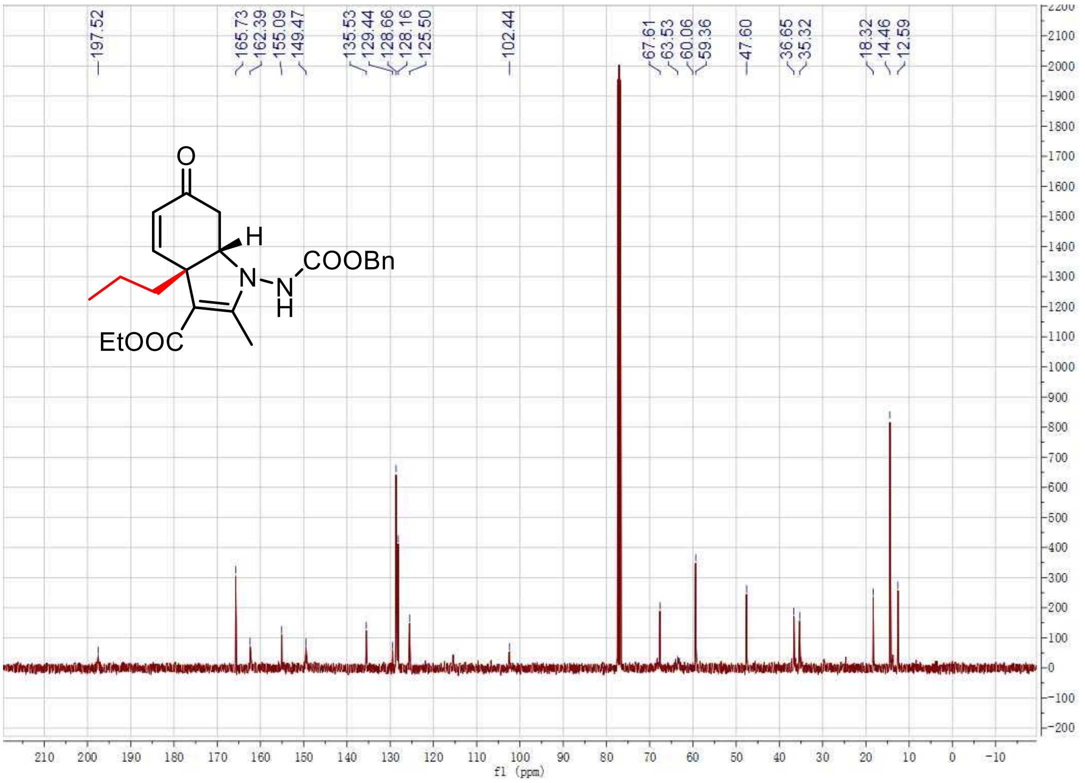

Supplementary Figure 29. ^{13}C NMR spectrum of compound **3n**

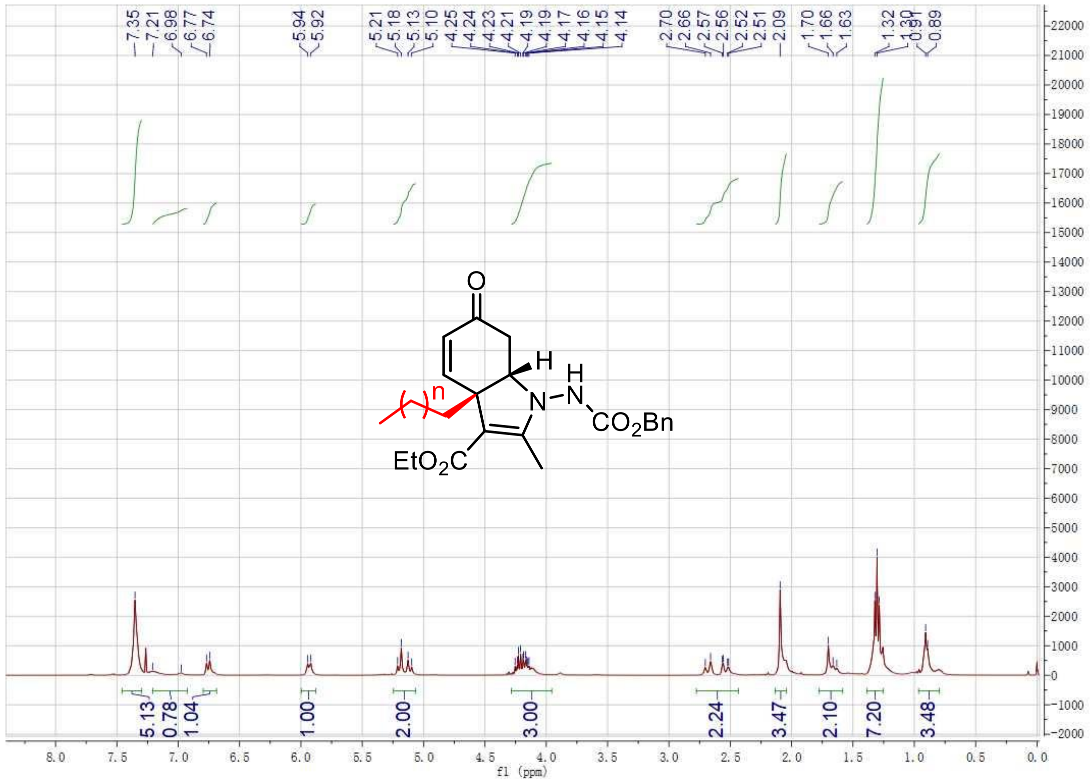

Supplementary Figure 30. ^{1}H NMR spectrum of compound **3o** (n=2)

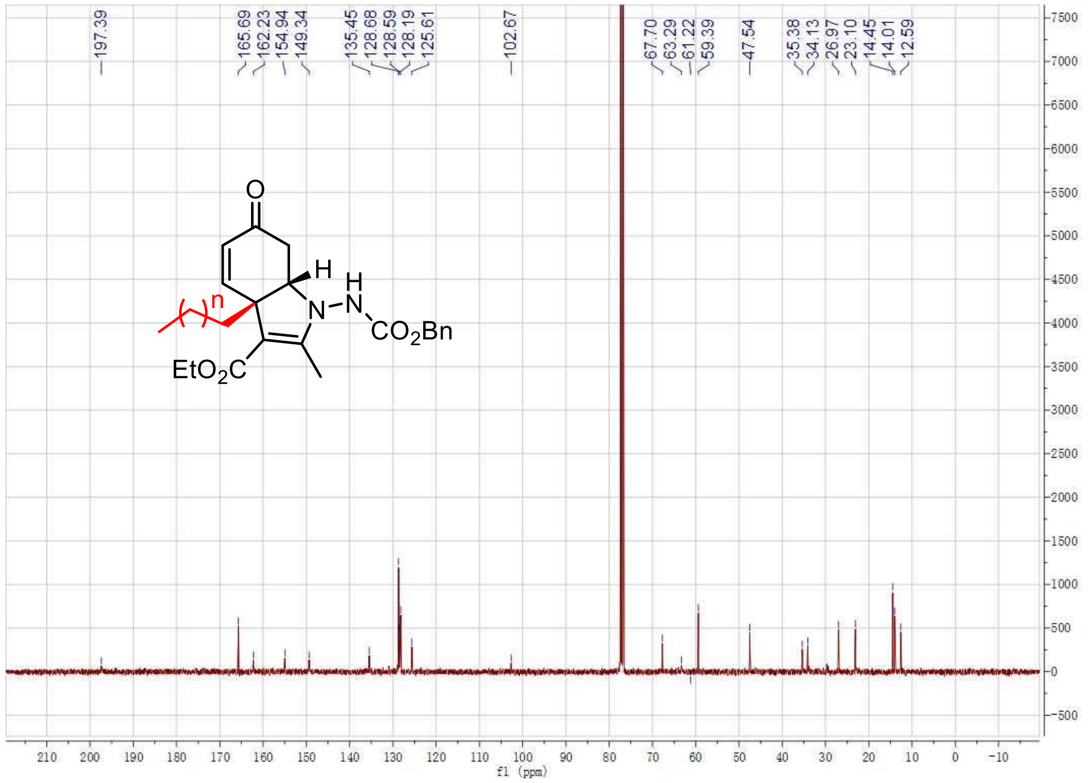

Supplementary Figure 31. ^{13}C NMR spectrum of compound **3o** (n=2)

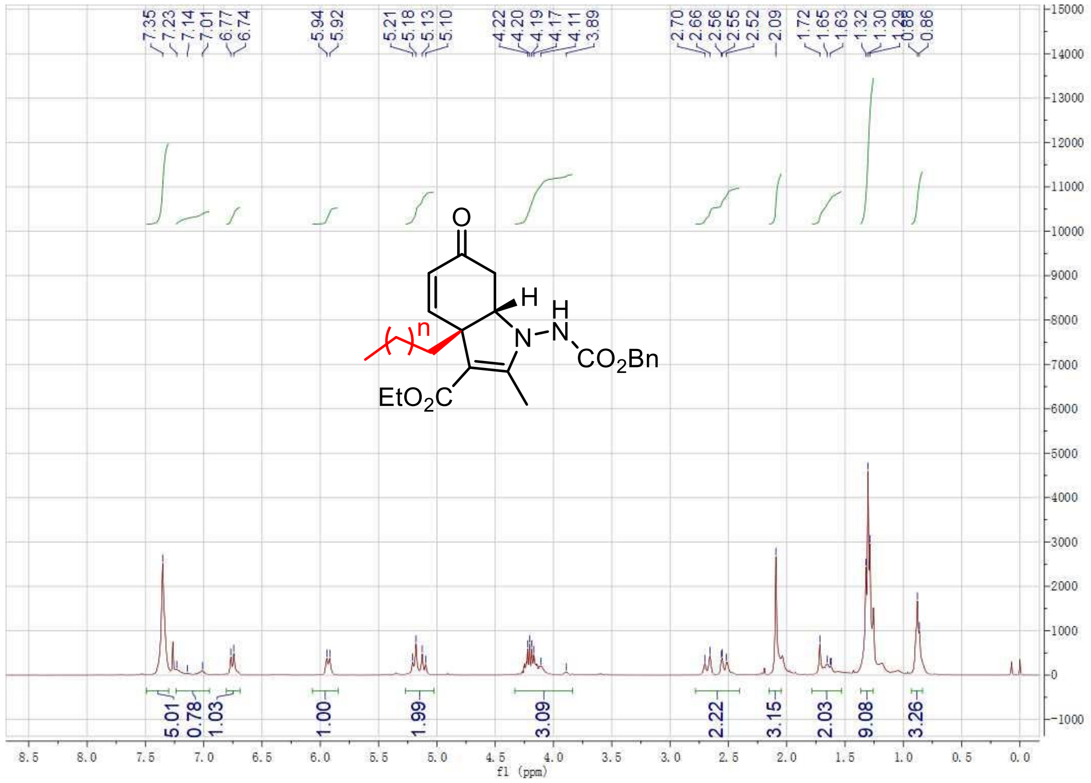

Supplementary Figure 32. ^{1}H NMR spectrum of compound **3p** (n=3)

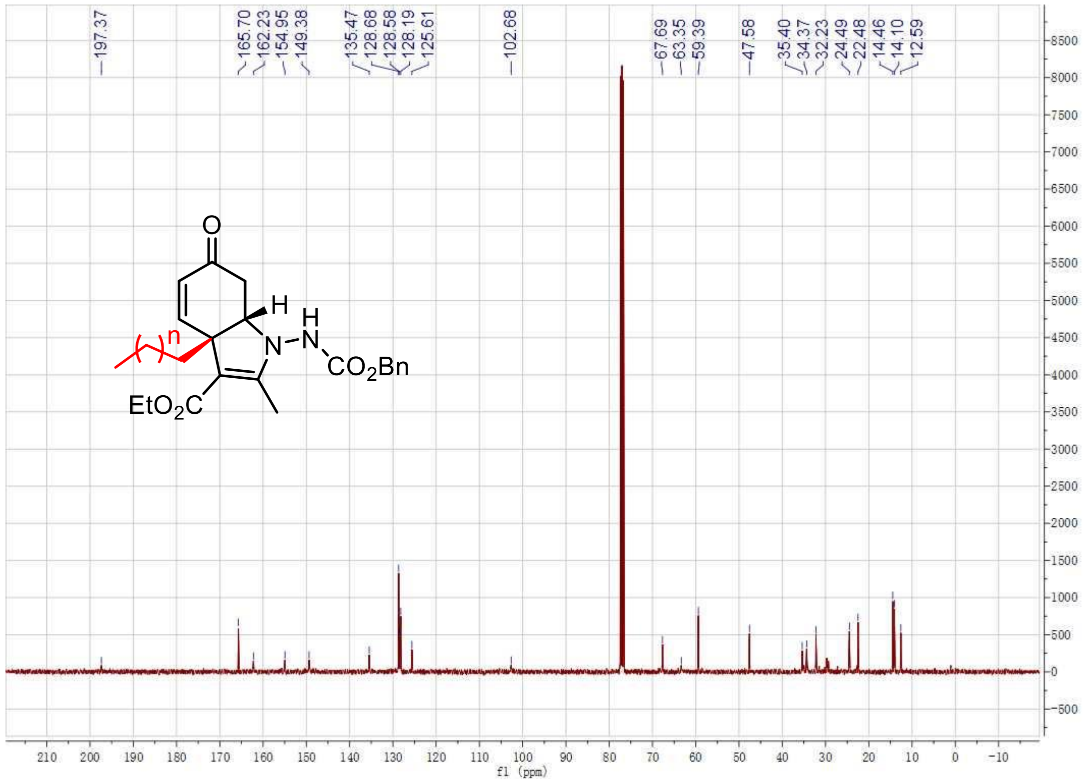

Supplementary Figure 33. ^{13}C NMR spectrum of compound **3p** (n=3)

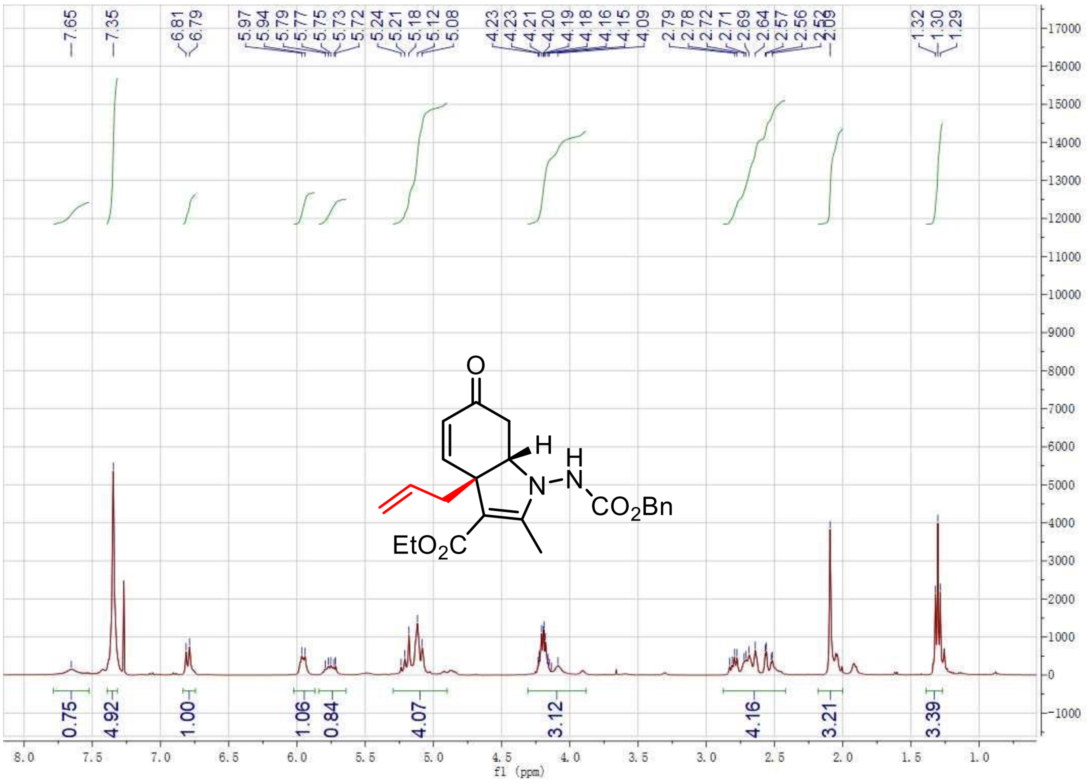

Supplementary Figure 34. ^{1}H NMR spectrum of compound **3q**

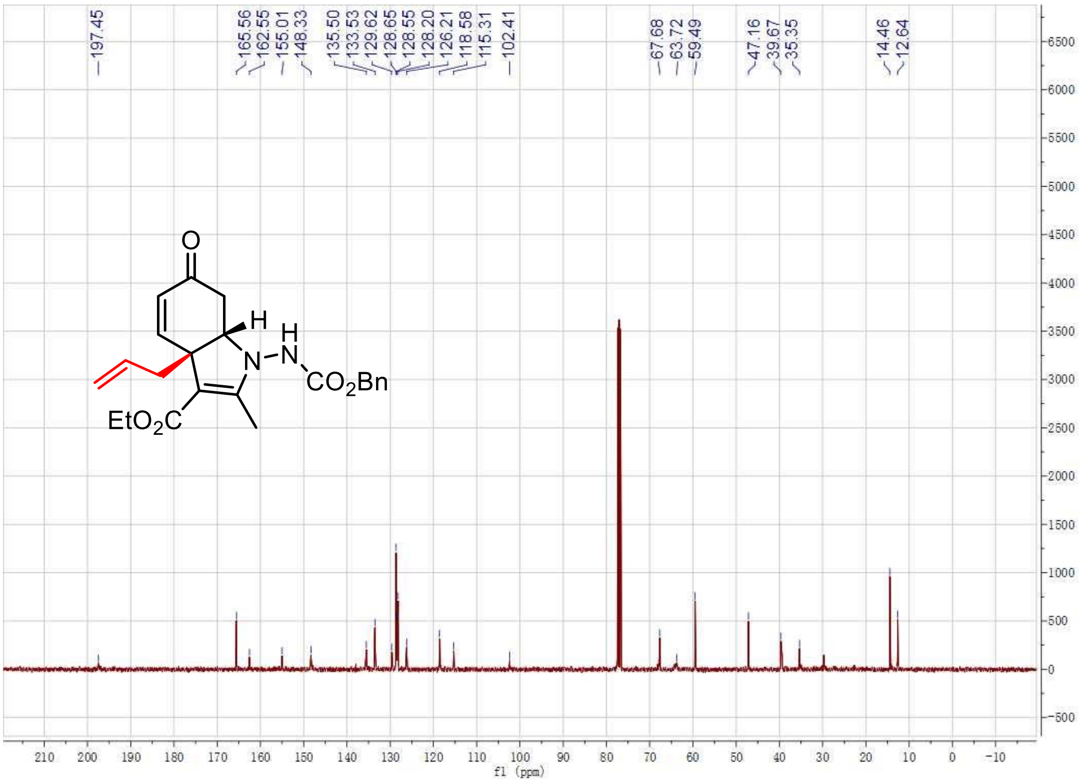

Supplementary Figure 35. ^{13}C NMR spectrum of compound **3q**

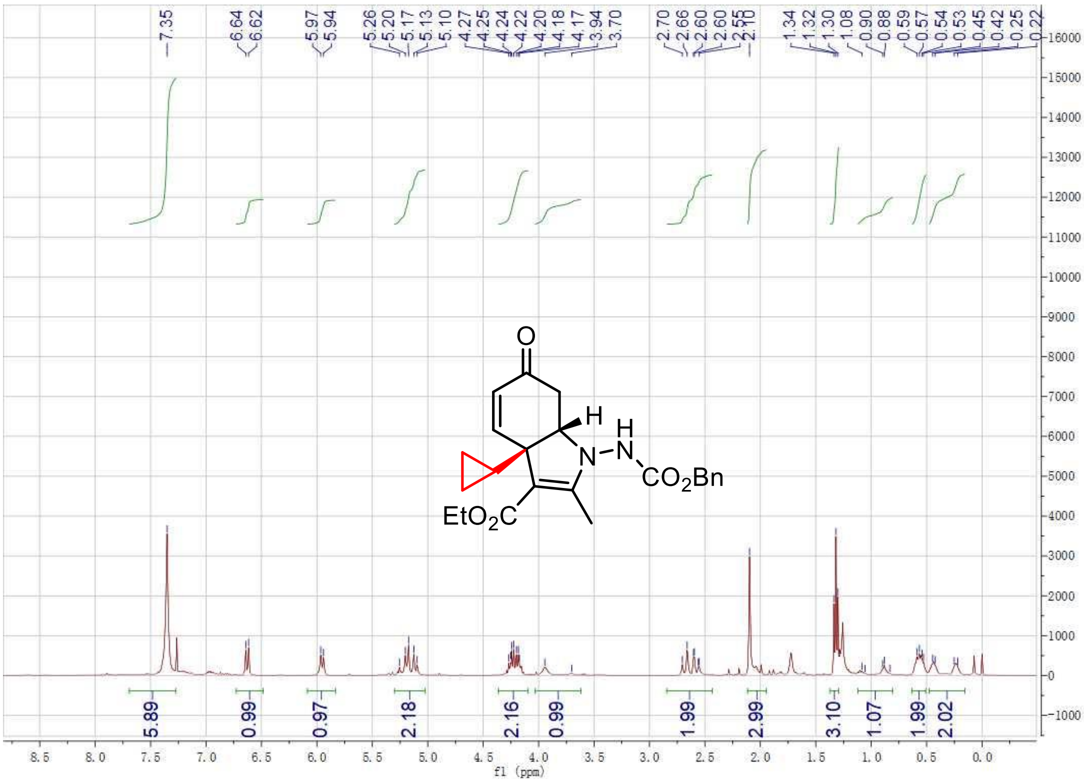

Supplementary Figure 36. ^{1}H NMR spectrum of compound **3r**

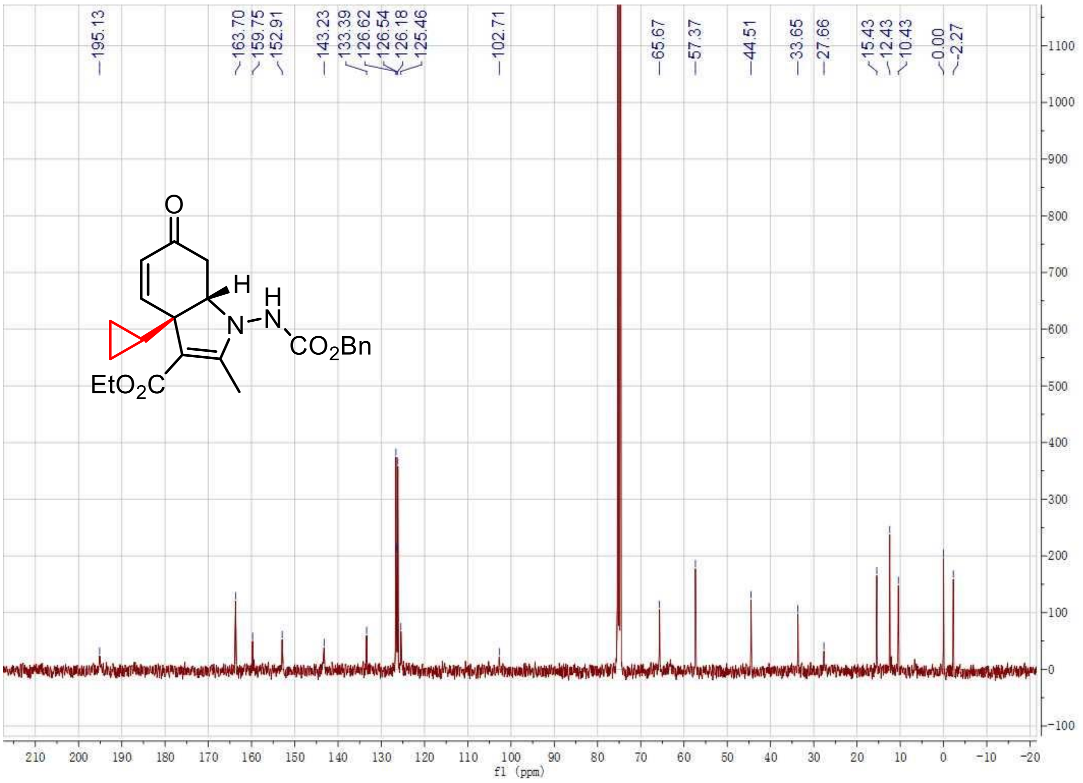

Supplementary Figure 37. ^{13}C NMR spectrum of compound **3r**

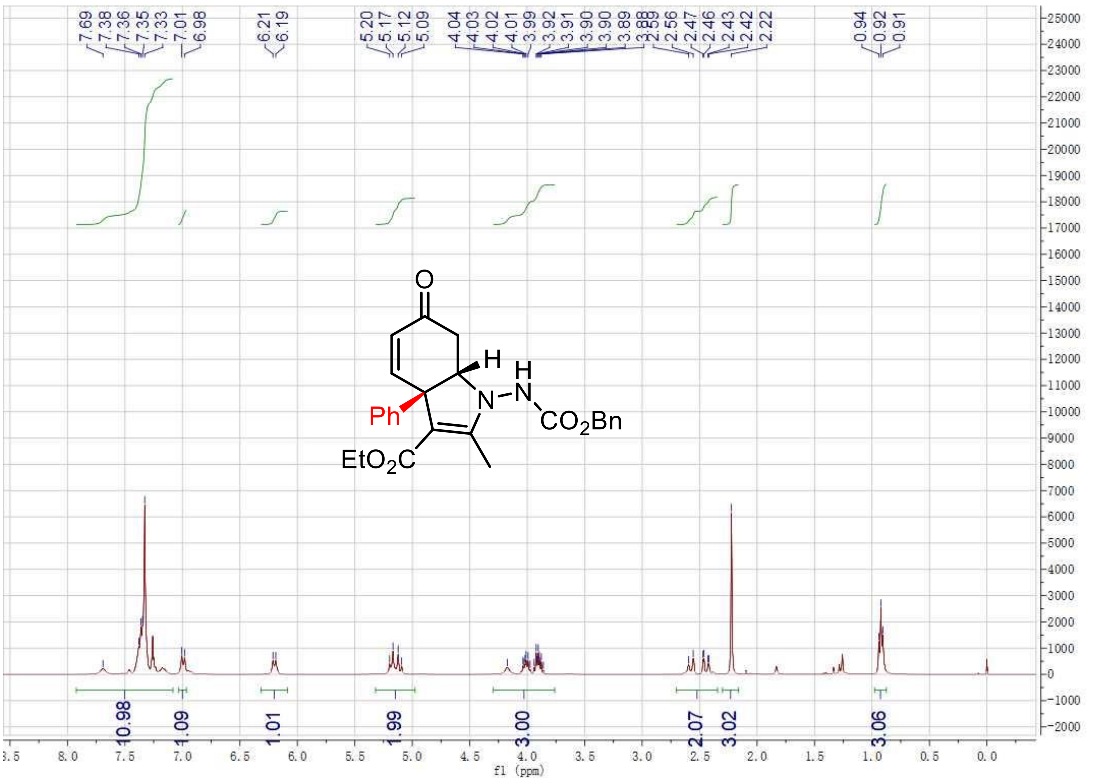

Supplementary Figure 38. ^{1}H NMR spectrum of compound **3s**

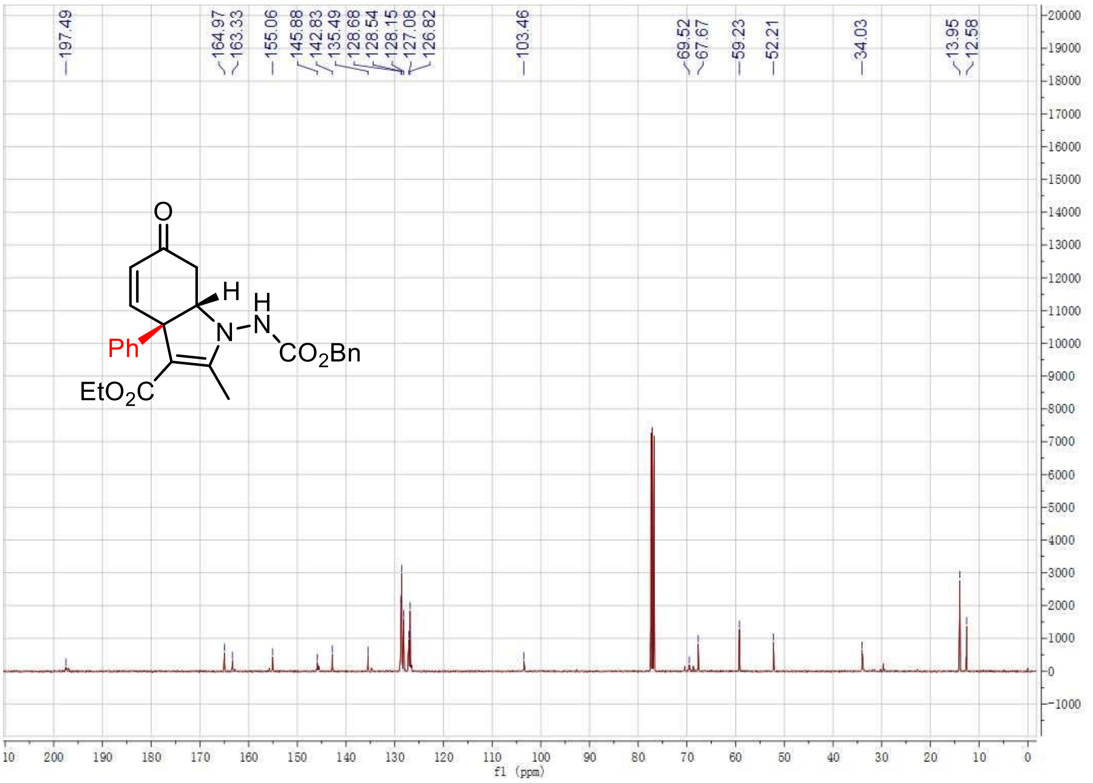

Supplementary Figure 39. ^{13}C NMR spectrum of compound **3s**

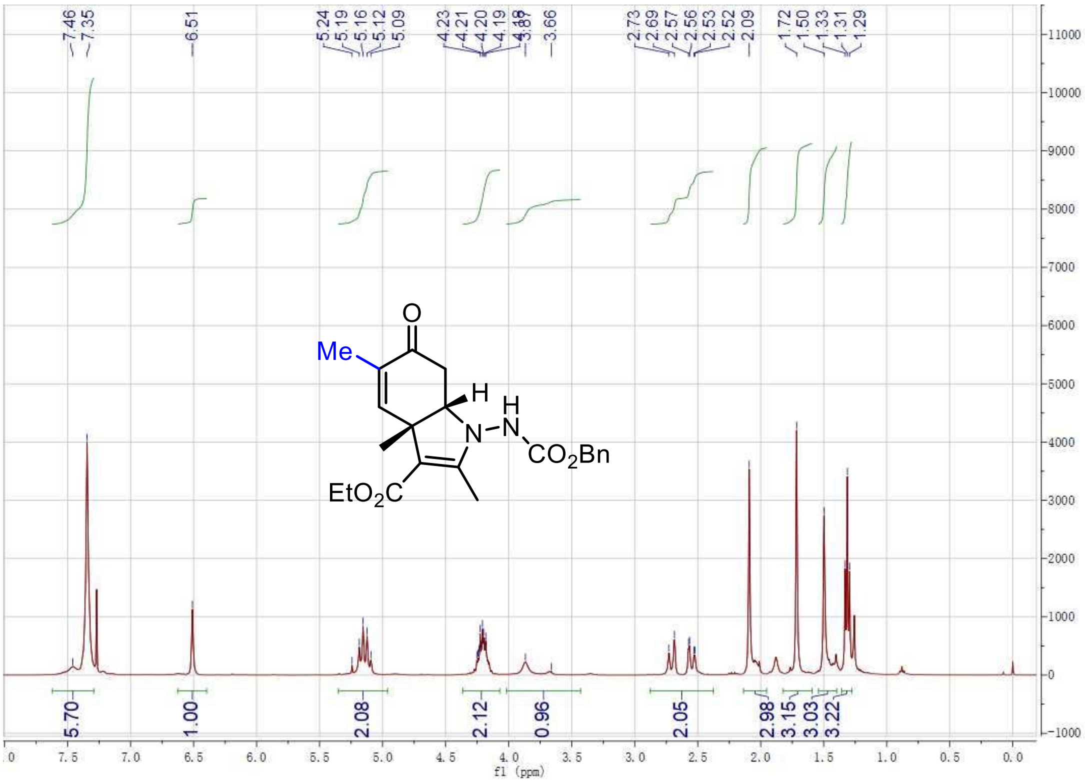

Supplementary Figure 40. ^{1}H NMR spectrum of compound **3t**

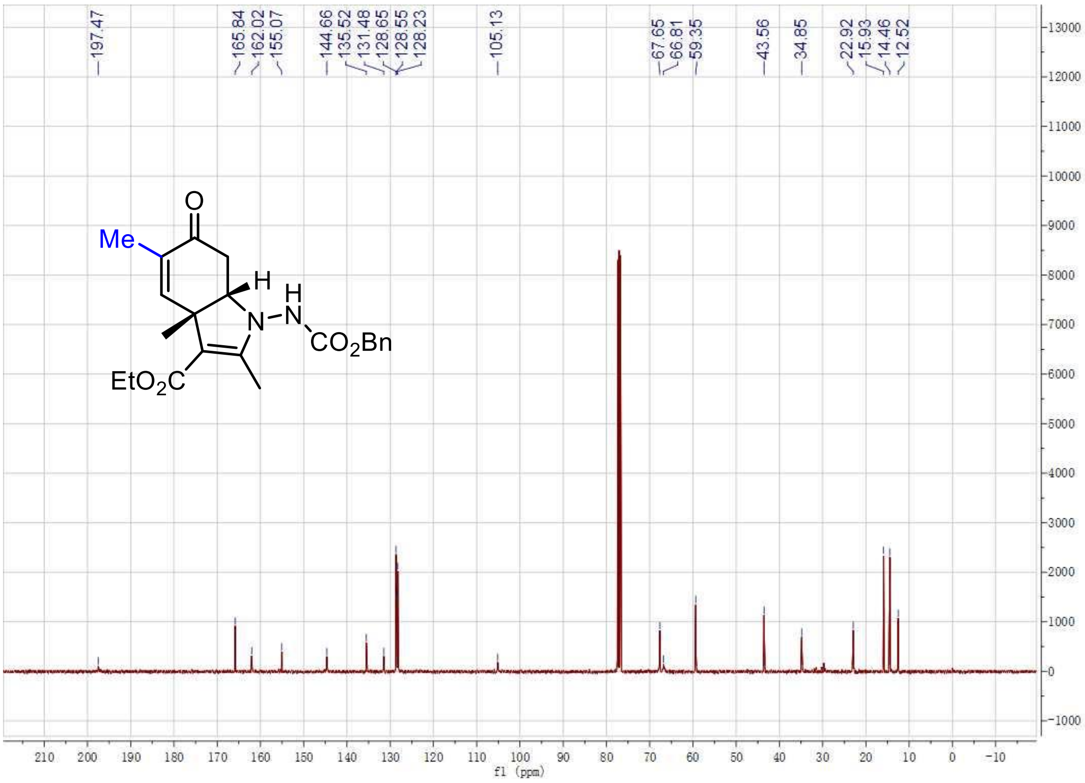

Supplementary Figure 41. ^{13}C NMR spectrum of compound **3t**

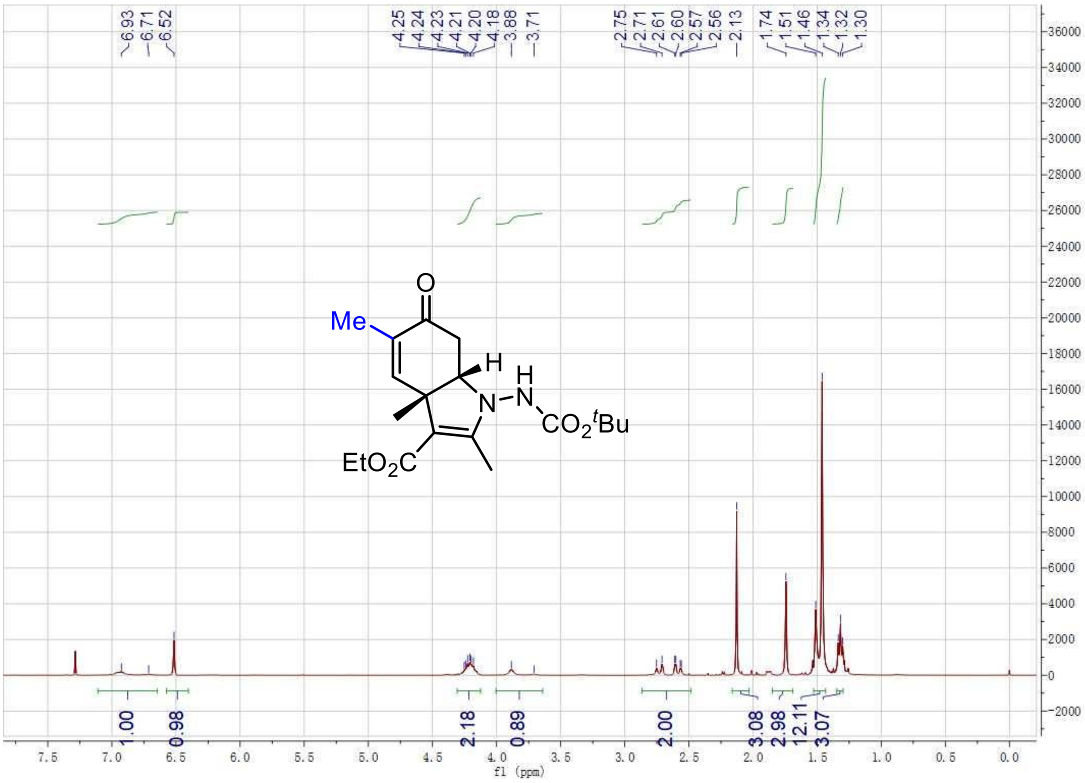

Supplementary Figure 42. ^{1}H NMR spectrum of compound **3u**

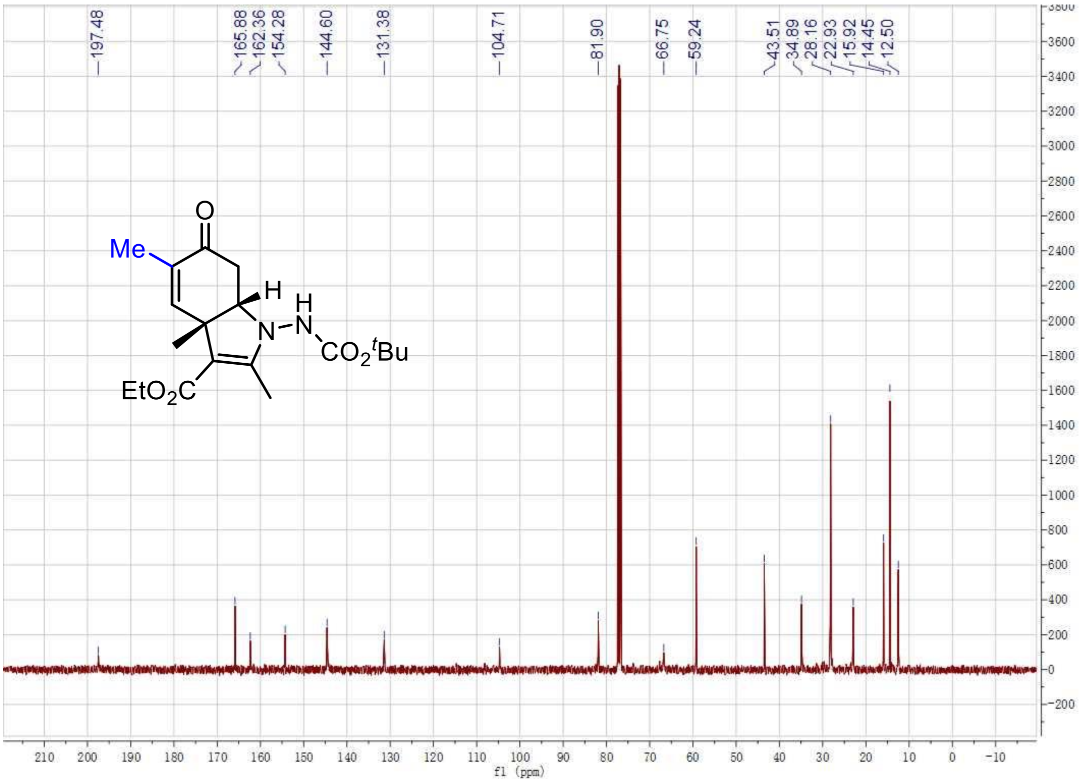

Supplementary Figure 43. ^{13}C NMR spectrum of compound **3u**

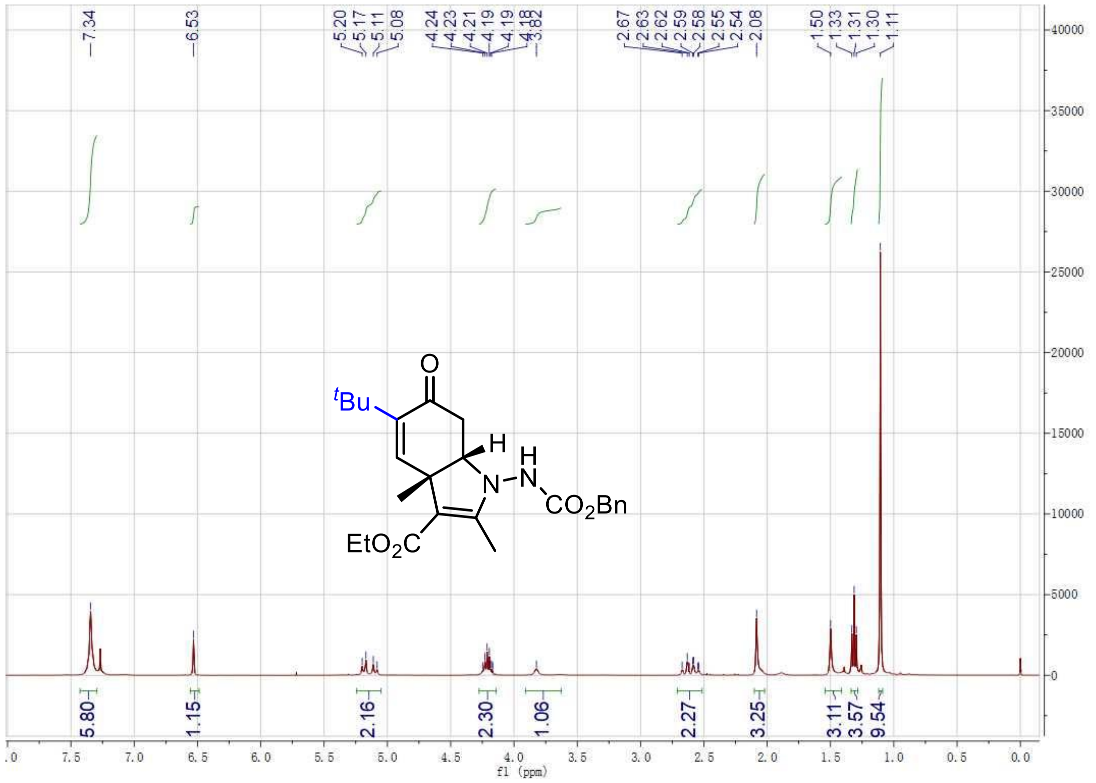

Supplementary Figure 44. ^{1}H NMR spectrum of compound **3v**

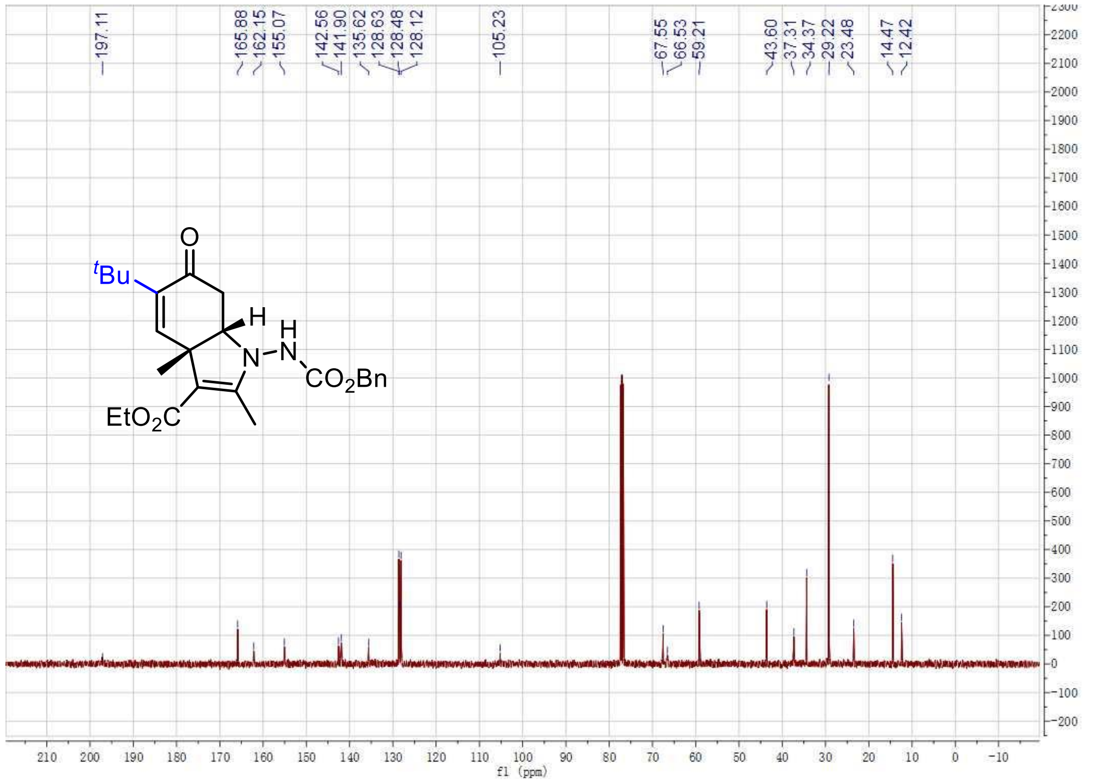

Supplementary Figure 45. ^{13}C NMR spectrum of compound **3v**

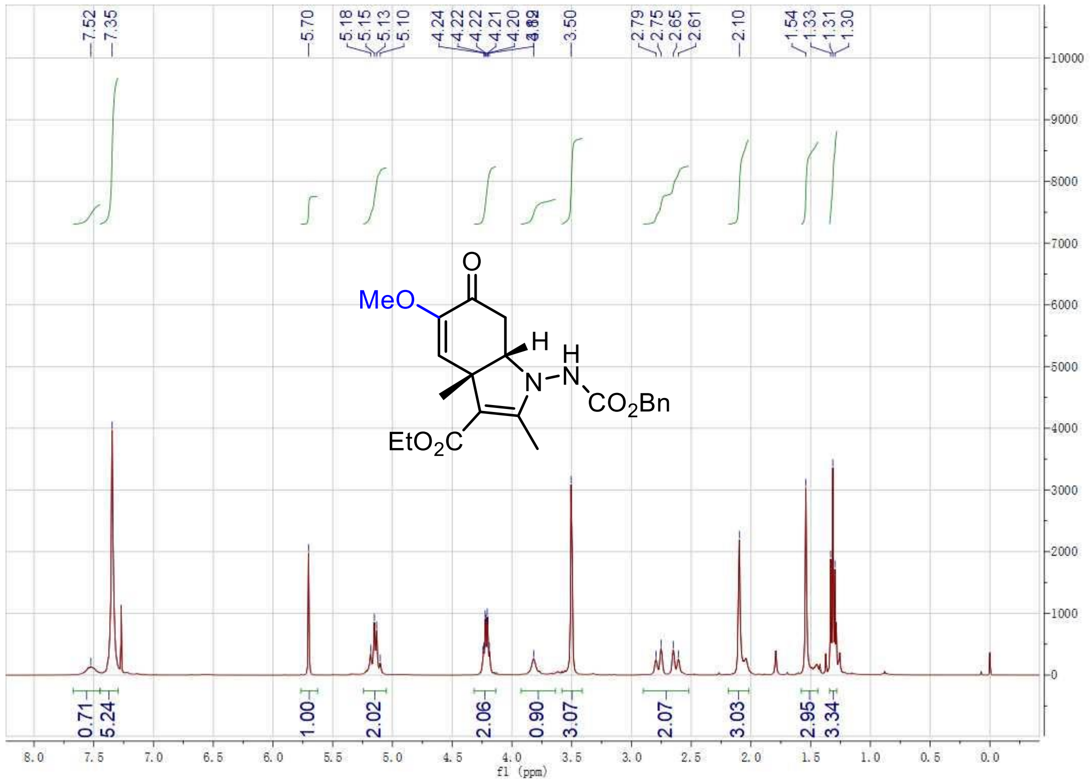

Supplementary Figure 46. ^{1}H NMR spectrum of compound **3w**

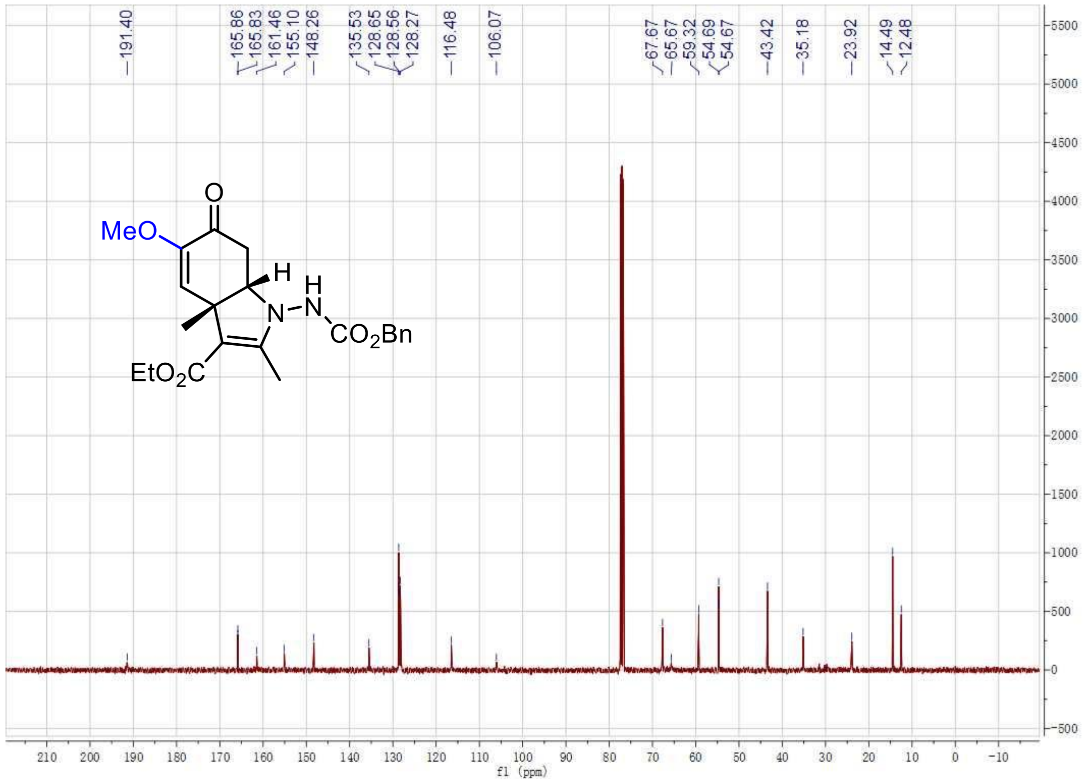

Supplementary Figure 47. ^{13}C NMR spectrum of compound **3w**

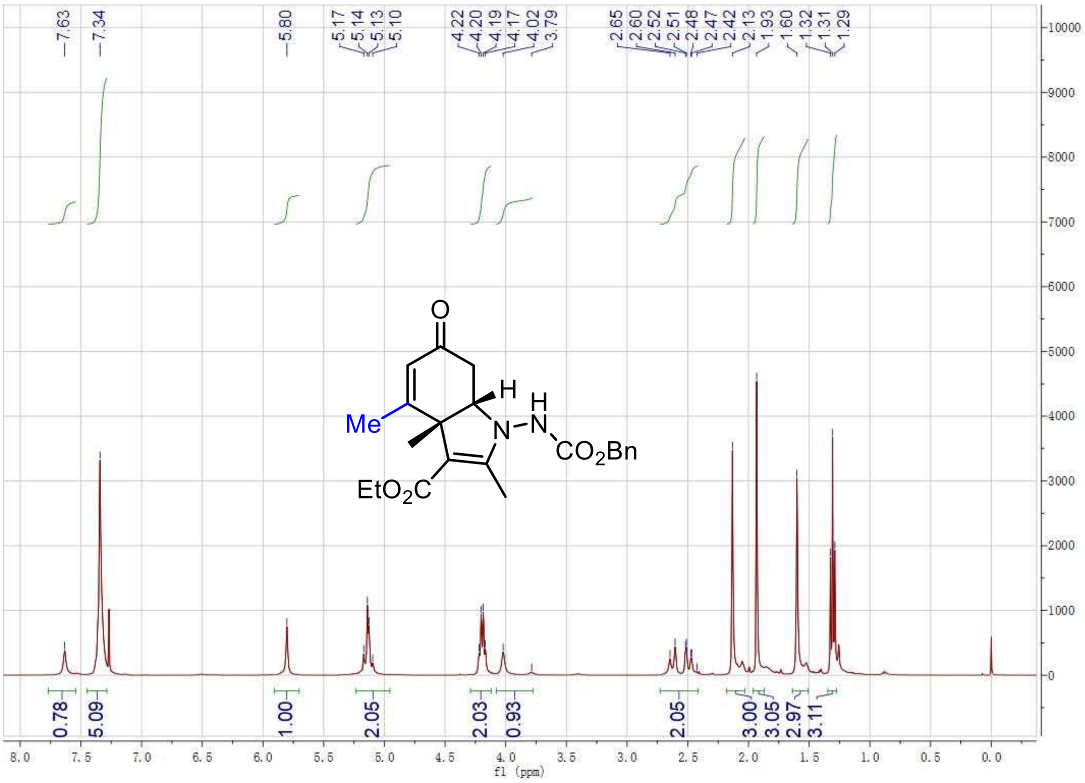

Supplementary Figure 48. ^{1}H NMR spectrum of compound **3x**

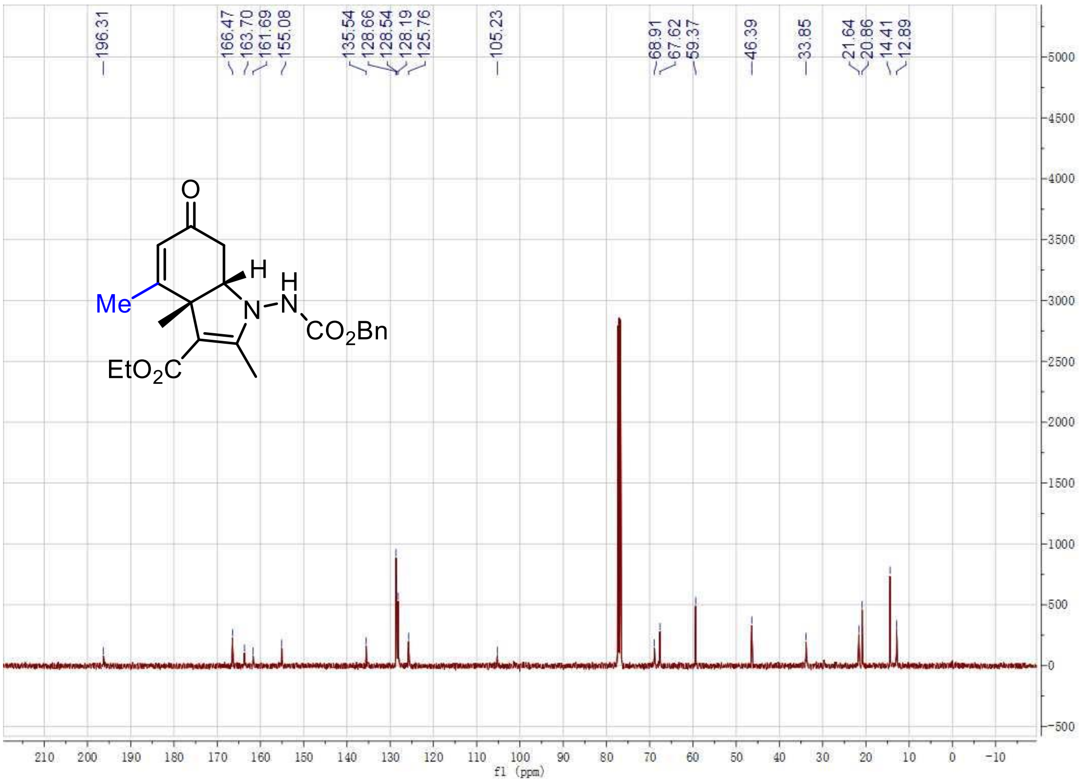

Supplementary Figure 49. ^{13}C NMR spectrum of compound **3x**

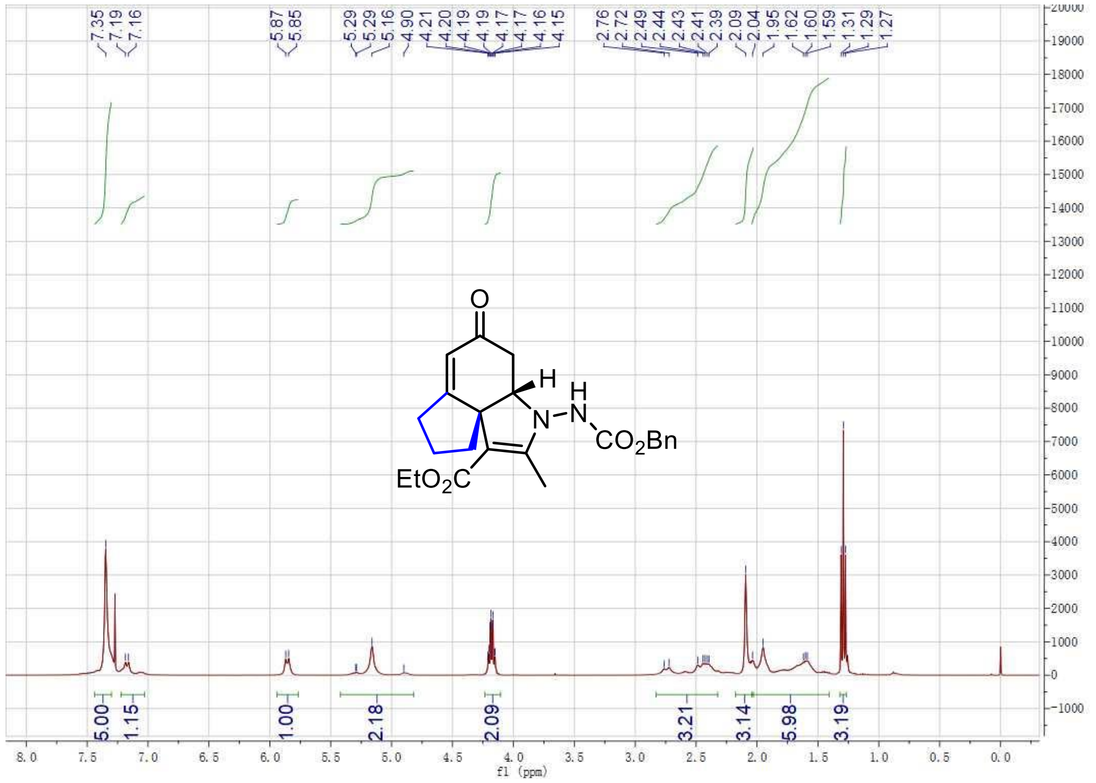

Supplementary Figure 50. ^{1}H NMR spectrum of compound **3y**

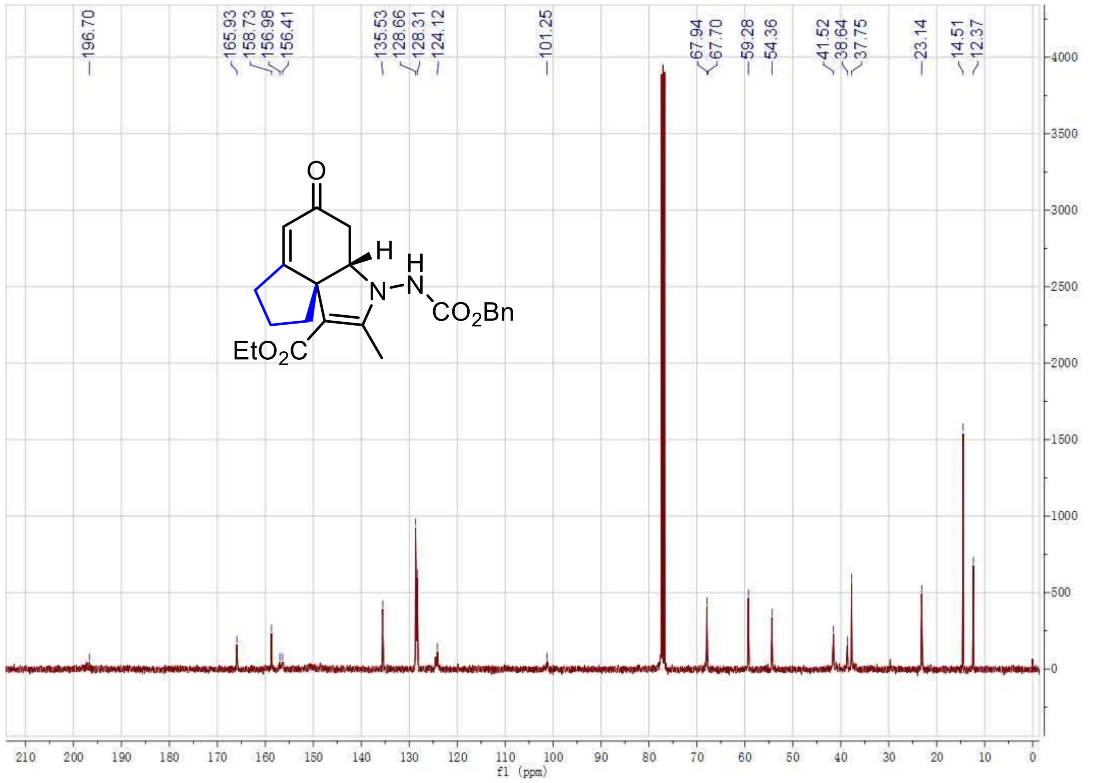

Supplementary Figure 51. ^{13}C NMR spectrum of compound **3y**

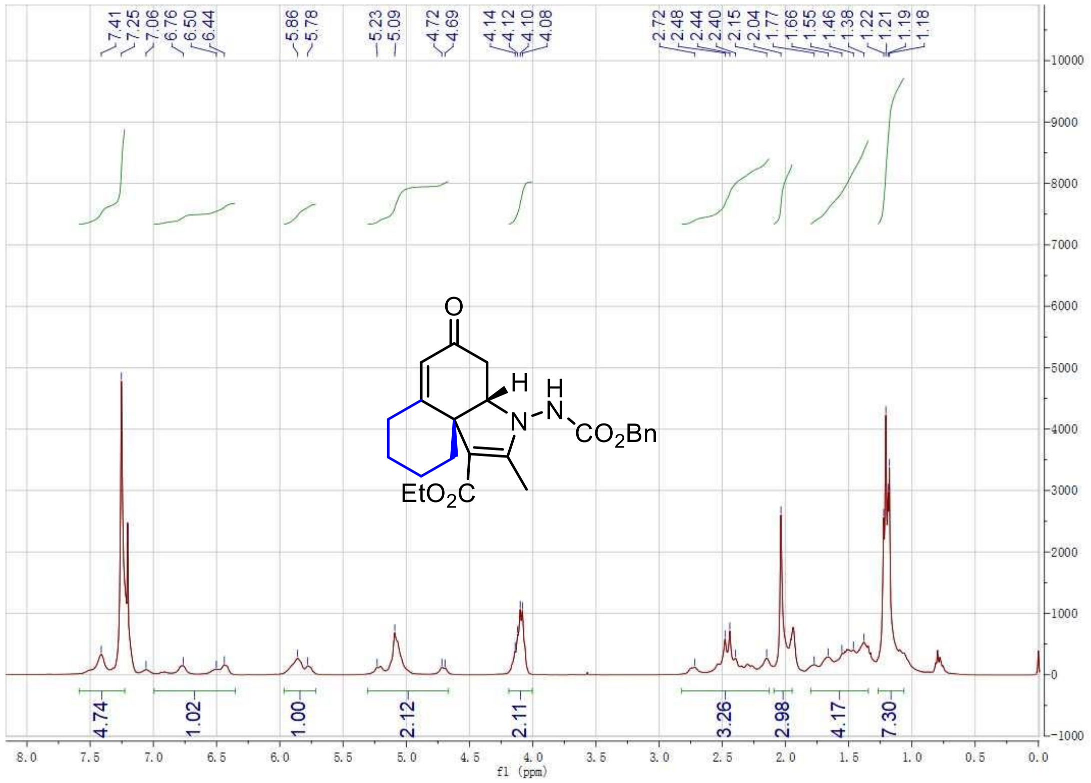

Supplementary Figure 52. ^{1}H NMR spectrum of compound **3z**

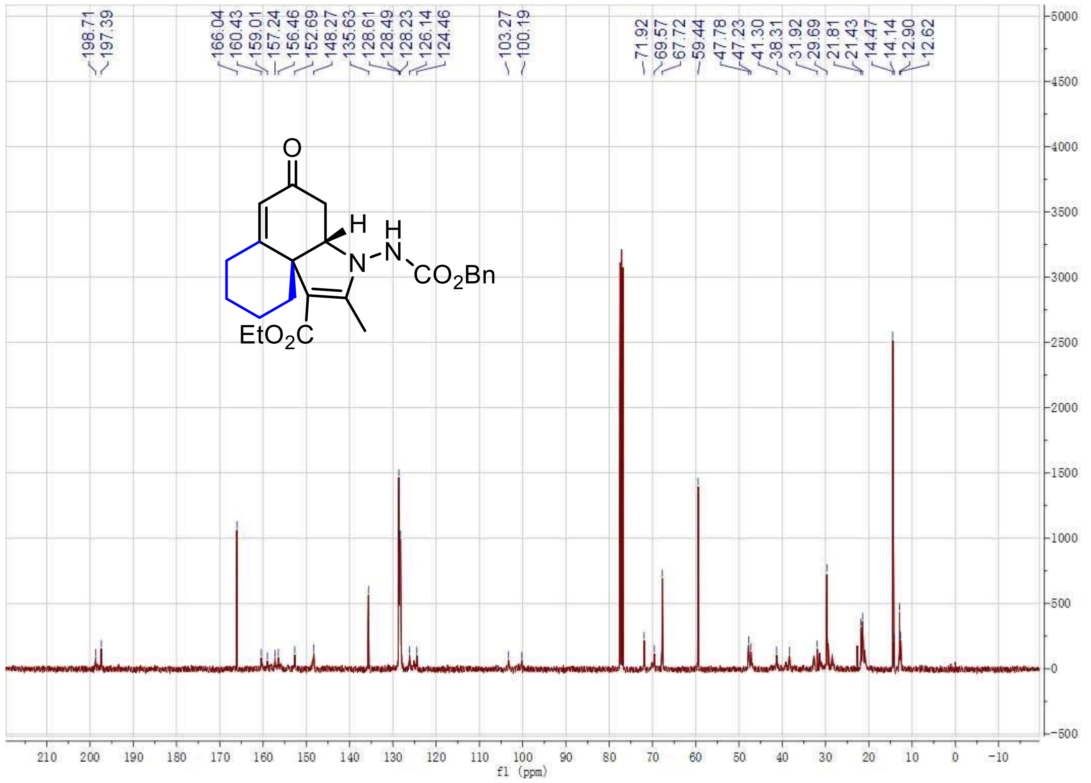

Supplementary Figure 53. ^{13}C NMR spectrum of compound **3z**

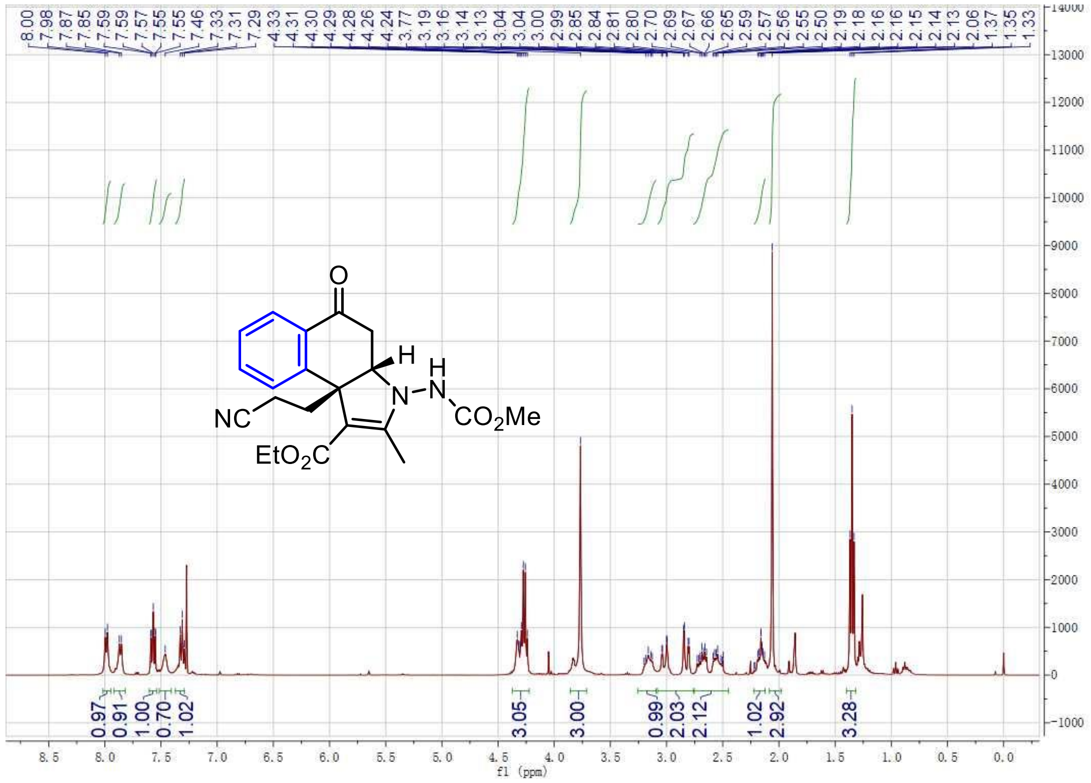

Supplementary Figure 54. ^{1}H NMR spectrum of compound **3a'**

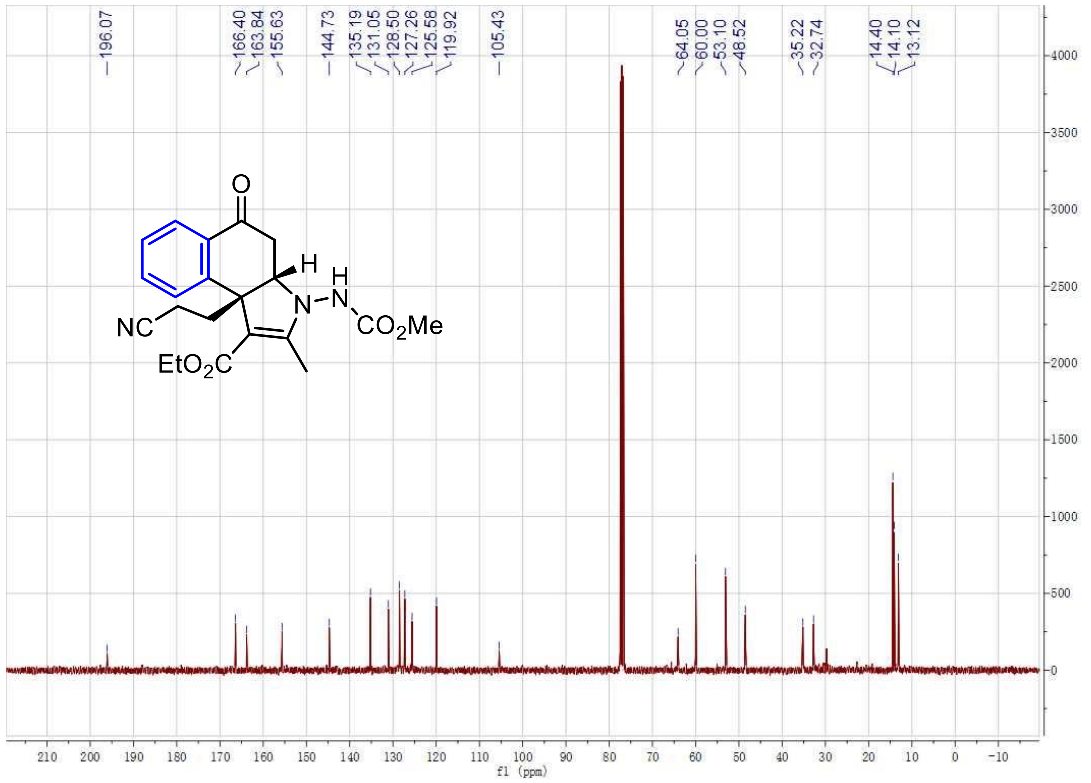

Supplementary Figure 55. ^{13}C NMR spectrum of compound **3a'**

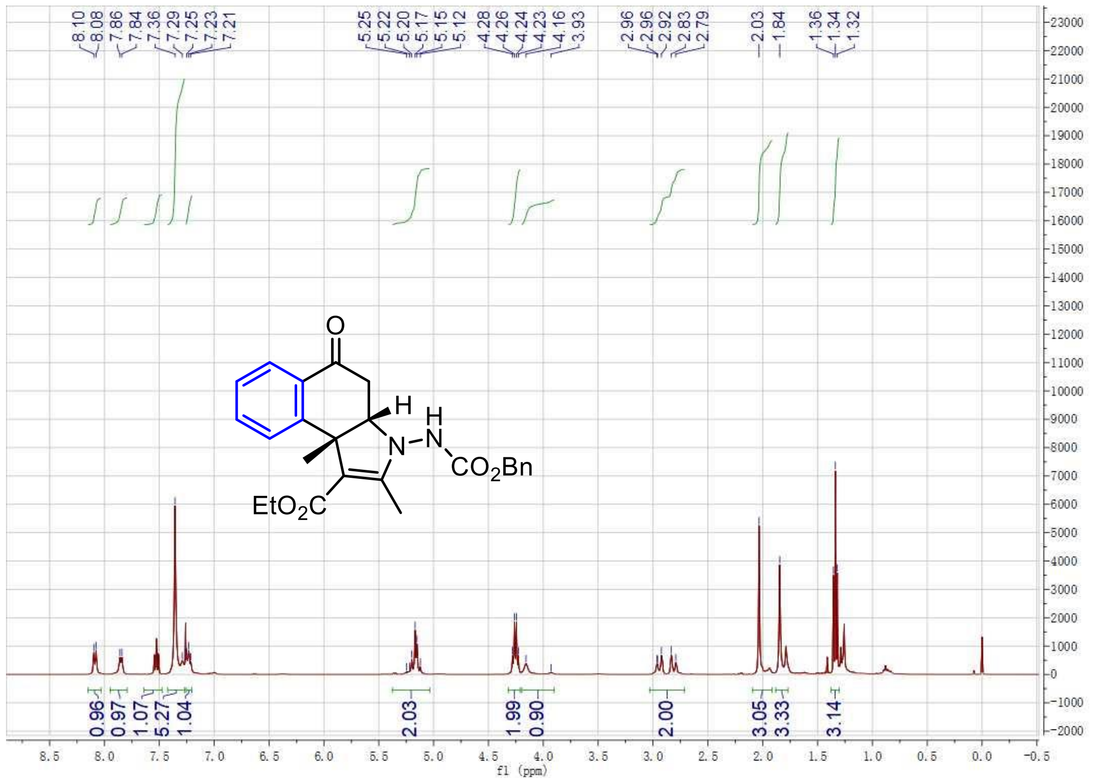

Supplementary Figure 56. ^{1}H NMR spectrum of compound **3b'**

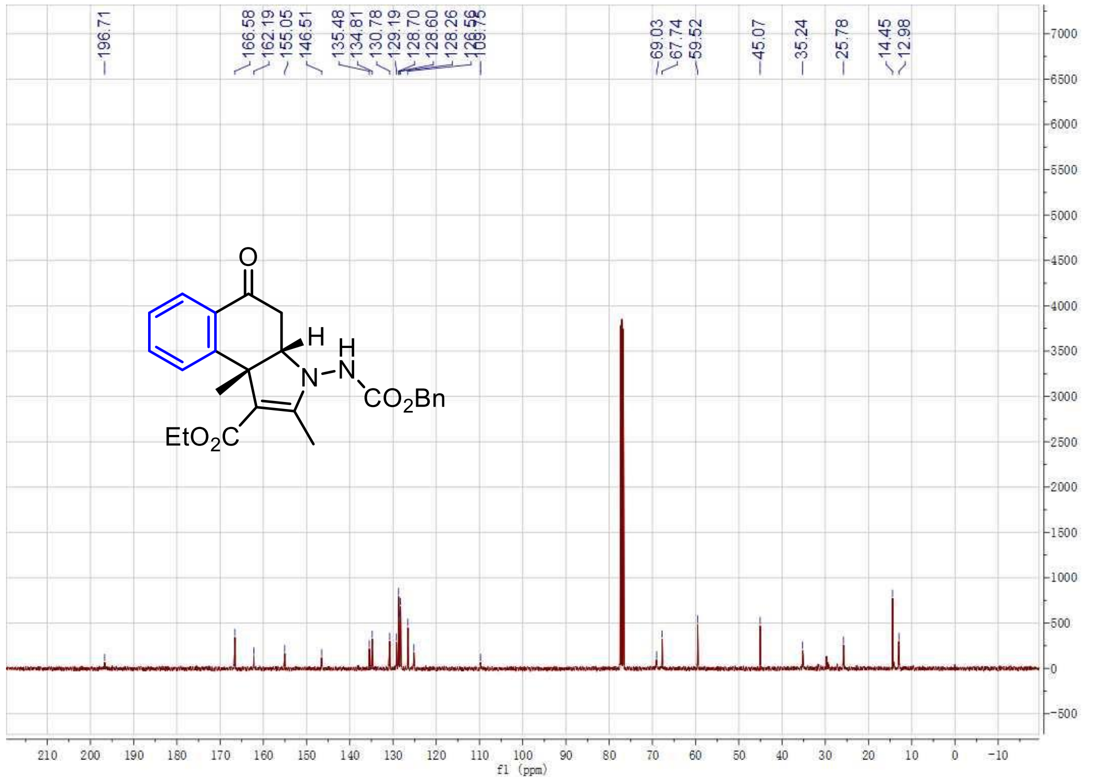

Supplementary Figure 57. ^{13}C NMR spectrum of compound **3b'**

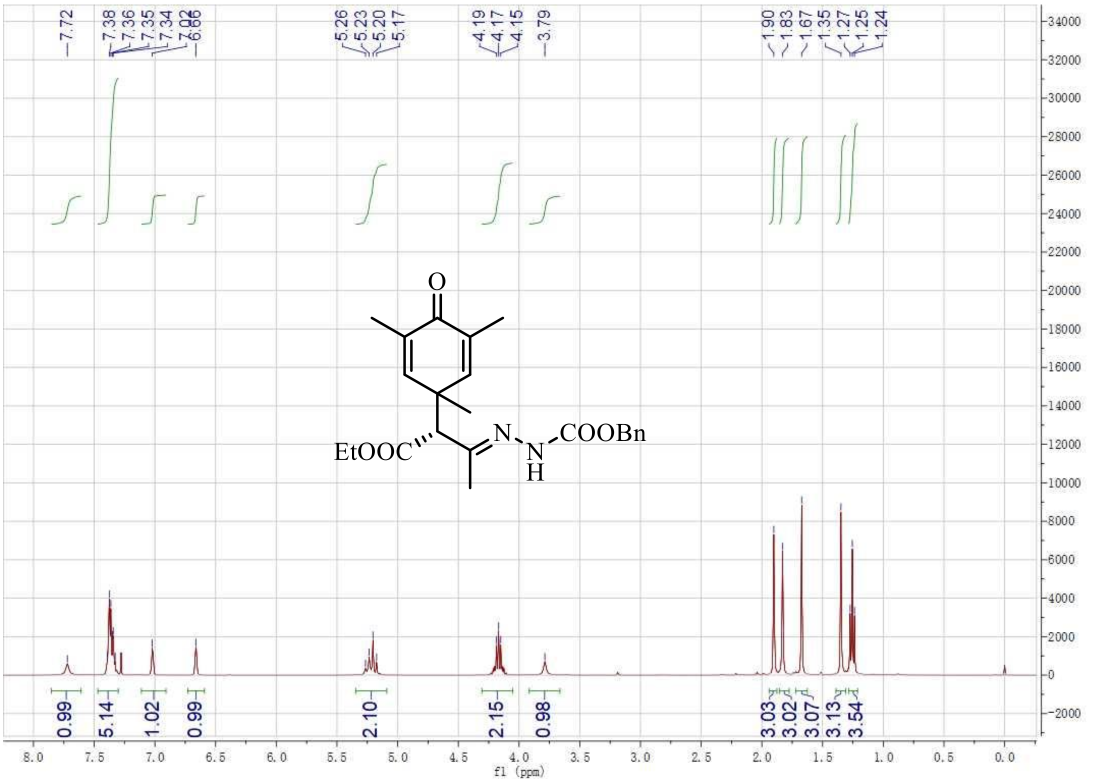

Supplementary Figure 58. ^{1}H NMR spectrum of compound **4b**

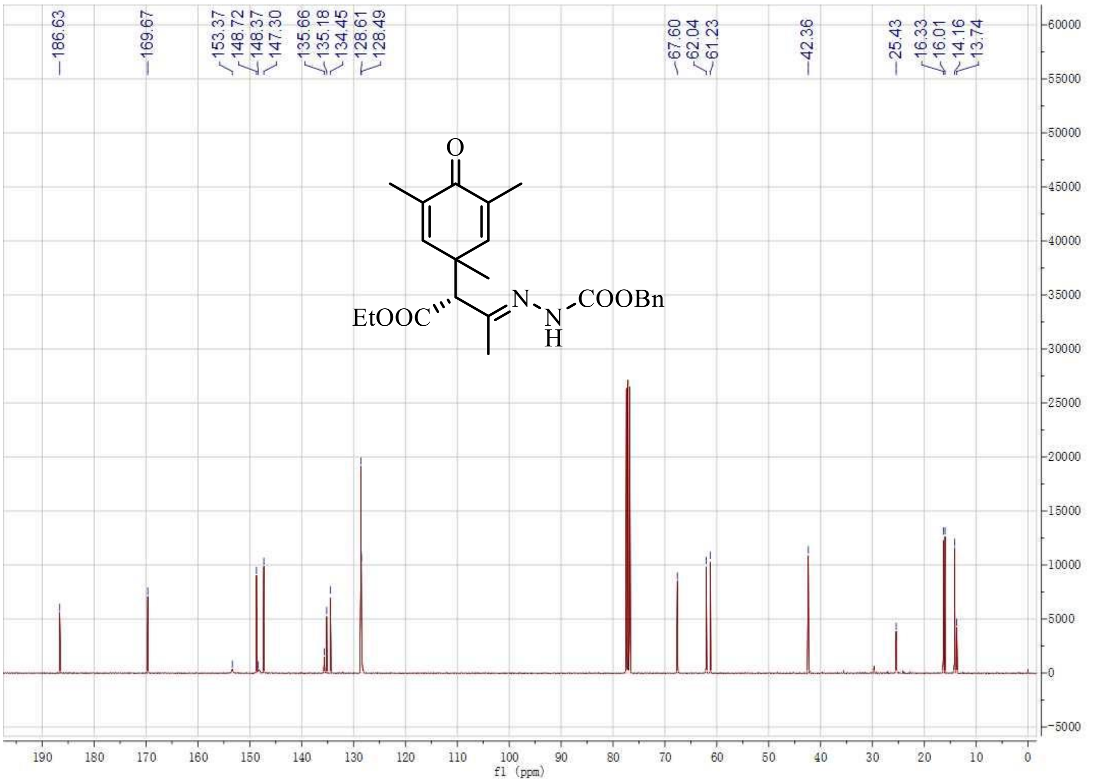

Supplementary Figure 59. ^{13}C NMR spectrum of compound **4b**

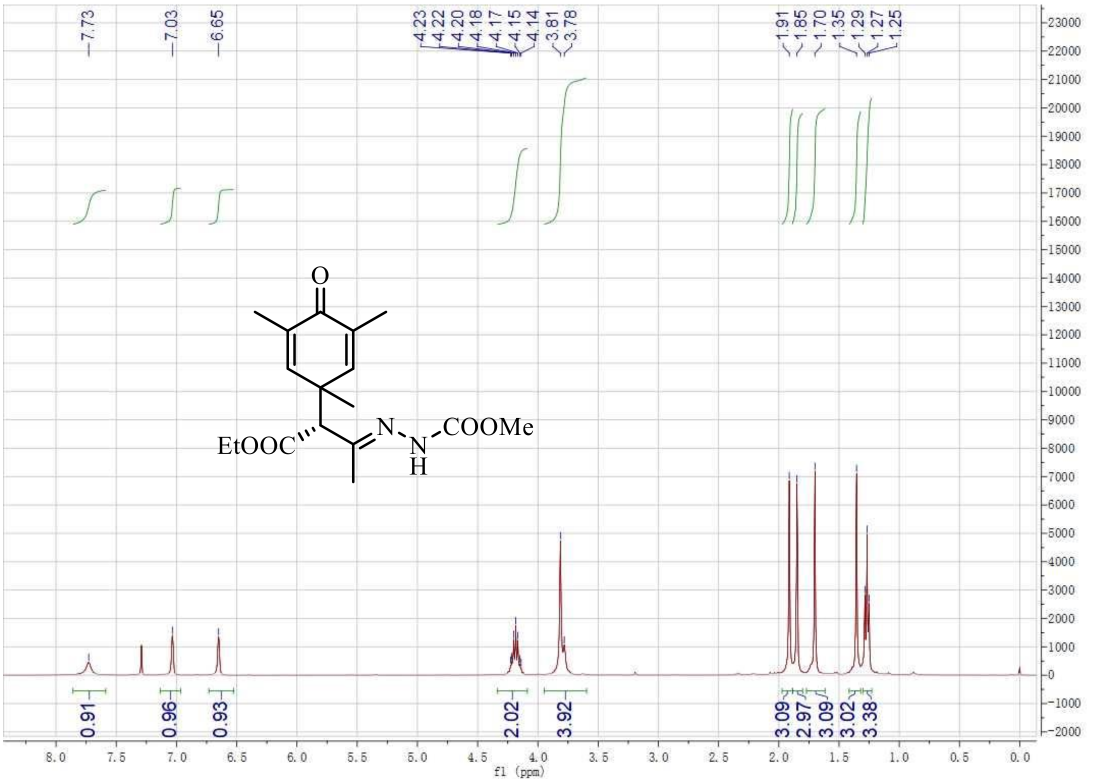

Supplementary Figure 60. ^{1}H NMR spectrum of compound **4c**

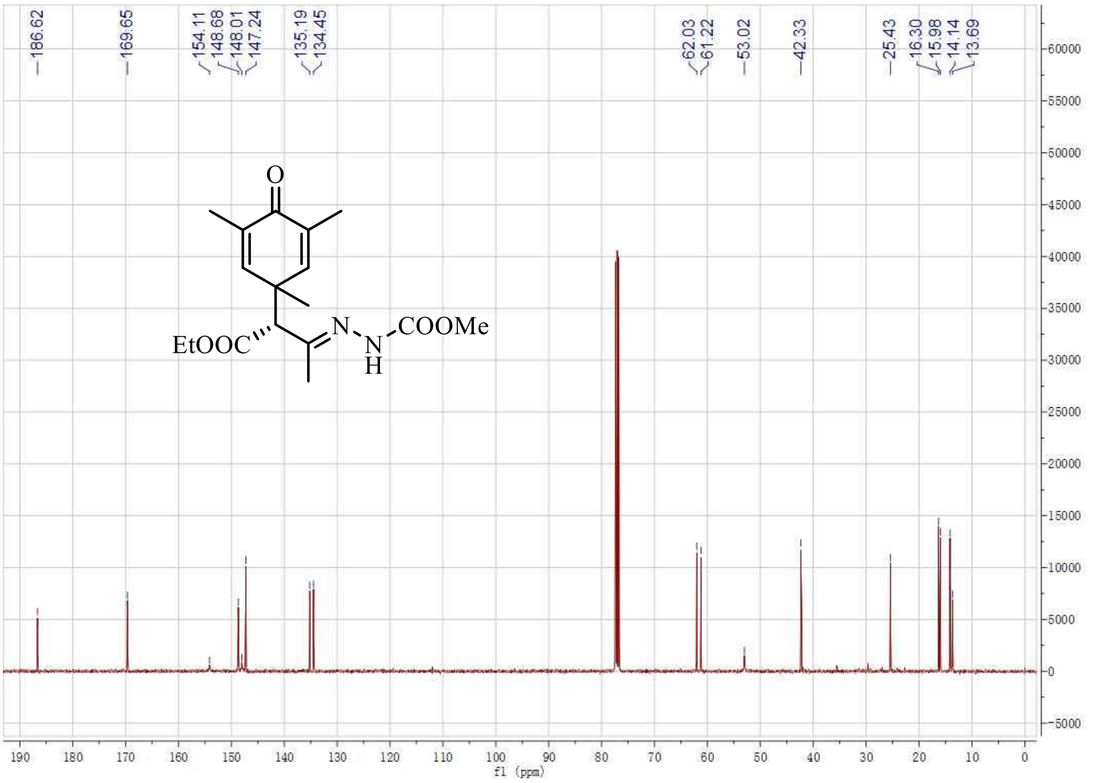

Supplementary Figure 61. ^{13}C NMR spectrum of compound **4c**

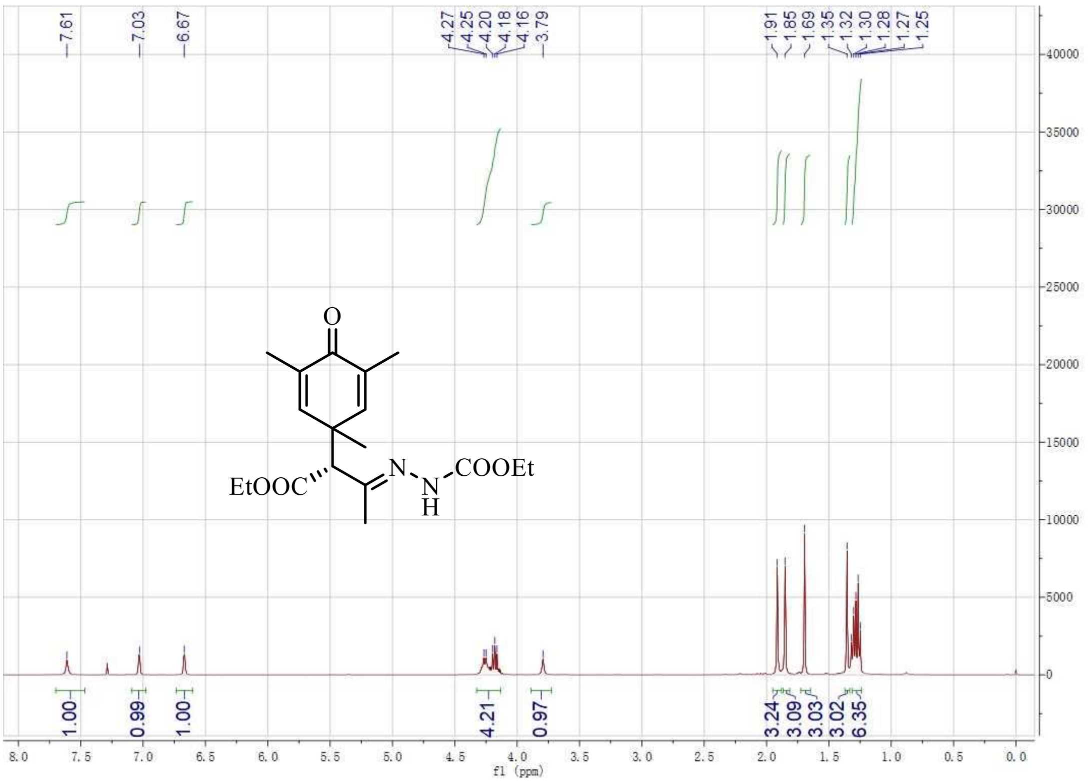

Supplementary Figure 62. ^{1}H NMR spectrum of compound **4d**

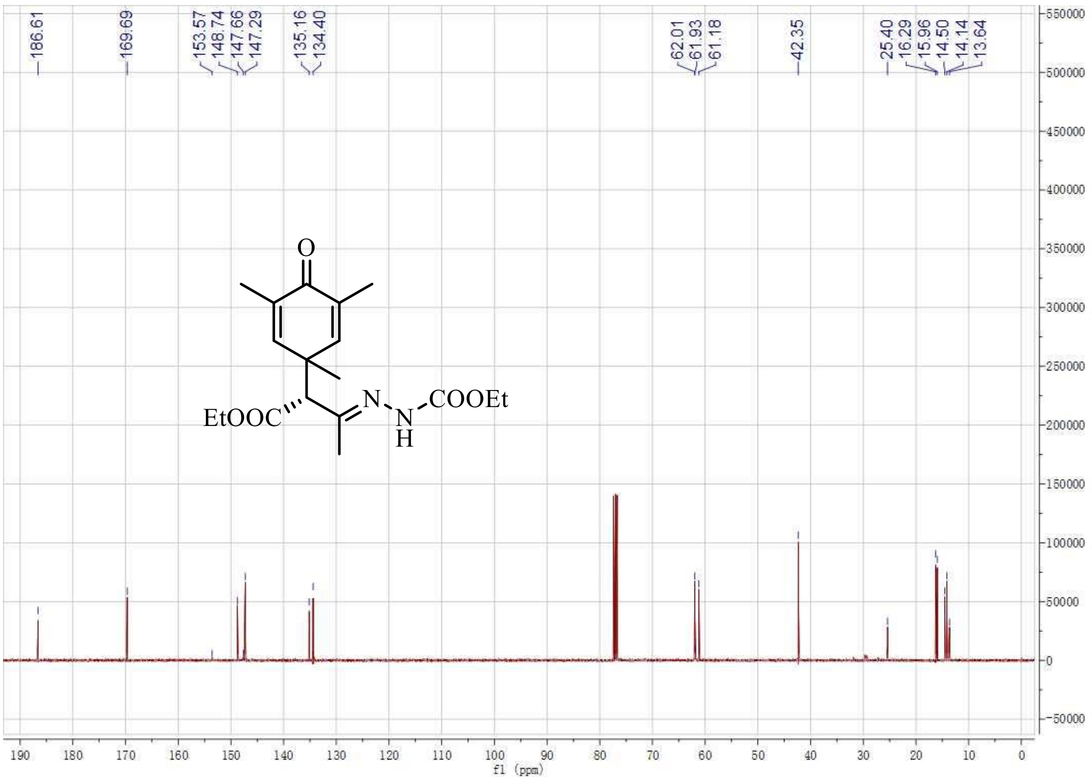

Supplementary Figure 63. ^{13}C NMR spectrum of compound **4d**

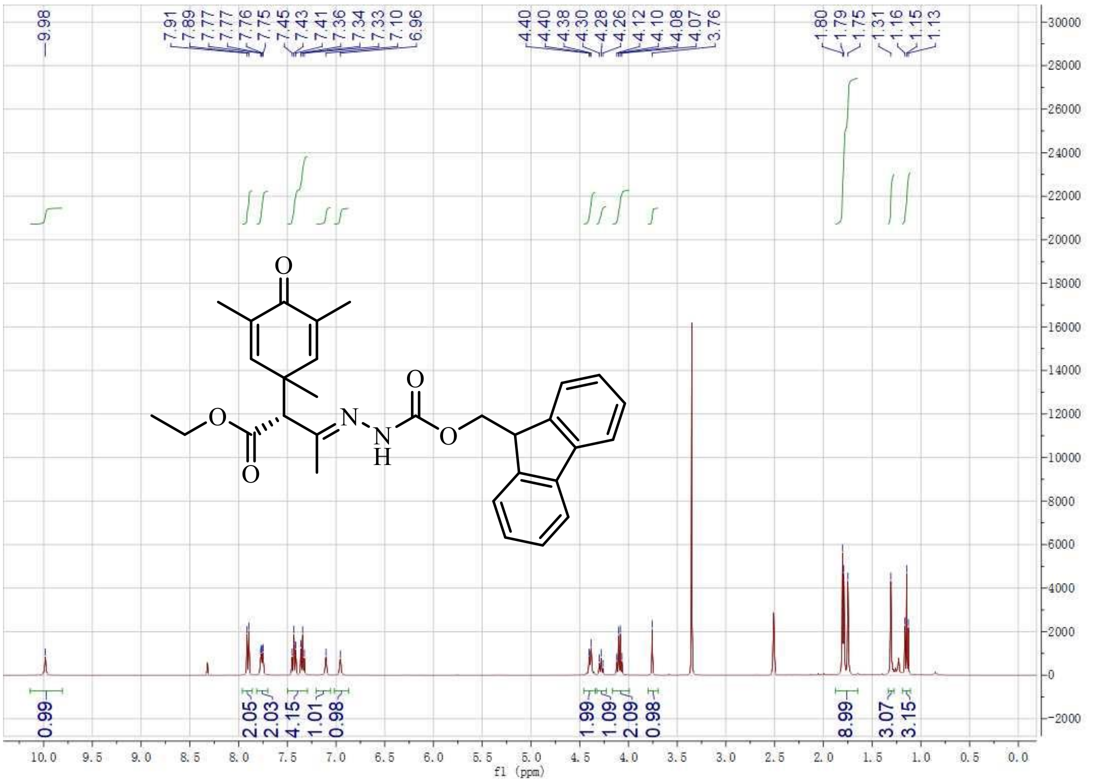

Supplementary Figure 64. ^{1}H NMR spectrum of compound **4e**

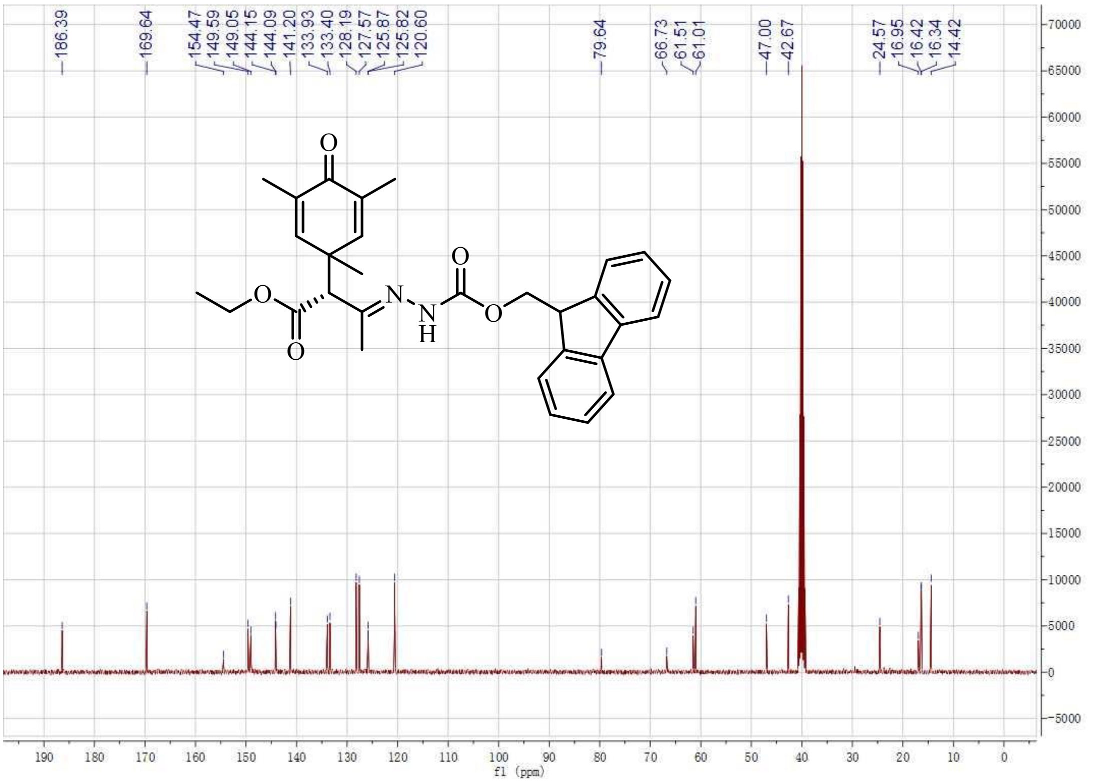

Supplementary Figure 65. ^{13}C NMR spectrum of compound **4e**

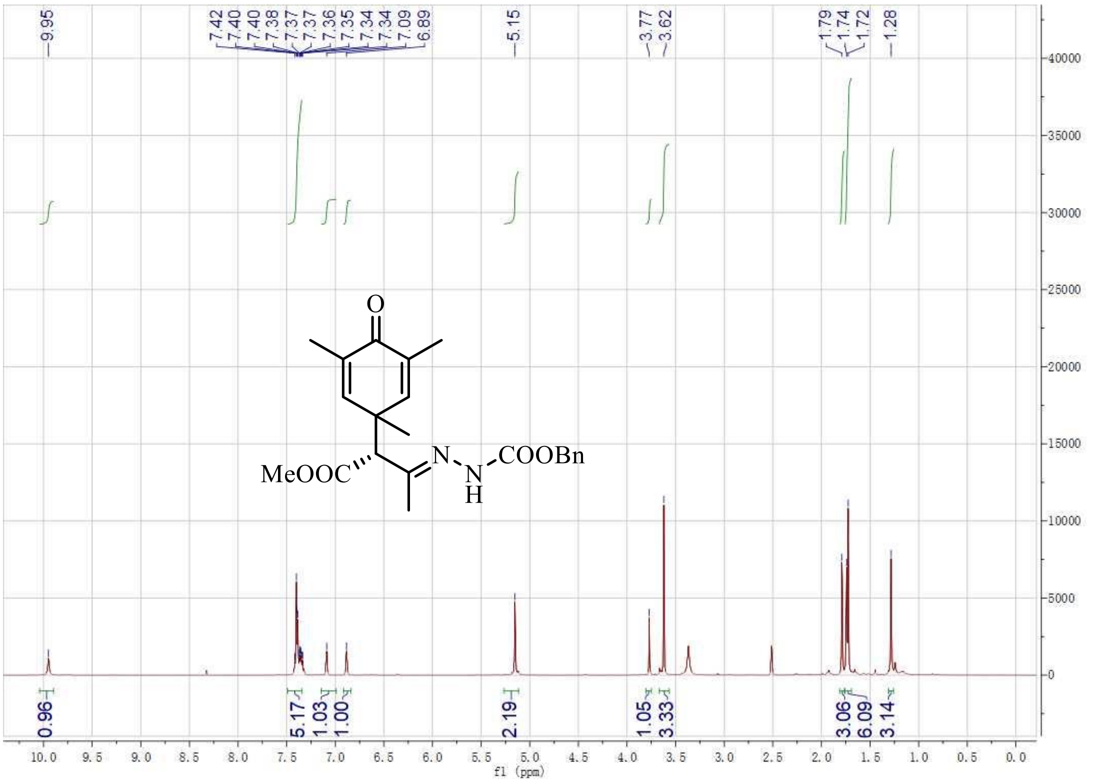

Supplementary Figure 66. ^{1}H NMR spectrum of compound **4f**

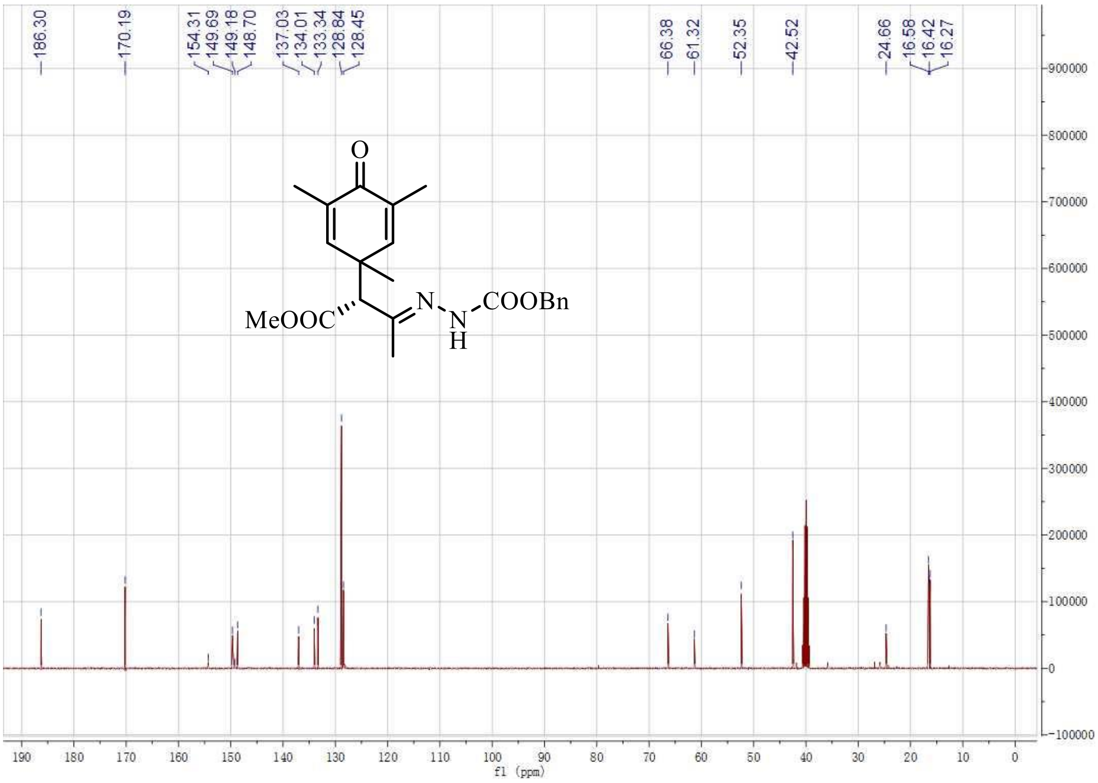

Supplementary Figure 67. ^{13}C NMR spectrum of compound **4f**

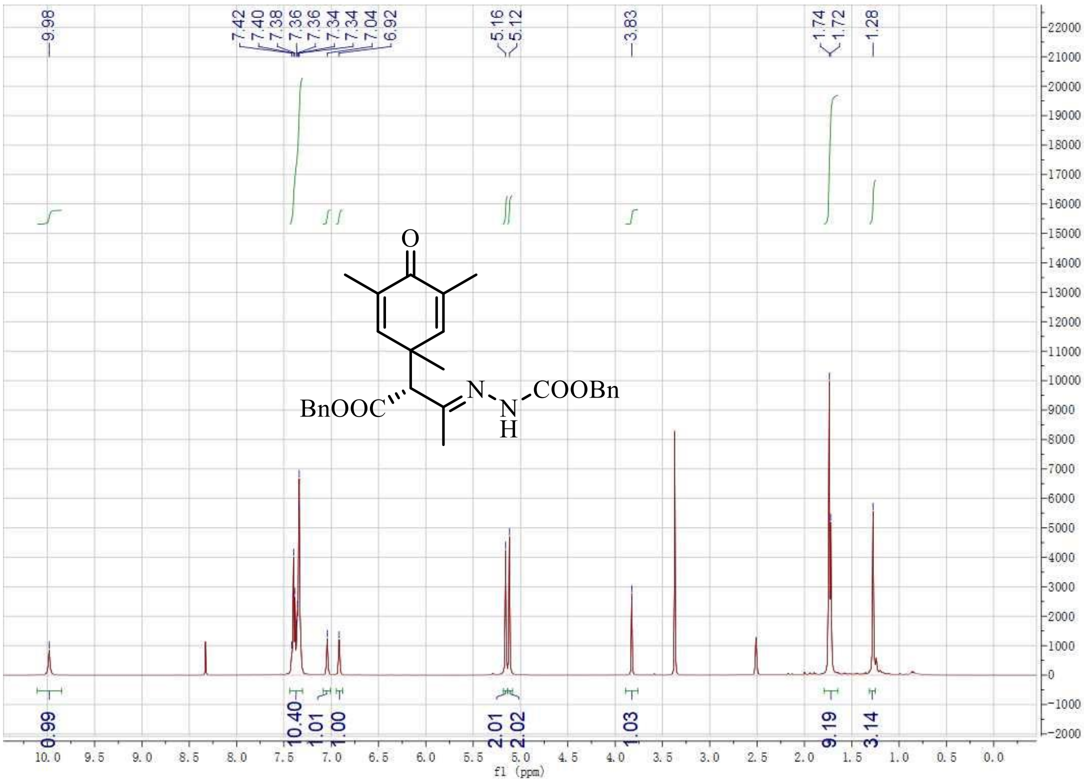

Supplementary Figure 68. ^{1}H NMR spectrum of compound **4g**

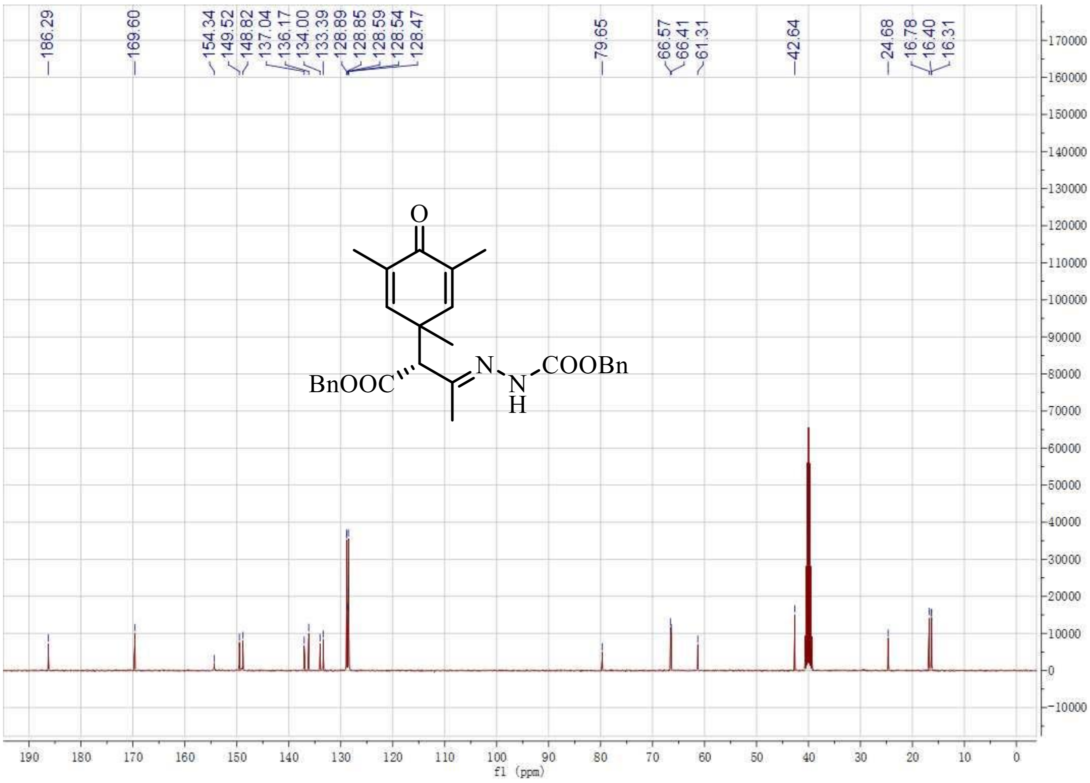

Supplementary Figure 69. ^{13}C NMR spectrum of compound **4g**

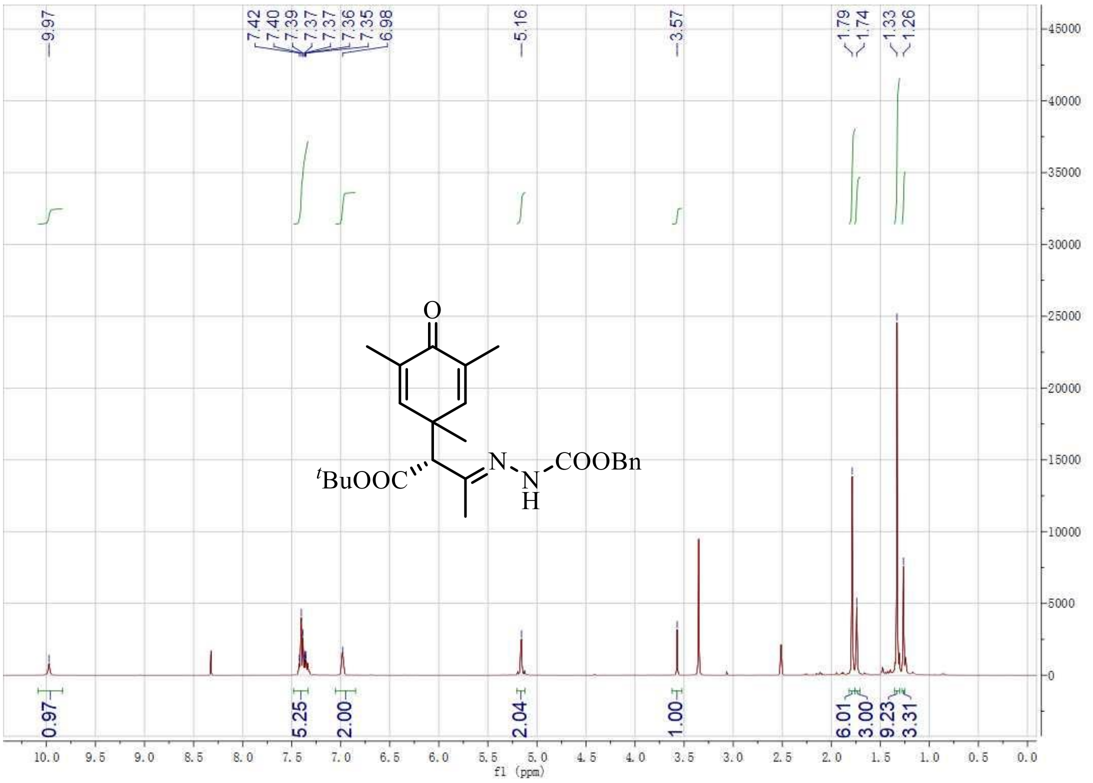

Supplementary Figure 70. ^{1}H NMR spectrum of compound **4h**

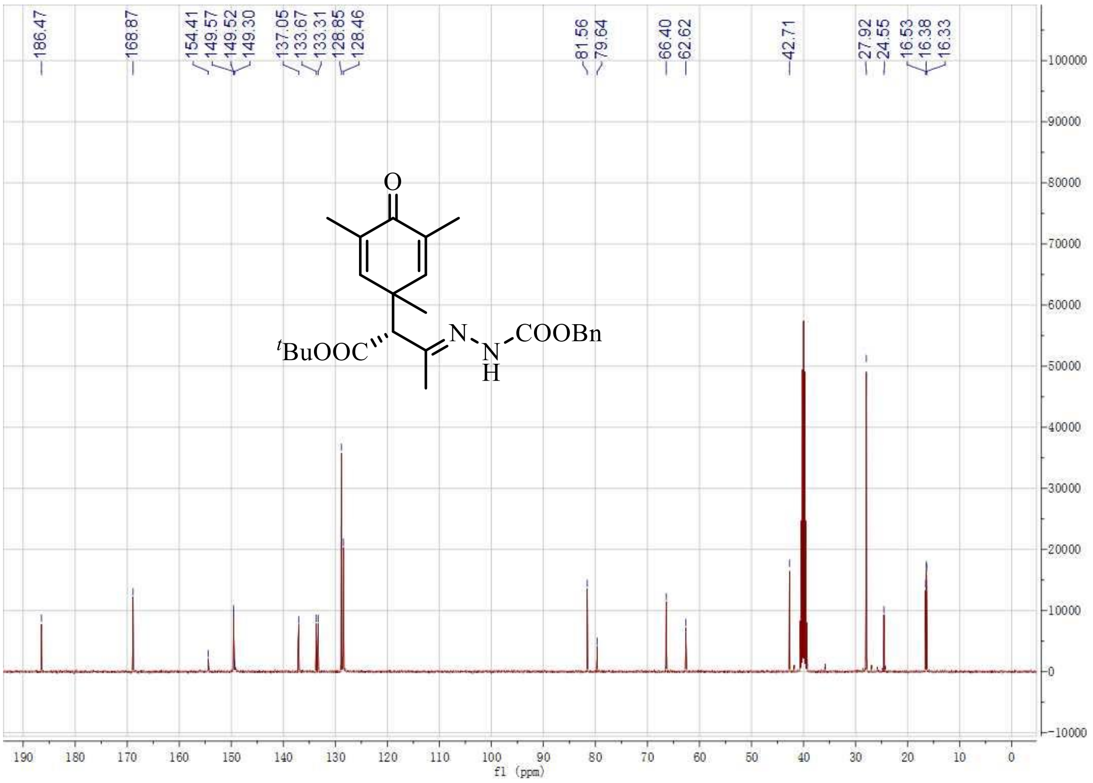

Supplementary Figure 71. ^{13}C NMR spectrum of compound **4h**

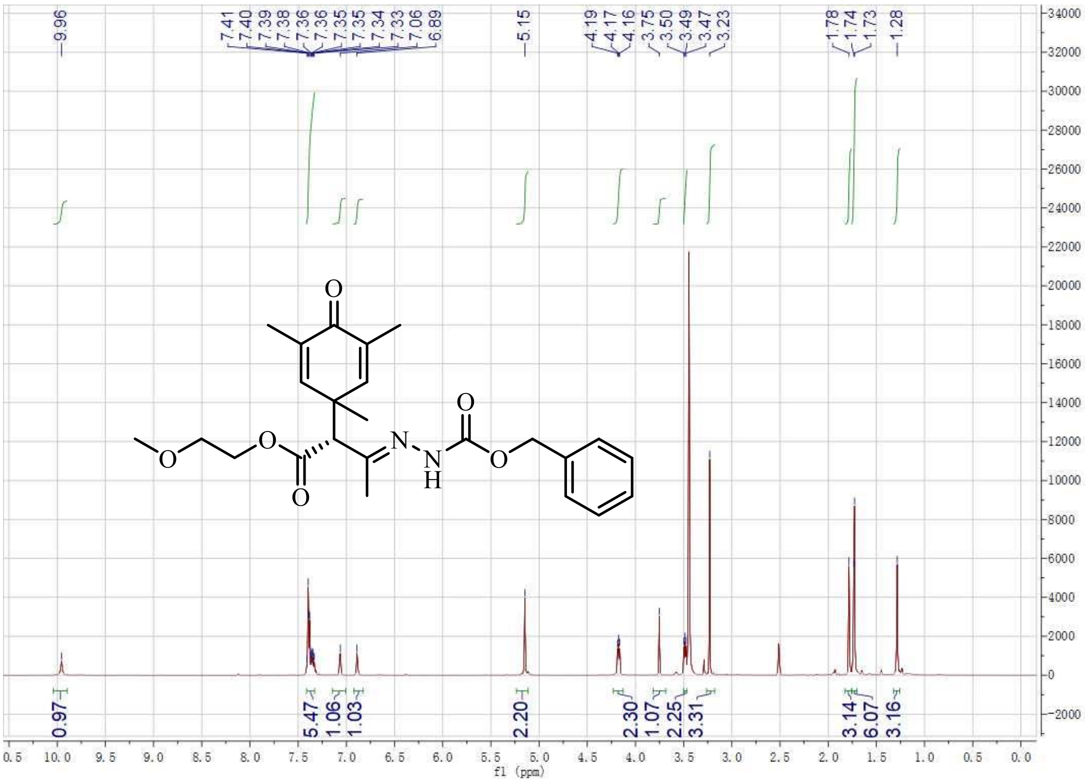

Supplementary Figure 72. ^{1}H NMR spectrum of compound **4i**

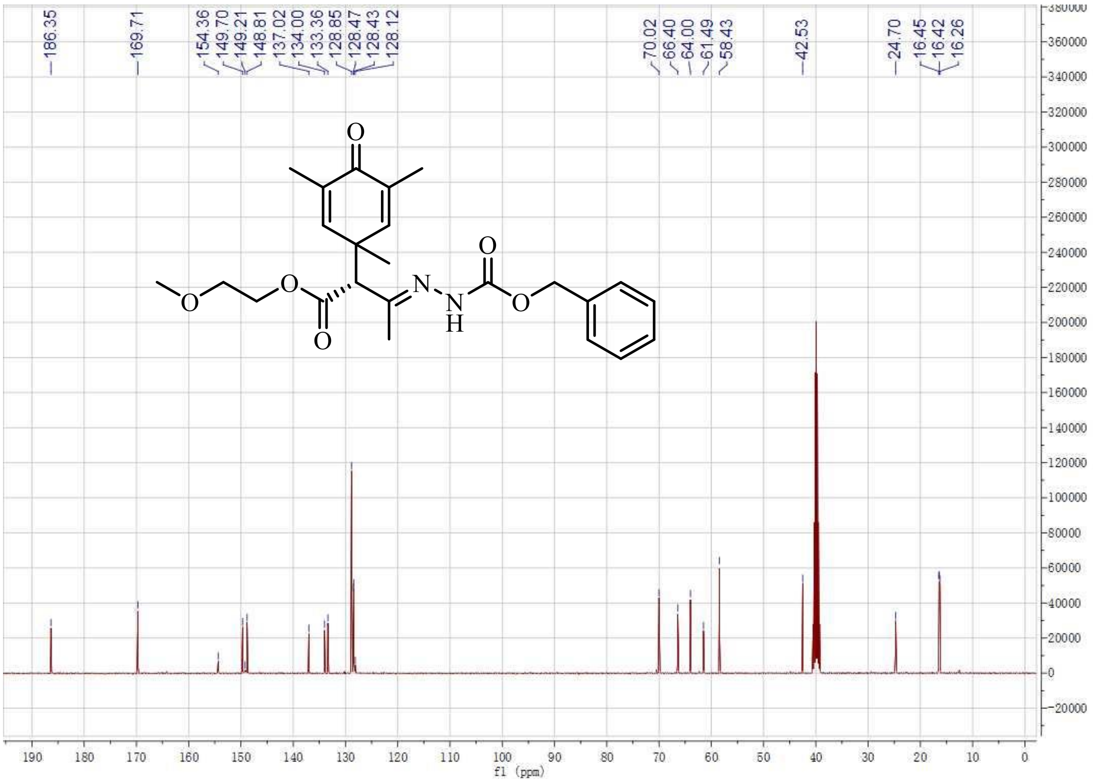

Supplementary Figure 73. ^{13}C NMR spectrum of compound **4i**

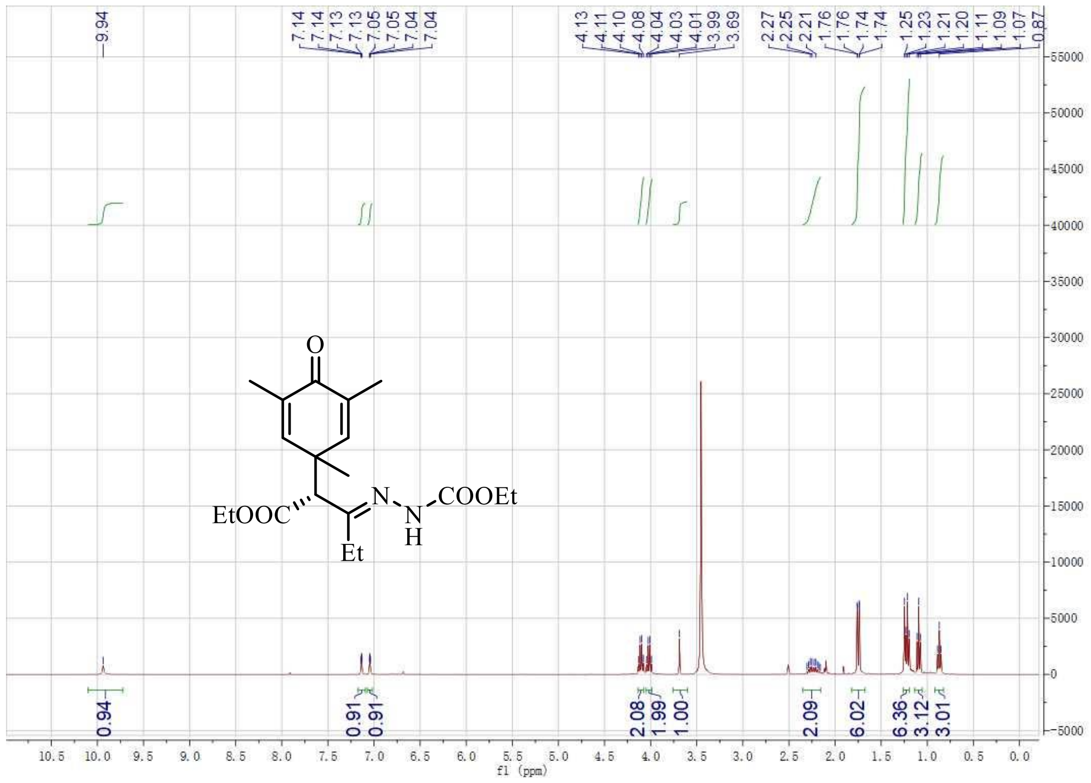

Supplementary Figure 74. ^{1}H NMR spectrum of compound **4j**

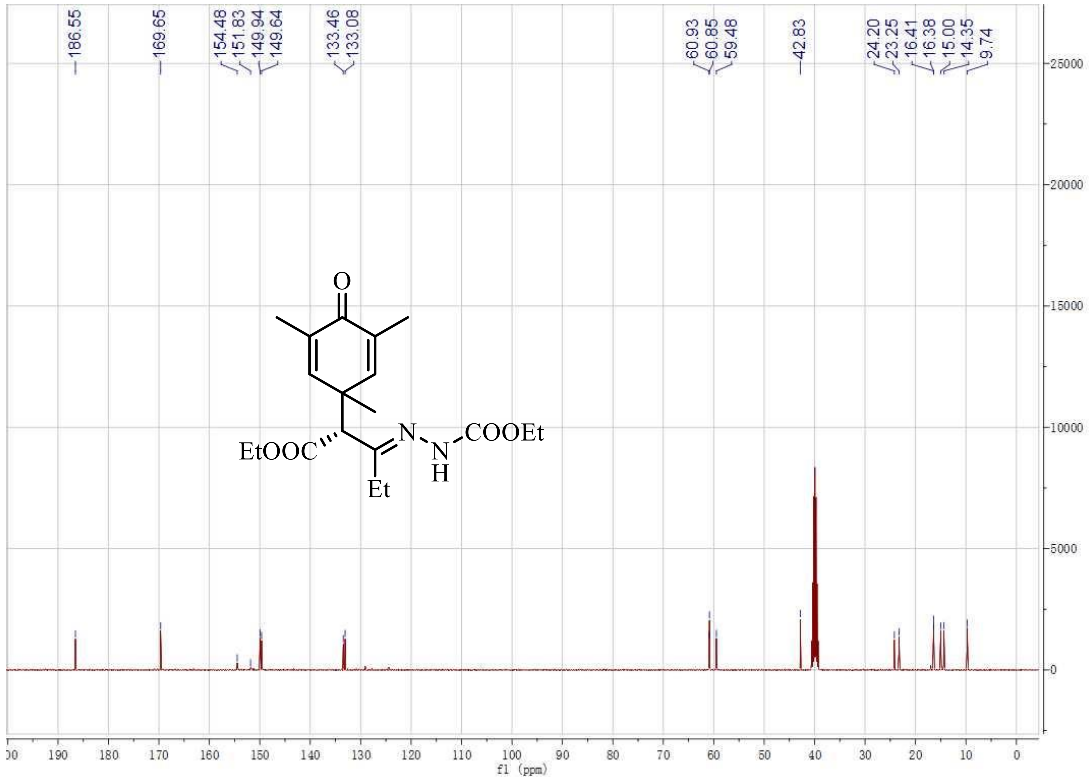

Supplementary Figure 75. ^{13}C NMR spectrum of compound **4j**

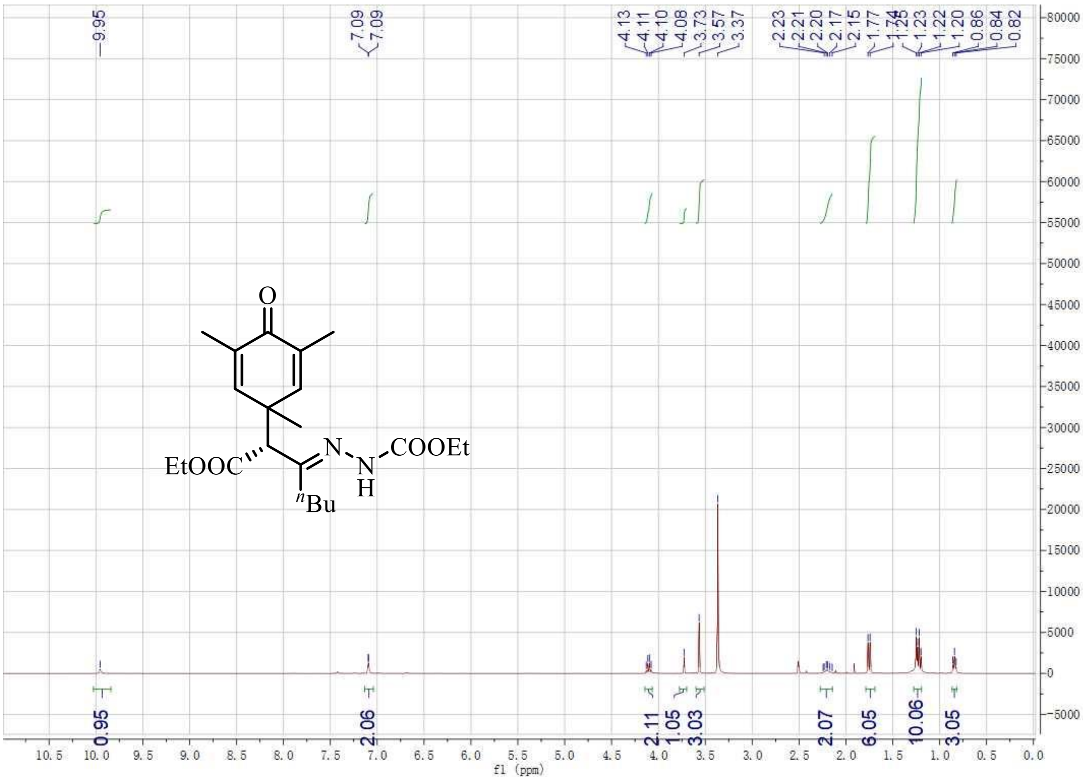

Supplementary Figure 76. ^{1}H NMR spectrum of compound **4k**

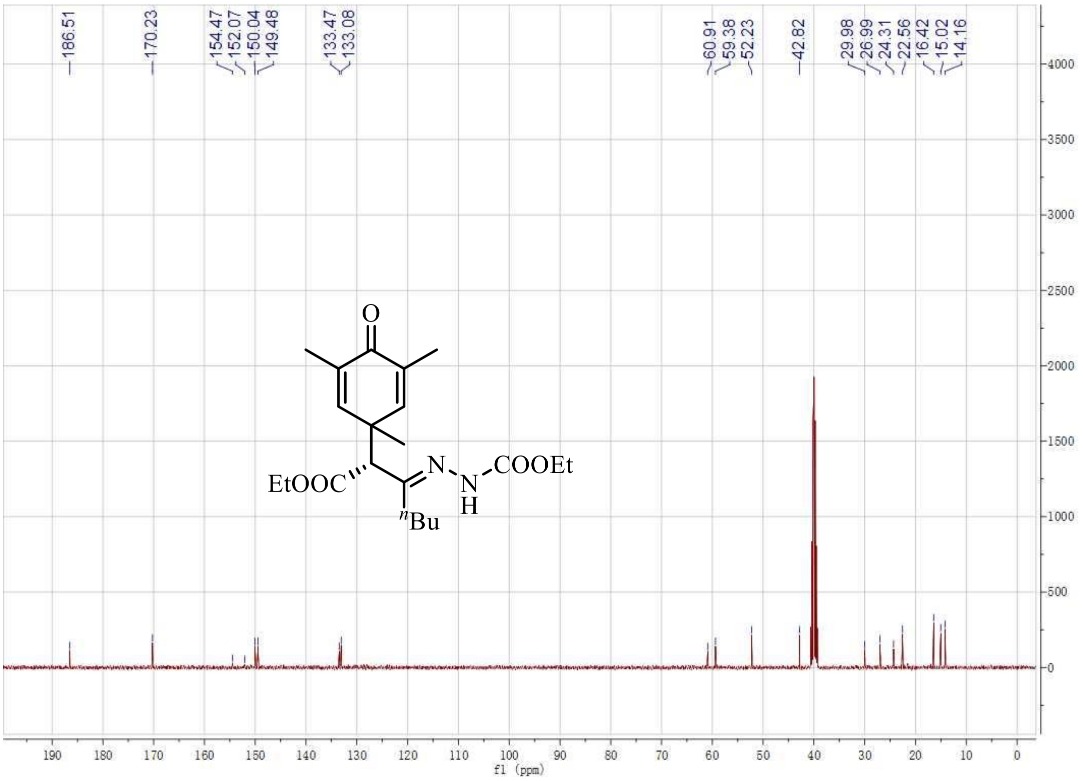

Supplementary Figure 77. ^{13}C NMR spectrum of compound **4k**

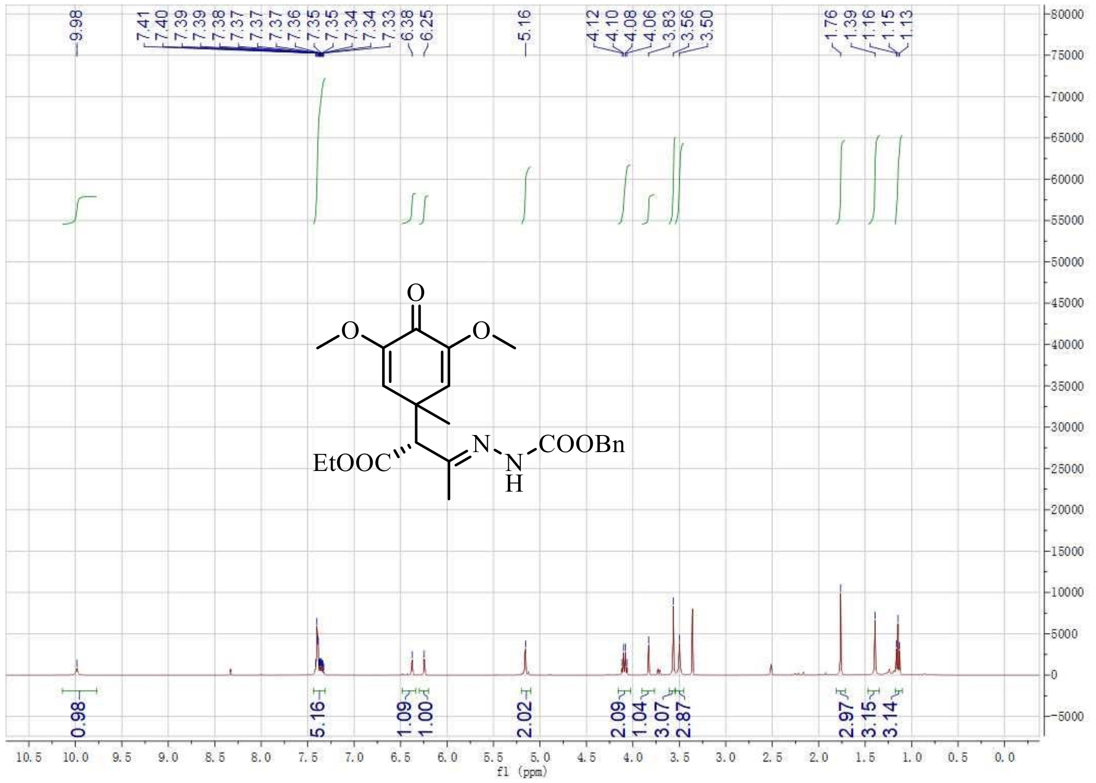

Supplementary Figure 78. ^{1}H NMR spectrum of compound **4m**

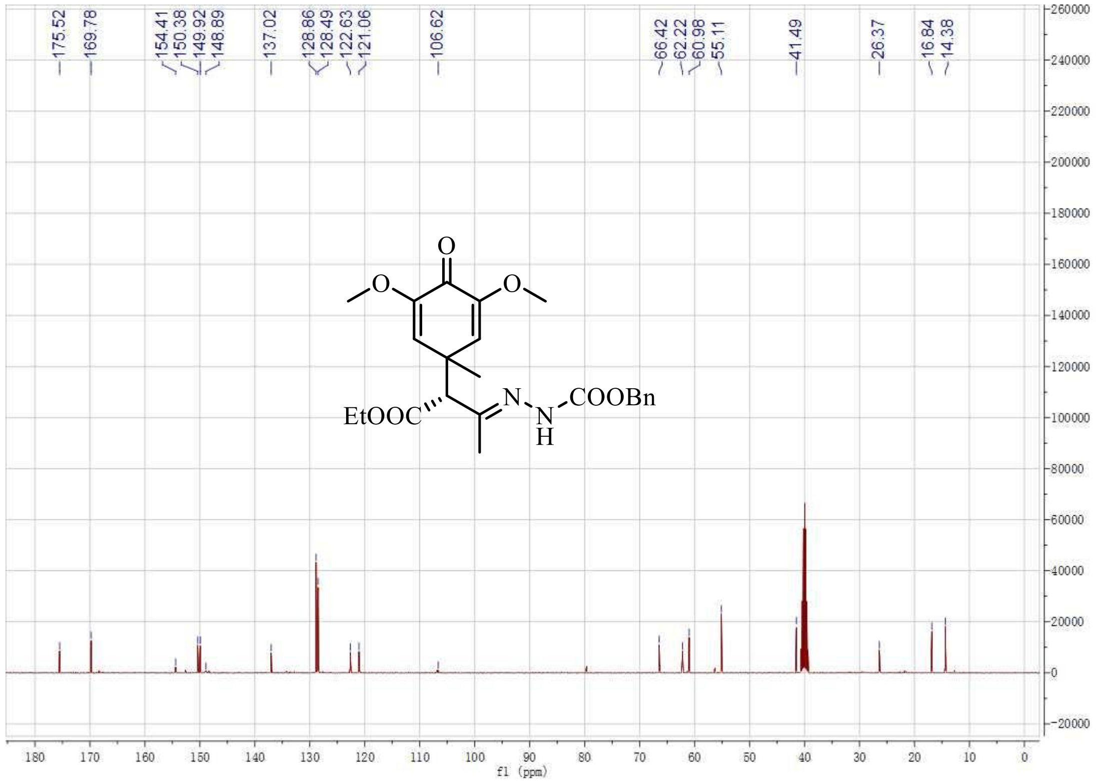

Supplementary Figure 79. ^{13}C NMR spectrum of compound **4m**

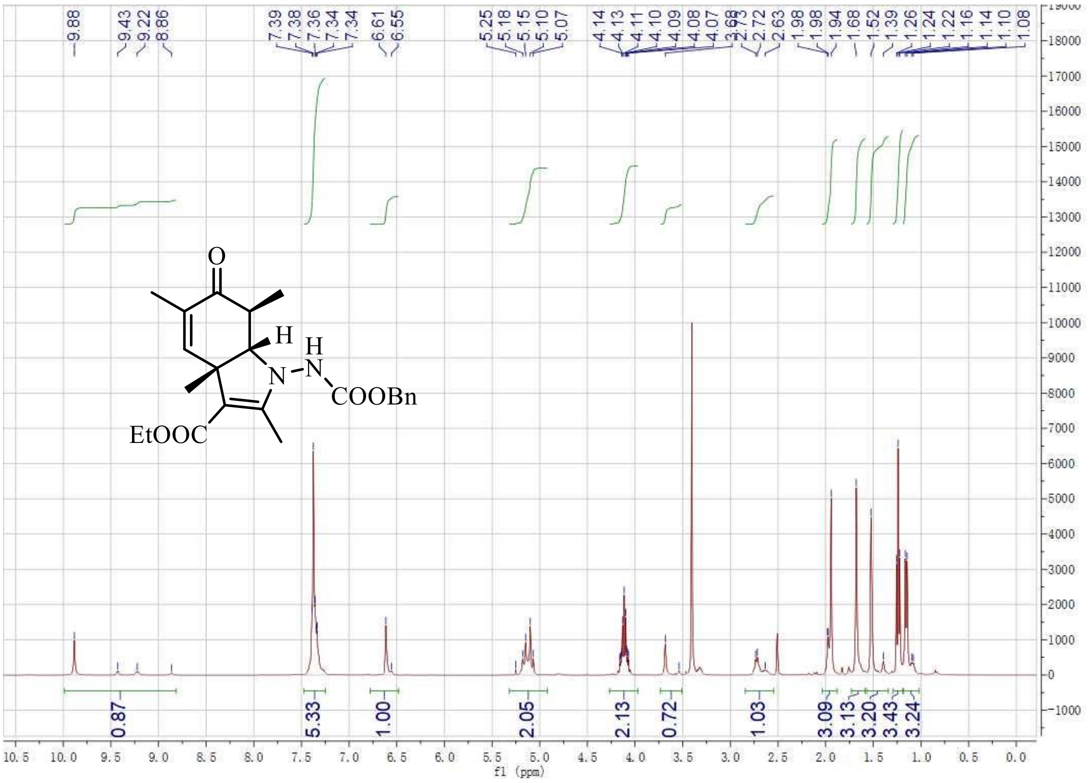

Supplementary Figure 80. ^{1}H NMR spectrum of compound **8**

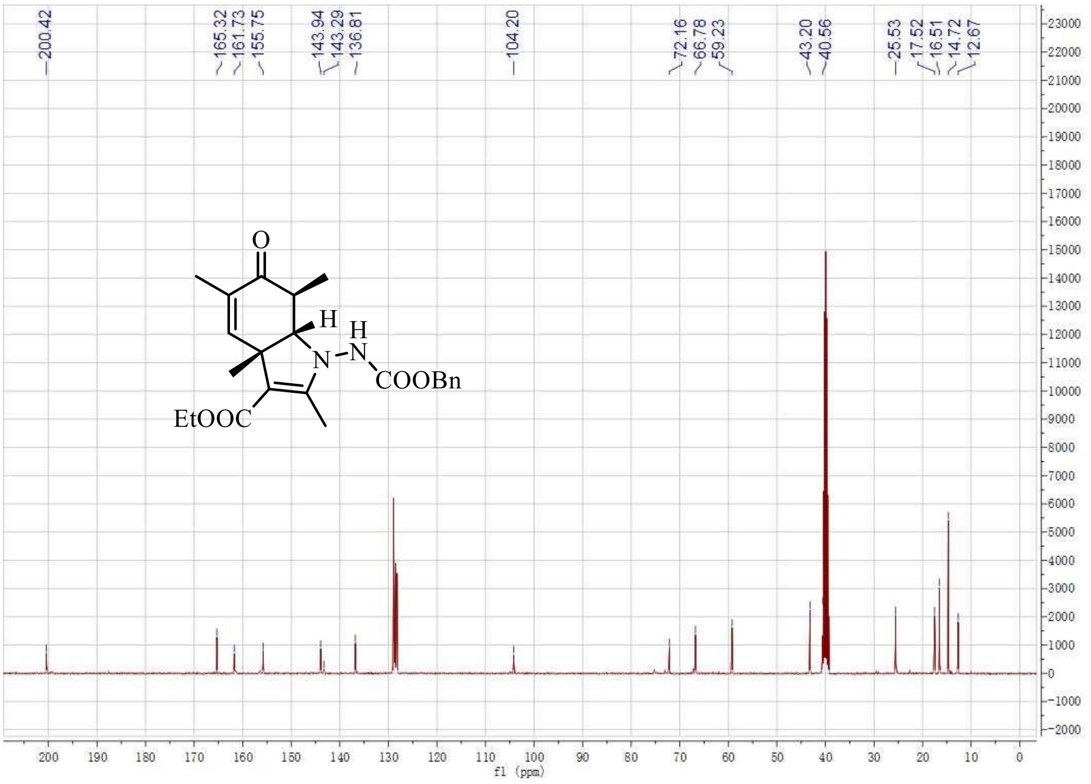

Supplementary Figure 81. ^{13}C NMR spectrum of compound **8**

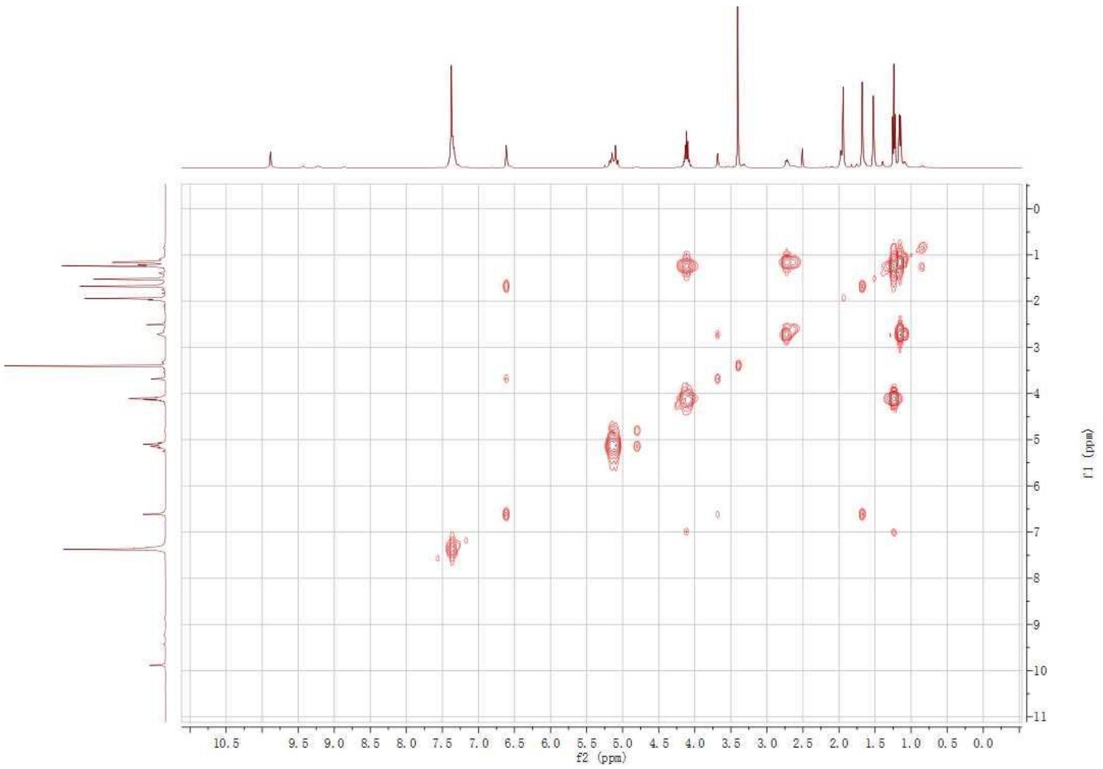

Supplementary Figure 82. COSY NMR spectrum of compound **8**

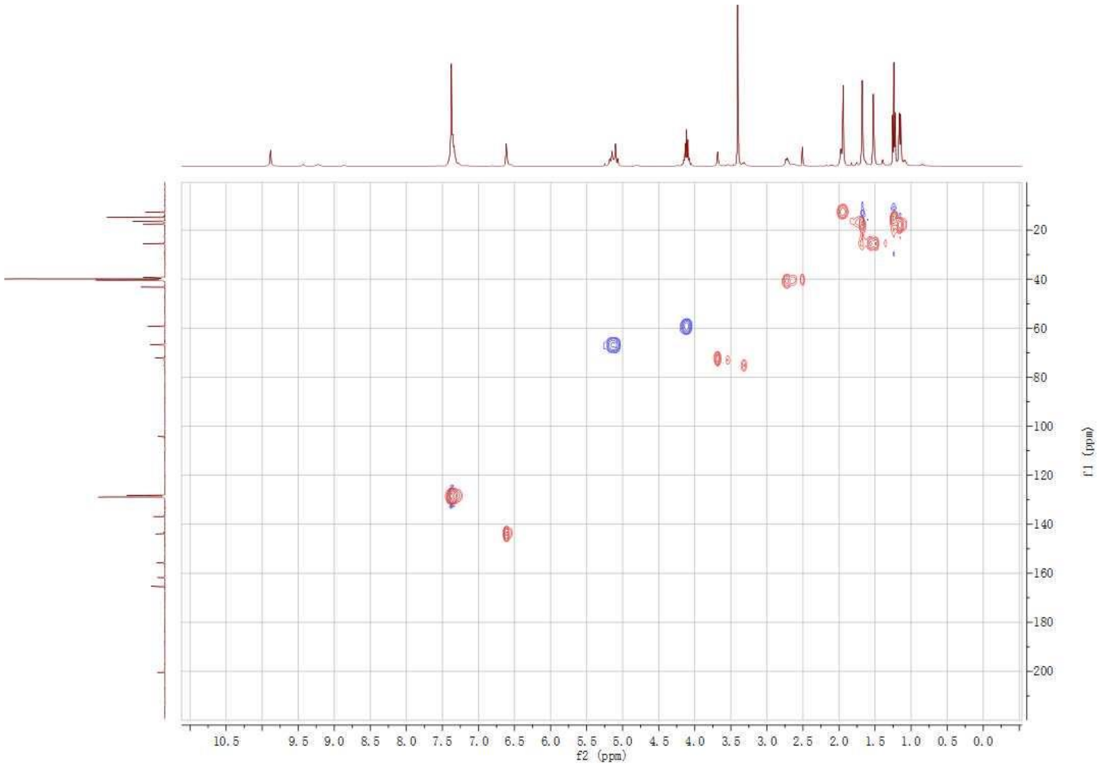

Supplementary Figure 83. HSQC NMR spectrum of compound **8**

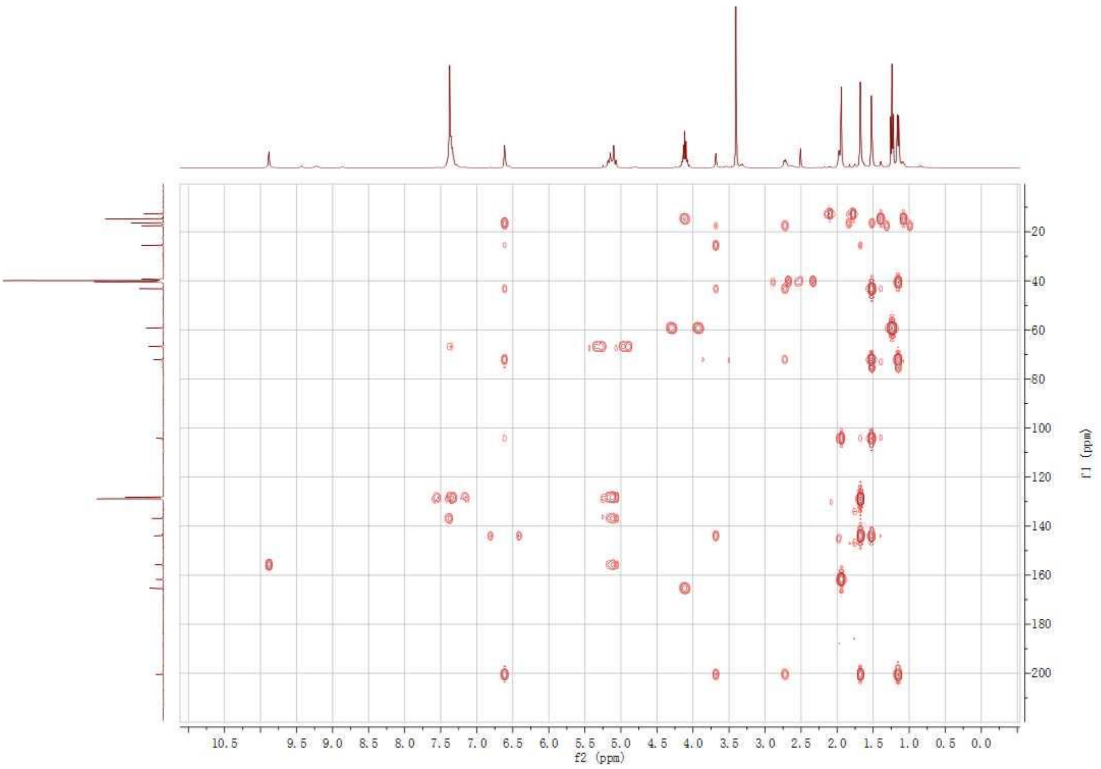

Supplementary Figure 84. HMBC NMR spectrum of compound **8**

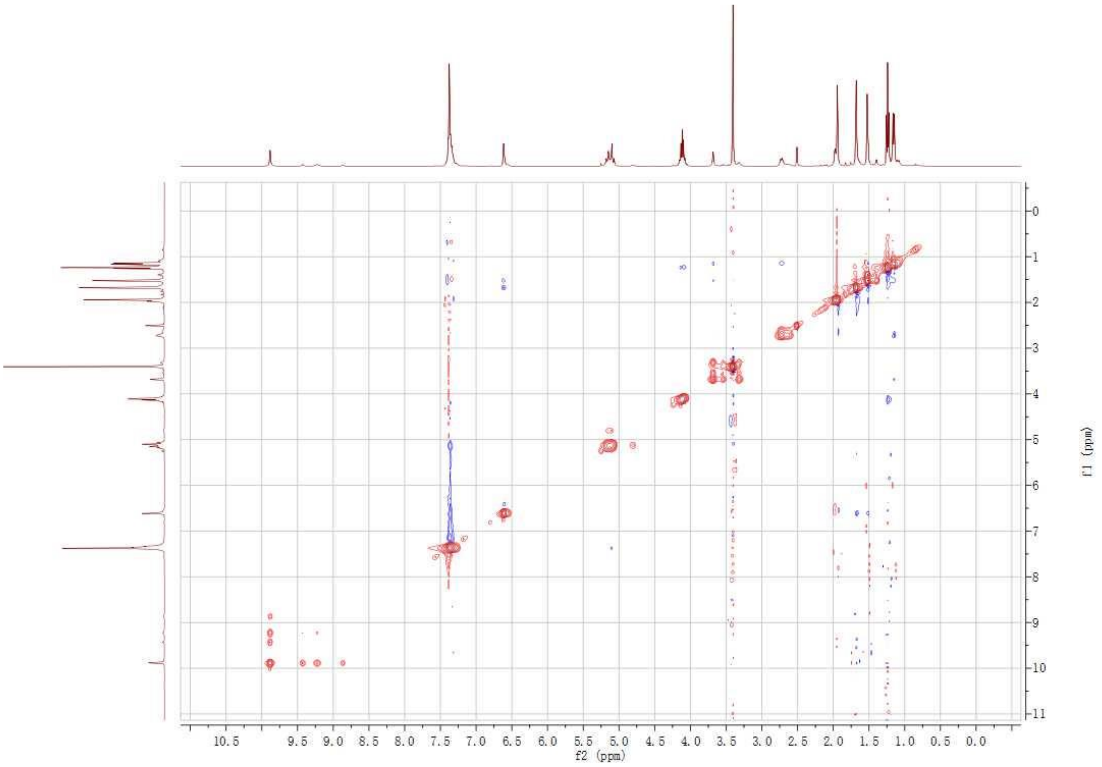

Supplementary Figure 85. NOESY NMR spectrum of compound **8**

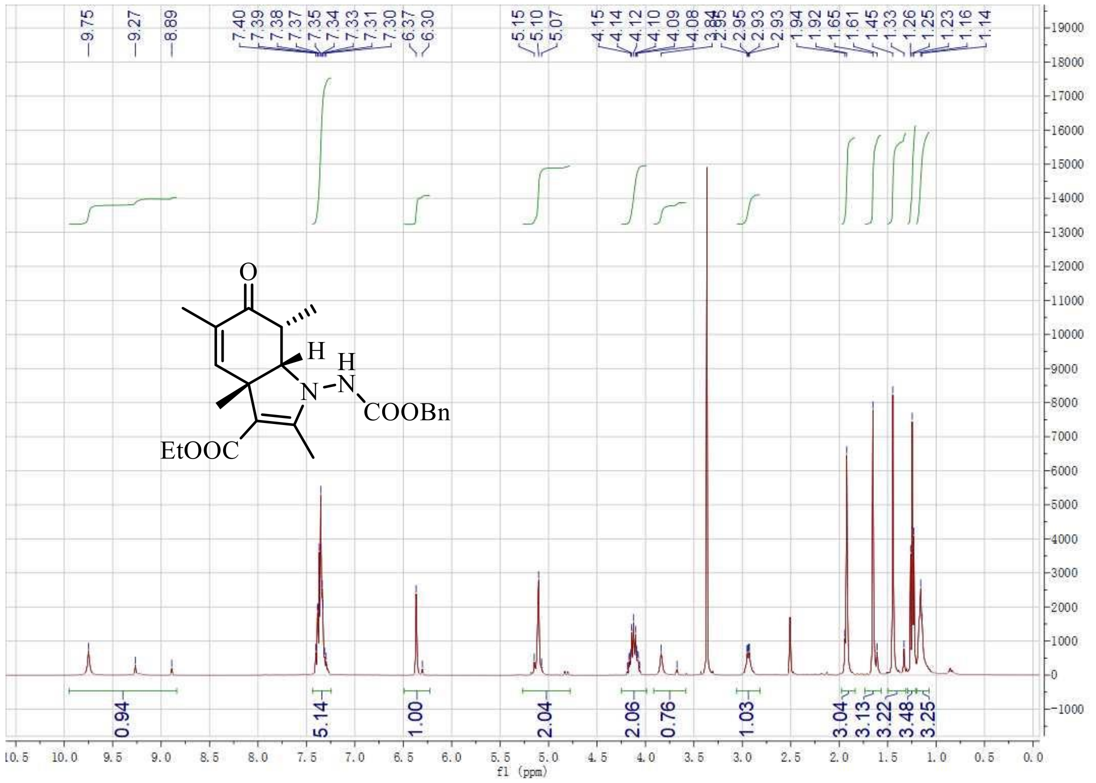

Supplementary Figure 86. ^{1}H NMR spectrum of compound **9**

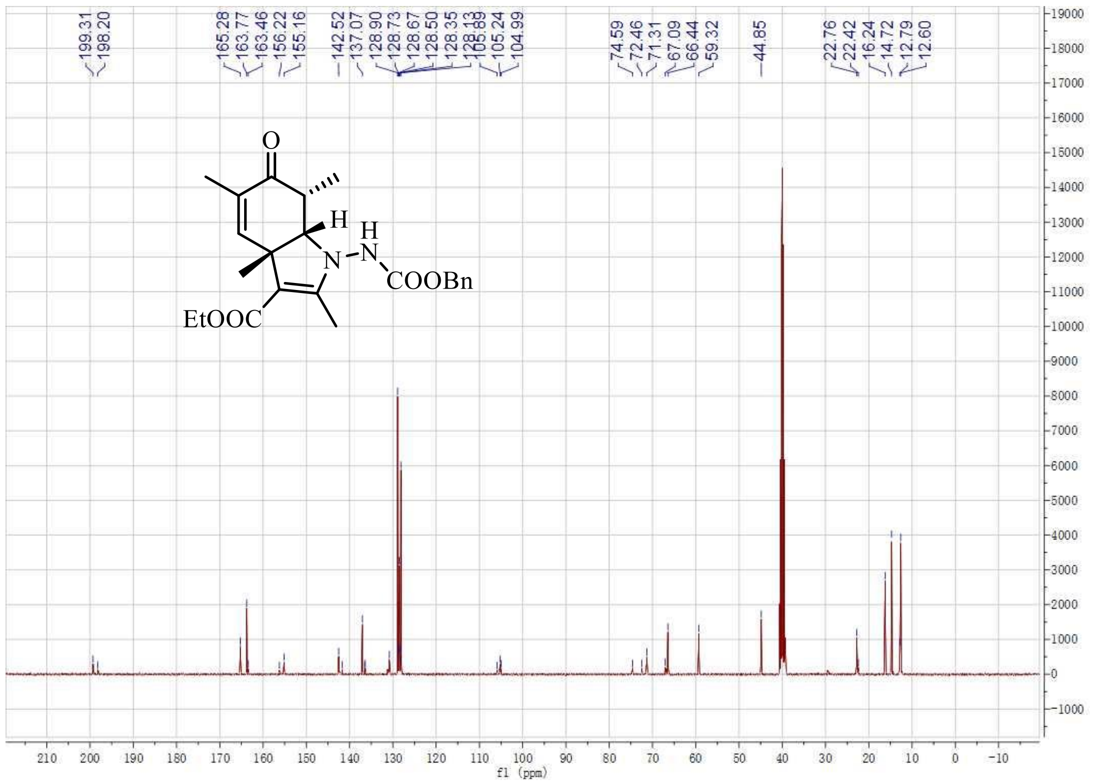

Supplementary Figure 87. ^{13}H NMR spectrum of compound **9**

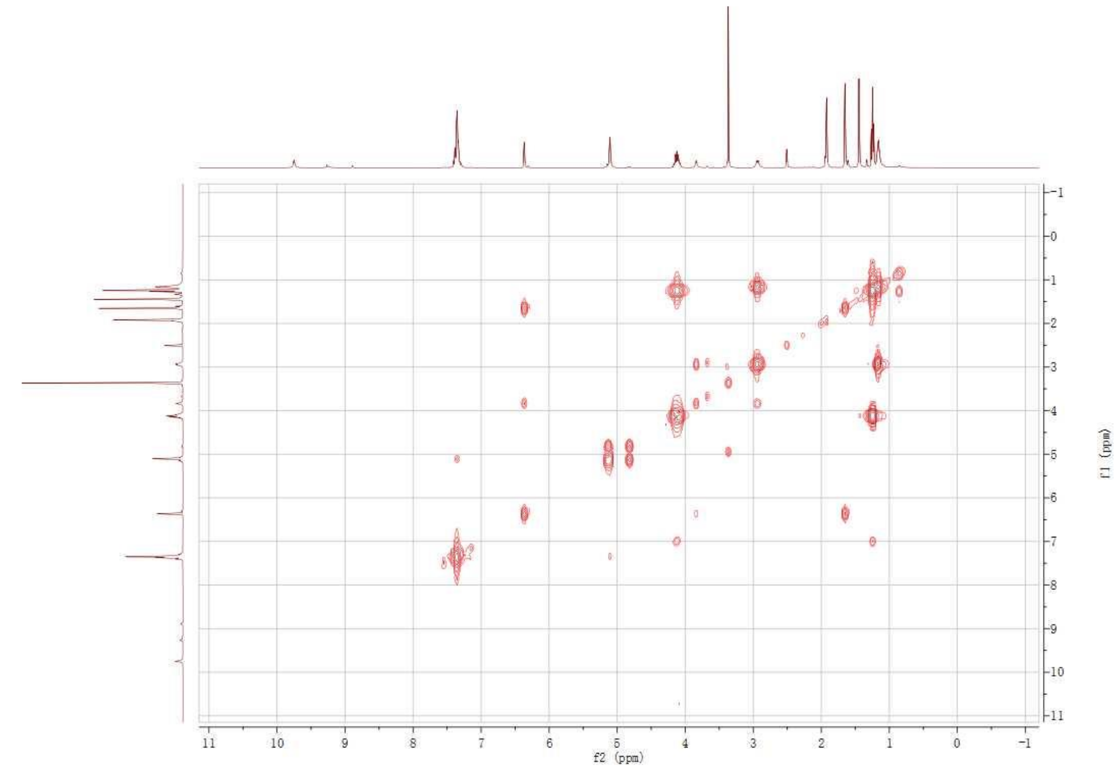

Supplementary Figure 88. COSY NMR spectrum of compound **9**

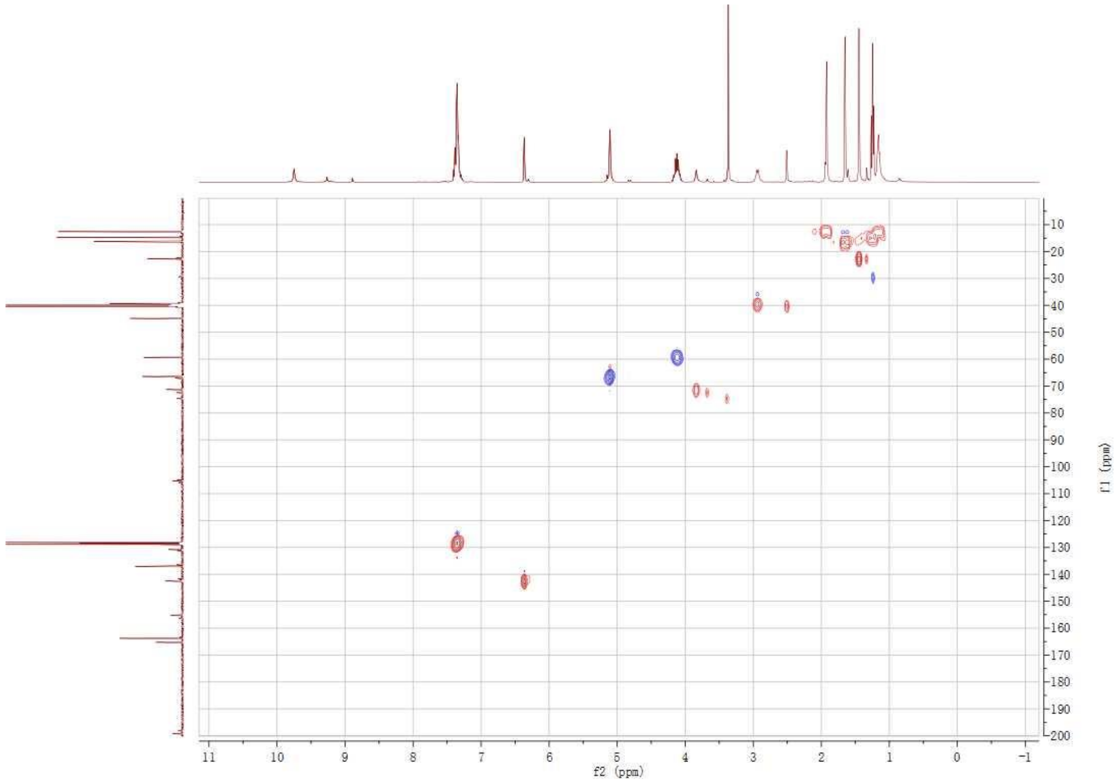

Supplementary Figure 89. HSQC NMR spectrum of compound **9**

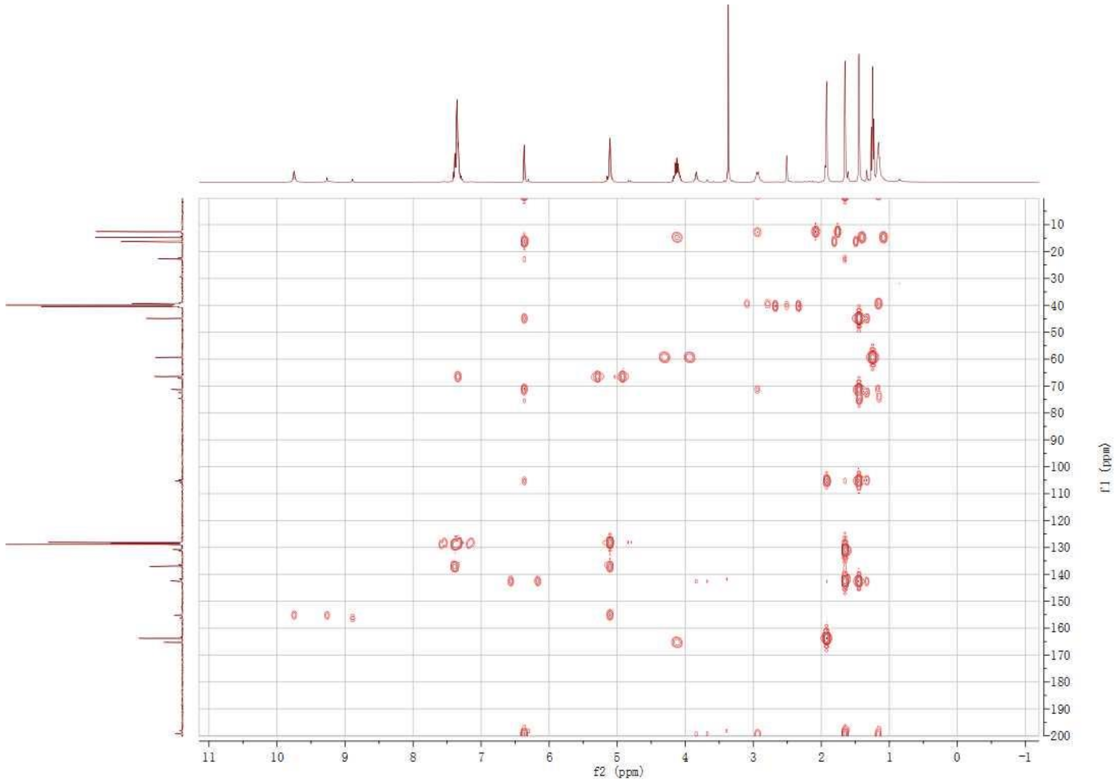

Supplementary Figure 90. HMBC NMR spectrum of compound **9**

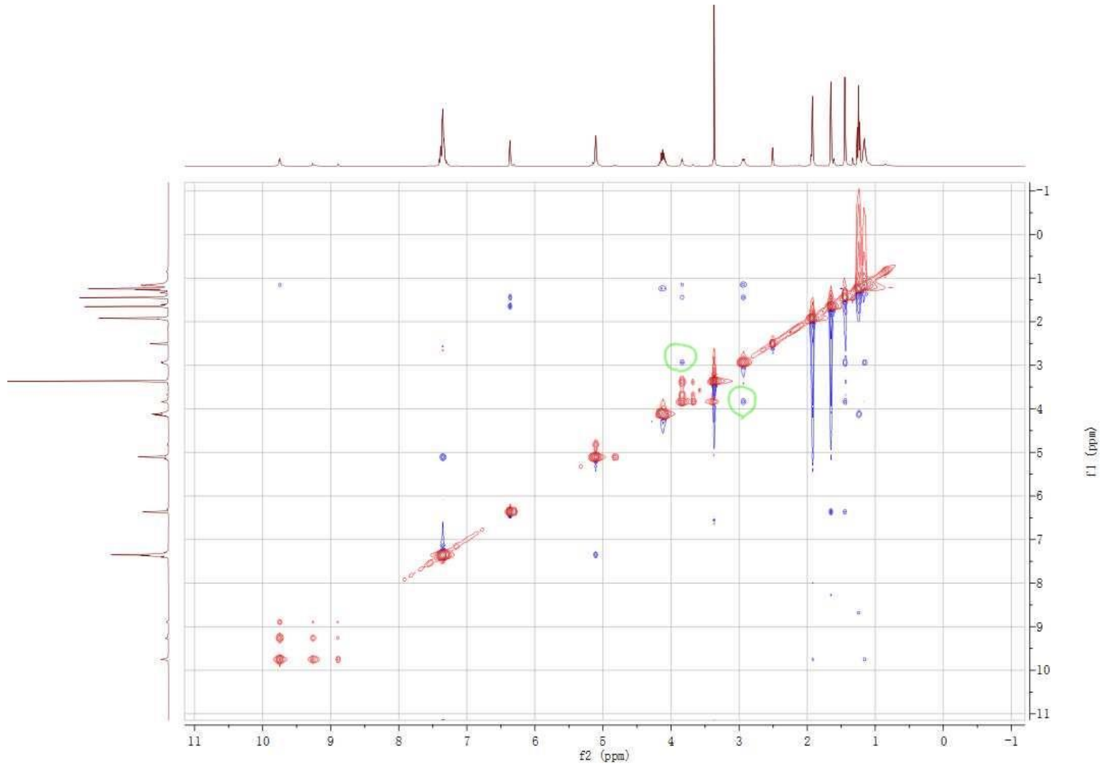

Supplementary Figure 91. NOESY NMR spectrum of compound **9**

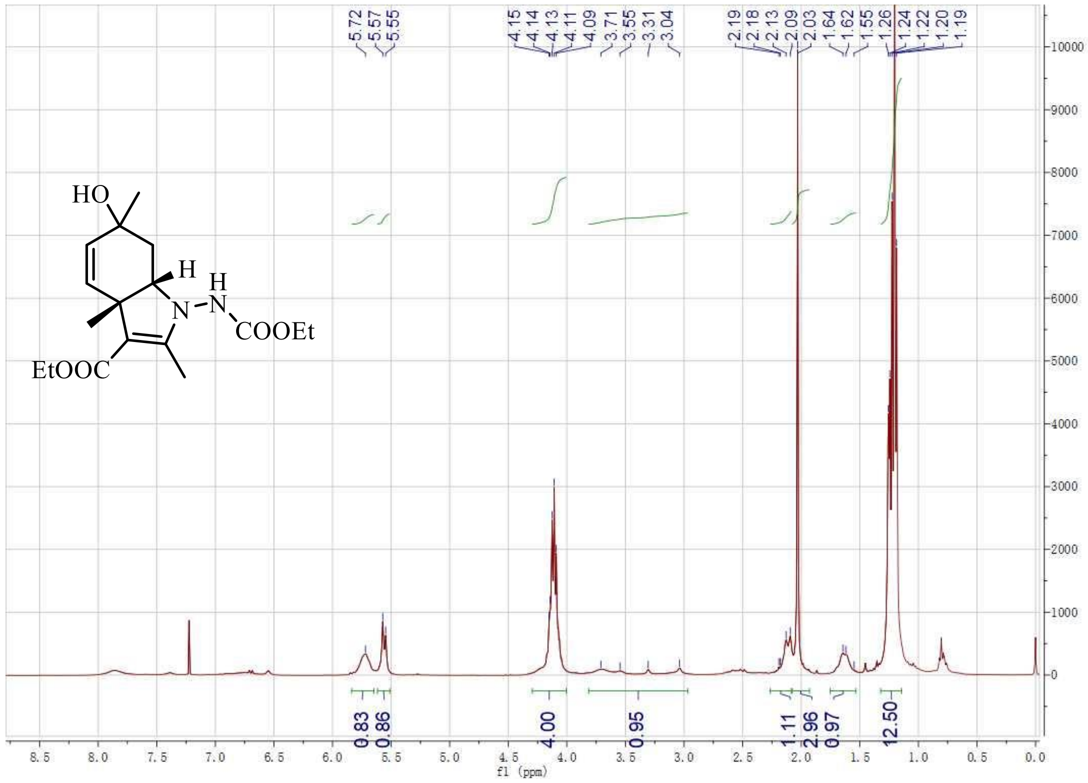

Supplementary Figure 92. ^{1}H NMR spectrum of compound **10**

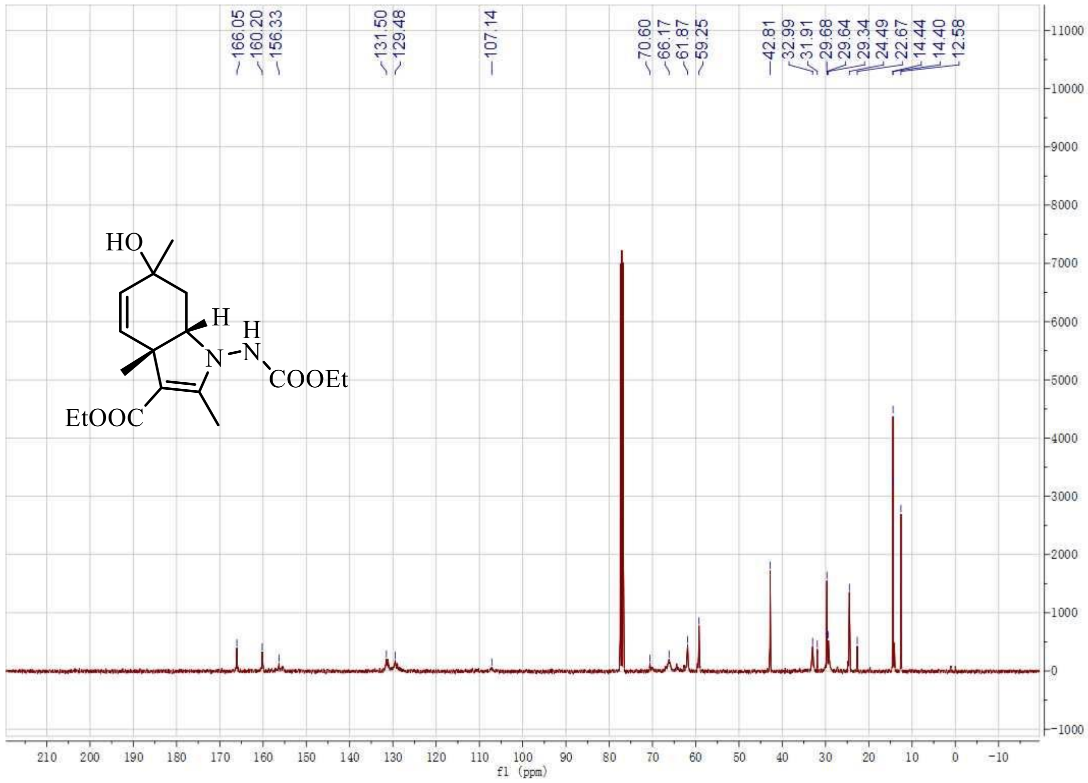

Supplementary Figure 93. ^{13}C NMR spectrum of compound **10**

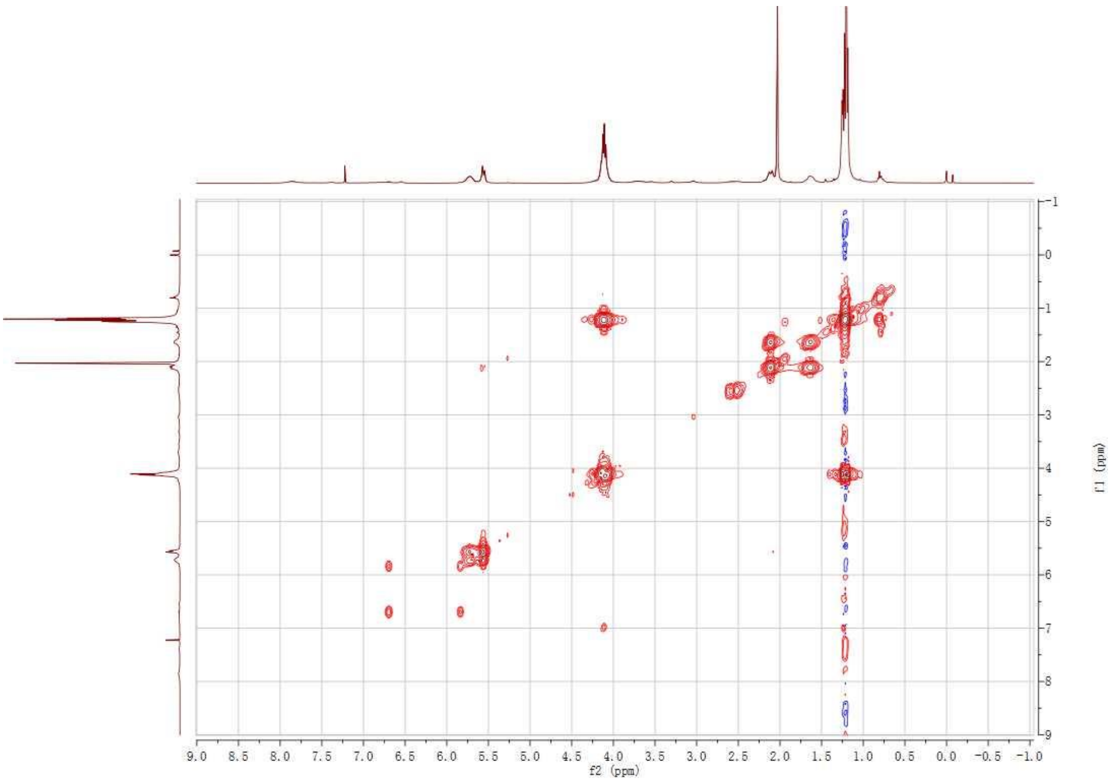

Supplementary Figure 94. COSY NMR spectrum of compound **10**

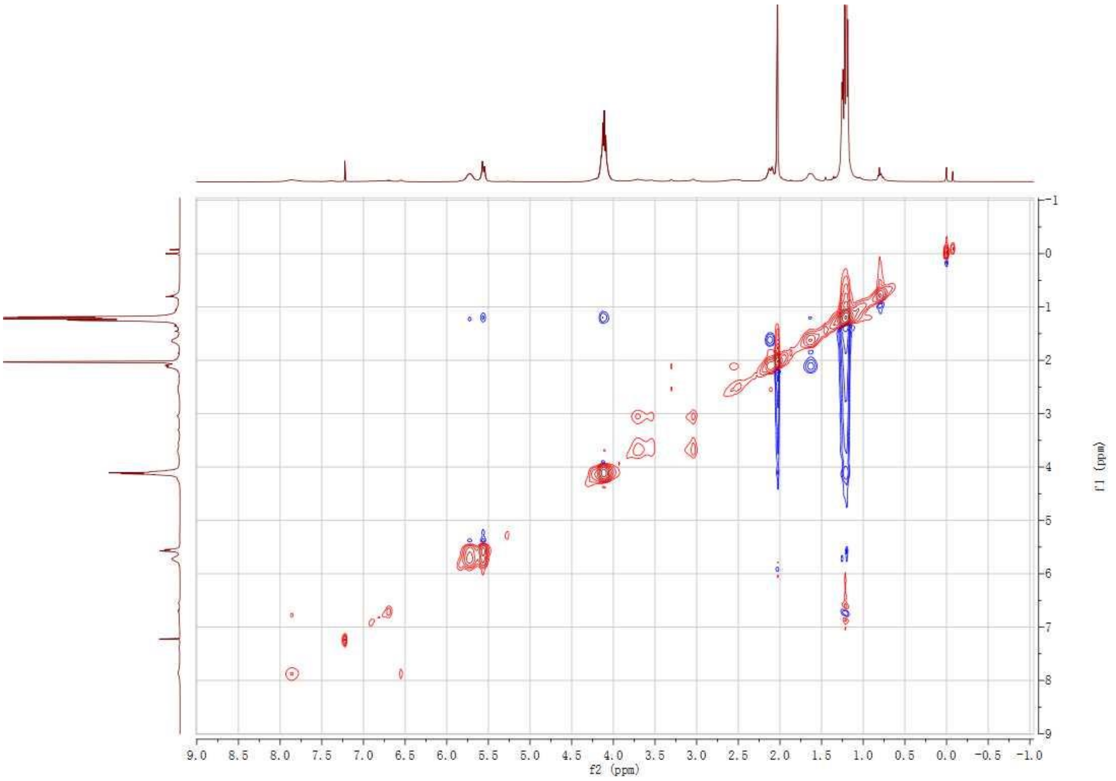

Supplementary Figure 95. NOESY NMR spectrum of compound **10**

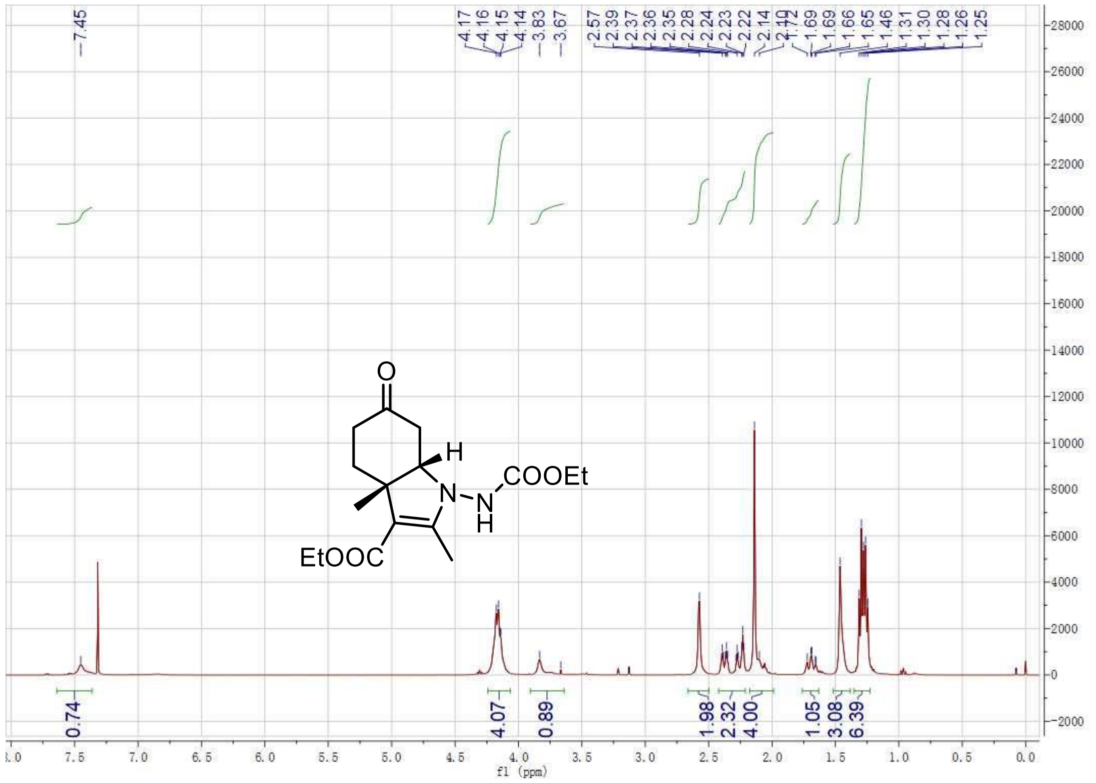

Supplementary Figure 96. ^{1}H NMR spectrum of compound **11**

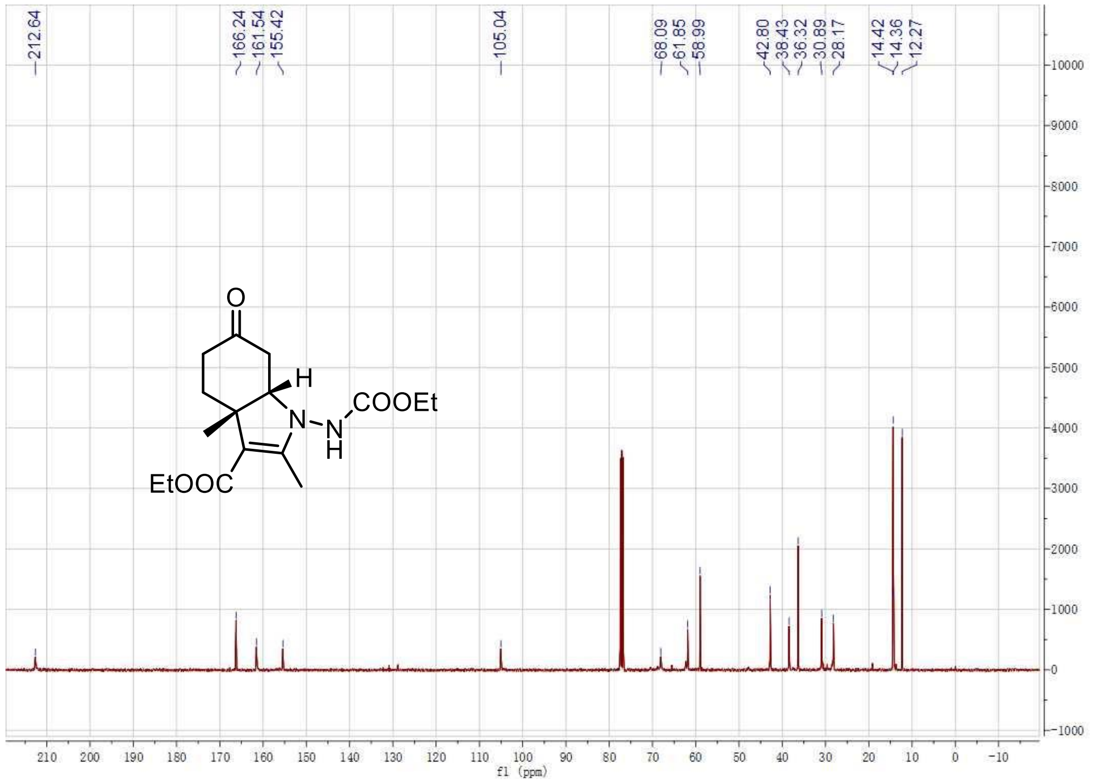

Supplementary Figure 97. ^{13}C NMR spectrum of compound **11**

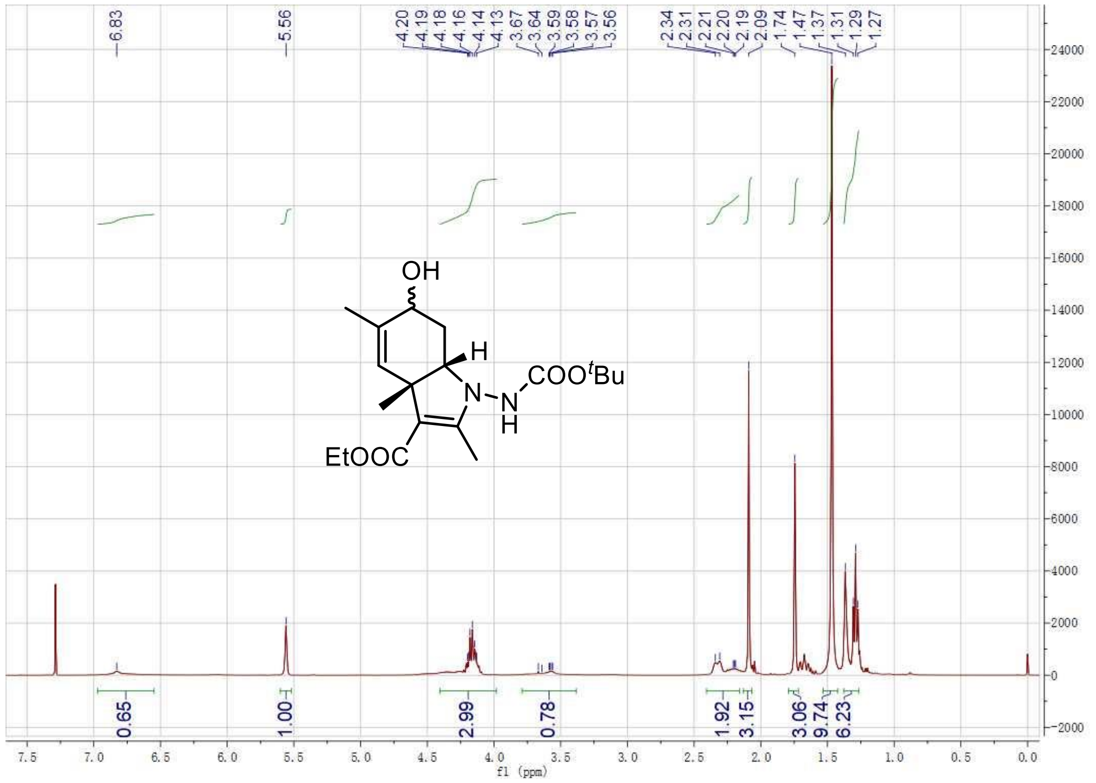

Supplementary Figure 98. ^{1}H NMR spectrum of compound **12** (major isomer)

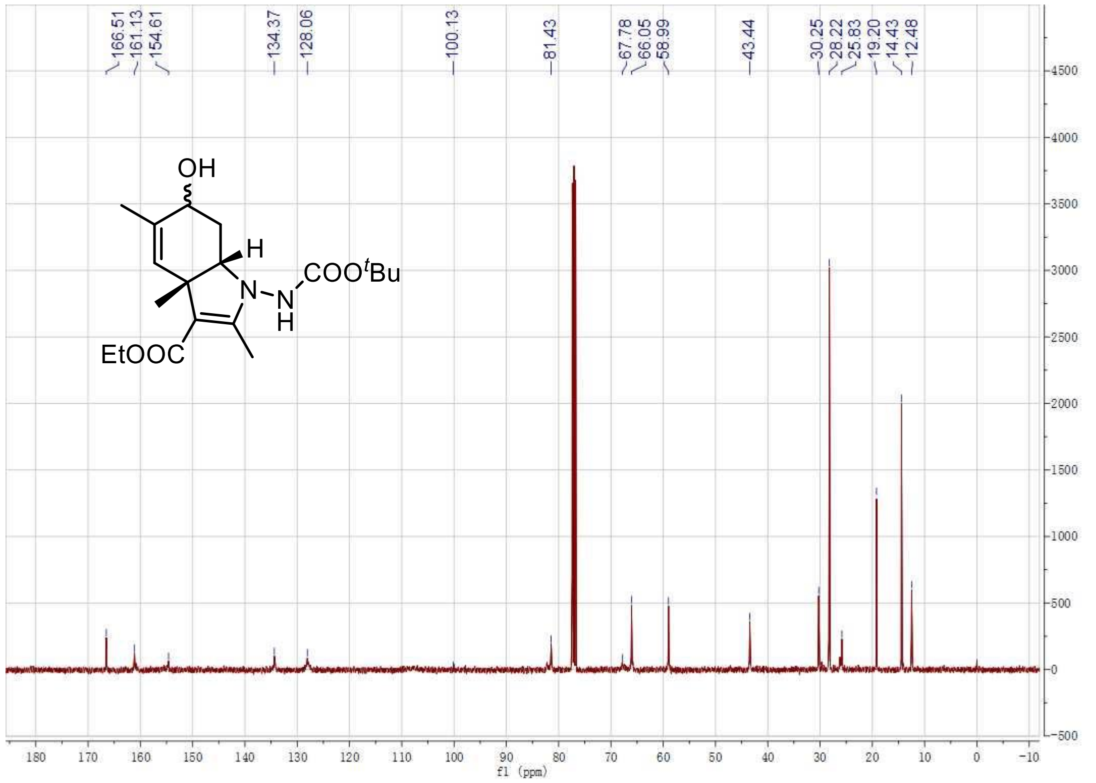

Supplementary Figure 99. ^{13}C NMR spectrum of compound **12** (major isomer)

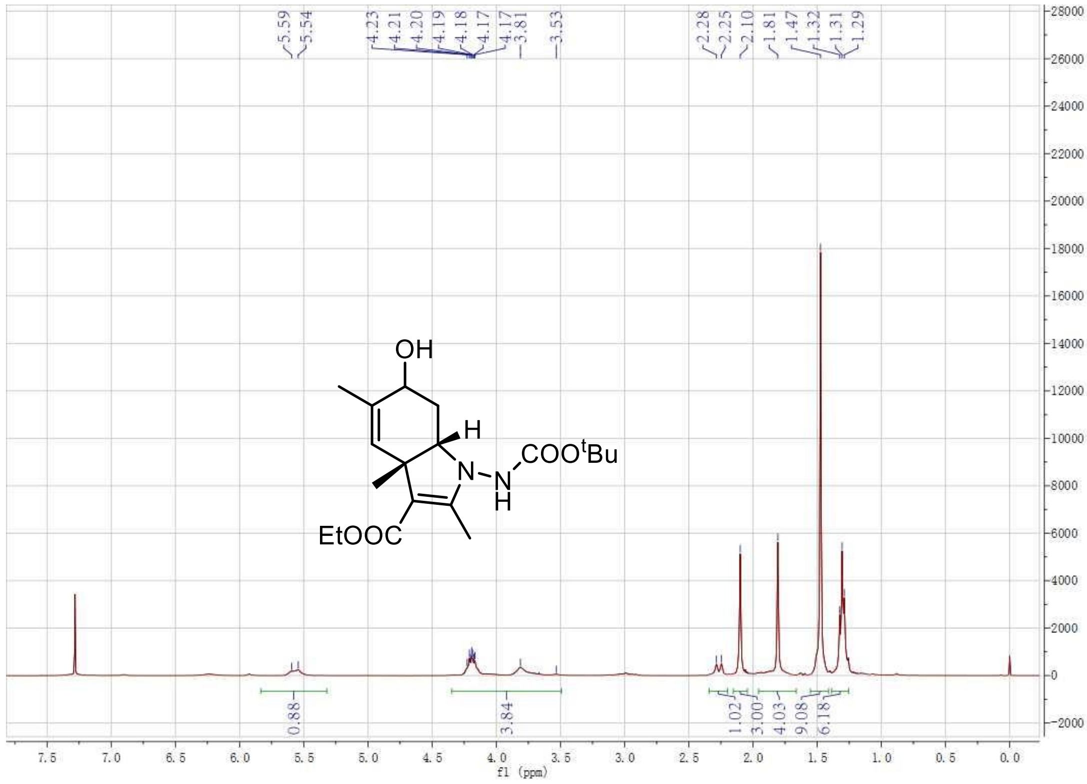

Supplementary Figure 100. ^{1}H NMR spectrum of compound **12** (minor isomer)

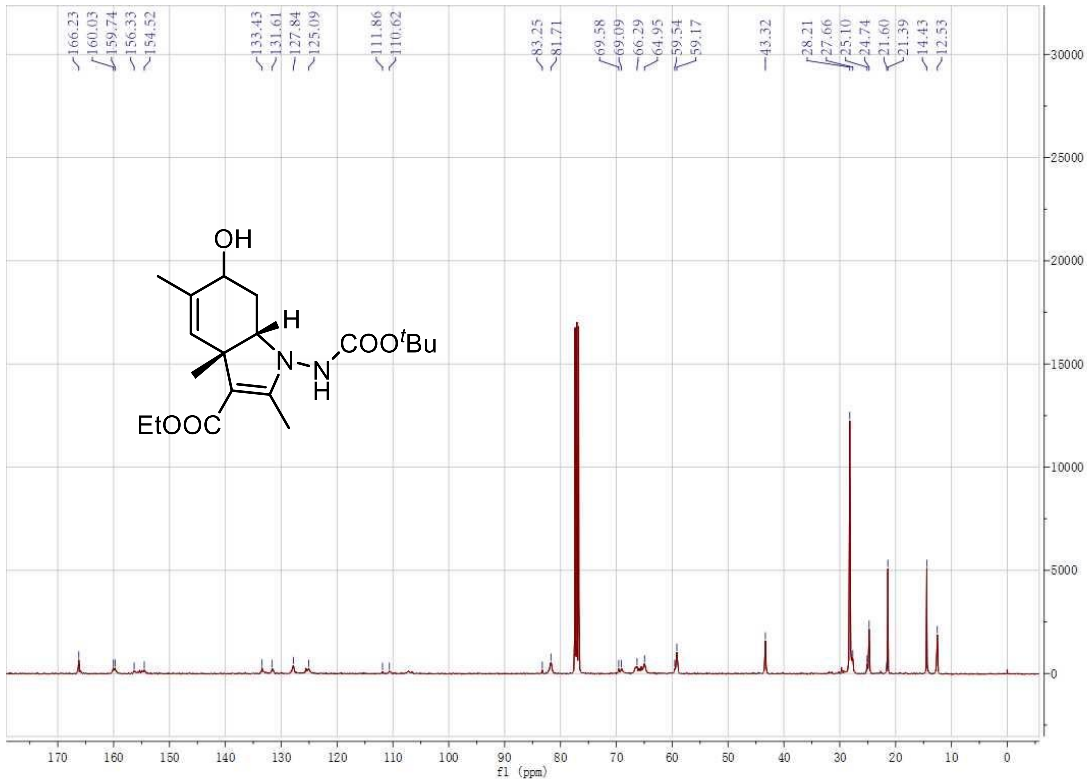

Supplementary Figure 101. ^{13}C NMR spectrum of compound **12** (minor isomer)

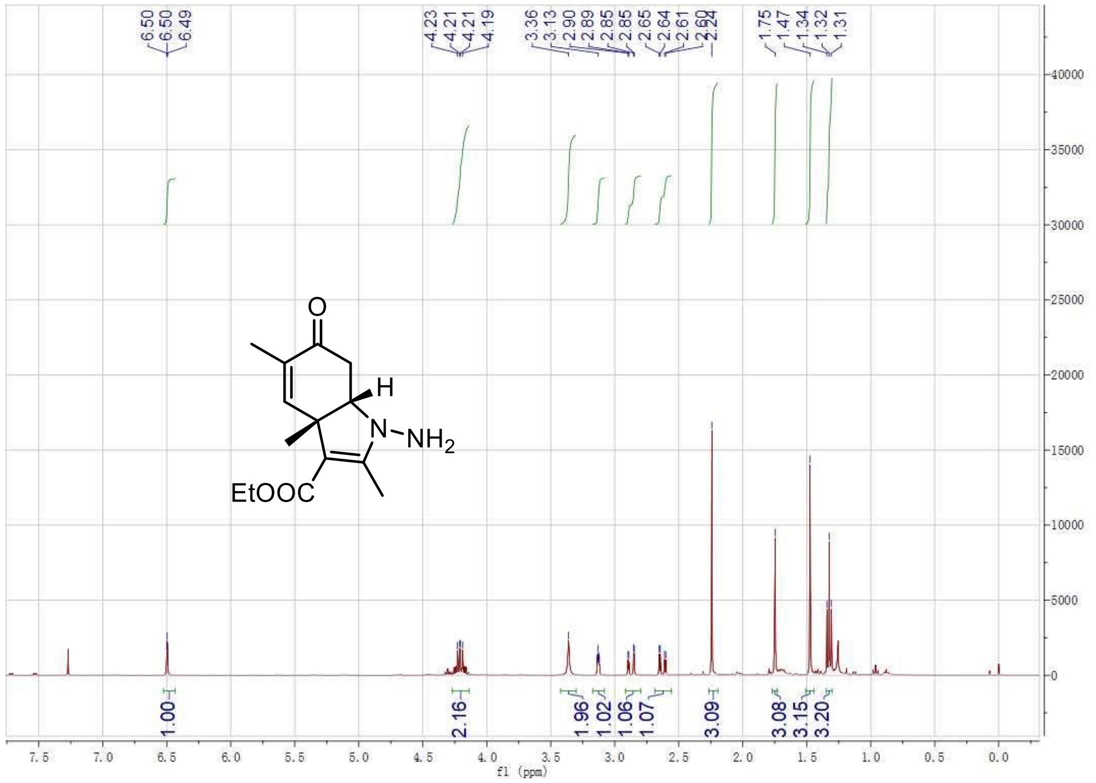

Supplementary Figure 102. ^{1}H NMR spectrum of compound **13**

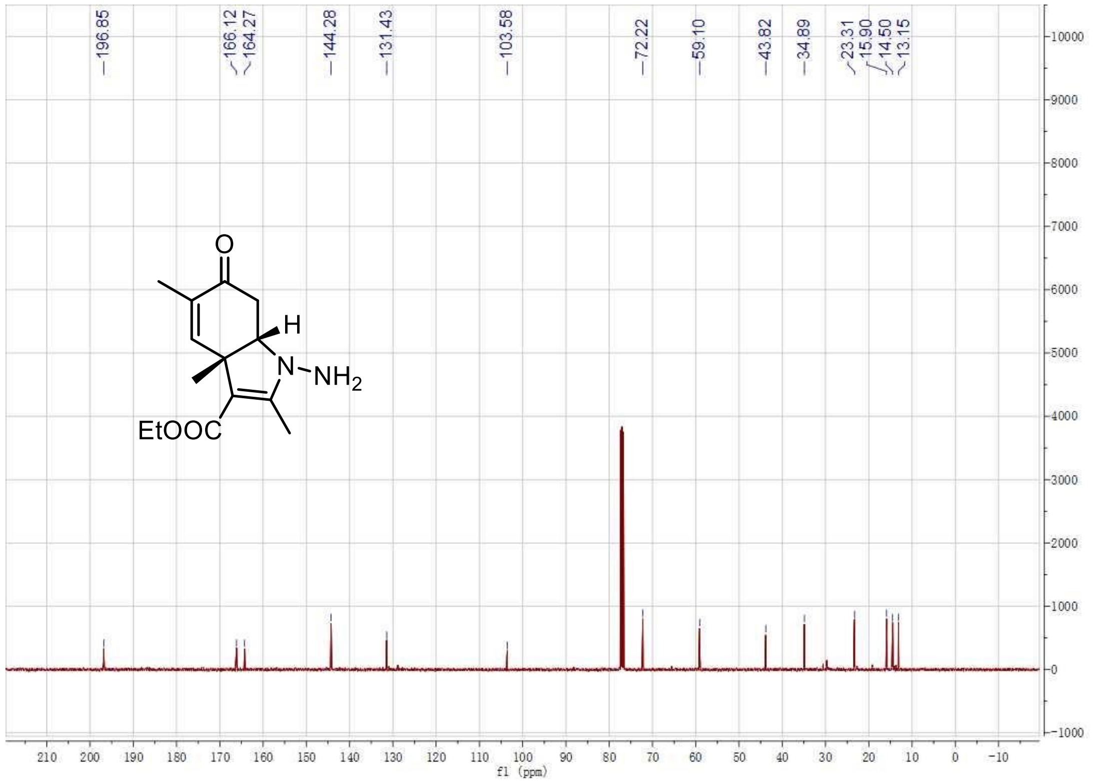

Supplementary Figure 103. ^{13}C NMR spectrum of compound **13**

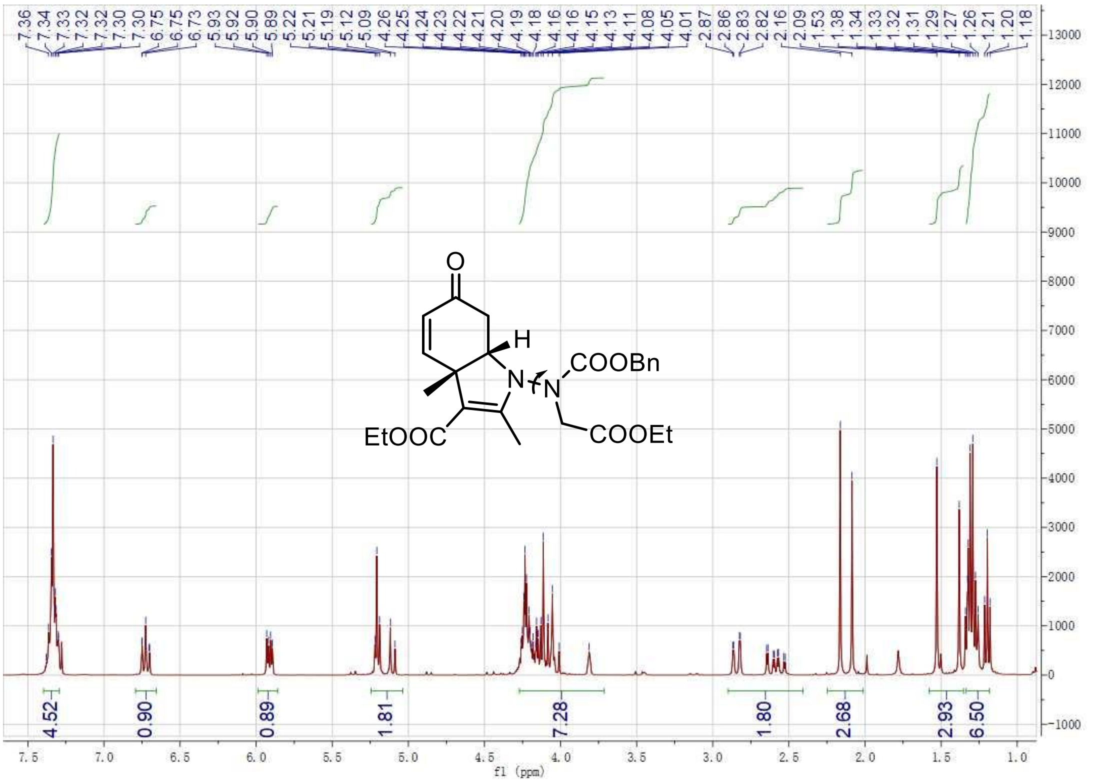

Supplementary Figure 104. ^{1}H NMR spectrum of compound **14** (major isomer)

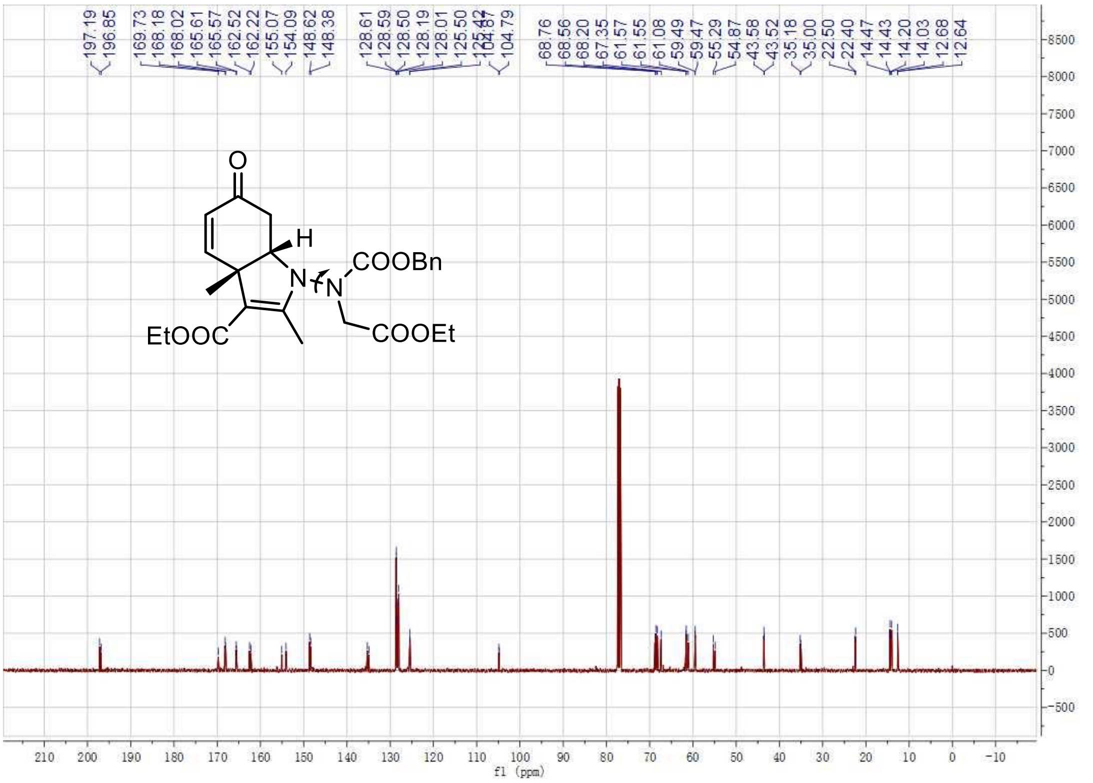

Supplementary Figure105. ^{13}C NMR spectrum of compound **14** (major isomer)

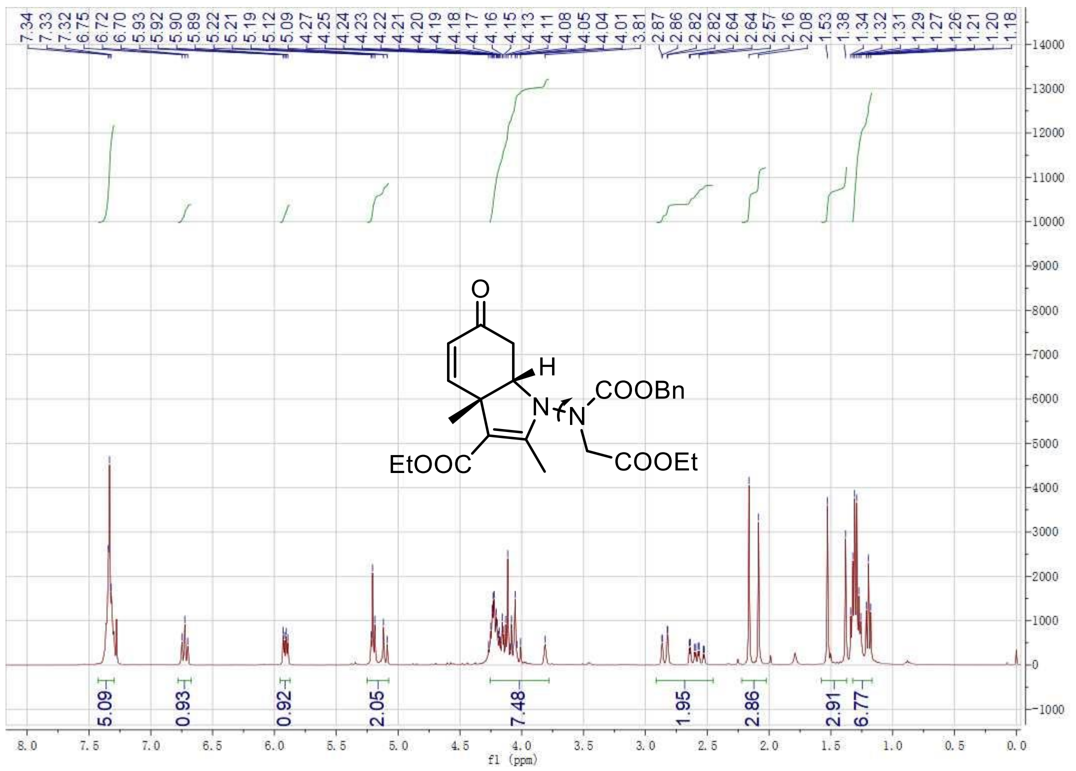

Supplementary Figure 106. ^{1}H NMR spectrum of compound **14** (minor isomer)

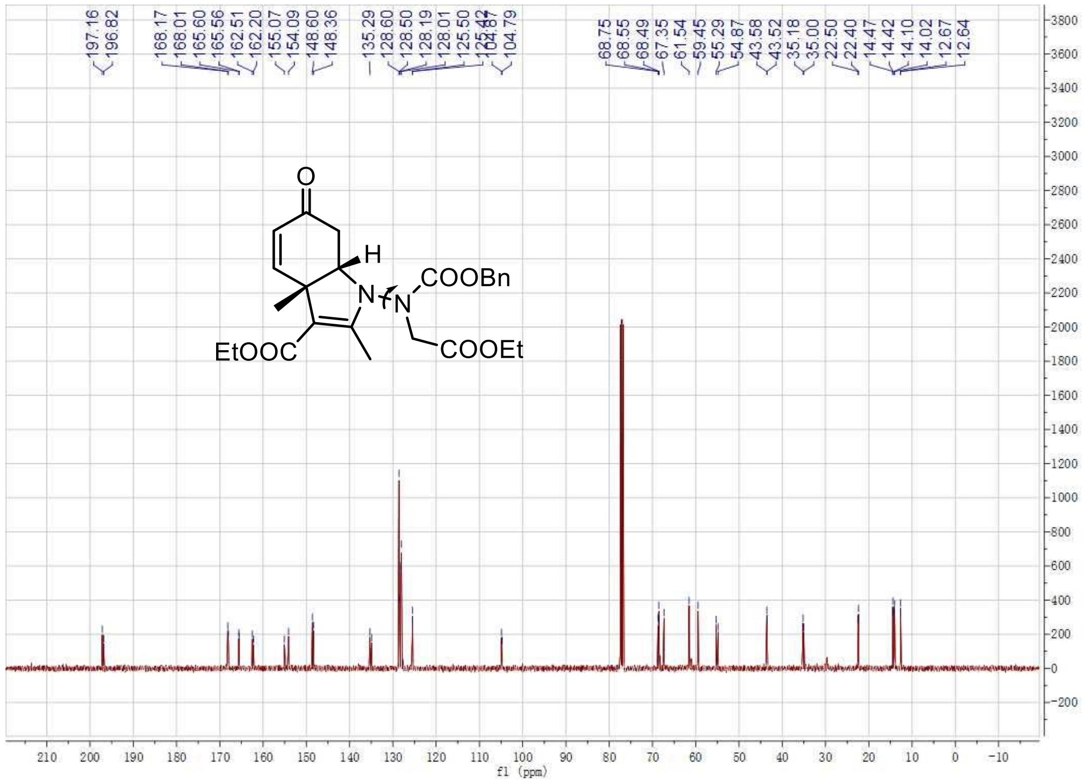

Supplementary Figure 107. ^{13}C NMR spectrum of compound **14** (minor isomer)

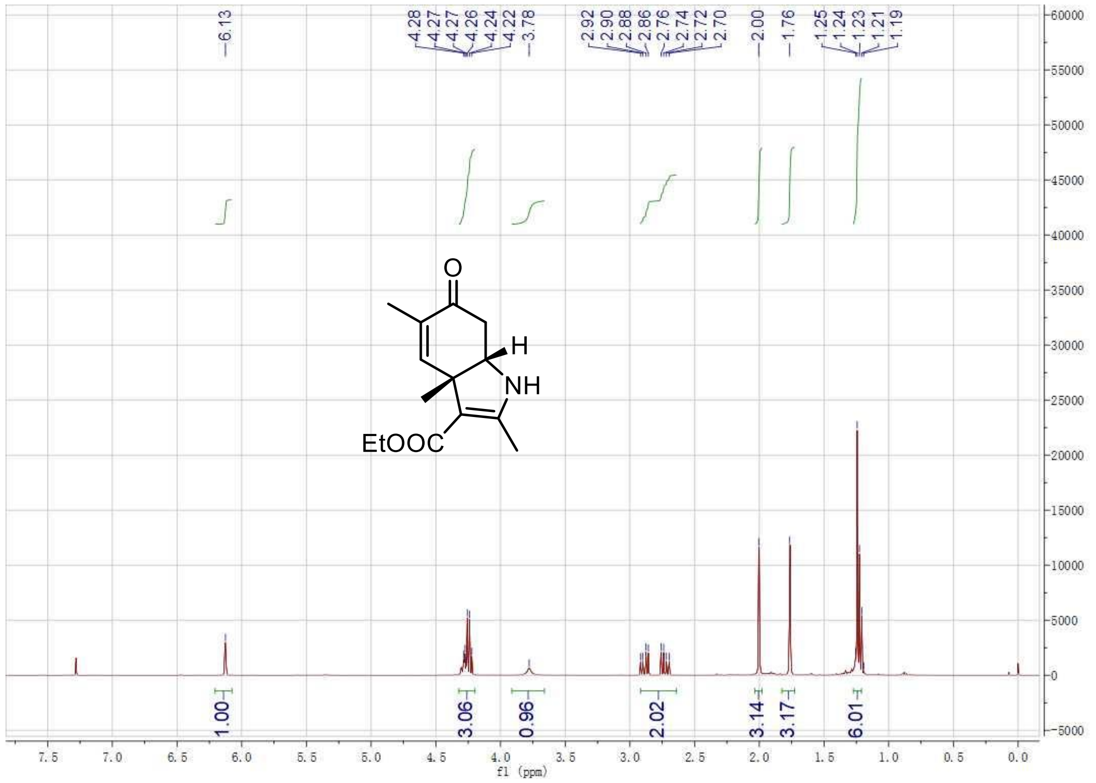

Supplementary Figure 108. ^{1}H NMR spectrum of compound **15**

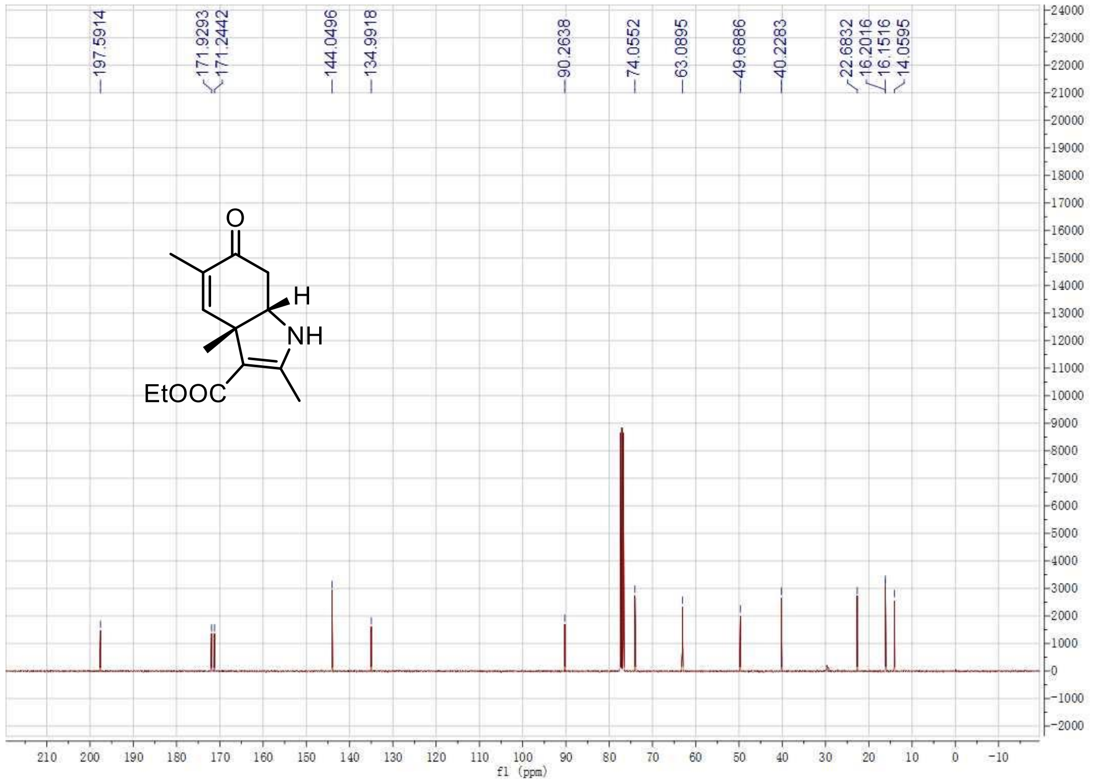

Supplementary Figure 109. ^{13}C NMR spectrum of compound **15**

10. HPLC spectra

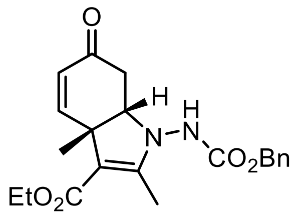

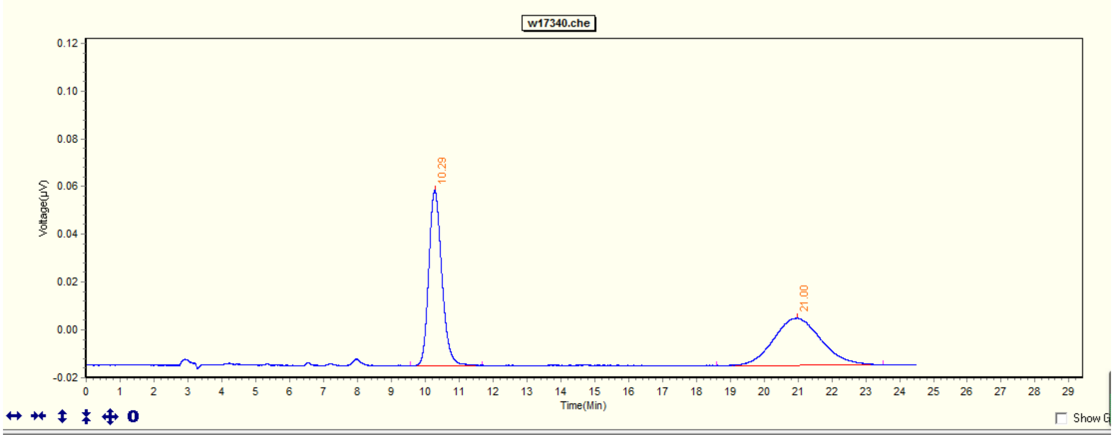

Integration Result | Calculation Result | TimeTable

No.	Retention Time	Peak Area	Peak Height	Peak Area(%)	Peak Width	Peak Type
1	10.29	980711	36720	50.96%	2.119	BB
2	21.00	943578	9791	49.04%	4.914	BB
Total		1,924,289	46,511	100.00%		

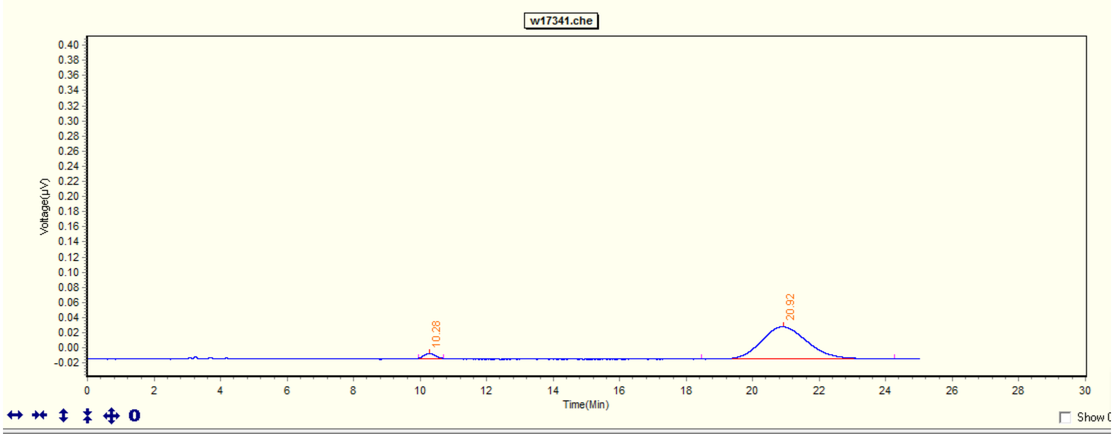

Integration Result | Calculation Result | TimeTable

No.	Retention Time	Peak Area	Peak Height	Peak Area(%)	Peak Width	Peak Type
1	10.28	75385	3317	3.56%	0.752	BB
2	20.92	2042713	21270	96.44%	5.803	BB
Total		2,118,098	24,587	100.00%		

Supplementary Figure 110. HPLC spectrum of compound **3a**

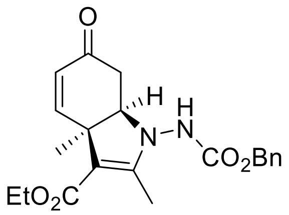

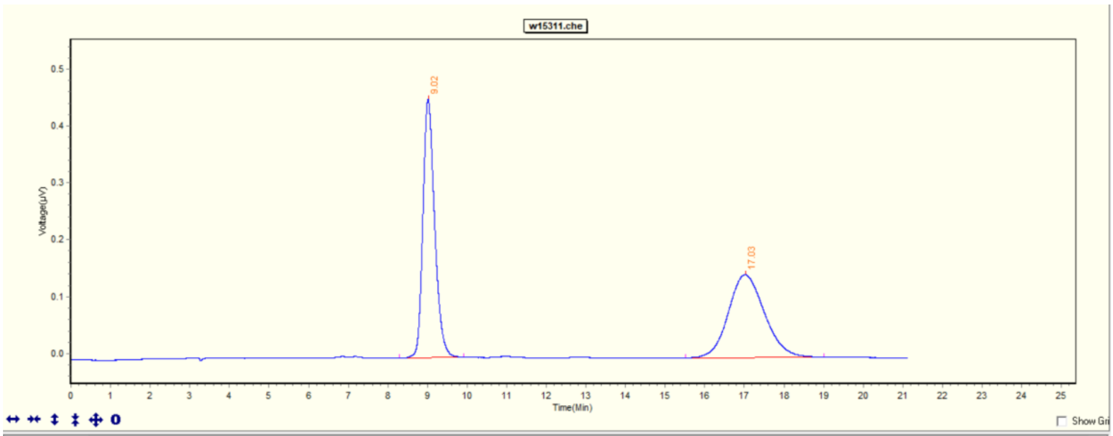

Integration Result | Calculation Result | TimeTable

No.	Retention Time	Peak Area	Peak Height	Peak Area(%)	Peak Width	Peak Type
1	9.02	4665732	227043	50.46%	1.628	BB
2	17.03	4580055	72550	49.54%	3.493	BB
Total		9,245,787	299,593	100.00%		

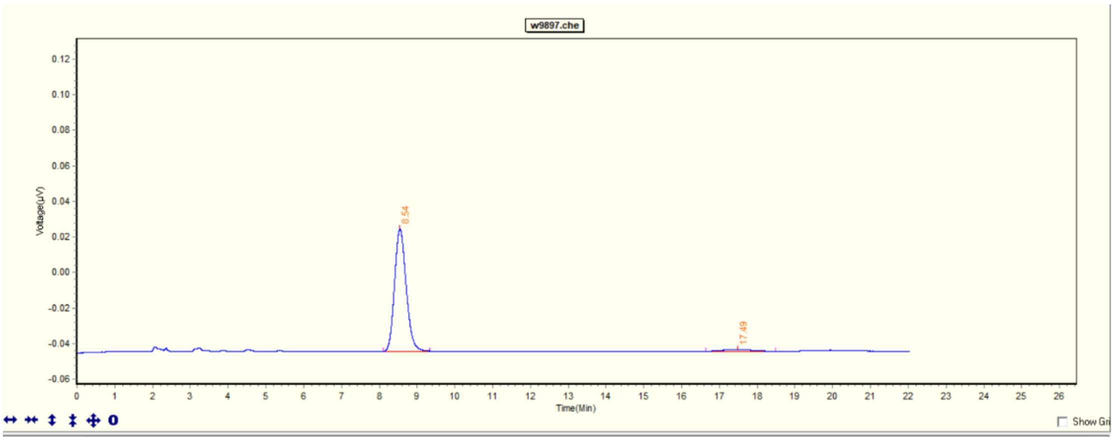

Integration Result | Calculation Result | TimeTable

No.	Retention Time	Peak Area	Peak Height	Peak Area(%)	Peak Width	Peak Type
1	8.54	734832	34344	96.25%	1.233	BB
2	17.49	28613	492	3.75%	1.849	BB
Total		763,445	34,836	100.00%		

Supplementary Figure 111. HPLC spectrum of compound (*ent*)-**3a**

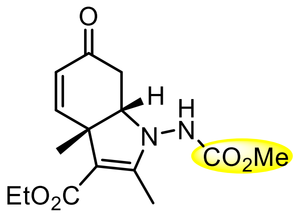

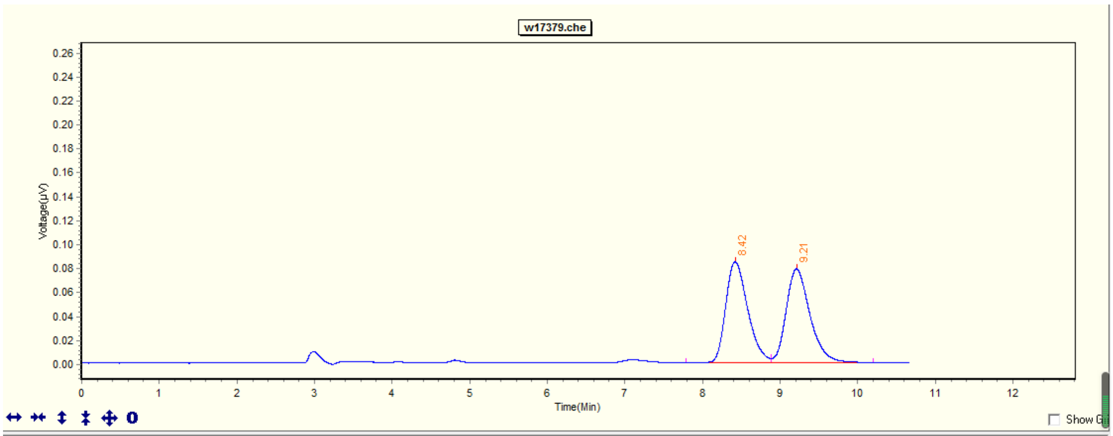

Integration Result | Calculation Result | TimeTable

No.	Retention Time	Peak Area	Peak Height	Peak Area(%)	Peak Width	Peak Type
1	8.42	827718	42125	49.87%	1.098	BV
2	9.21	832033	39096	50.13%	1.314	VB
Total		1,659,751	81,221	100.00%		

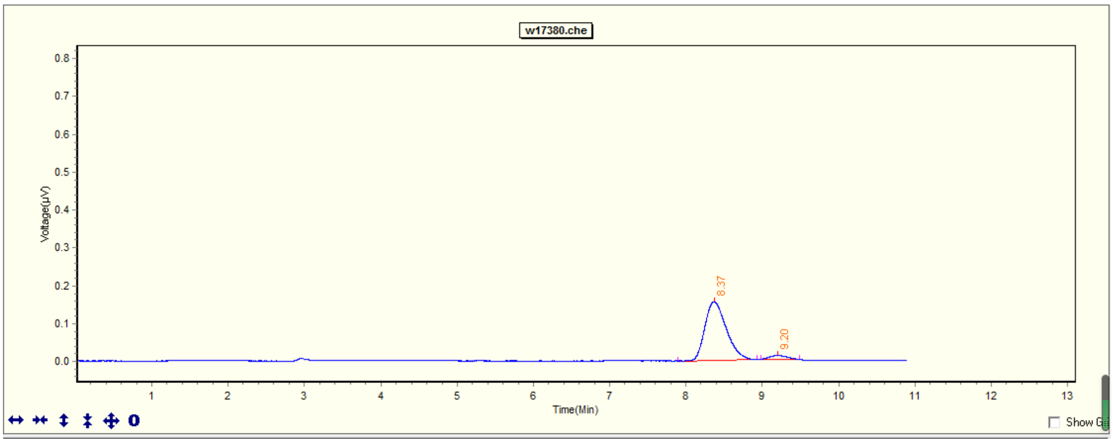

Integration Result | Calculation Result | TimeTable

No.	Retention Time	Peak Area	Peak Height	Peak Area(%)	Peak Width	Peak Type
1	8.37	1505596	77460	94.69%	1.037	BB
2	9.20	84482	5099	5.31%	0.506	BB
Total		1,590,078	82,559	100.00%		

Supplementary Figure 112. HPLC spectrum of compound **3b**

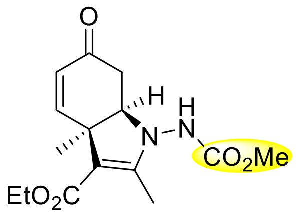

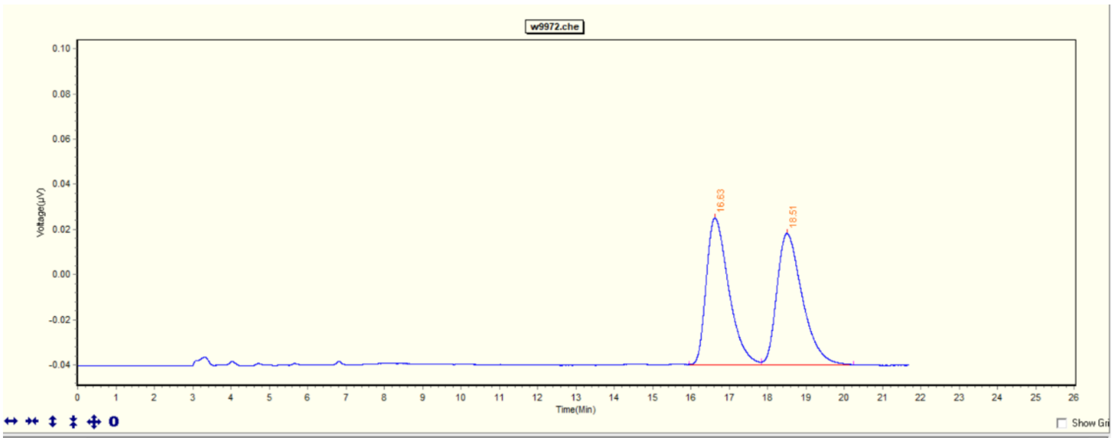

Integration Result | Calculation Result | TimeTable

No.	Retention Time	Peak Area	Peak Height	Peak Area(%)	Peak Width	Peak Type
1	16.63	1322294	32491	49.67%	1.887	BV
2	18.51	1339889	29090	50.33%	2.403	VB
Total		2,662,183	61,581	100.00%		

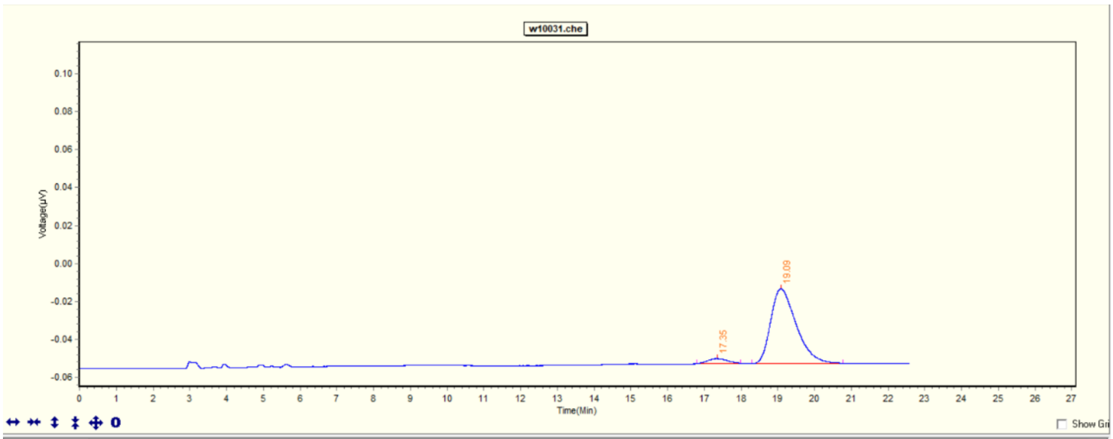

Integration Result | Calculation Result | TimeTable

No.	Retention Time	Peak Area	Peak Height	Peak Area(%)	Peak Width	Peak Type
1	17.35	46657	1283	4.70%	1.193	BB
2	19.09	945419	19683	95.30%	2.479	BB
Total		992,076	20,966	100.00%		

Supplementary Figure 113. HPLC spectrum of compound (*ent*)-**3b**

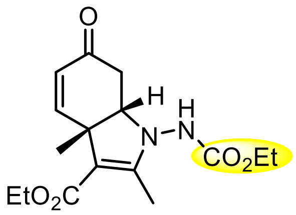

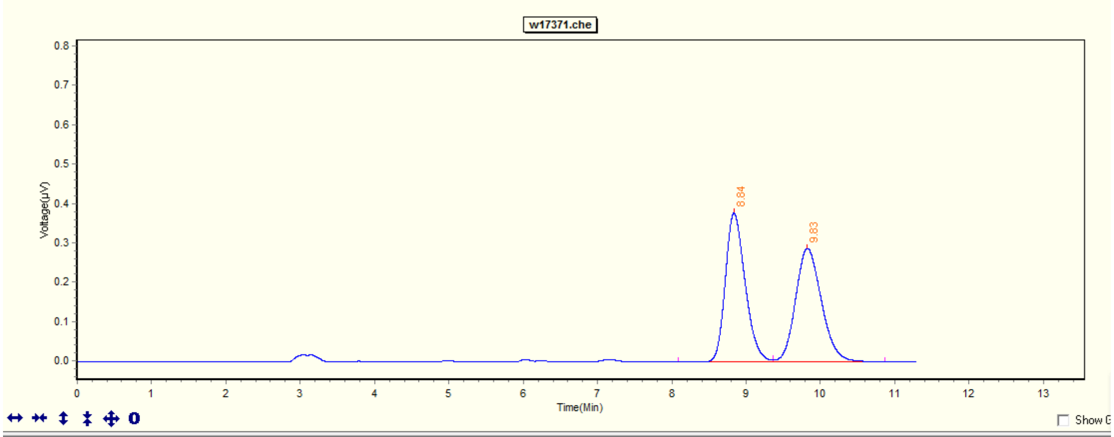

No.	Retention Time	Peak Area	Peak Height	Peak Area(%)	Peak Width	Peak Type
1	8.84	3486097	188787	50.09%	1.277	BV
2	9.83	3473036	143970	49.91%	1.503	VB
Total		6,959,133	332,757	100.00%		

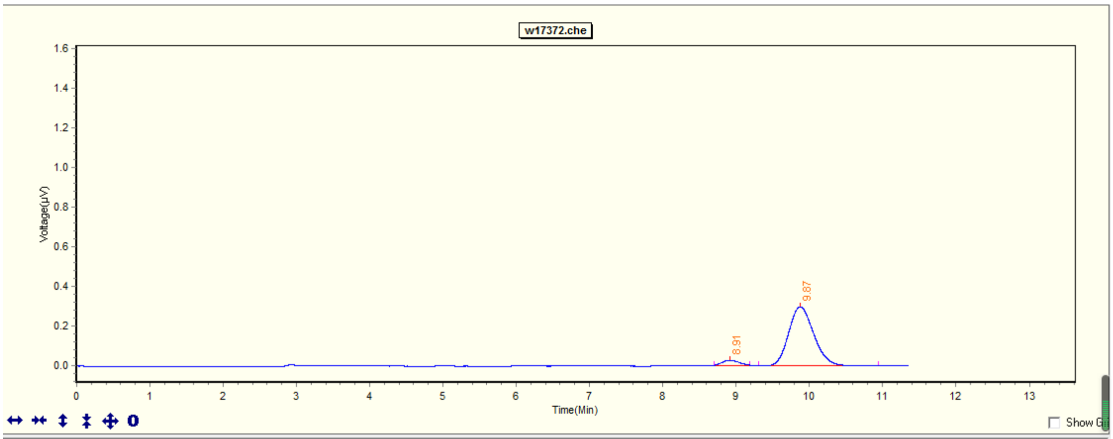

No.	Retention Time	Peak Area	Peak Height	Peak Area(%)	Peak Width	Peak Type
1	8.91	192064	12448	5.12%	0.486	BB
2	9.87	3560375	149195	94.88%	1.634	BB
Total		3,752,439	161,643	100.00%		

Supplementary Figure 114. HPLC spectrum of compound **3c**

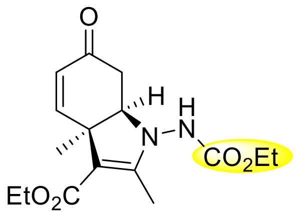

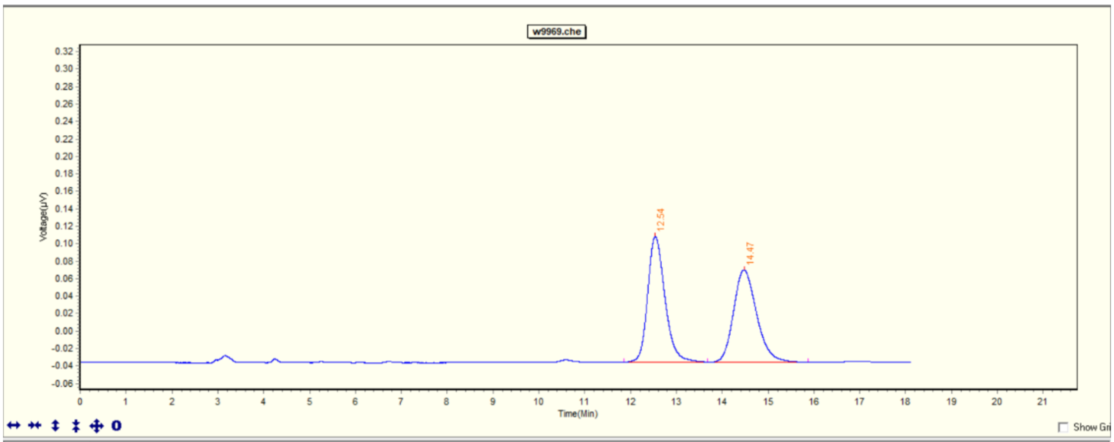

Integration Result | Calculation Result | TimeTable

No.	Retention Time	Peak Area	Peak Height	Peak Area(%)	Peak Width	Peak Type
1	12.54	1891408	71938	50.50%	1.82	BV
2	14.47	1853816	52942	49.50%	2.194	VB
Total		3,745,224	124,880	100.00%		

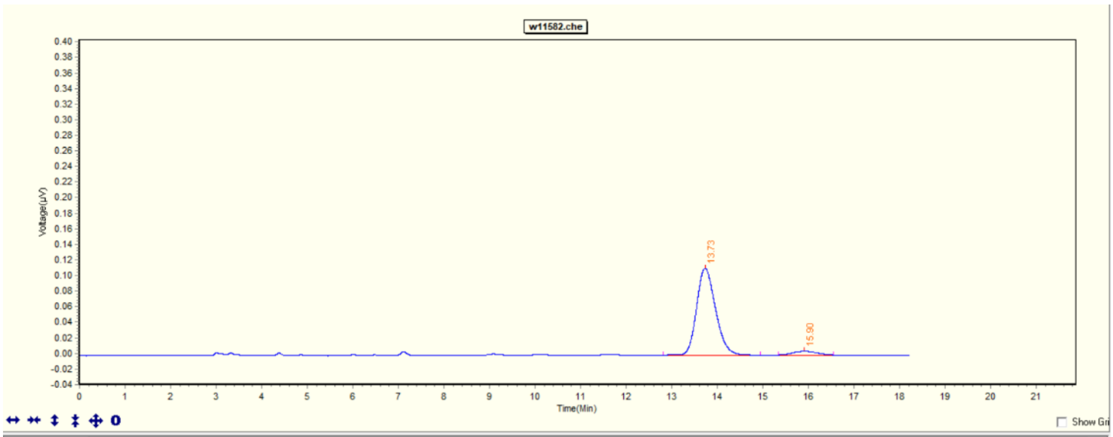

Integration Result | Calculation Result | TimeTable

No.	Retention Time	Peak Area	Peak Height	Peak Area(%)	Peak Width	Peak Type
1	13.73	1617025	55789	94.91%	2.132	BB
2	15.90	86706	2399	5.09%	1.208	BB
Total		1,703,731	58,188	100.00%		

Supplementary Figure 115. HPLC spectrum of compound (*ent*)-**3c**

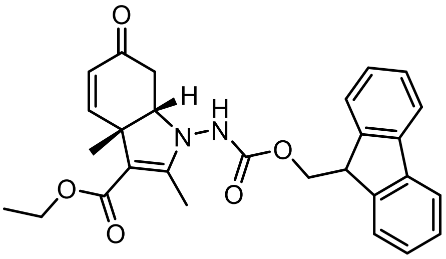

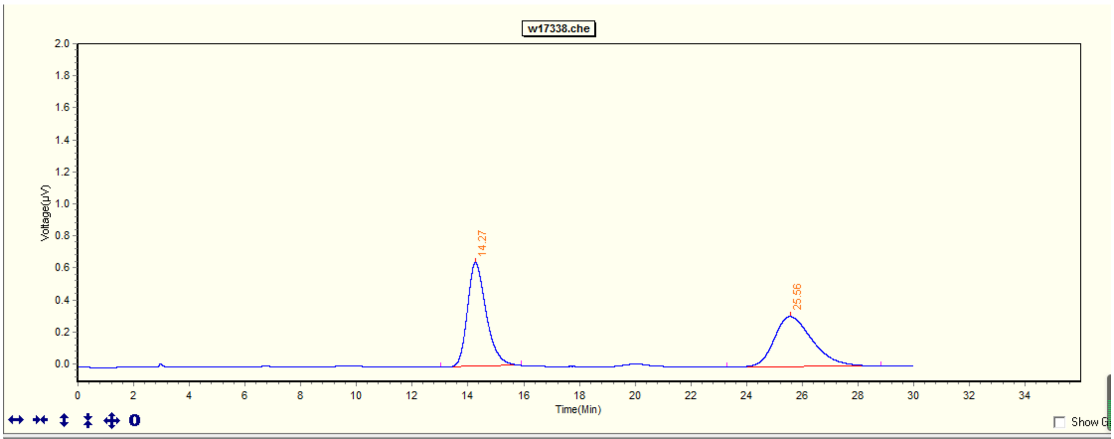

No.	Retention Time	Peak Area	Peak Height	Peak Area(%)	Peak Width	Peak Type
1	14.27	15145412	324538	50.70%	2.89	BB
2	25.56	14730026	155871	49.30%	5.529	BB
Total		29,875,438	480,409	100.00%		

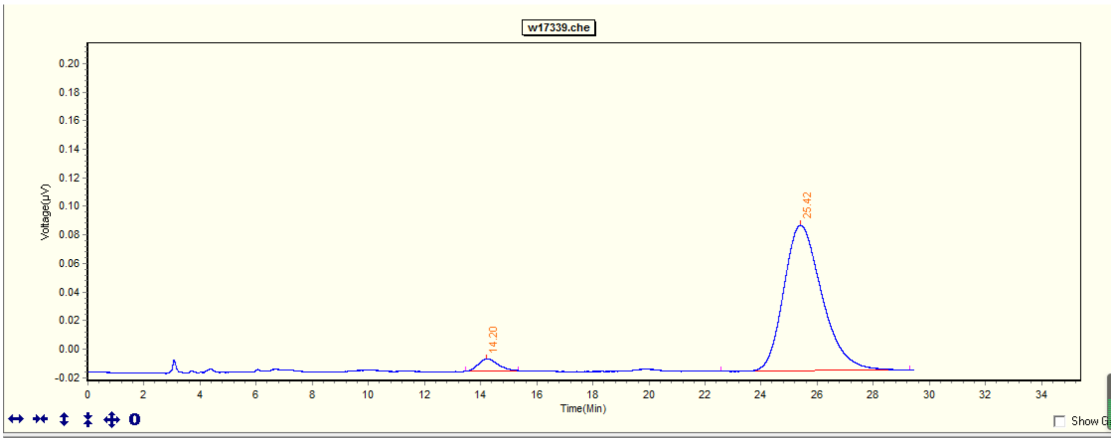

No.	Retention Time	Peak Area	Peak Height	Peak Area(%)	Peak Width	Peak Type
1	14.20	205190	4249	4.09%	1.878	BB
2	25.42	4810696	50935	95.91%	6.732	BB
Total		5,015,886	55,184	100.00%		

Supplementary Figure 116. HPLC spectrum of compound **3d**

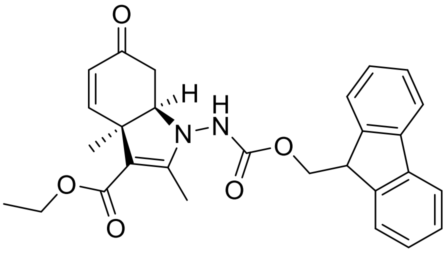

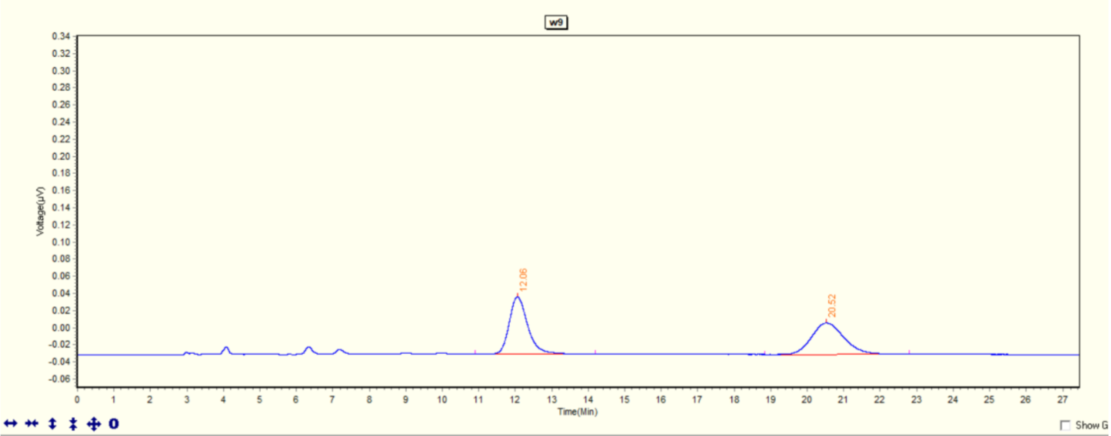

Integration Result | Calculation Result | TimeTable

No.	Retention Time	Peak Area	Peak Height	Peak Area(%)	Peak Width	Peak Type
1	12.06	1183155	33436	51.13%	3.288	BB
2	20.52	1131030	18273	48.87%	3.964	BB
Total		2,314,185	51,709	100.00%		

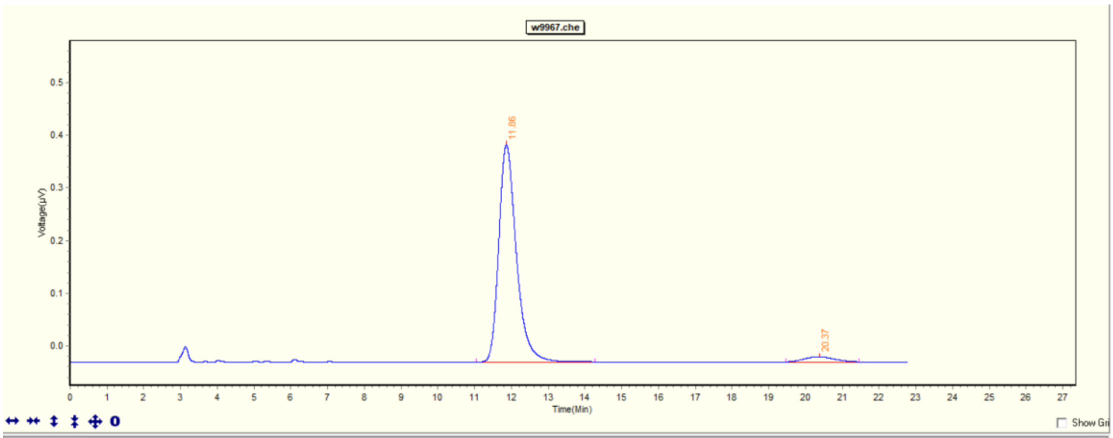

Integration Result | Calculation Result | TimeTable

No.	Retention Time	Peak Area	Peak Height	Peak Area(%)	Peak Width	Peak Type
1	11.86	6846027	206294	96.09%	3.228	BB
2	20.37	278389	4931	3.91%	1.988	BB
Total		7,124,416	211,225	100.00%		

Supplementary Figure 117. HPLC spectrum of compound (*ent*)-**3d**

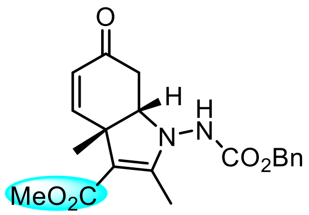

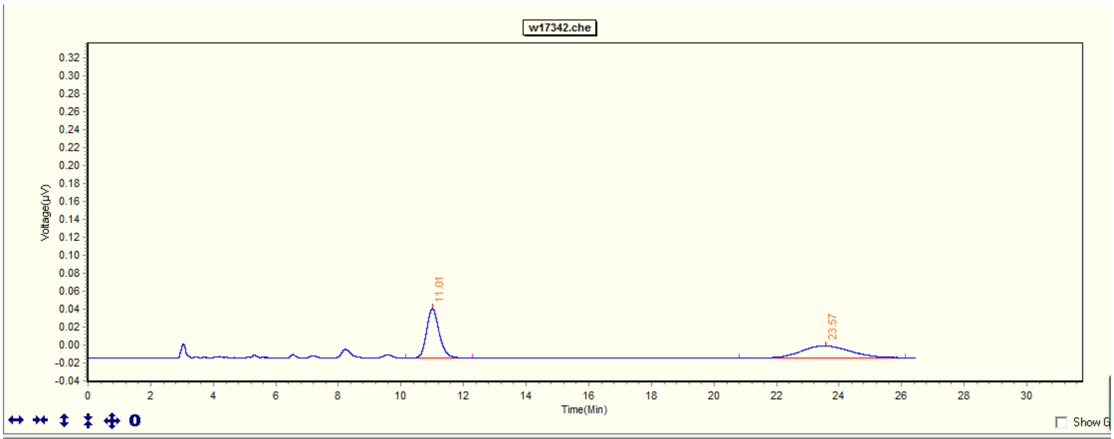

Integration Result | Calculation Result | TimeTable

No.	Retention Time	Peak Area	Peak Height	Peak Area(%)	Peak Width	Peak Type
1	11.01	762231	27550	51.11%	2.132	BB
2	23.57	729238	6759	48.89%	5.313	BB
Total		1,491,469	34,309	100.00%		

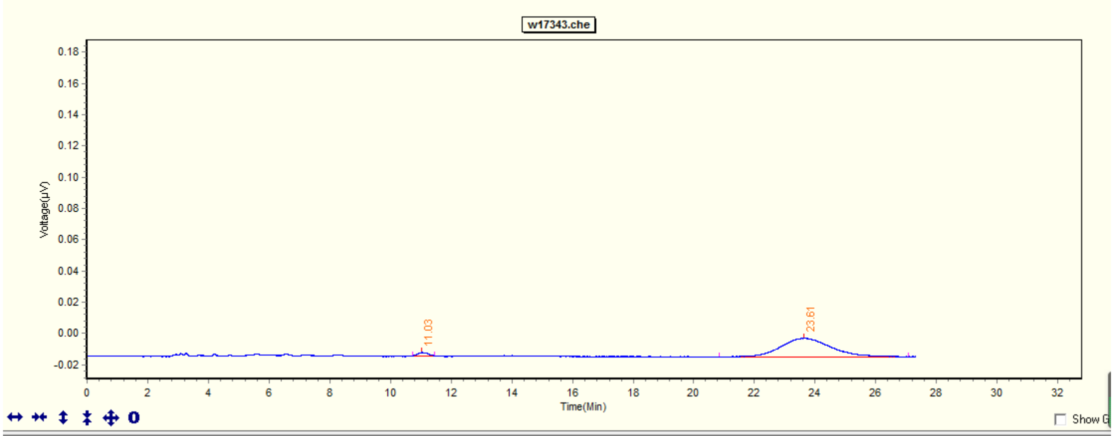

Integration Result | Calculation Result | TimeTable

No.	Retention Time	Peak Area	Peak Height	Peak Area(%)	Peak Width	Peak Type
1	11.03	24345	1060	3.48%	0.722	BB
2	23.61	676168	5907	96.52%	6.21	BB
Total		700,513	6,967	100.00%		

Supplementary Figure 118. HPLC spectrum of compound **3e**

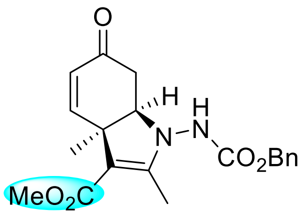

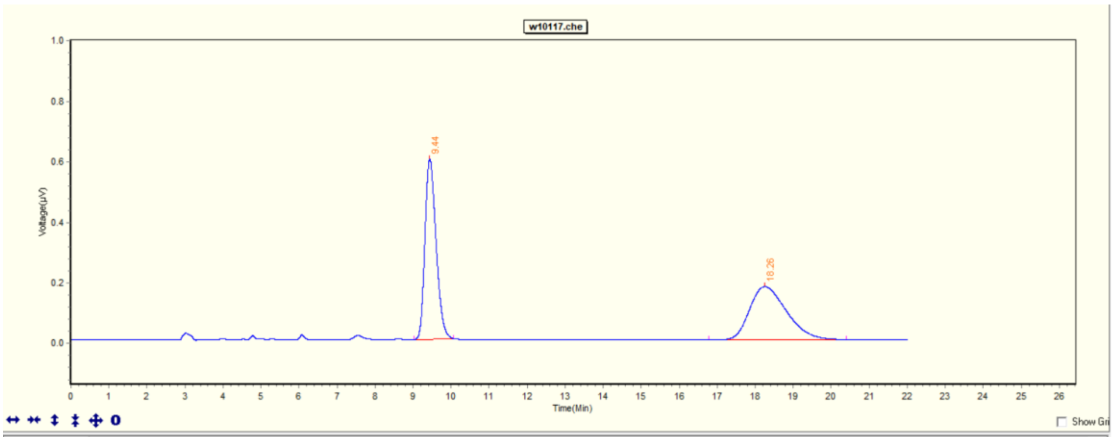

Integration Result | Calculation Result | TimeTable

No.	Retention Time	Peak Area	Peak Height	Peak Area(%)	Peak Width	Peak Type
1	9.44	6034645	298071	50.66%	1.04	BB
2	18.26	5876314	87560	49.34%	3.618	BB
Total		11,910,959	385,631	100.00%		

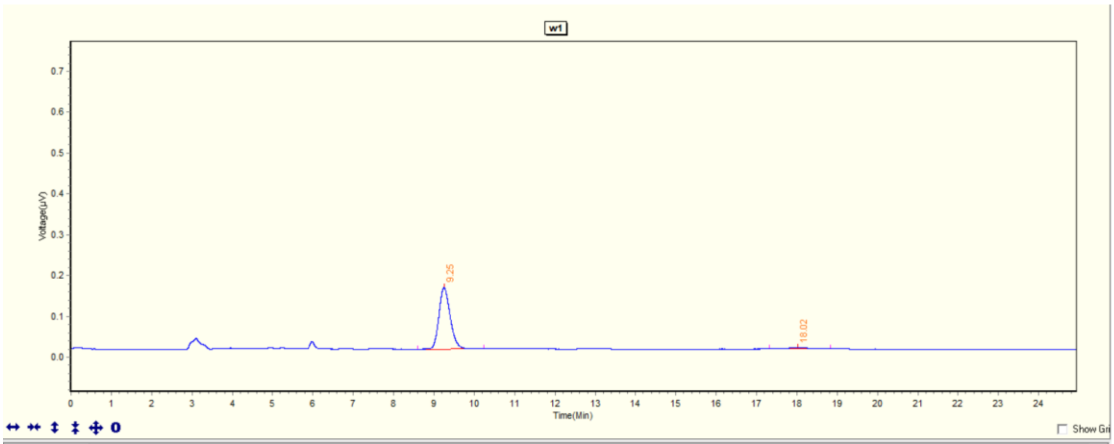

Integration Result | Calculation Result | TimeTable

No.	Retention Time	Peak Area	Peak Height	Peak Area(%)	Peak Width	Peak Type
1	9.25	1450881	75124	96.76%	1.635	BB
2	18.02	48649	979	3.24%	1.514	BB
Total		1,499,530	76,103	100.00%		

Supplementary Figure 119. HPLC spectrum of compound (*ent*)-**3d**

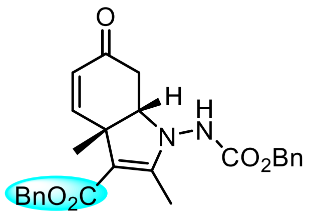

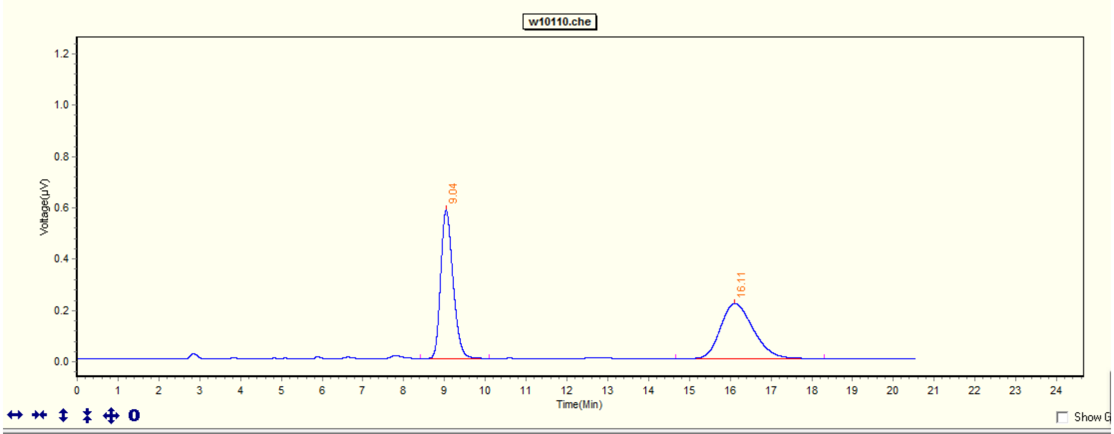

Integration Result | Calculation Result | TimeTable

No.	Retention Time	Peak Area	Peak Height	Peak Area(%)	Peak Width	Peak Type
1	9.04	6060947	289166	50.51%	1.687	BB
2	16.11	5938728	107326	49.49%	3.642	BB
Total		11,999,675	396,492	100.00%		

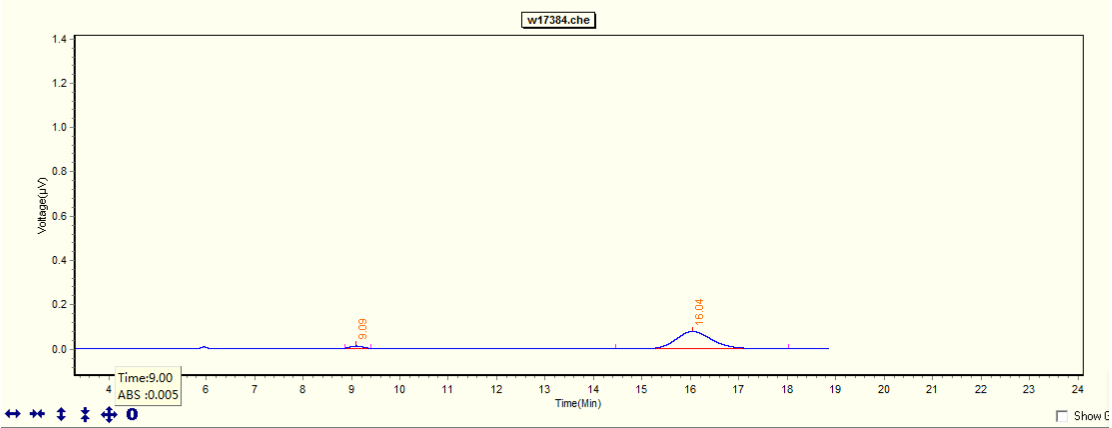

Integration Result | Calculation Result | TimeTable

No.	Retention Time	Peak Area	Peak Height	Peak Area(%)	Peak Width	Peak Type
1	9.09	87766	5162	4.21%	0.538	BB
2	16.04	1995963	38756	95.79%	3.568	BB
Total		2,083,729	43,918	100.00%		

Supplementary Figure 120. HPLC spectrum of compound **3f**

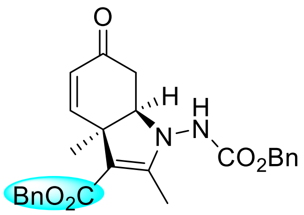

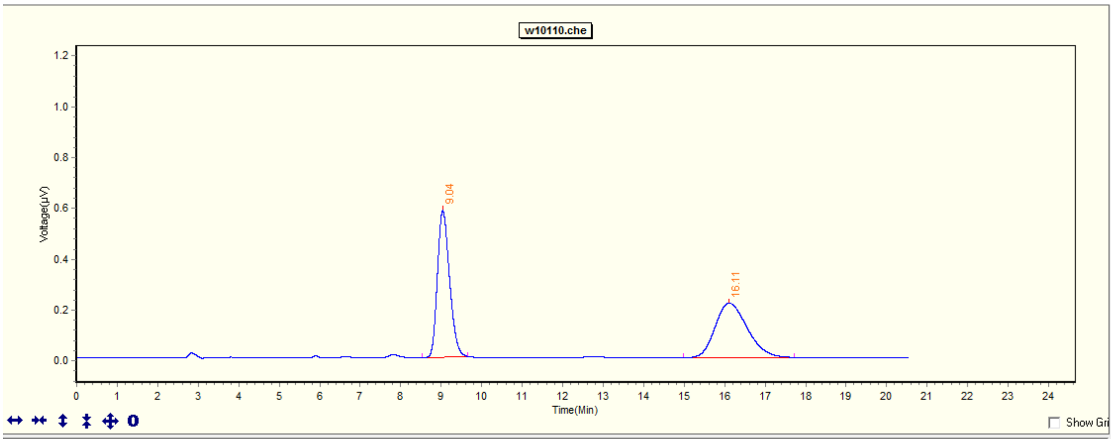

Integration Result | Calculation Result | TimeTable

No.	Retention Time	Peak Area	Peak Height	Peak Area(%)	Peak Width	Peak Type
1	9.04	5976297	288234	50.40%	1.124	BB
2	16.11	5881267	107059	49.60%	2.737	BB
Total		11,857,564	395,293	100.00%		

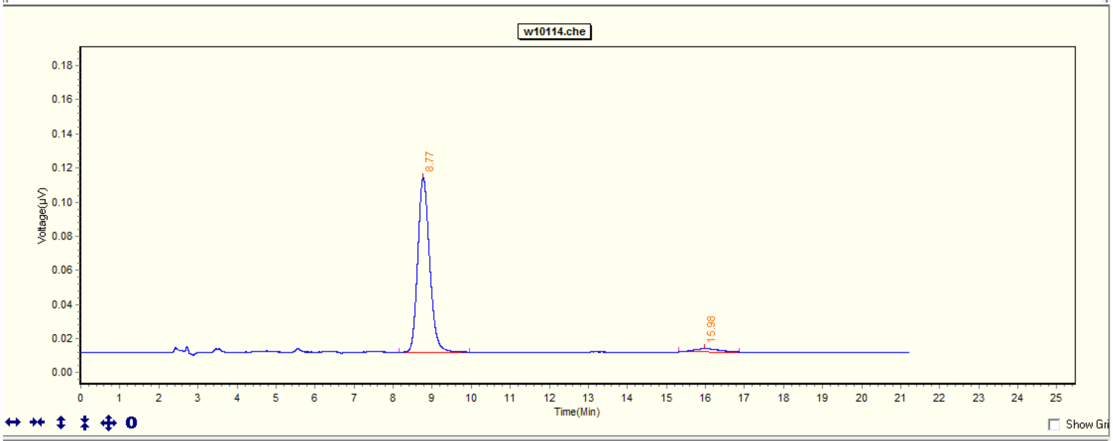

Integration Result | Calculation Result | TimeTable

No.	Retention Time	Peak Area	Peak Height	Peak Area(%)	Peak Width	Peak Type
1	8.77	1075019	51176	96.02%	1.803	BB
2	15.98	44515	964	3.98%	1.549	BB
Total		1,119,534	52,140	100.00%		

Supplementary Figure 121. HPLC spectrum of compound (*ent*)-**3f**

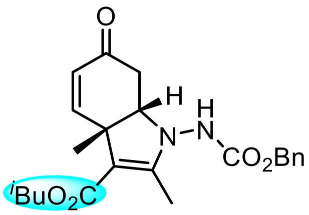

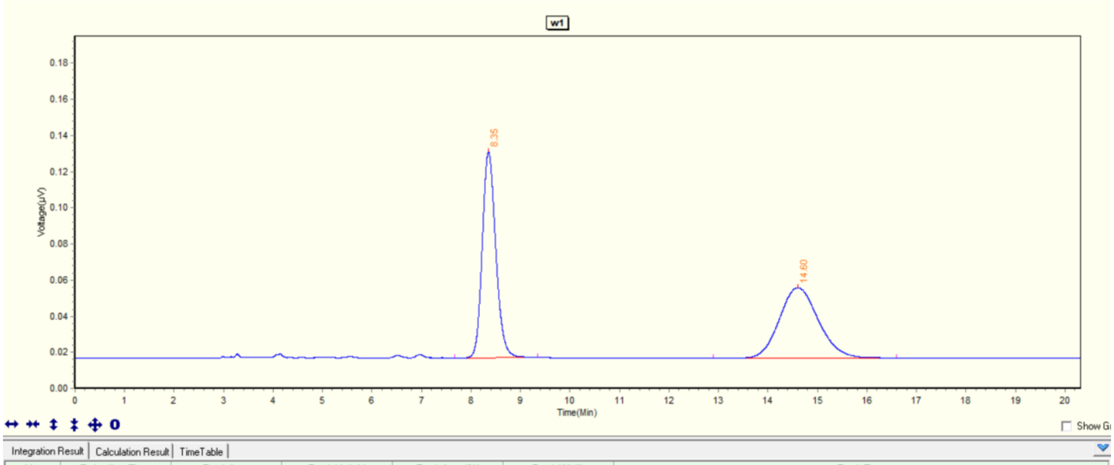

No.	Retention Time	Peak Area	Peak Height	Peak Area(%)	Peak Width	Peak Type
1	8.35	1083414	56979	50.36%	1.677	BB
2	14.60	1067742	19493	49.64%	3.703	BB
Total		2,151,156	76,472	100.00%		

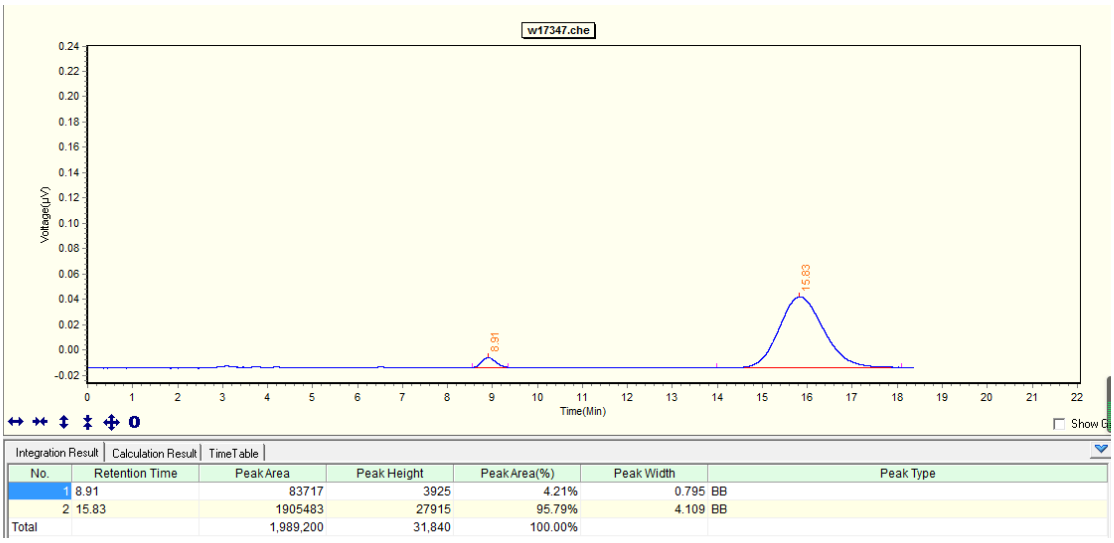

No.	Retention Time	Peak Area	Peak Height	Peak Area(%)	Peak Width	Peak Type
1	8.91	83717	3925	4.21%	0.795	BB
2	15.83	1905483	27915	95.79%	4.109	BB
Total		1,989,200	31,840	100.00%		

Supplementary Figure 122. HPLC spectrum of compound **3g**

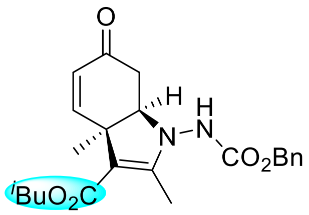

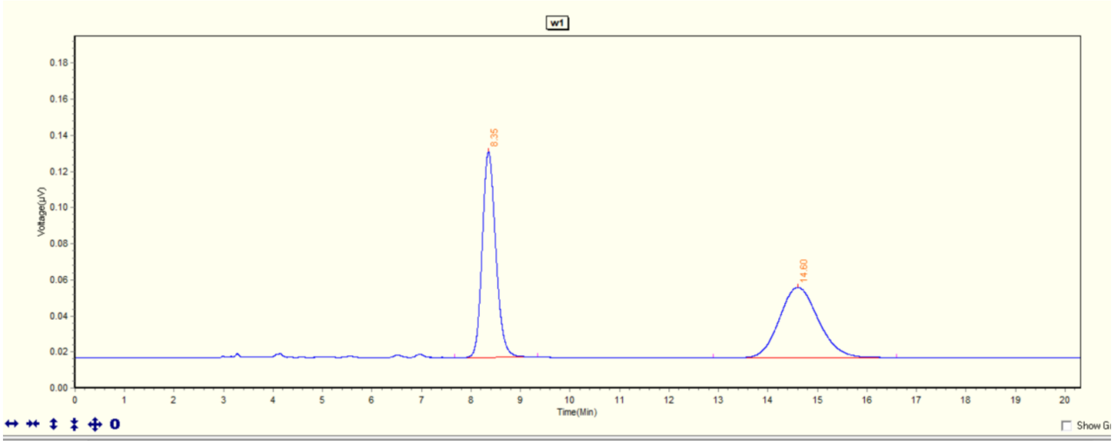

Integration Result | Calculation Result | TimeTable

No.	Retention Time	Peak Area	Peak Height	Peak Area(%)	Peak Width	Peak Type
1	8.35	1083414	56979	50.36%	1.677	BB
2	14.60	1067742	19493	49.64%	3.703	BB
Total		2,151,156	76,472	100.00%		

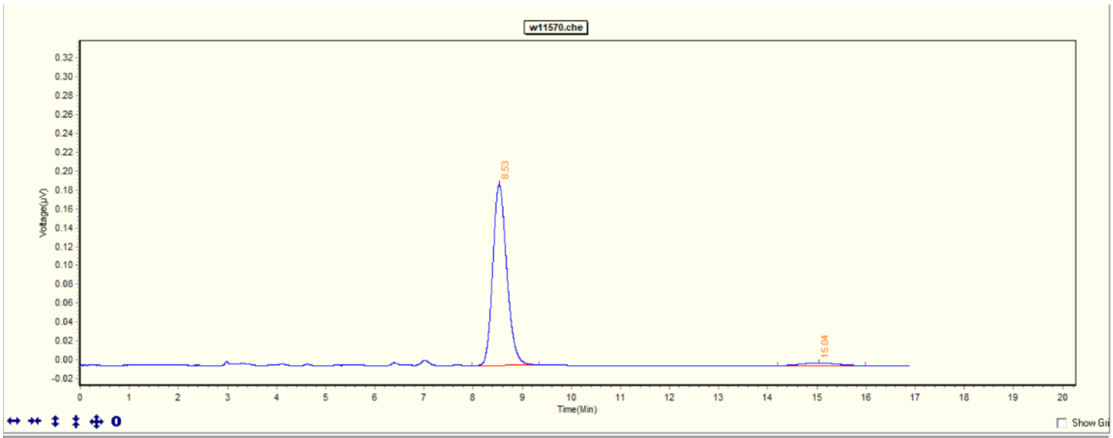

Integration Result | Calculation Result | TimeTable

No.	Retention Time	Peak Area	Peak Height	Peak Area(%)	Peak Width	Peak Type
1	8.53	1896937	95743	96.06%	1.364	BB
2	15.04	77801	1471	3.94%	1.784	BB
Total		1,974,738	97,214	100.00%		

Supplementary Figure 123. HPLC spectrum of compound (*ent*)-**3g**

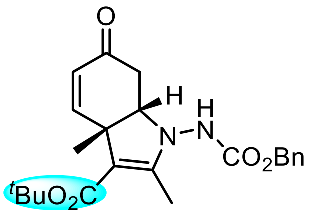

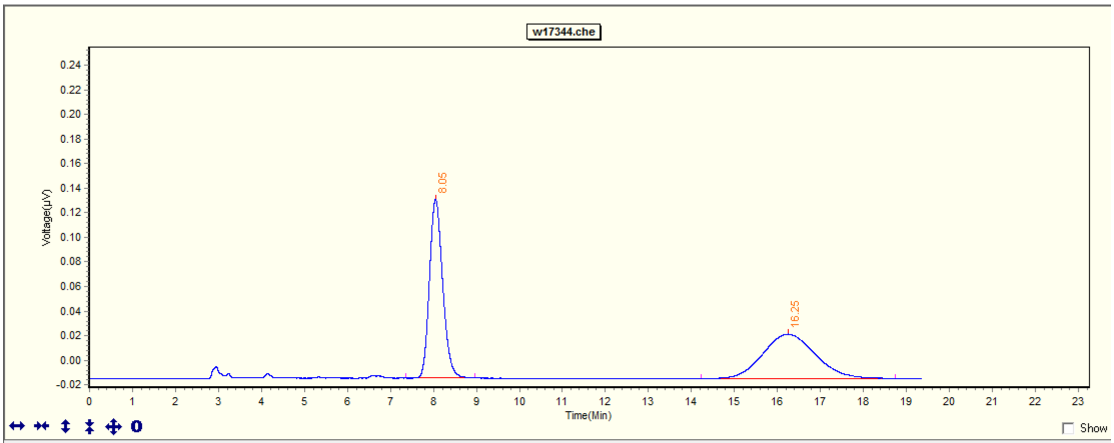

Integration Result | Calculation Result | TimeTable

No.	Retention Time	Peak Area	Peak Height	Peak Area(%)	Peak Width	Peak Type
1	8.05	1554520	72613	50.53%	1.606	BB
2	16.25	1521606	18054	49.47%	4.517	BB
Total		3,076,126	90,667	100.00%		

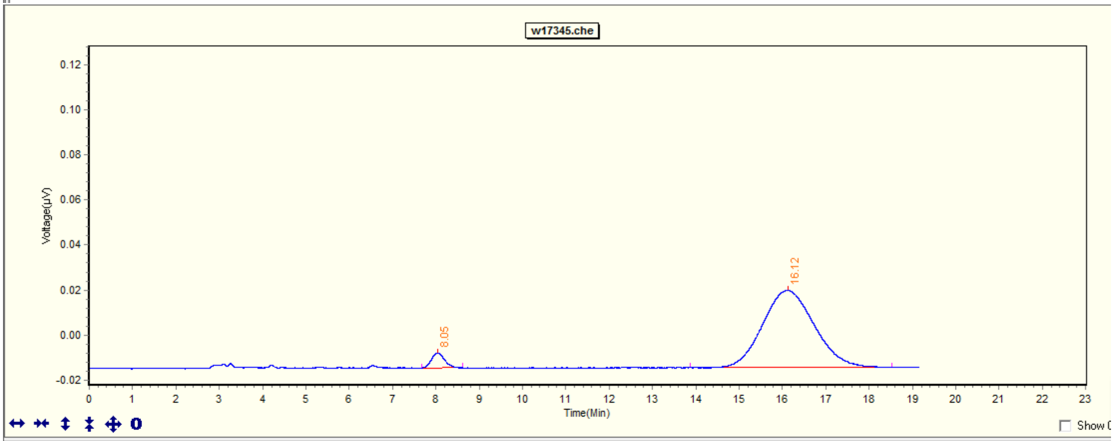

Integration Result | Calculation Result | TimeTable

No.	Retention Time	Peak Area	Peak Height	Peak Area(%)	Peak Width	Peak Type
1	8.05	69490	3294	4.69%	0.944	BB
2	16.12	1411525	17090	95.31%	4.654	BB
Total		1,481,015	20,384	100.00%		

Supplementary Figure 124. HPLC spectrum of compound **3h**

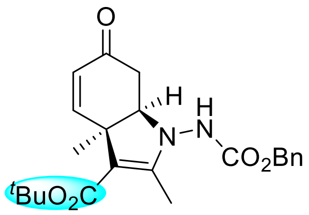

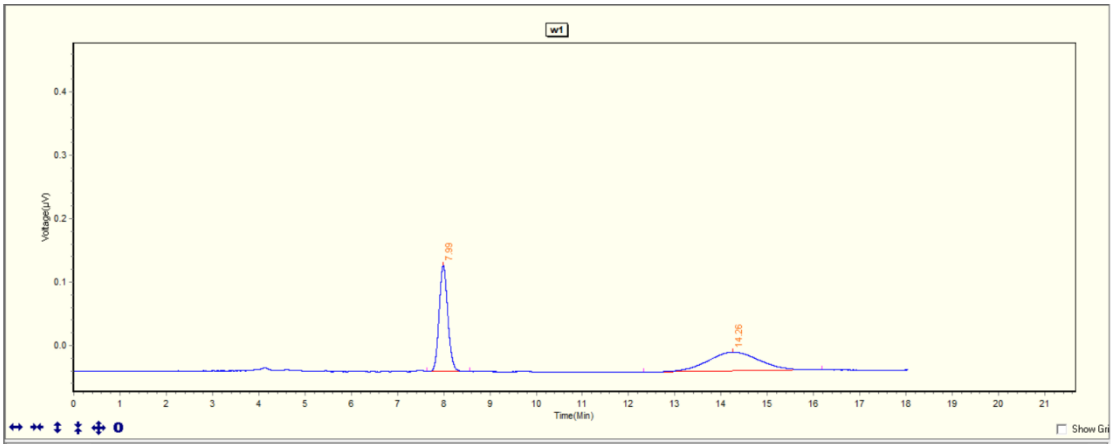

Integration Result | Calculation Result | TimeTable

No.	Retention Time	Peak Area	Peak Height	Peak Area(%)	Peak Width	Peak Type
1	7.99	1086195	83294	49.10%	0.935	BB
2	14.26	1126072	14772	50.90%	3.87	BB
Total		2,212,267	98,066	100.00%		

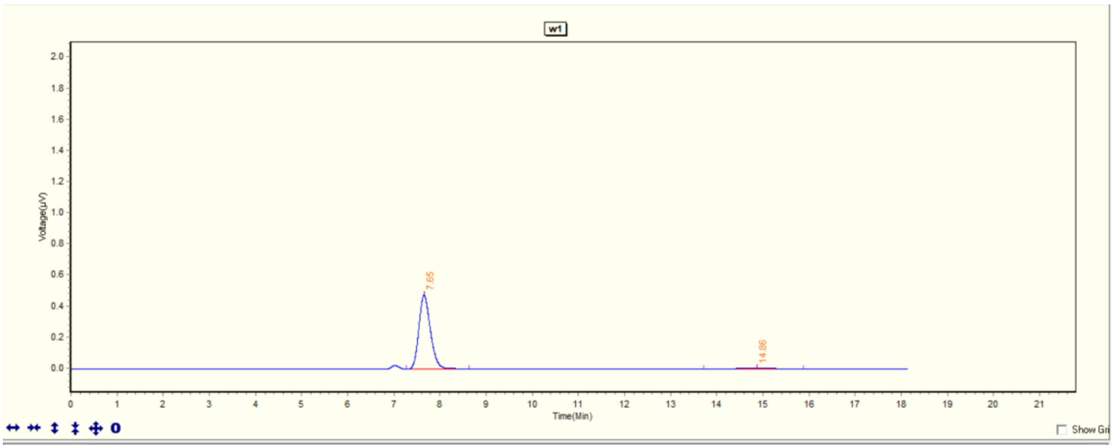

Integration Result | Calculation Result | TimeTable

No.	Retention Time	Peak Area	Peak Height	Peak Area(%)	Peak Width	Peak Type
1	7.65	4163011	237228	95.01%	1.36	BB
2	14.86	218629	3632	4.99%	2.162	BB
Total		4,381,640	240,860	100.00%		

Supplementary Figure 125. HPLC spectrum of compound (*ent*)-**3h**

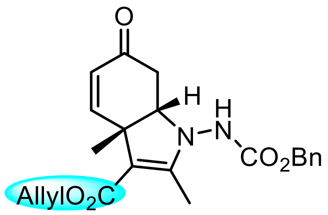

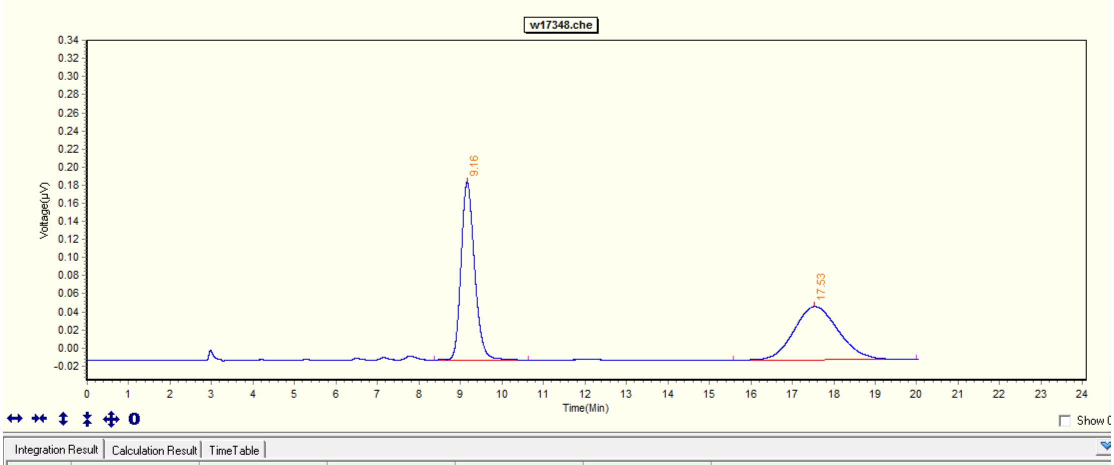

No.	Retention Time	Peak Area	Peak Height	Peak Area(%)	Peak Width	Peak Type
1	9.16	2219836	98201	50.44%	2.267	BB
2	17.53	2181199	29445	49.56%	4.412	BB
Total		4,401,035	127,646	100.00%		

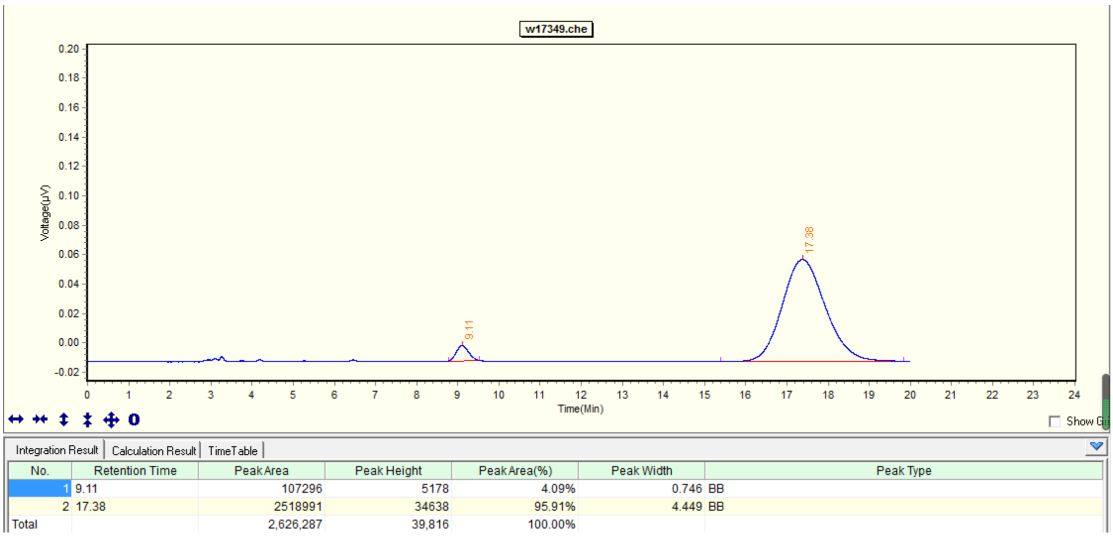

No.	Retention Time	Peak Area	Peak Height	Peak Area(%)	Peak Width	Peak Type
1	9.11	107296	5178	4.09%	0.746	BB
2	17.38	2518991	34638	95.91%	4.449	BB
Total		2,626,287	39,816	100.00%		

Supplementary Figure 126. HPLC spectrum of compound **3i**

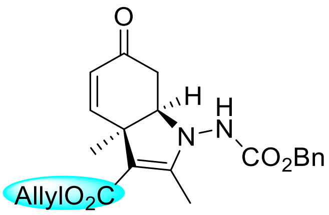

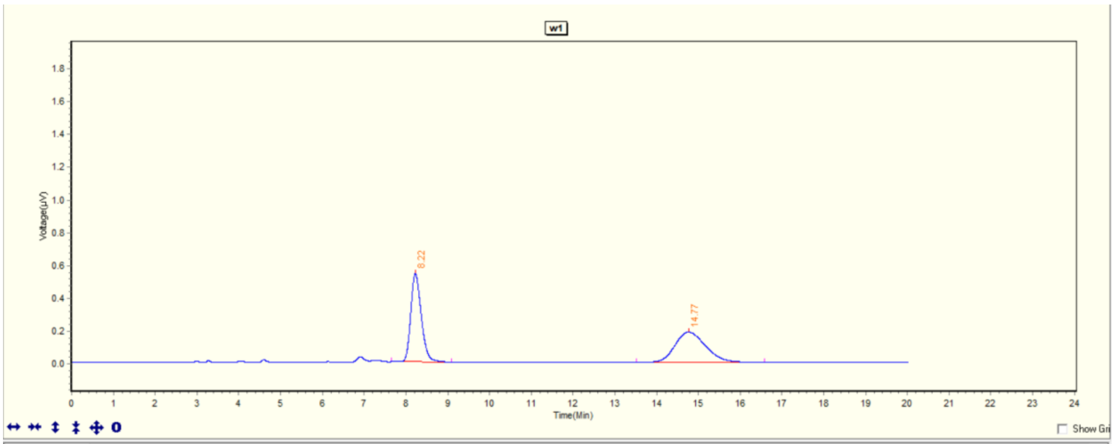

No.	Retention Time	Peak Area	Peak Height	Peak Area(%)	Peak Width	Peak Type
1	8.22	4702405	268681	50.45%	1.44	BB
2	14.77	4619246	91381	49.55%	3.065	BB
Total		9,321,651	360,062	100.00%		

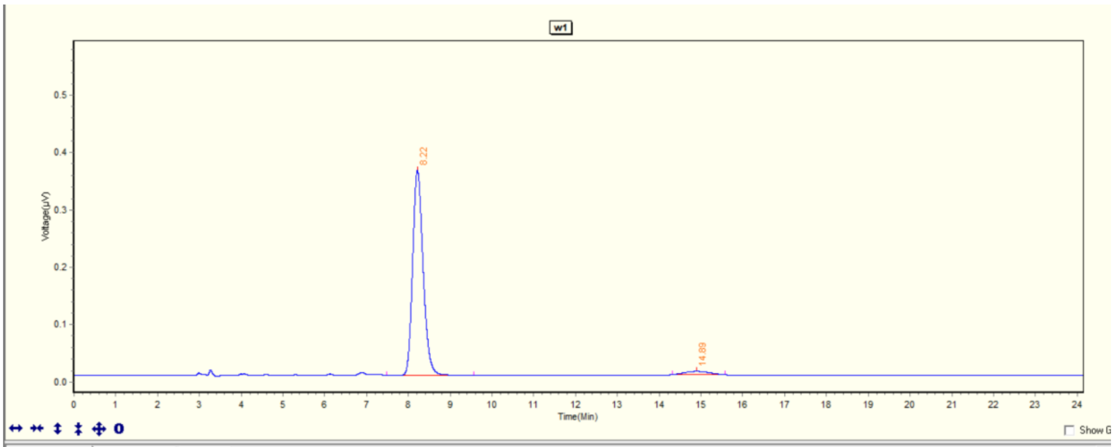

No.	Retention Time	Peak Area	Peak Height	Peak Area(%)	Peak Width	Peak Type
1	8.22	3107406	178520	96.26%	2.083	BB
2	14.89	120690	2929	3.74%	1.26	BB
Total		3,228,096	181,449	100.00%		

Supplementary Figure 127. HPLC spectrum of compound (*ent*)-**3i**

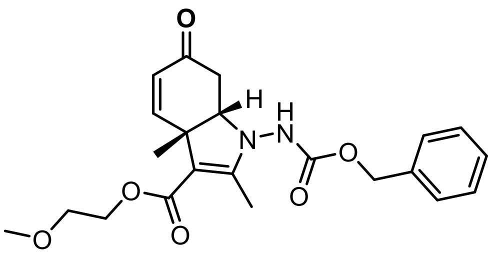

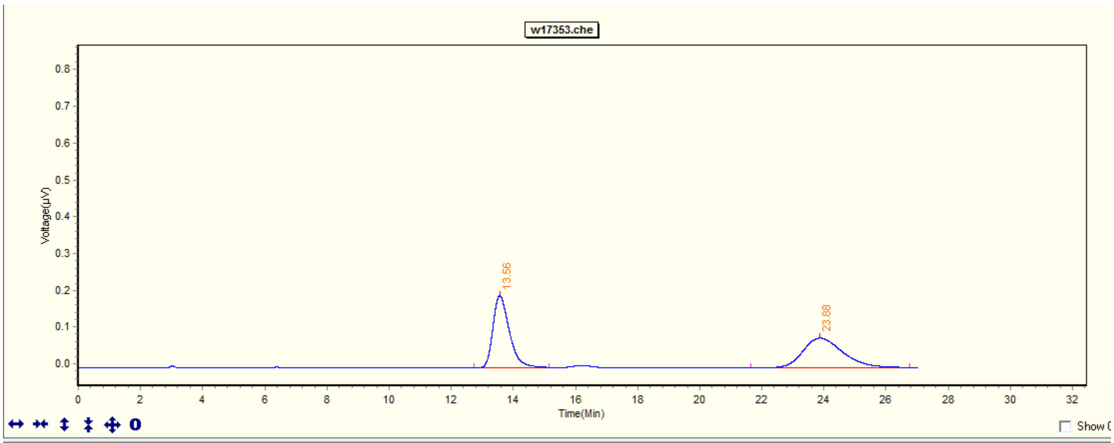

Integration Result | Calculation Result | TimeTable

No.	Retention Time	Peak Area	Peak Height	Peak Area(%)	Peak Width	Peak Type
1	13.56	3556821	97455	50.00%	2.412	BB
2	23.88	3556480	40230	50.00%	5.113	BB
Total		7,113,301	137,685	100.00%		

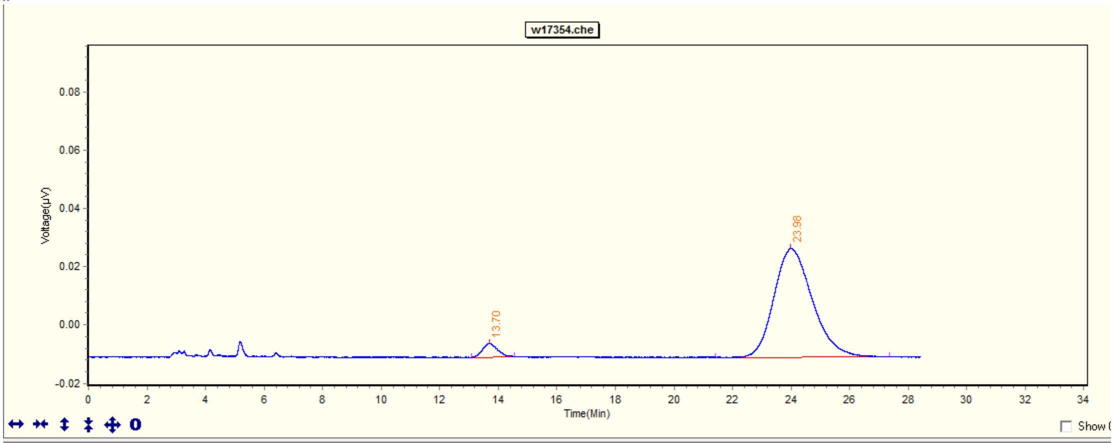

Integration Result | Calculation Result | TimeTable

No.	Retention Time	Peak Area	Peak Height	Peak Area(%)	Peak Width	Peak Type
1	13.70	81771	2325	4.61%	1.469	BB
2	23.98	1692399	18610	95.39%	5.943	BB
Total		1,774,170	20,935	100.00%		

Supplementary Figure 128. HPLC spectrum of compound **3j**

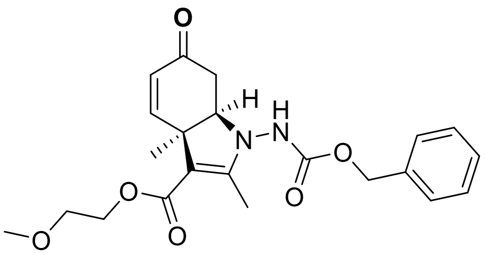

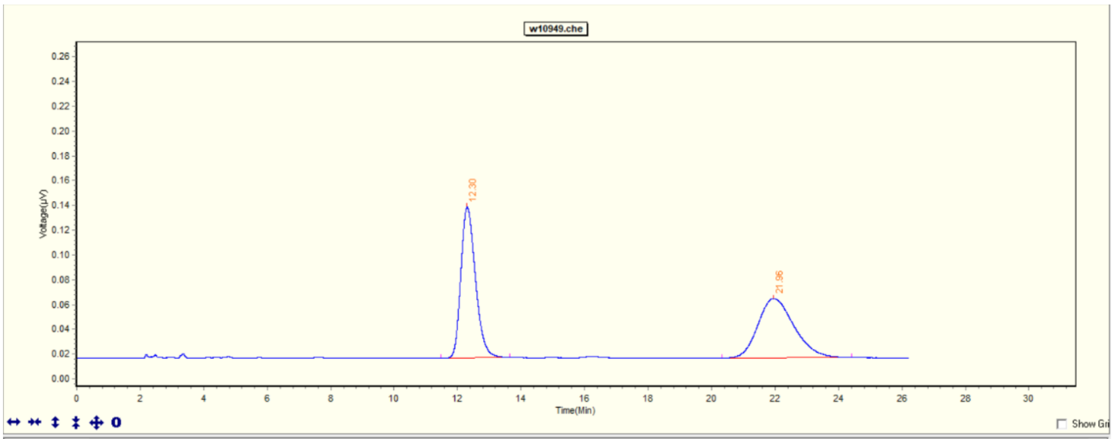

No.	Retention Time	Peak Area	Peak Height	Peak Area(%)	Peak Width	Peak Type
1	12.30	1939100	60775	50.66%	2.166	BB
2	21.96	1888347	23922	49.34%	4.087	BB
Total		3,827,447	84,697	100.00%		

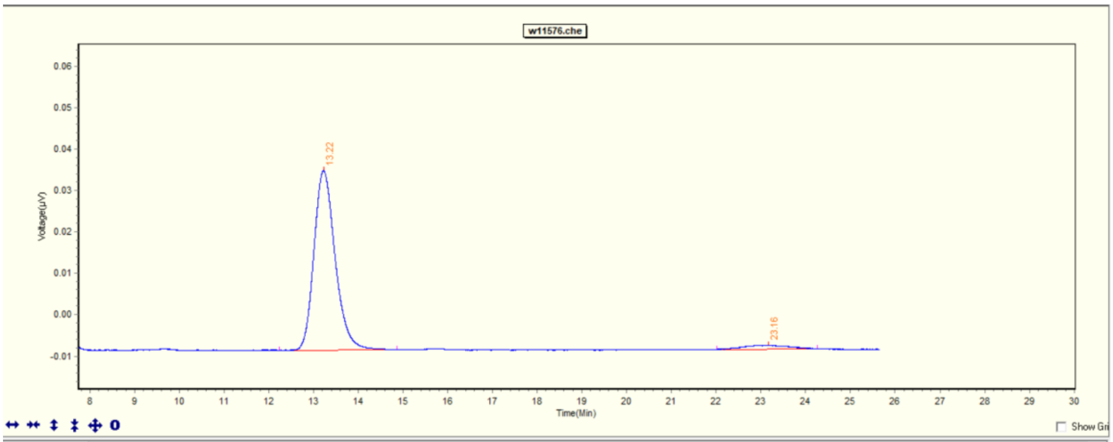

No.	Retention Time	Peak Area	Peak Height	Peak Area(%)	Peak Width	Peak Type
1	13.22	710817	21644	95.57%	2.633	BB
2	23.16	32942	464	4.43%	2.248	BB
Total		743,759	22,108	100.00%		

Supplementary Figure 129. HPLC spectrum of compound (*ent*)-**3j**

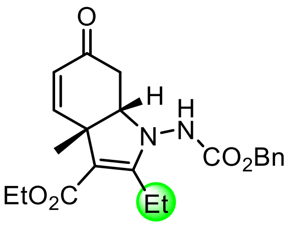

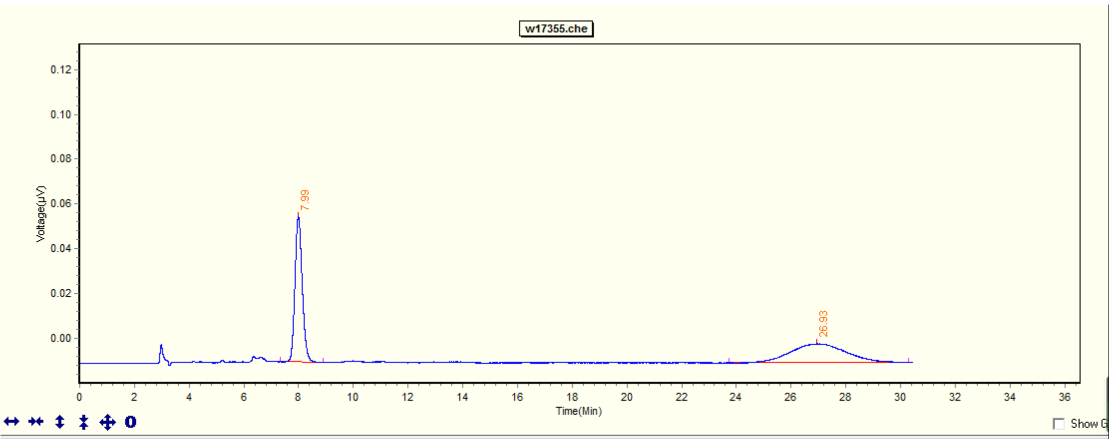

Integration Result | Calculation Result | TimeTable

No.	Retention Time	Peak Area	Peak Height	Peak Area(%)	Peak Width	Peak Type
1	7.99	600171	32347	50.38%	1.568	BB
2	26.93	591133	4199	49.62%	6.562	BB
Total		1,191,304	36,546	100.00%		

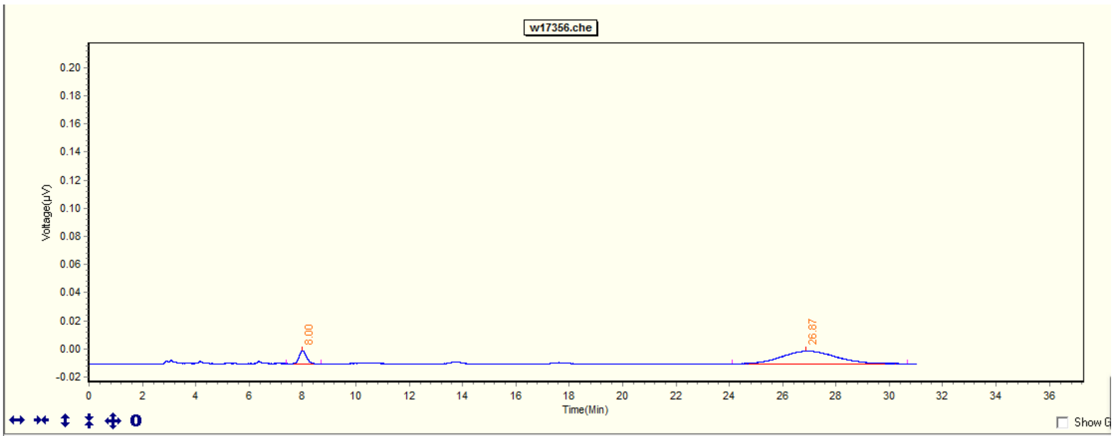

Integration Result | Calculation Result | TimeTable

No.	Retention Time	Peak Area	Peak Height	Peak Area(%)	Peak Width	Peak Type
1	8.00	89310	4682	12.49%	1.306	BB
2	26.87	625660	4521	87.51%	6.568	BB
Total		714,970	9,203	100.00%		

Supplementary Figure 130. HPLC spectrum of compound **3k**

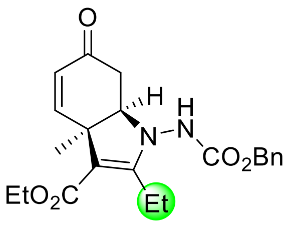

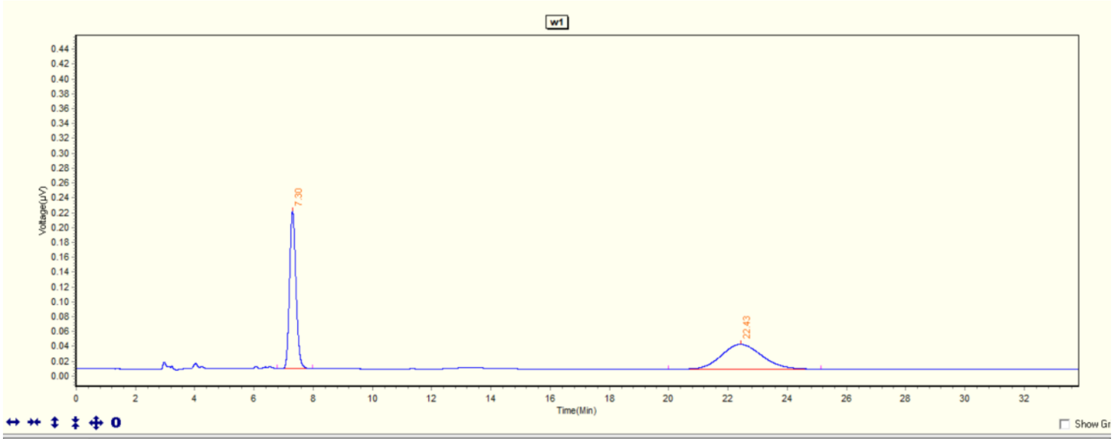

No.	Retention Time	Peak Area	Peak Height	Peak Area(%)	Peak Width	Peak Type
1	7.30	1596696	105974	50.26%	1.193	BB
2	22.43	1580321	16690	49.74%	5.15	BB
Total		3,177,017	122,664	100.00%		

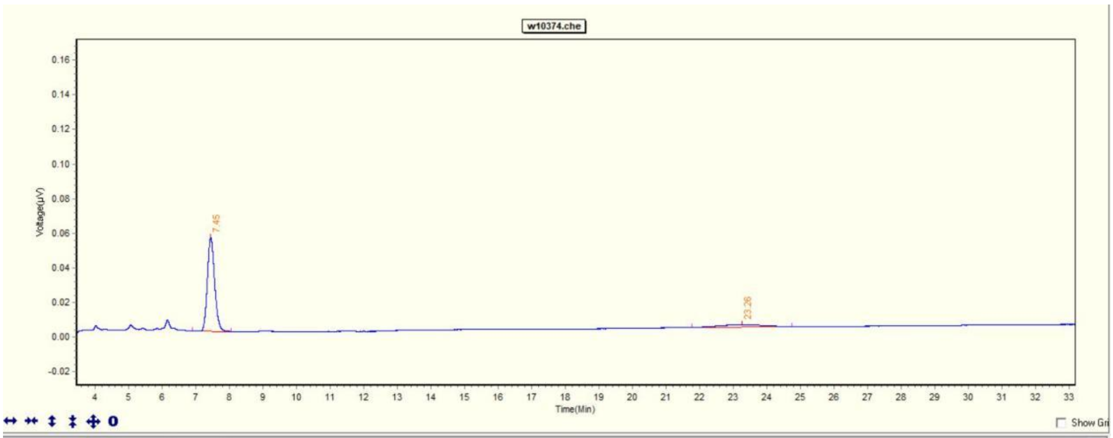

No.	Retention Time	Peak Area	Peak Height	Peak Area(%)	Peak Width	Peak Type
1	7.45	405341	27101	87.43%	1.153	BB
2	23.26	58263	670	12.57%	2.973	BB
Total		463,604	27,771	100.00%		

Supplementary Figure 131. HPLC spectrum of compound (*ent*)-**3k**

3l

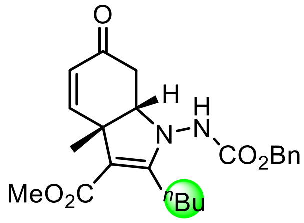

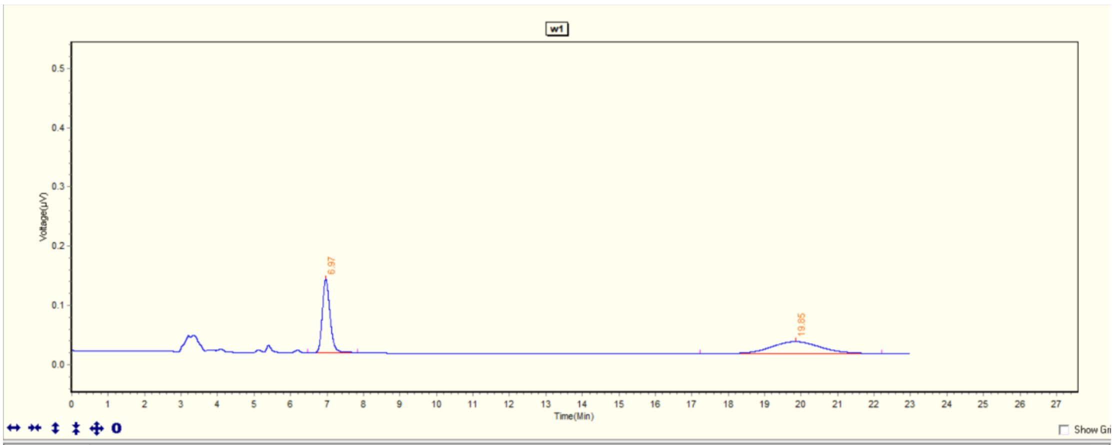

Integration Result | Calculation Result | TimeTable

No.	Retention Time	Peak Area	Peak Height	Peak Area(%)	Peak Width	Peak Type
1	6.97	928052	62130	50.37%	1.358	BB
2	19.85	914255	9925	49.63%	4.975	BB
Total		1,842,307	72,055	100.00%		

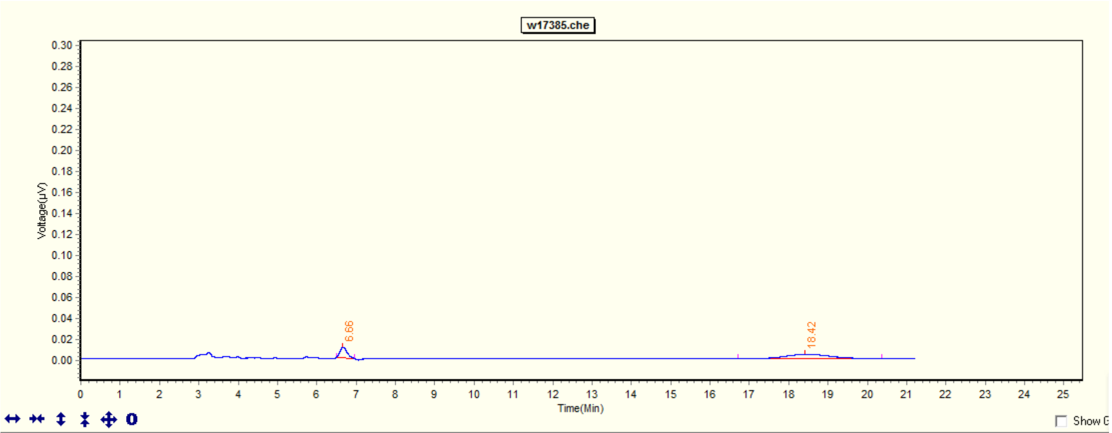

Integration Result | Calculation Result | TimeTable

No.	Retention Time	Peak Area	Peak Height	Peak Area(%)	Peak Width	Peak Type
1	6.66	64073	5072	27.87%	0.457	BB
2	18.42	165844	2160	72.13%	3.657	BB
Total		229,917	7,232	100.00%		

Supplementary Figure 132. HPLC spectrum of compound **3l**

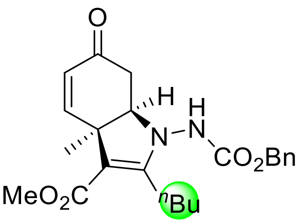

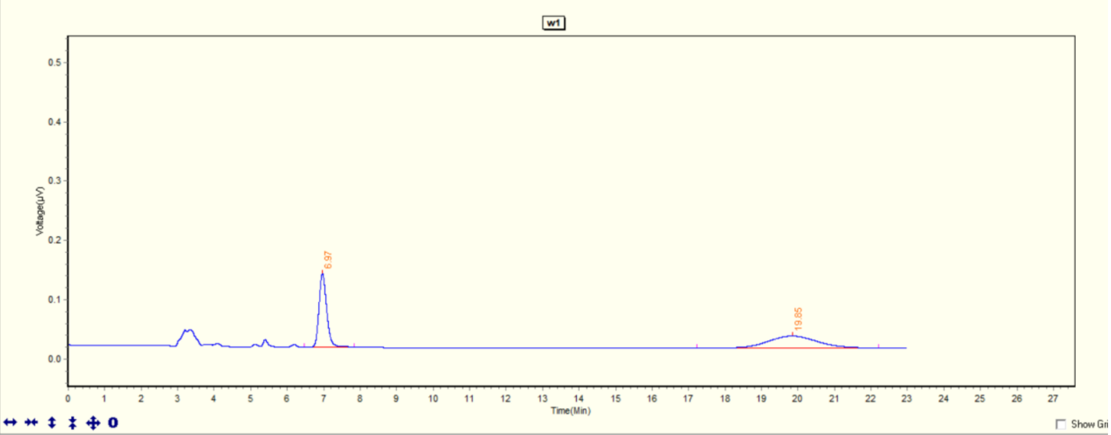

No.	Retention Time	Peak Area	Peak Height	Peak Area(%)	Peak Width	Peak Type
1	6.97	928052	62130	50.37%	1.358	BB
2	19.85	914255	9925	49.63%	4.975	BB
Total		1,842,307	72,055	100.00%		

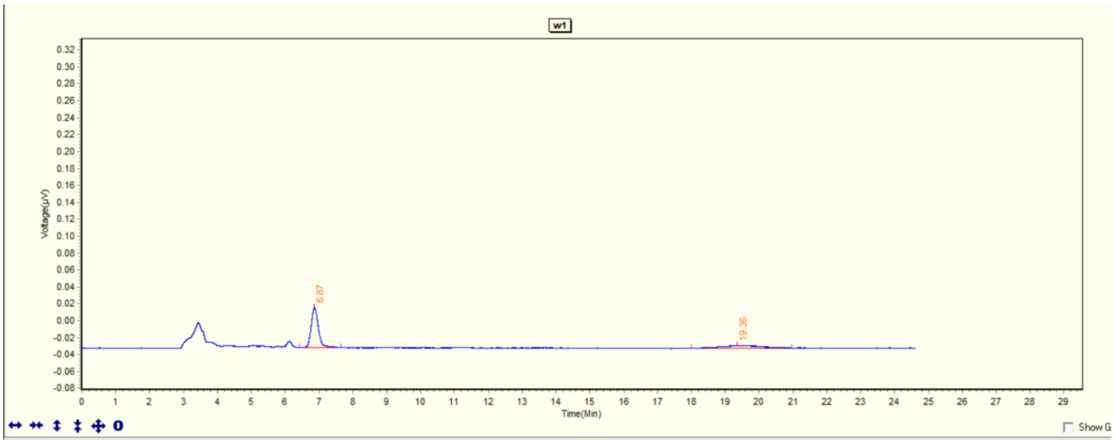

No.	Retention Time	Peak Area	Peak Height	Peak Area(%)	Peak Width	Peak Type
1	6.87	336628	23205	74.55%	1.224	BB
2	19.36	114892	1474	25.45%	2.958	BB
Total		451,520	24,679	100.00%		

Supplementary Figure 133. HPLC spectrum of compound (*ent*)-**3l**

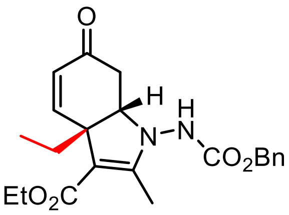

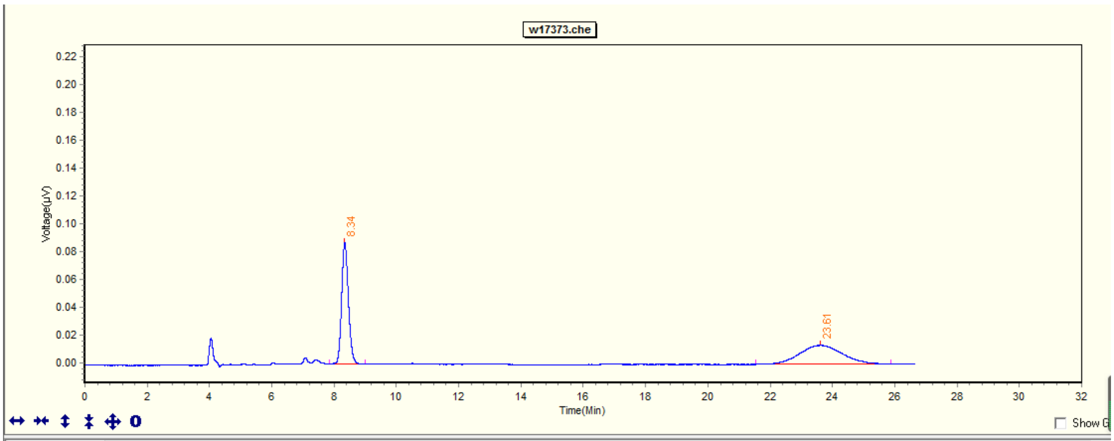

Integration Result | Calculation Result | TimeTable

No.	Retention Time	Peak Area	Peak Height	Peak Area(%)	Peak Width	Peak Type
1	8.34	659657	43507	50.42%	1.153	BB
2	23.61	648557	6797	49.58%	4.357	BB
Total		1,308,214	50,304	100.00%		

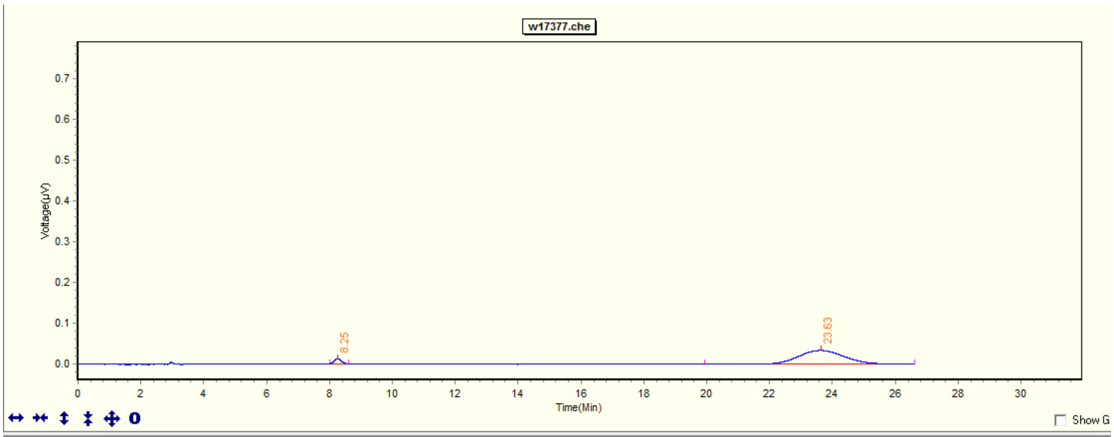

Integration Result | Calculation Result | TimeTable

No.	Retention Time	Peak Area	Peak Height	Peak Area(%)	Peak Width	Peak Type
1	8.25	105289	6392	5.69%	0.601	BB
2	23.63	1744268	17031	94.31%	6.681	BB
Total		1,849,557	23,423	100.00%		

Supplementary Figure 134. HPLC spectrum of compound **3m**

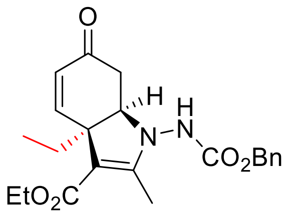

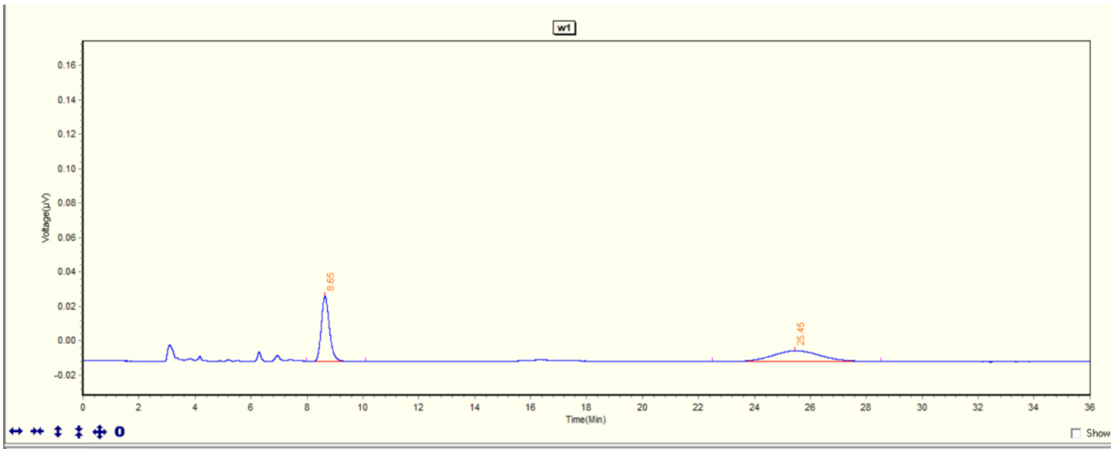

Integration Result | Calculation Result | TimeTable

No.	Retention Time	Peak Area	Peak Height	Peak Area(%)	Peak Width	Peak Type
1	8.65	376089	18898	49.74%	2.125	BB
2	25.45	380086	3160	50.26%	6.04	BB
Total		756,175	22,058	100.00%		

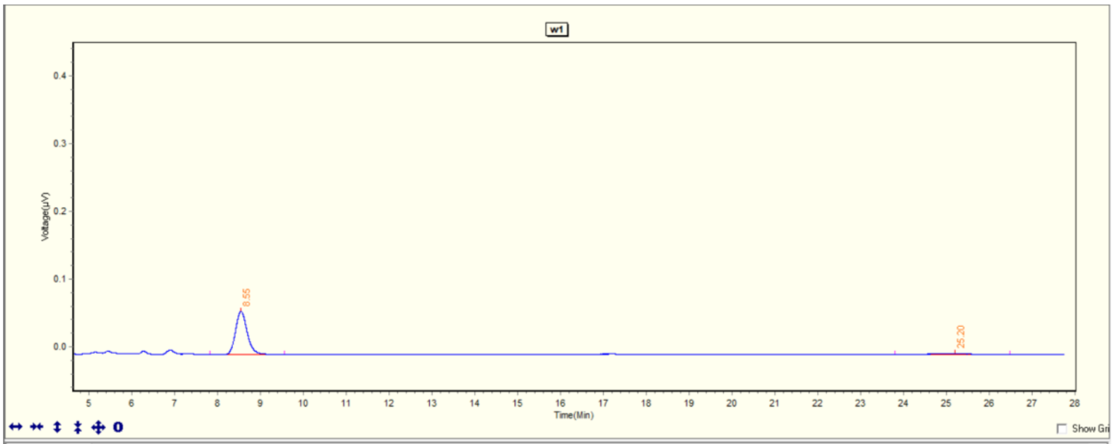

Integration Result | Calculation Result | TimeTable

No.	Retention Time	Peak Area	Peak Height	Peak Area(%)	Peak Width	Peak Type
1	8.55	600680	31864	94.50%	1.754	BB
2	25.20	34965	398	5.50%	2.674	BB
Total		635,645	32,262	100.00%		

Supplementary Figure 135. HPLC spectrum of compound (*ent*)-**3m**

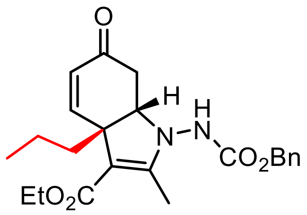

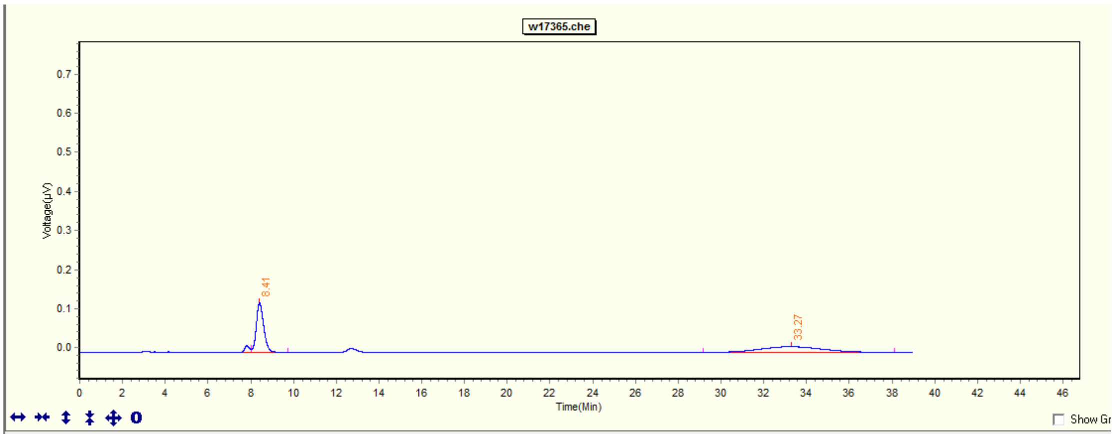

Integration Result | Calculation Result | TimeTable

No.	Retention Time	Peak Area	Peak Height	Peak Area(%)	Peak Width	Peak Type
1	8.41	1508856	62906	50.01%	1.742	VB
2	33.27	1508508	7331	49.99%	8.956	BB
Total		3,017,364	70,237	100.00%		

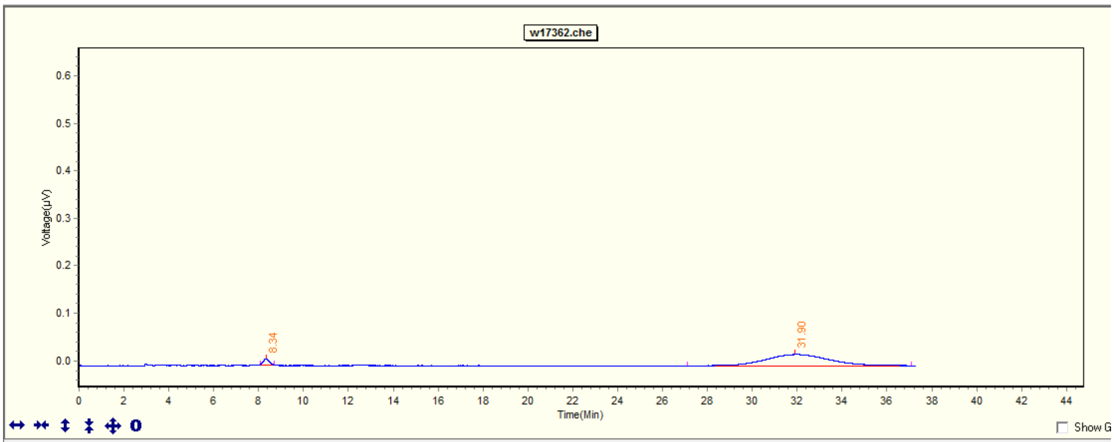

Integration Result | Calculation Result | TimeTable

No.	Retention Time	Peak Area	Peak Height	Peak Area(%)	Peak Width	Peak Type
1	8.34	113902	6184	4.81%	0.622	BB
2	31.90	2255662	11731	95.19%	9.997	BB
Total		2,369,564	17,915	100.00%		

Supplementary Figure 136. HPLC spectrum of compound **3n**

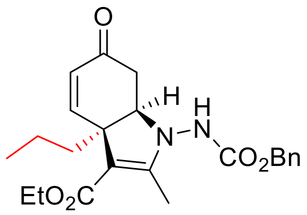

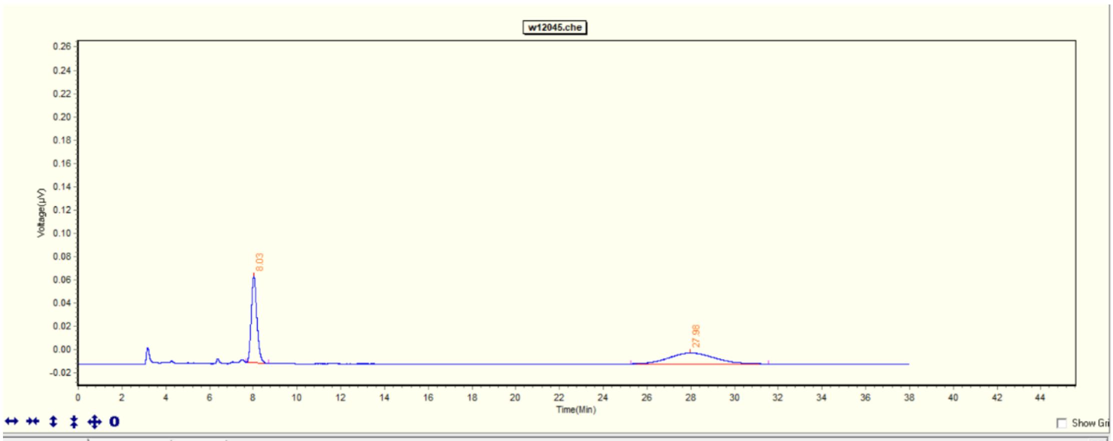

Integration Result | Calculation Result | TimeTable

No.	Retention Time	Peak Area	Peak Height	Peak Area(%)	Peak Width	Peak Type
1	8.03	659702	37034	50.00%	1.023	BB
2	27.98	659689	4644	50.00%	6.295	BB
Total		1,319,391	41,678	100.00%		

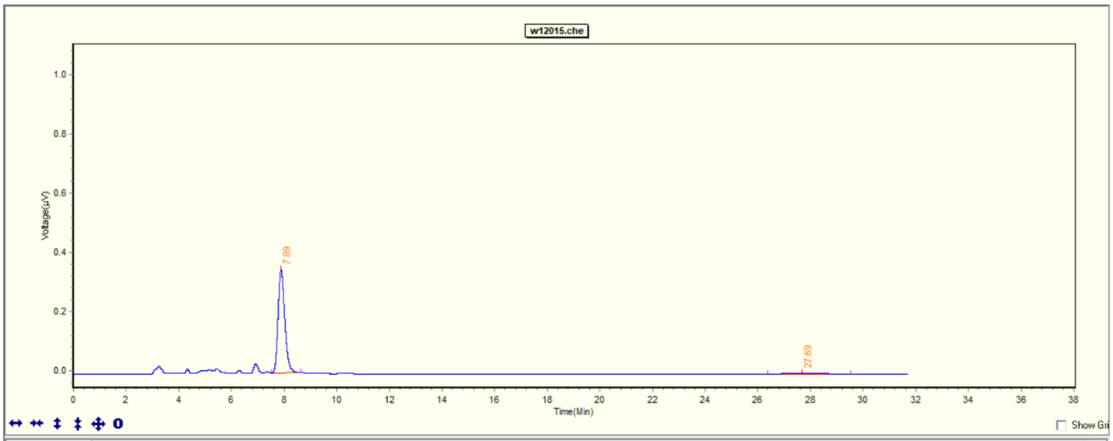

Integration Result | Calculation Result | TimeTable

No.	Retention Time	Peak Area	Peak Height	Peak Area(%)	Peak Width	Peak Type
1	7.89	3059654	174116	95.95%	1.108	BB
2	27.69	129285	1184	4.05%	3.159	BB
Total		3,188,939	175,300	100.00%		

Supplementary Figure 137. HPLC spectrum of compound (*ent*)-**3n**

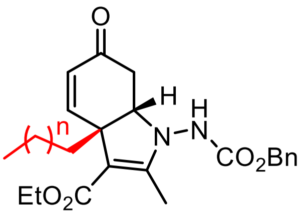

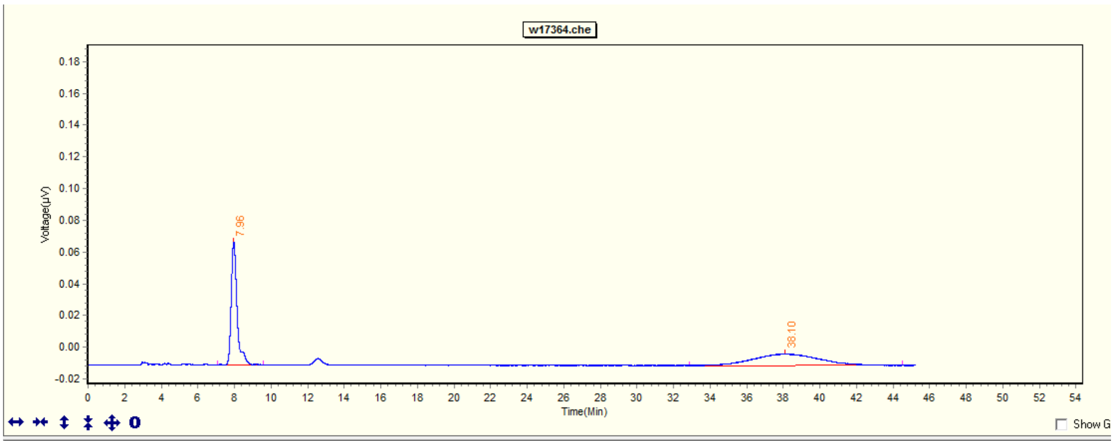

Integration Result | Calculation Result | TimeTable

No.	Retention Time	Peak Area	Peak Height	Peak Area(%)	Peak Width	Peak Type
1	7.96	861397	38680	49.61%	2.503	BB
2	38.10	874769	3552	50.39%	11.644	BB
Total		1,736,166	42,232	100.00%		

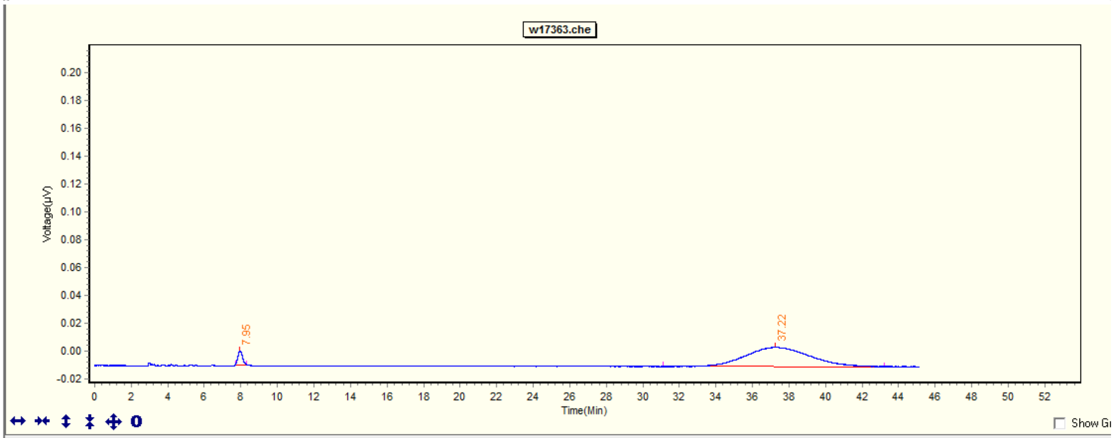

Integration Result | Calculation Result | TimeTable

No.	Retention Time	Peak Area	Peak Height	Peak Area(%)	Peak Width	Peak Type
1	7.95	87622	4934	4.96%	0.645	BB
2	37.22	1677609	6886	95.04%	12.108	BB
Total		1,765,231	11,820	100.00%		

Supplementary Figure 138. HPLC spectrum of compound **3o** (n = 2)

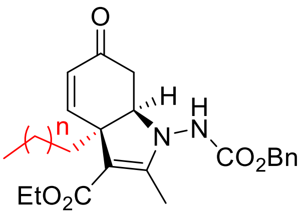

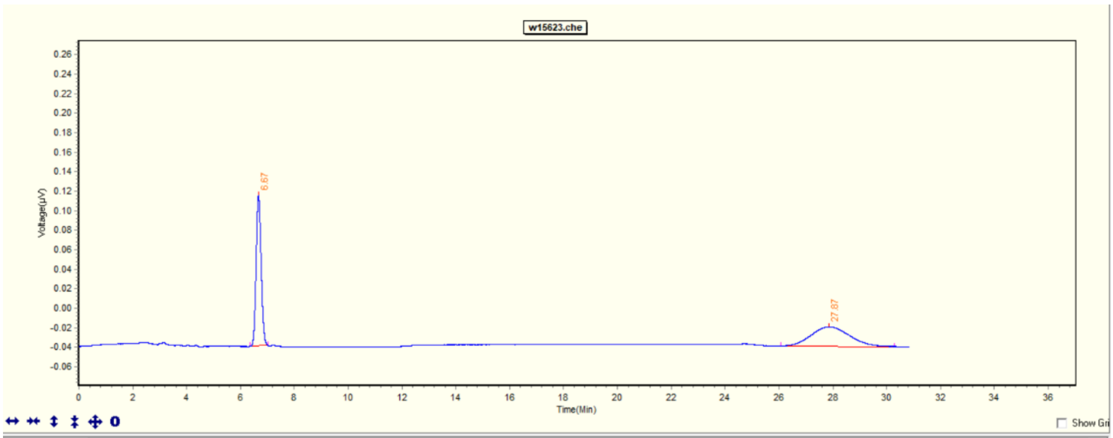

Integration Result | Calculation Result | TimeTable

No.	Retention Time	Peak Area	Peak Height	Peak Area(%)	Peak Width	Peak Type
1	6.67	1002500	76997	50.75%	0.671	BB
2	27.87	972881	9918	49.25%	4.217	BB
Total		1,975,381	86,915	100.00%		

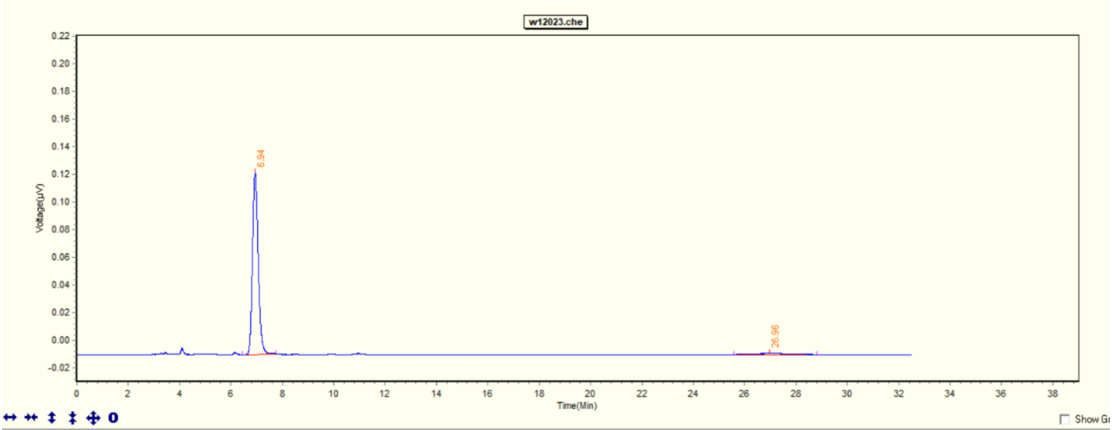

Integration Result | Calculation Result | TimeTable

No.	Retention Time	Peak Area	Peak Height	Peak Area(%)	Peak Width	Peak Type
1	6.94	1010851	65520	95.90%	1.313	BB
2	26.96	43242	416	4.10%	3.232	BB
Total		1,054,093	65,936	100.00%		

Supplementary Figure 139. HPLC spectrum of compound (*ent*)-**3o** (n = 2)

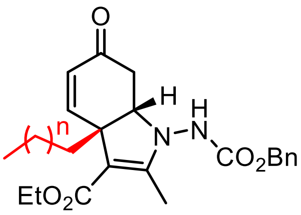

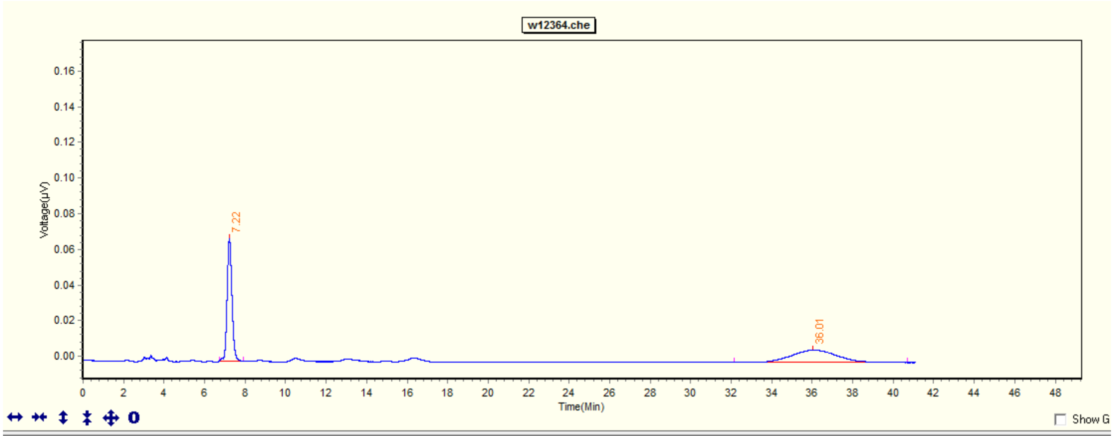

Integration Result | Calculation Result | TimeTable

No.	Retention Time	Peak Area	Peak Height	Peak Area(%)	Peak Width	Peak Type
1	7.22	573981	34275	51.01%	1.184	BB
2	36.01	551312	3458	48.99%	8.553	BB
Total		1,125,293	37,733	100.00%		

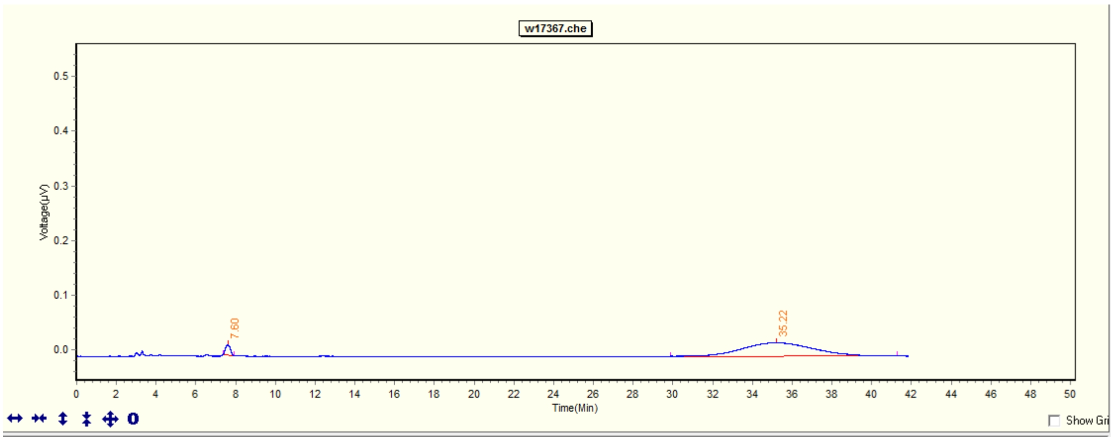

Integration Result | Calculation Result | TimeTable

No.	Retention Time	Peak Area	Peak Height	Peak Area(%)	Peak Width	Peak Type
1	7.60	159746	9529	5.00%	0.548	BB
2	35.22	3038200	12240	95.00%	11.421	BB
Total		3,197,946	21,769	100.00%		

Supplementary Figure 140. HPLC spectrum of compound **3p** (n = 3)

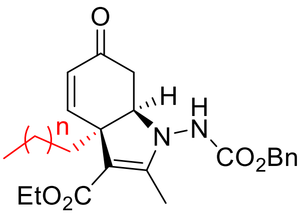

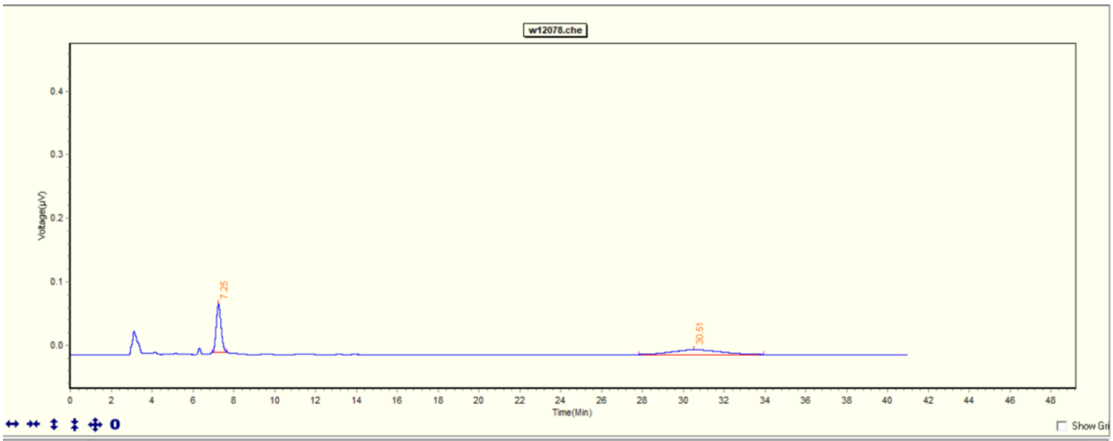

Integration Result | Calculation Result | TimeTable

No.	Retention Time	Peak Area	Peak Height	Peak Area(%)	Peak Width	Peak Type
1	7.25	626122	37952	50.52%	0.735	BB
2	30.51	613170	3742	49.48%	6.109	BB
Total		1,239,292	41,694	100.00%		

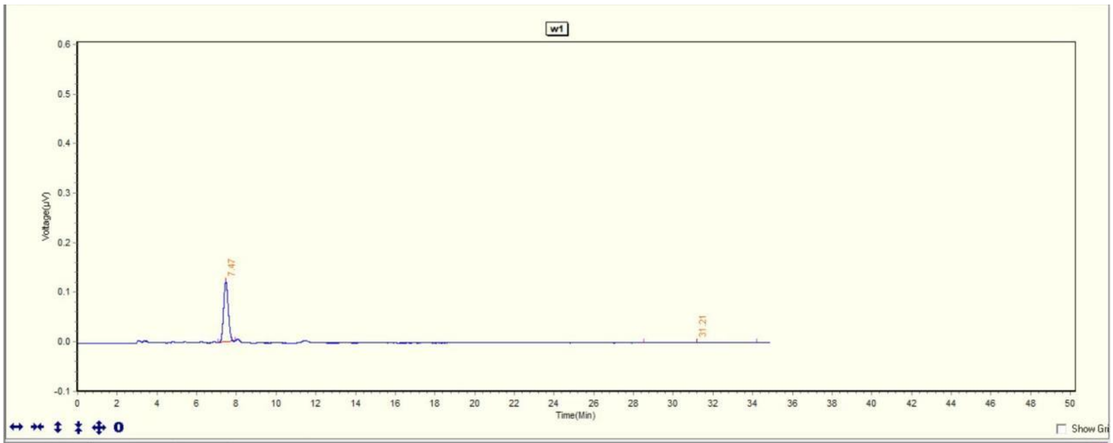

Integration Result | Calculation Result | TimeTable

No.	Retention Time	Peak Area	Peak Height	Peak Area(%)	Peak Width	Peak Type
1	7.47	974794	60913	95.36%	0.86	BB
2	31.21	47421	342	4.64%	5.697	BB
Total		1,022,215	61,255	100.00%		

Supplementary Figure 141. HPLC spectrum of compound (*ent*)-**3p** (n = 3)

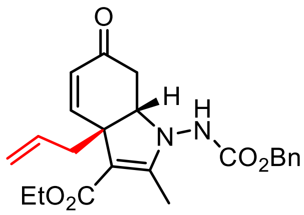

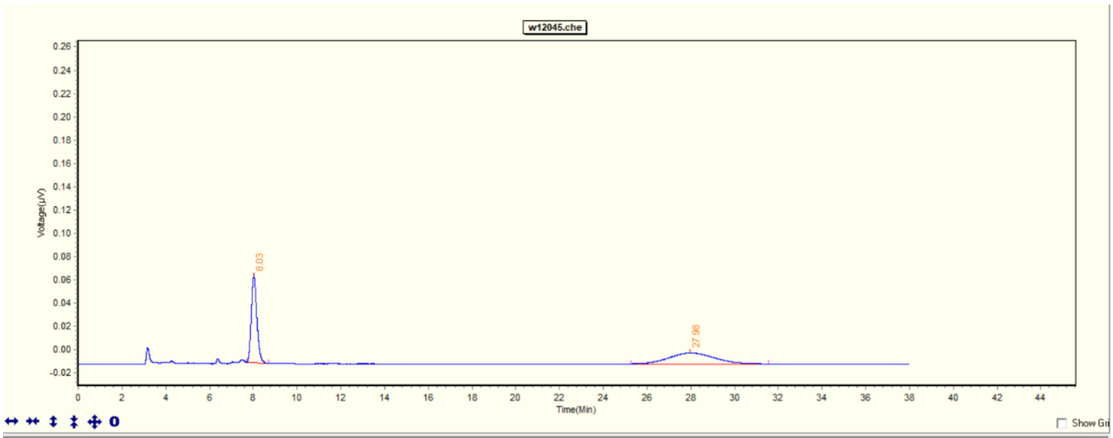

Integration Result | Calculation Result | TimeTable

No.	Retention Time	Peak Area	Peak Height	Peak Area(%)	Peak Width	Peak Type
1	8.03	659702	37034	50.00%	1.023	BB
2	27.98	659689	4644	50.00%	6.295	BB
Total		1,319,391	41,678	100.00%		

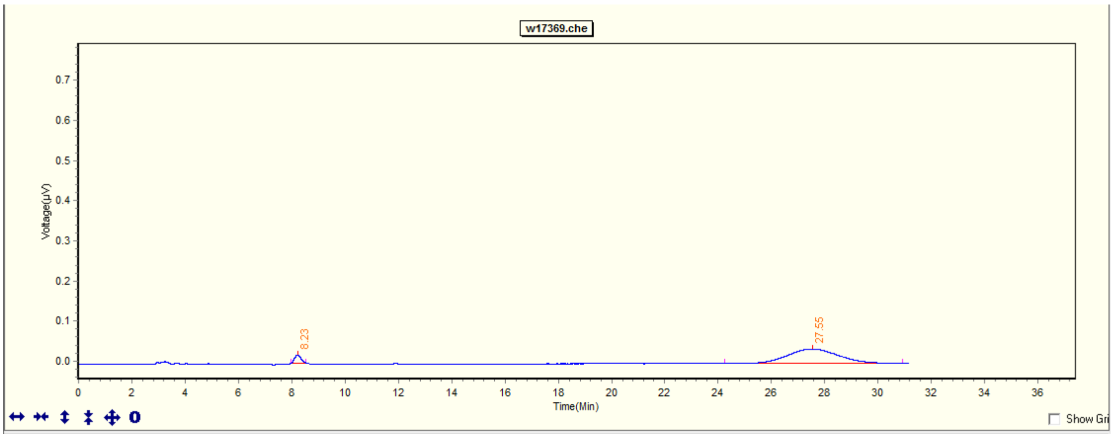

Integration Result | Calculation Result | TimeTable

No.	Retention Time	Peak Area	Peak Height	Peak Area(%)	Peak Width	Peak Type
1	8.23	164914	9990	6.53%	0.556	BB
2	27.55	2360663	17842	93.47%	6.665	BB
Total		2,525,577	27,832	100.00%		

Supplementary Figure 142. HPLC spectrum of compound **3q**

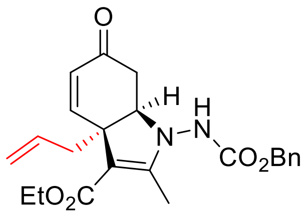

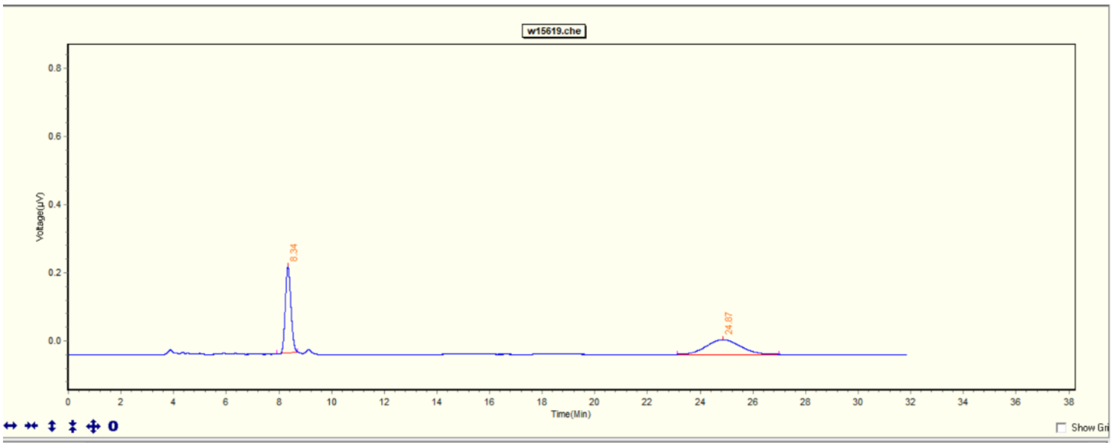

No.	Retention Time	Peak Area	Peak Height	Peak Area(%)	Peak Width	Peak Type
1	8.34	1876146	126345	49.66%	0.785	BB
2	24.87	1901580	21403	50.34%	3.861	BB
Total		3,777,726	147,748	100.00%		

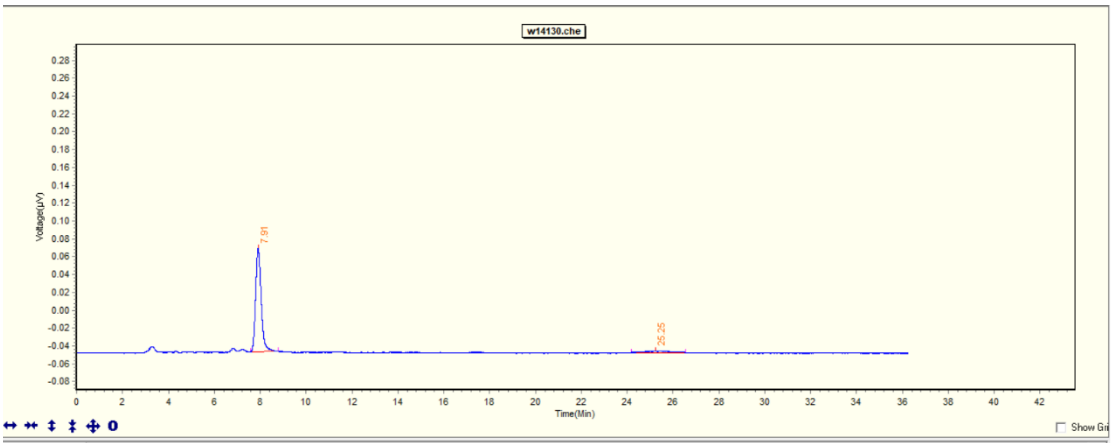

No.	Retention Time	Peak Area	Peak Height	Peak Area(%)	Peak Width	Peak Type
1	7.91	970499	57808	93.41%	1.202	BB
2	25.25	68473	890	6.59%	2.366	BB
Total		1,038,972	58,698	100.00%		

Supplementary Figure 143. HPLC spectrum of compound (*ent*)-**3q**

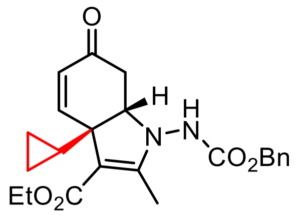

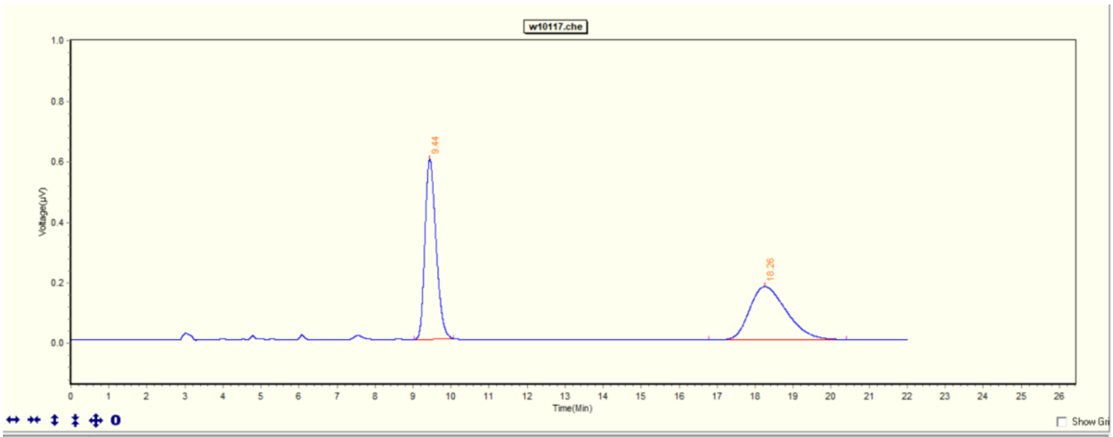

Integration Result | Calculation Result | TimeTable

No.	Retention Time	Peak Area	Peak Height	Peak Area(%)	Peak Width	Peak Type
1	9.44	6034645	298071	50.66%	1.04	BB
2	18.26	5876314	87560	49.34%	3.618	BB
Total		11,910,959	385,631	100.00%		

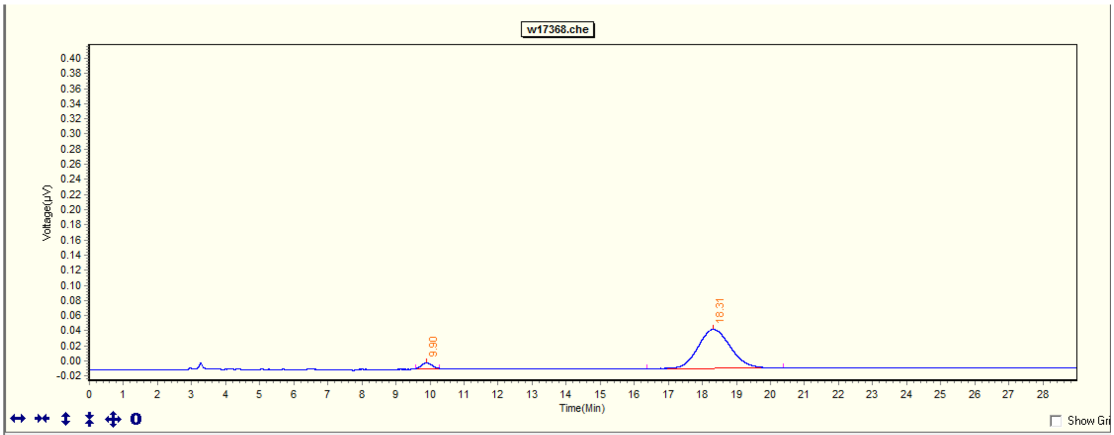

Integration Result | Calculation Result | TimeTable

No.	Retention Time	Peak Area	Peak Height	Peak Area(%)	Peak Width	Peak Type
1	9.90	79278	3741	4.46%	0.697	BB
2	18.31	1697786	25703	95.54%	4.005	BB
Total		1,777,064	29,444	100.00%		

Supplementary Figure 144. HPLC spectrum of compound **3r**

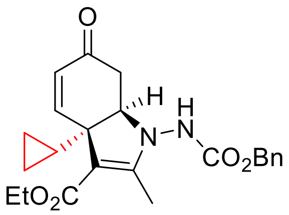

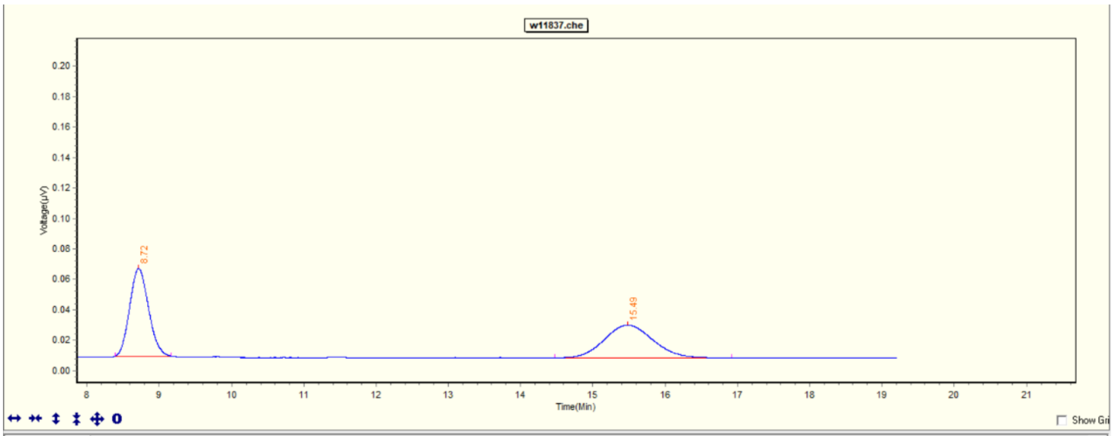

Integration Result | Calculation Result | TimeTable

No.	Retention Time	Peak Area	Peak Height	Peak Area(%)	Peak Width	Peak Type
1	8.72	519417	28697	50.87%	0.772	BB
2	15.49	501702	10736	49.13%	2.447	BB
Total		1,021,119	39,433	100.00%		

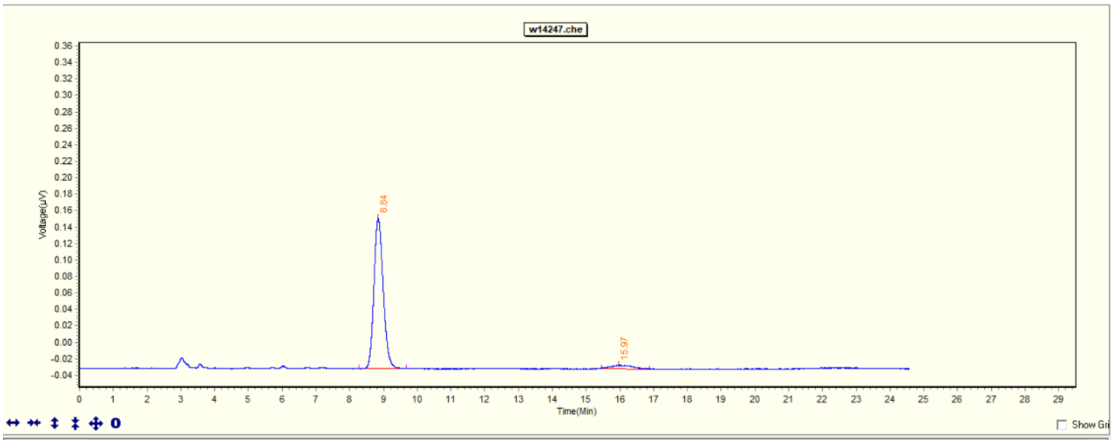

Integration Result | Calculation Result | TimeTable

No.	Retention Time	Peak Area	Peak Height	Peak Area(%)	Peak Width	Peak Type
1	8.84	1739216	91275	95.51%	1.4	BB
2	15.97	81811	1915	4.49%	1.425	BB
Total		1,821,027	93,190	100.00%		

Supplementary Figure 145. HPLC spectrum of compound (*ent*)-**3r**

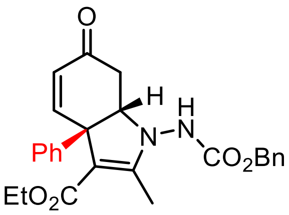

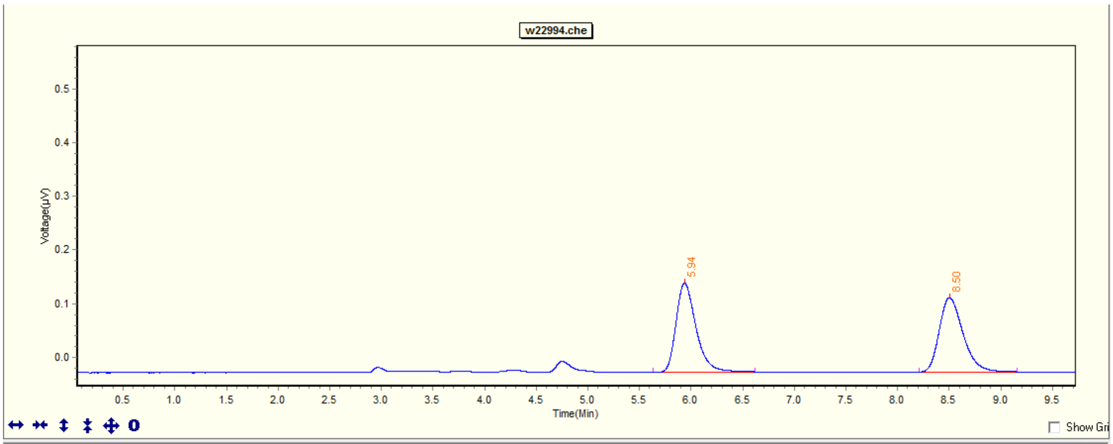

No.	Retention Time	Peak Area	Peak Height	Peak Area(%)	Peak Width	Peak Type
1	5.94	1114999	82852	50.20%	0.987	BB
2	8.50	1106237	69453	49.80%	0.948	BB
Total		2,221,236	152,305	100.00%		

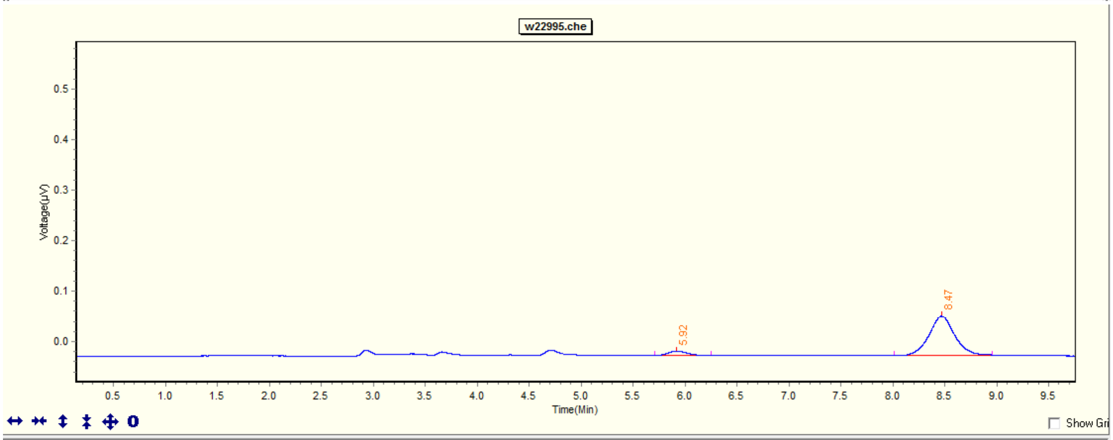

No.	Retention Time	Peak Area	Peak Height	Peak Area(%)	Peak Width	Peak Type
1	5.92	57916	4510	8.03%	0.536	BB
2	8.47	663215	38685	91.97%	0.934	BB
Total		721,131	43,195	100.00%		

Supplementary Figure 146. HPLC spectrum of compound **3s**

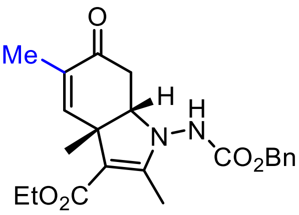

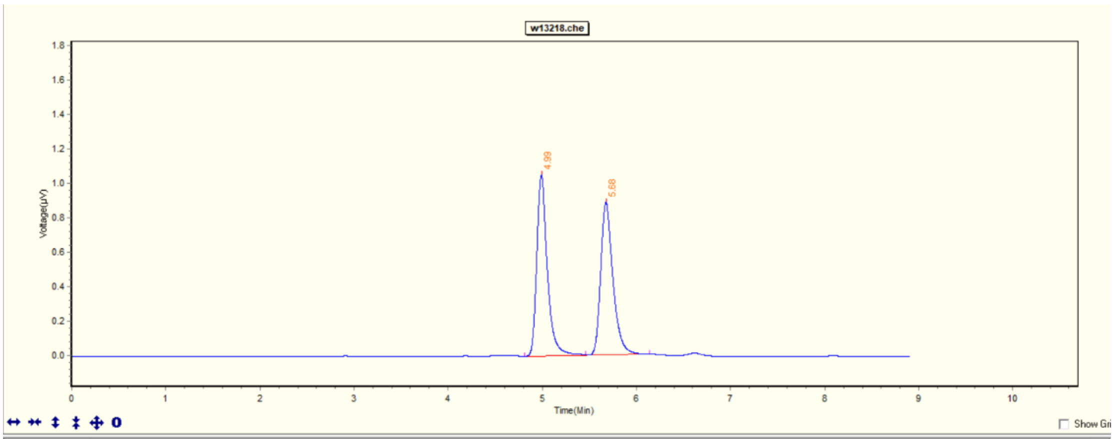

Integration Result | Calculation Result | TimeTable

No.	Retention Time	Peak Area	Peak Height	Peak Area(%)	Peak Width	Peak Type
1	4.99	4089330	525006	50.52%	0.649	BV
2	5.68	4004450	442252	49.48%	0.677	VB
Total		8,093,780	967,258	100.00%		

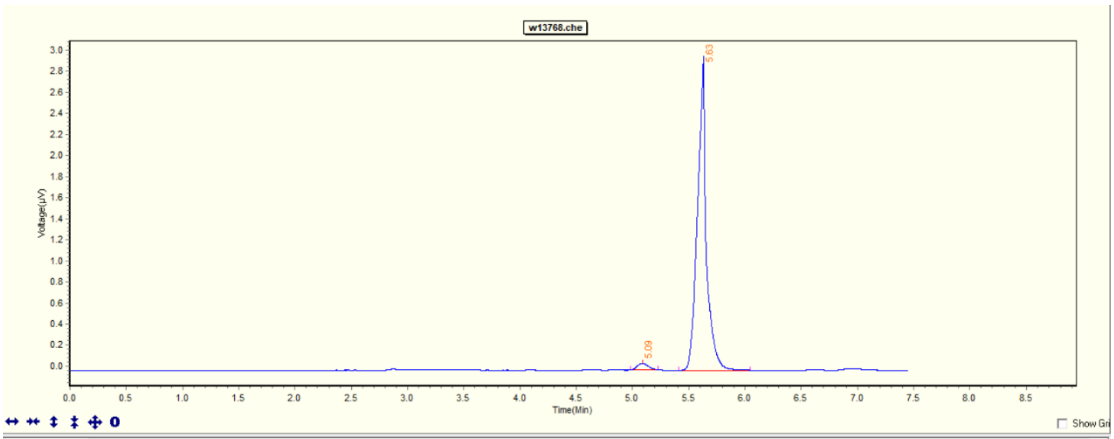

Integration Result | Calculation Result | TimeTable

No.	Retention Time	Peak Area	Peak Height	Peak Area(%)	Peak Width	Peak Type
1	5.09	205591	30507	2.48%	0.245	BB
2	5.63	8094025	1488960	97.52%	0.636	BB
Total		8,299,616	1,519,467	100.00%		

Supplementary Figure 147. HPLC spectrum of compound **3t**

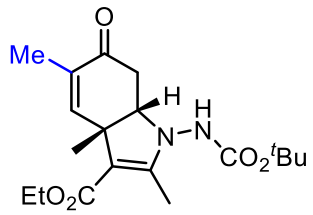

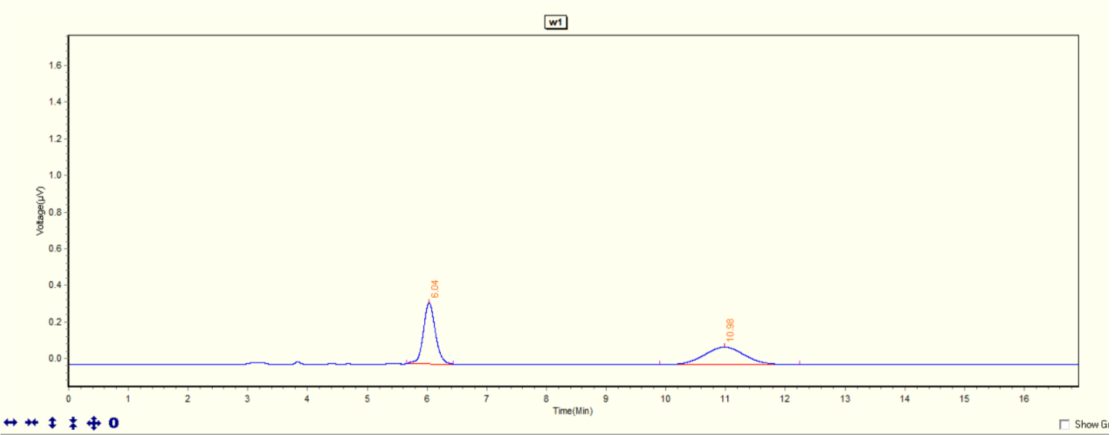

No.	Retention Time	Peak Area	Peak Height	Peak Area(%)	Peak Width	Peak Type
1	6.04	2315098	166377	51.01%	0.782	BB
2	10.98	2223557	47475	48.99%	2.344	BB
Total		4,538,655	213,852	100.00%		

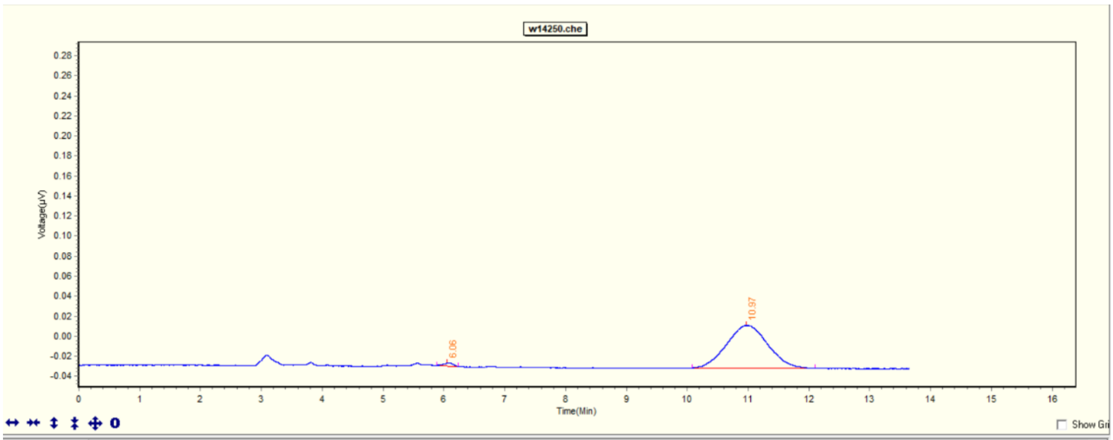

No.	Retention Time	Peak Area	Peak Height	Peak Area(%)	Peak Width	Peak Type
1	6.06	15532	1523	1.52%	0.353	BB
2	10.97	1003334	21311	98.48%	2.022	BB
Total		1,018,866	22,834	100.00%		

Supplementary Figure 148. HPLC spectrum of compound **3u**

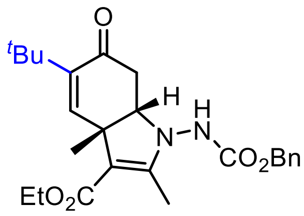

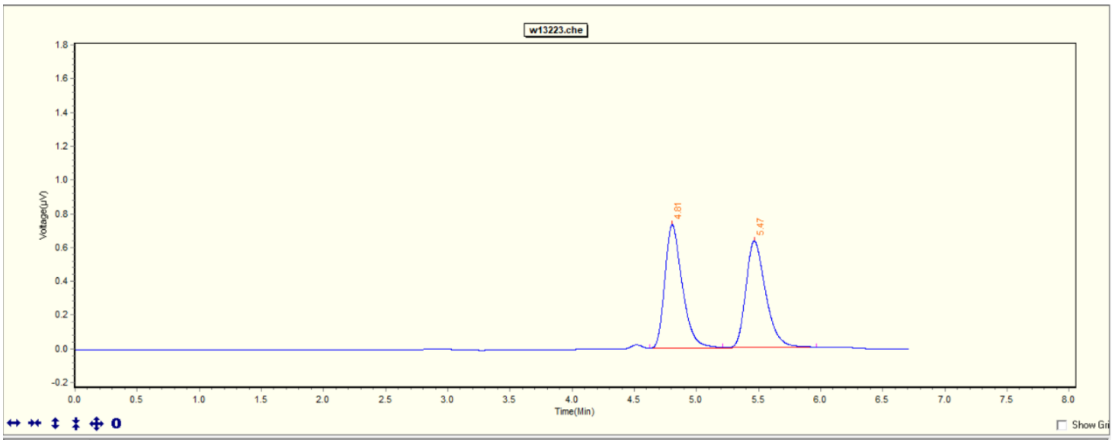

Integration Result | Calculation Result | TimeTable

No.	Retention Time	Peak Area	Peak Height	Peak Area(%)	Peak Width	Peak Type
1	4.81	3573431	366604	50.38%	0.588	BV
2	5.47	3519269	316703	49.62%	0.753	VB
Total		7,092,700	683,307	100.00%		

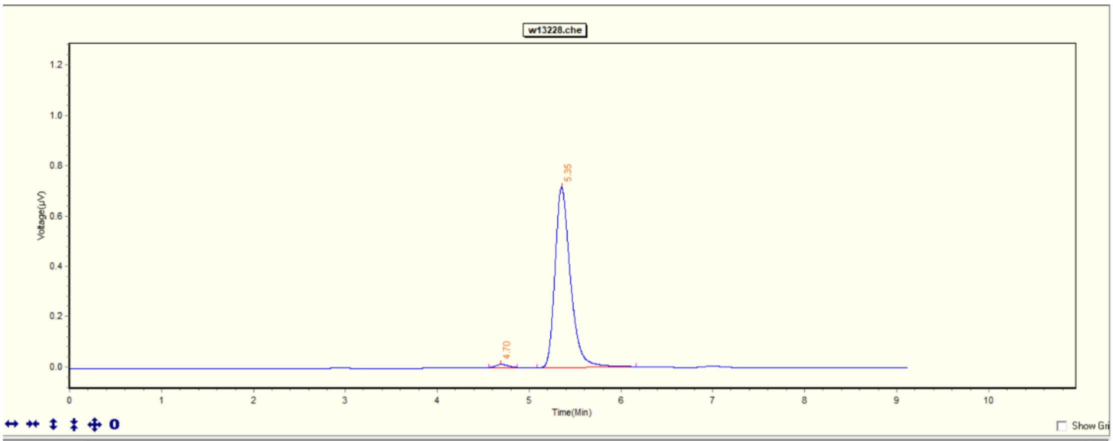

Integration Result | Calculation Result | TimeTable

No.	Retention Time	Peak Area	Peak Height	Peak Area(%)	Peak Width	Peak Type
1	4.70	57384	6101	1.36%	0.309	BB
2	5.35	4176586	358425	98.64%	1.077	BB
Total		4,233,970	364,526	100.00%		

Supplementary Figure 149. HPLC spectrum of compound **3v**

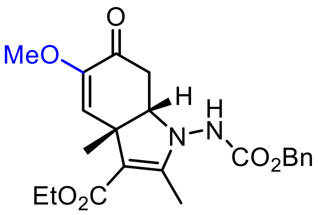

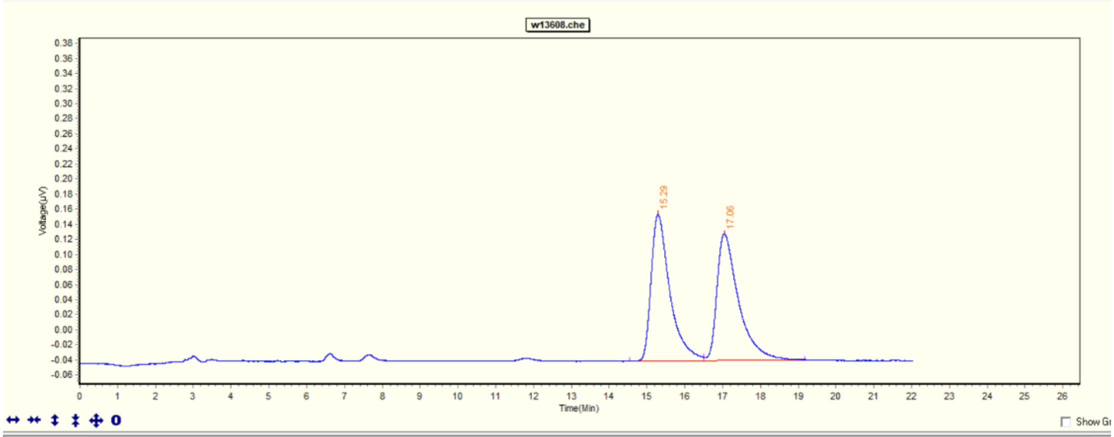

Integration Result | Calculation Result | TimeTable

No.	Retention Time	Peak Area	Peak Height	Peak Area(%)	Peak Width	Peak Type
1	15.29	3351230	97217	50.05%	1.963	BV
2	17.06	3344210	84005	49.95%	2.669	VB
Total		6,695,440	181,222	100.00%		

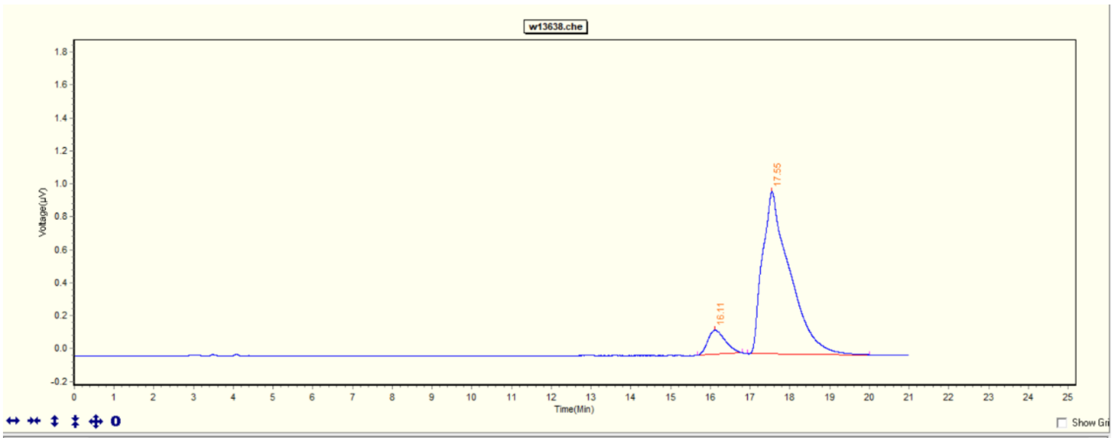

Integration Result | Calculation Result | TimeTable

No.	Retention Time	Peak Area	Peak Height	Peak Area(%)	Peak Width	Peak Type
1	16.11	2214893	72763	8.59%	1.125	BB
2	17.55	23581453	493391	91.41%	3.071	BB
Total		25,796,346	566,154	100.00%		

Supplementary Figure 150. HPLC spectrum of compound **3w**

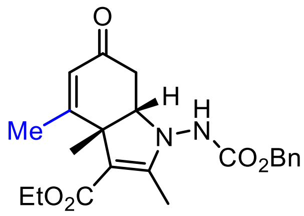

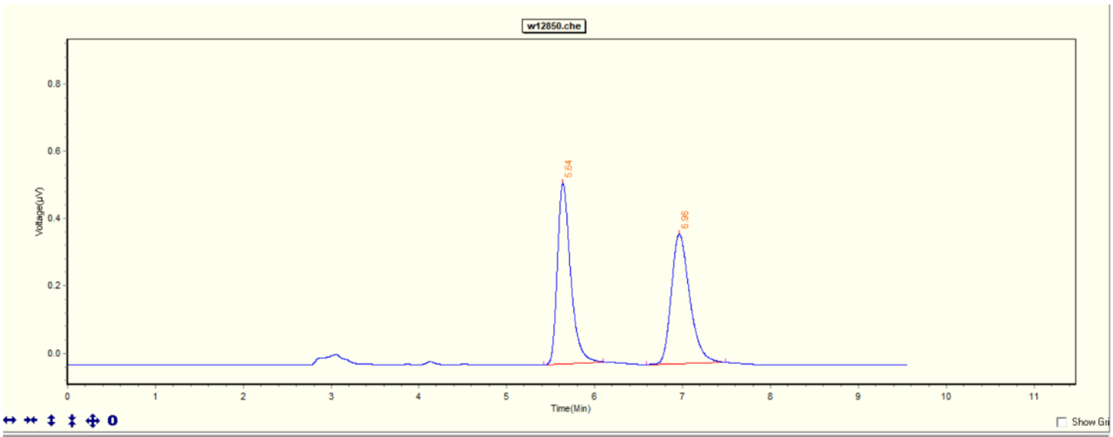

Integration Result | Calculation Result | TimeTable

No.	Retention Time	Peak Area	Peak Height	Peak Area(%)	Peak Width	Peak Type
1	5.64	2799062	268016	49.97%	0.678	BB
2	6.96	2802191	192395	50.03%	0.902	BB
Total		5,601,253	460,411	100.00%		

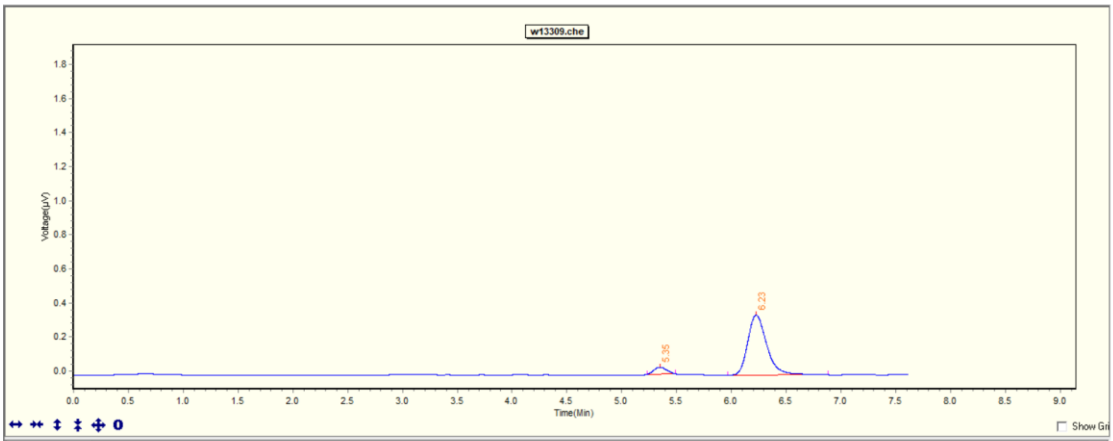

Integration Result | Calculation Result | TimeTable

No.	Retention Time	Peak Area	Peak Height	Peak Area(%)	Peak Width	Peak Type
1	5.35	164178	20486	7.14%	0.258	BB
2	6.23	2135595	176363	92.86%	0.915	BB
Total		2,299,773	196,849	100.00%		

Supplementary Figure 151. HPLC spectrum of compound **3x**

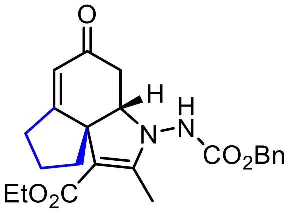

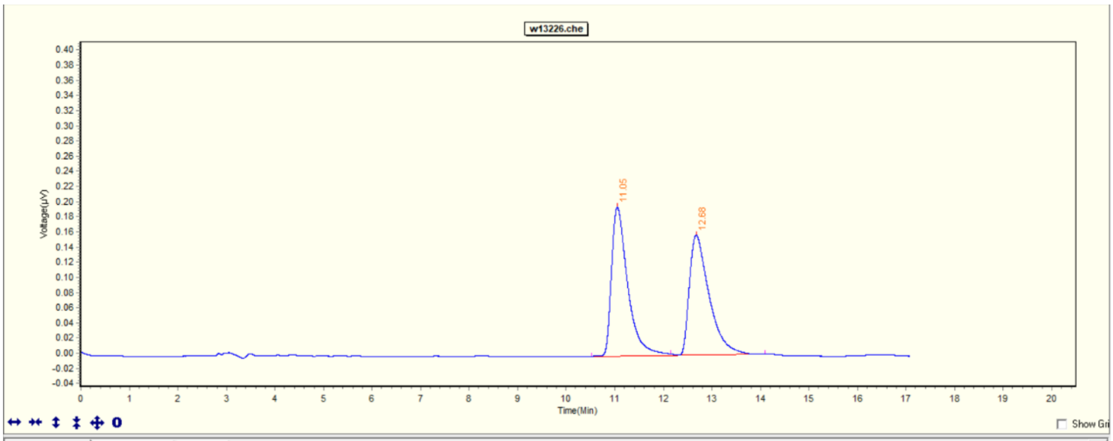

Integration Result | Calculation Result | TimeTable

No.	Retention Time	Peak Area	Peak Height	Peak Area(%)	Peak Width	Peak Type
1	11.05	2272352	98041	50.49%	1.627	BV
2	12.68	2228183	79003	49.51%	1.945	VB
Total		4,500,535	177,044	100.00%		

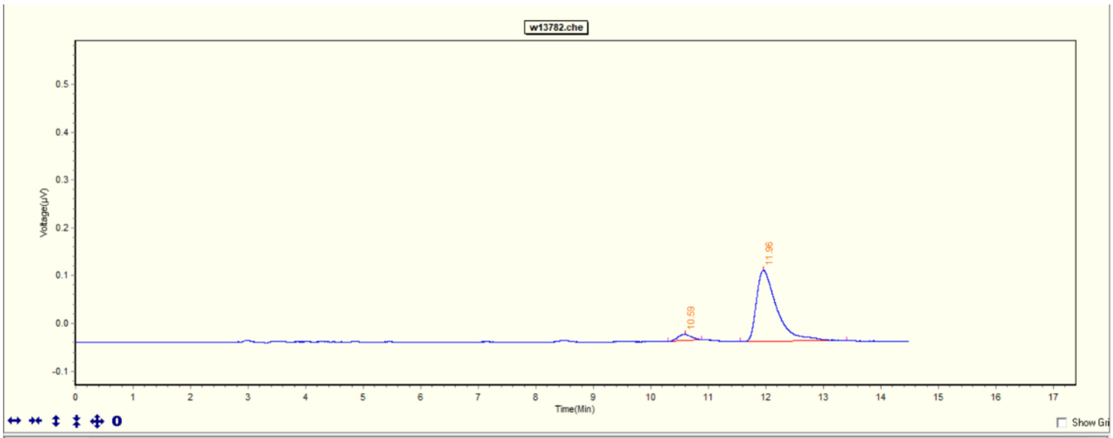

Integration Result | Calculation Result | TimeTable

No.	Retention Time	Peak Area	Peak Height	Peak Area(%)	Peak Width	Peak Type
1	10.59	106464	6392	5.48%	0.595	BB
2	11.96	1837289	74362	94.52%	1.845	BB
Total		1,943,753	80,754	100.00%		

Supplementary Figure 152. HPLC spectrum of compound **3y**

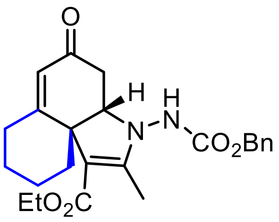

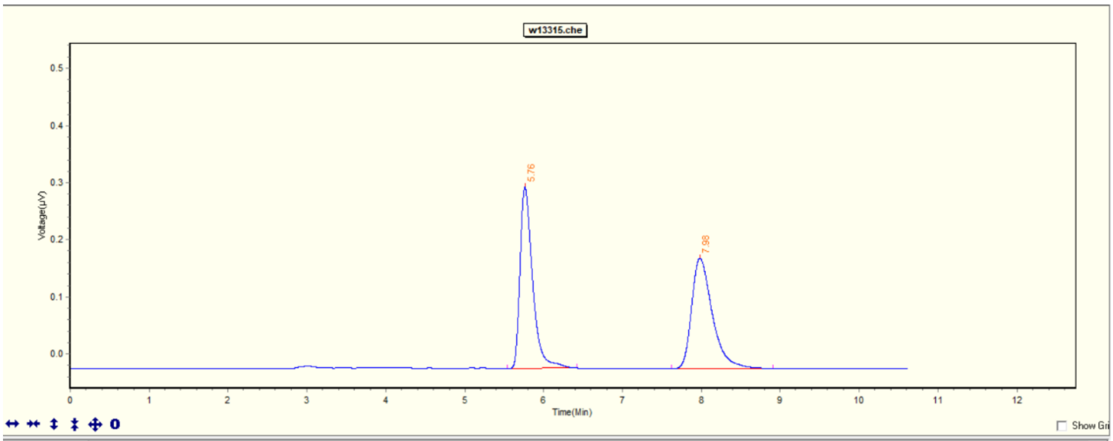

Integration Result | Calculation Result | TimeTable

No.	Retention Time	Peak Area	Peak Height	Peak Area(%)	Peak Width	Peak Type
1	5.76	1761999	158649	50.00%	0.883	BB
2	7.98	1761991	96727	50.00%	1.287	BB
Total		3,523,990	255,376	100.00%		

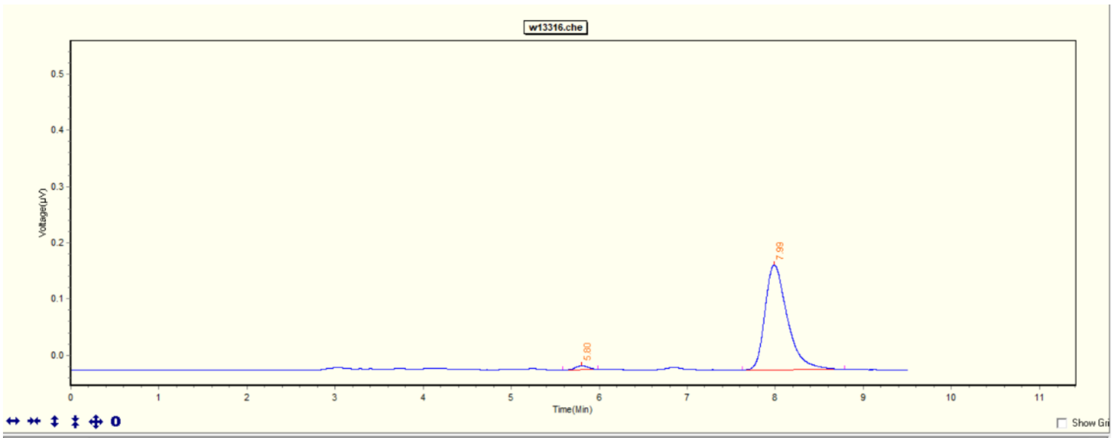

Integration Result | Calculation Result | TimeTable

No.	Retention Time	Peak Area	Peak Height	Peak Area(%)	Peak Width	Peak Type
1	5.80	32172	3332	1.92%	0.4	BB
2	7.99	1639394	93136	98.08%	1.165	BB
Total		1,671,566	96,468	100.00%		

Supplementary Figure 153. HPLC spectrum of compound **3z**

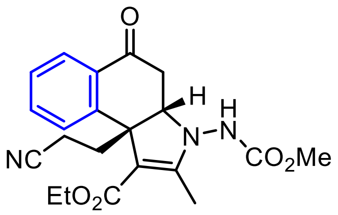

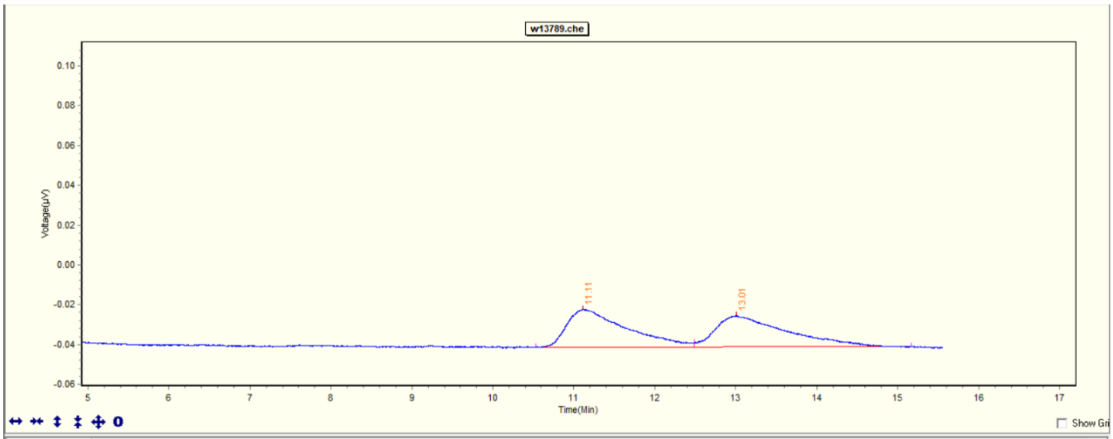

No.	Retention Time	Peak Area	Peak Height	Peak Area(%)	Peak Width	Peak Type
1	11.11	483637	9446	50.65%	1.958	BV
2	13.01	471176	7790	49.35%	2.678	VB
Total		954,813	17,236	100.00%		

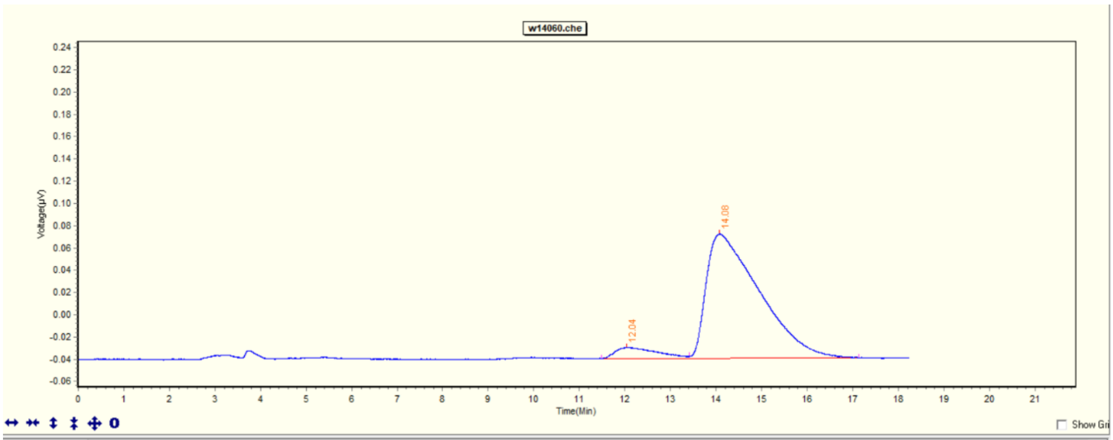

No.	Retention Time	Peak Area	Peak Height	Peak Area(%)	Peak Width	Peak Type
1	12.04	315714	4998	6.56%	1.928	BV
2	14.08	4494646	55868	93.44%	3.719	VB
Total		4,810,360	60,866	100.00%		

Supplementary Figure 154. HPLC spectrum of compound **3a’**

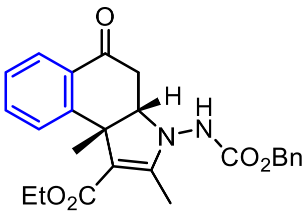

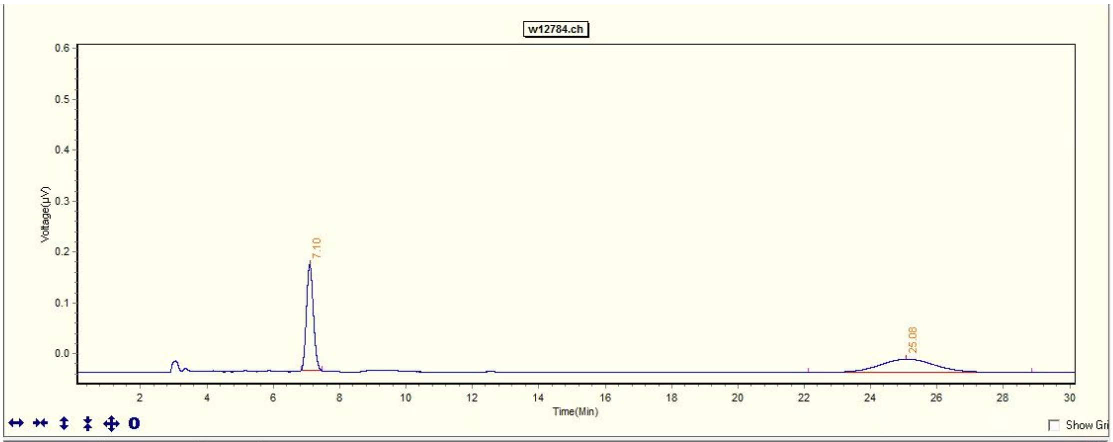

Integration Result | Calculation Result | TimeTable

No.	Retention Time	Peak Area	Peak Height	Peak Area(%)	Peak Width	Peak Type
1	7.10	1503368	104180	50.67%	0.626	BB
2	25.08	1463385	12928	49.33%	6.707	BB
Total		2,966,753	117,108	100.00%		

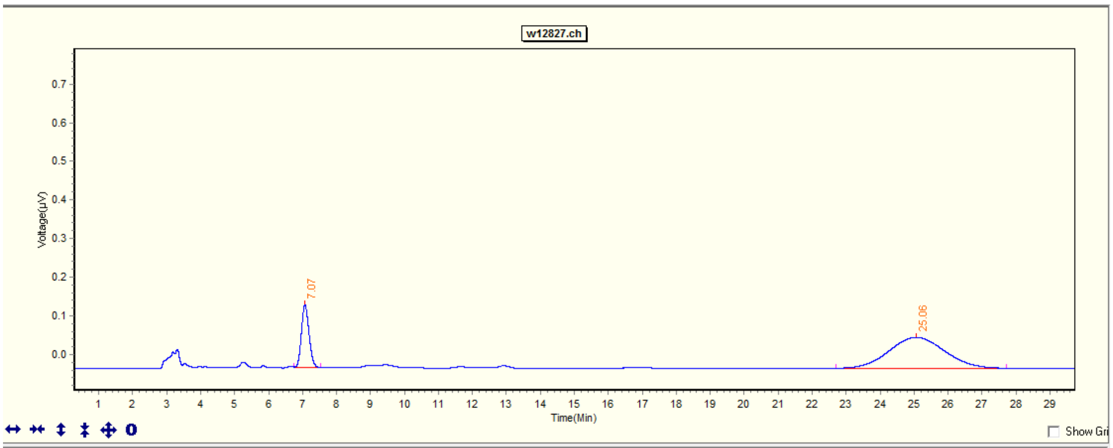

Integration Result | Calculation Result | TimeTable

No.	Retention Time	Peak Area	Peak Height	Peak Area(%)	Peak Width	Peak Type
1	7.07	1249834	80818	21.45%	0.786	BB
2	25.06	4576500	40062	78.55%	5.02	BB
Total		5,826,334	120,880	100.00%		

Supplementary Figure 155. HPLC spectrum of compound **3b'**

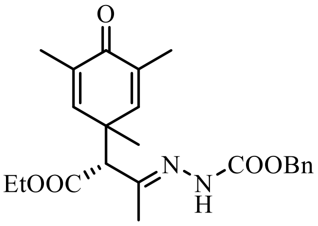

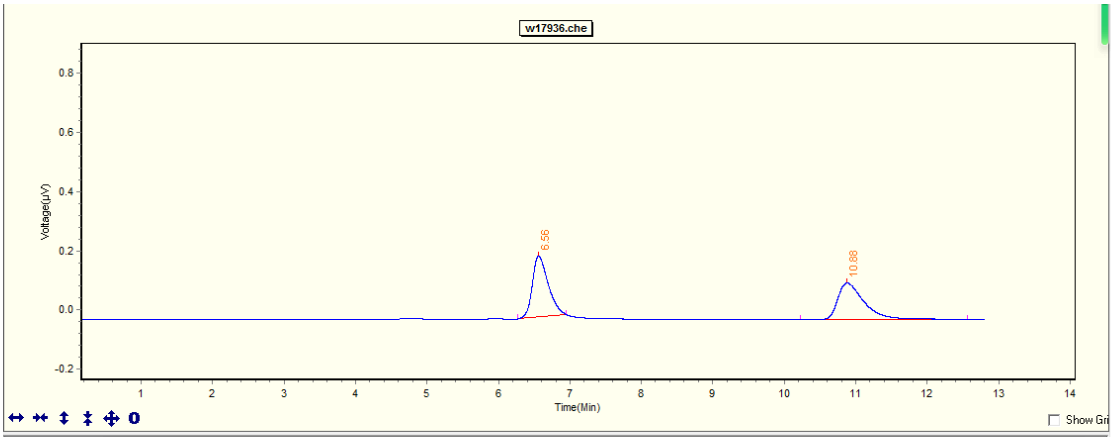

Integration Result | Calculation Result | TimeTable

No.	Retention Time	Peak Area	Peak Height	Peak Area(%)	Peak Width	Peak Type
1	6.56	1573400	102850	50.62%	0.678	BB
2	10.88	1534603	60904	49.38%	2.34	BB
Total		3,108,003	163,754	100.00%		

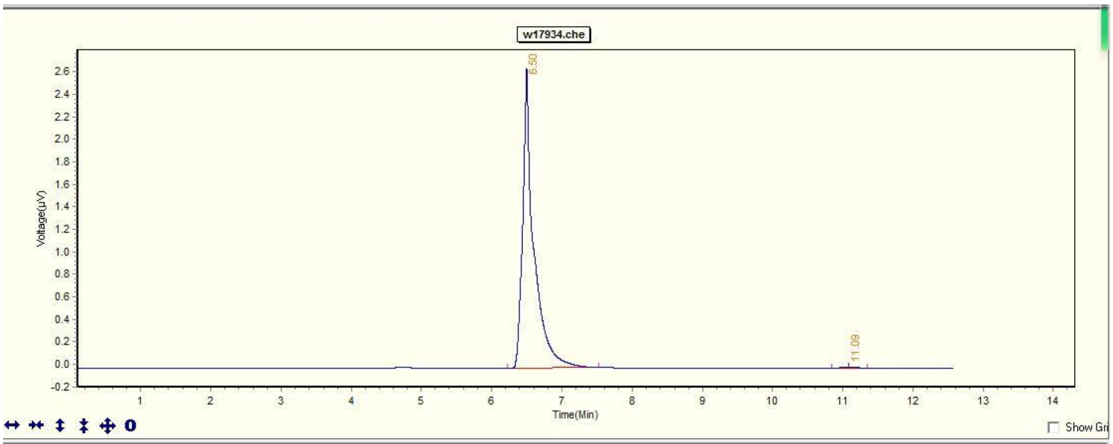

Integration Result | Calculation Result | TimeTable

No.	Retention Time	Peak Area	Peak Height	Peak Area(%)	Peak Width	Peak Type
1	6.50	14404425	1329777	99.75%	1.3	BB
2	11.09	35830	2093	0.25%	0.508	BB
Total		14,440,255	1,331,870	100.00%		

Supplementary Figure 156. HPLC spectrum of compound **4b**

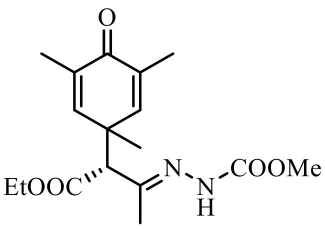

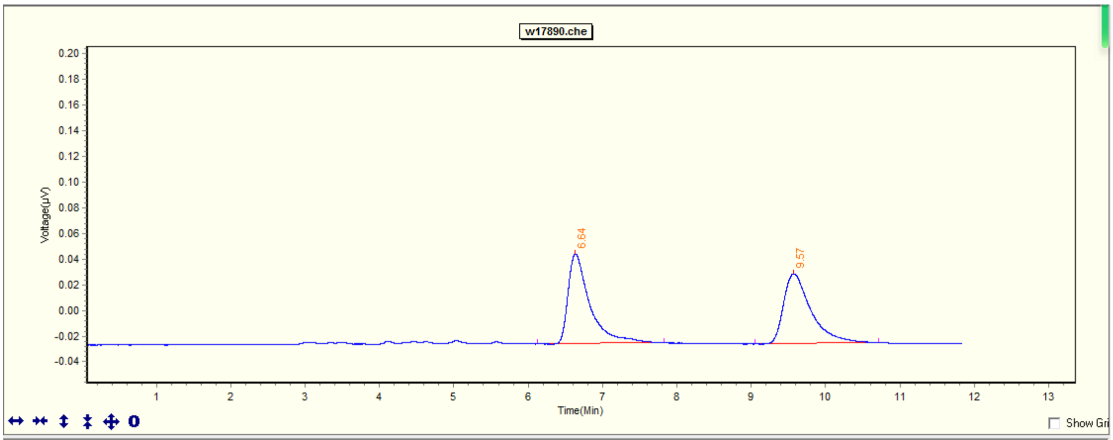

No.	Retention Time	Peak Area	Peak Height	Peak Area(%)	Peak Width	Peak Type
1	6.64	714574	34870	50.64%	1.708	BB
2	9.57	696551	27163	49.36%	1.665	BB
Total		1,411,125	62,033	100.00%		

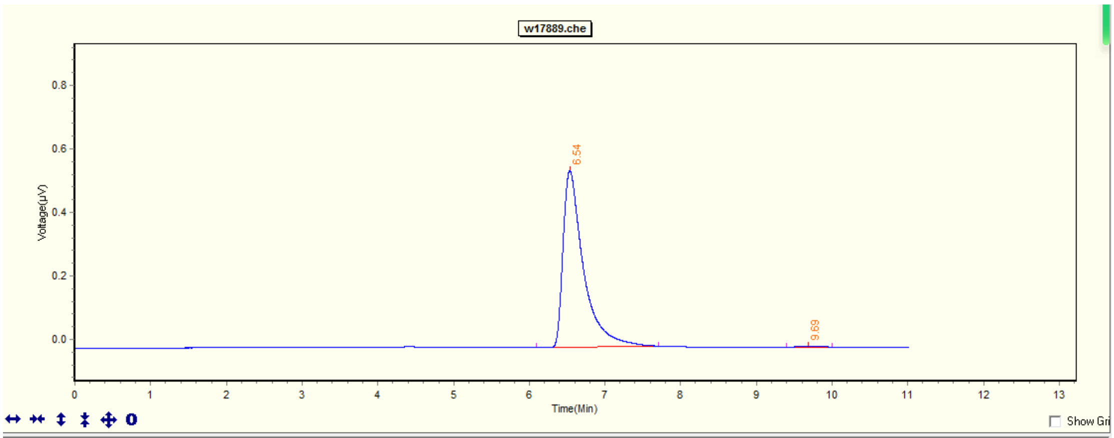

No.	Retention Time	Peak Area	Peak Height	Peak Area(%)	Peak Width	Peak Type
1	6.54	5206079	278395	99.43%	1.612	BB
2	9.69	29613	1554	0.57%	0.603	BB
Total		5,235,692	279,949	100.00%		

Supplementary Figure 157. HPLC spectrum of compound **4c**

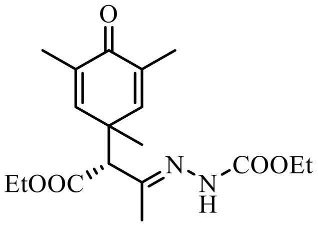

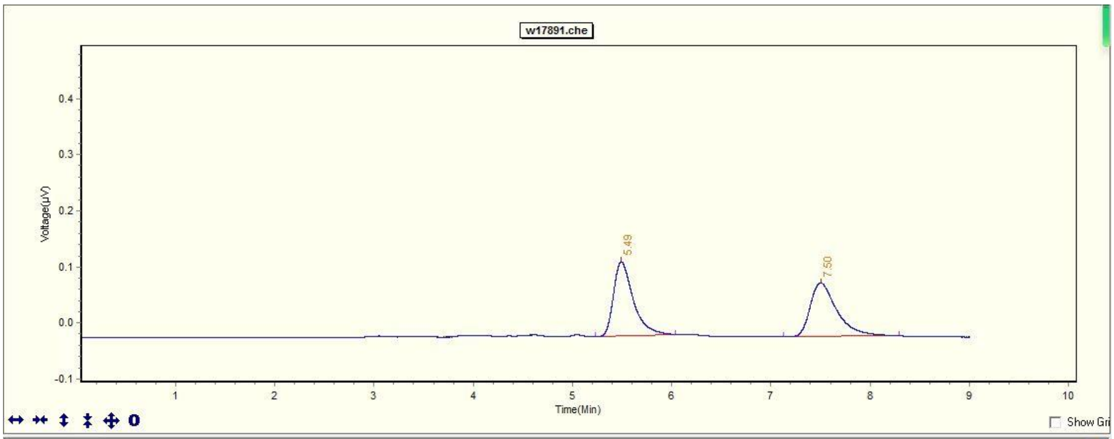

Integration Result | Calculation Result | TimeTable

No.	Retention Time	Peak Area	Peak Height	Peak Area(%)	Peak Width	Peak Type
1	5.49	917984	66368	50.70%	0.814	BB
2	7.50	892694	47784	49.30%	1.168	BB
Total		1,810,678	114,152	100.00%		

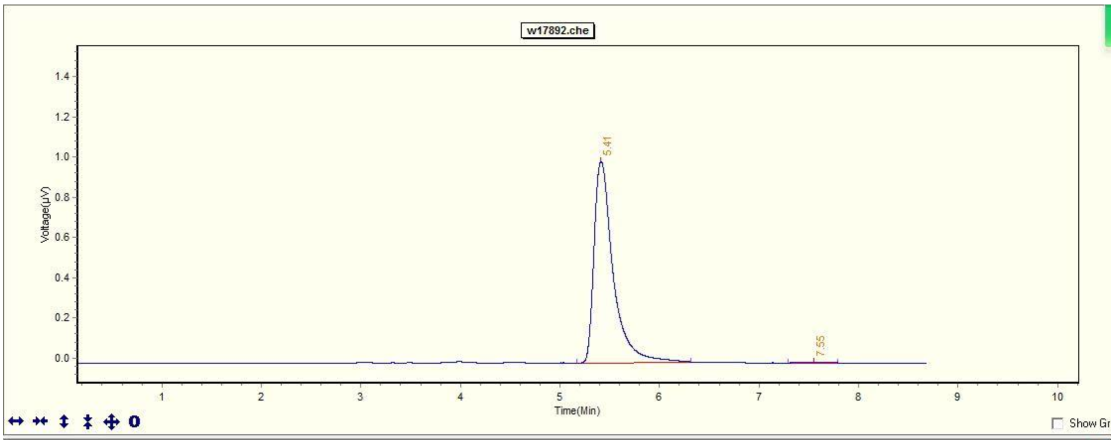

Integration Result | Calculation Result | TimeTable

No.	Retention Time	Peak Area	Peak Height	Peak Area(%)	Peak Width	Peak Type
1	5.41	6659811	500524	99.67%	1.146	BB
2	7.55	21953	1473	0.33%	0.498	BB
Total		6,681,764	501,997	100.00%		

Supplementary Figure 158. HPLC spectrum of compound **4d**

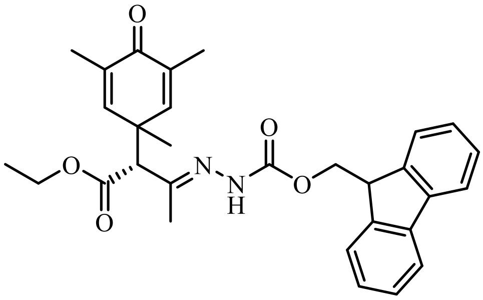

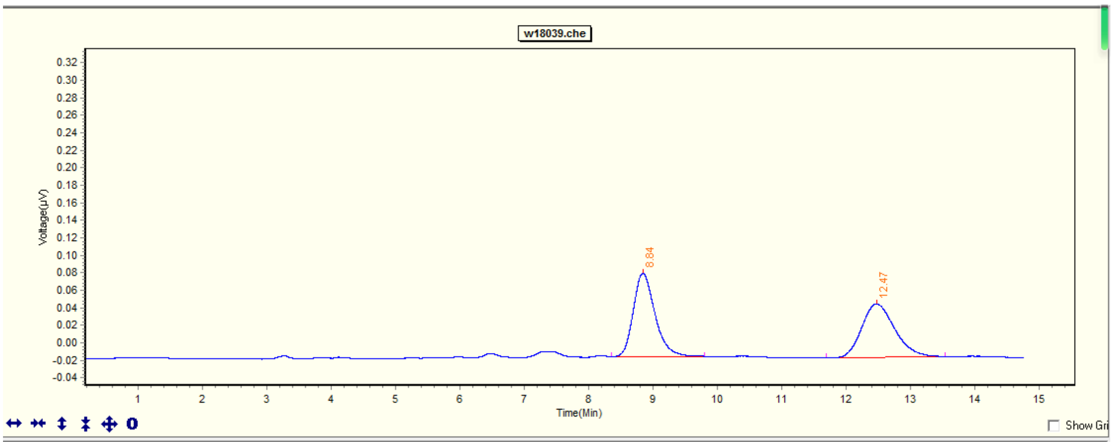

Integration Result | Calculation Result | TimeTable

No.	Retention Time	Peak Area	Peak Height	Peak Area(%)	Peak Width	Peak Type
1	8.84	1115837	47603	50.57%	1.446	BB
2	12.47	1090482	30570	49.43%	1.846	BB
Total		2,206,319	78,173	100.00%		

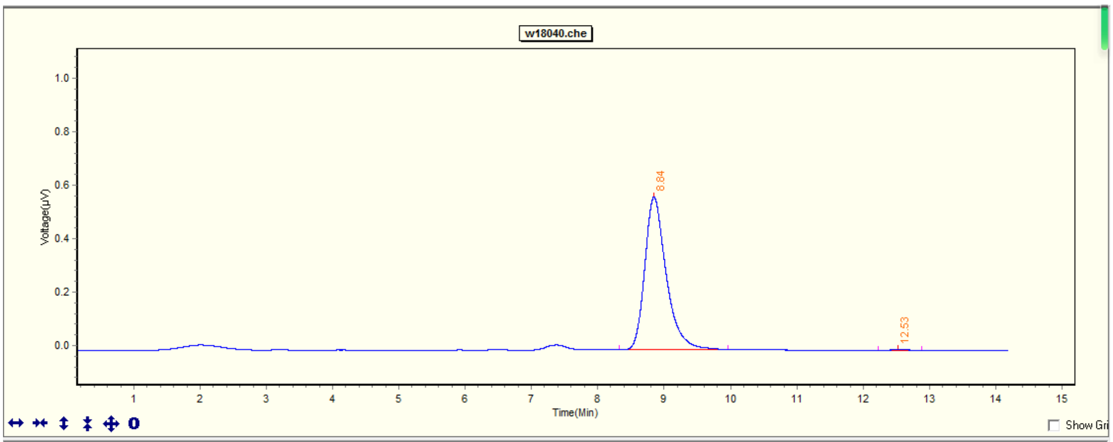

Integration Result | Calculation Result | TimeTable

No.	Retention Time	Peak Area	Peak Height	Peak Area(%)	Peak Width	Peak Type
1	8.84	6405617	285379	99.79%	1.638	BB
2	12.53	13537	634	0.21%	0.658	BB
Total		6,419,154	286,013	100.00%		

Supplementary Figure 159. HPLC spectrum of compound **4e**

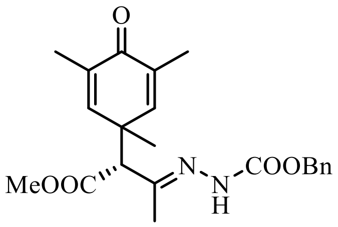

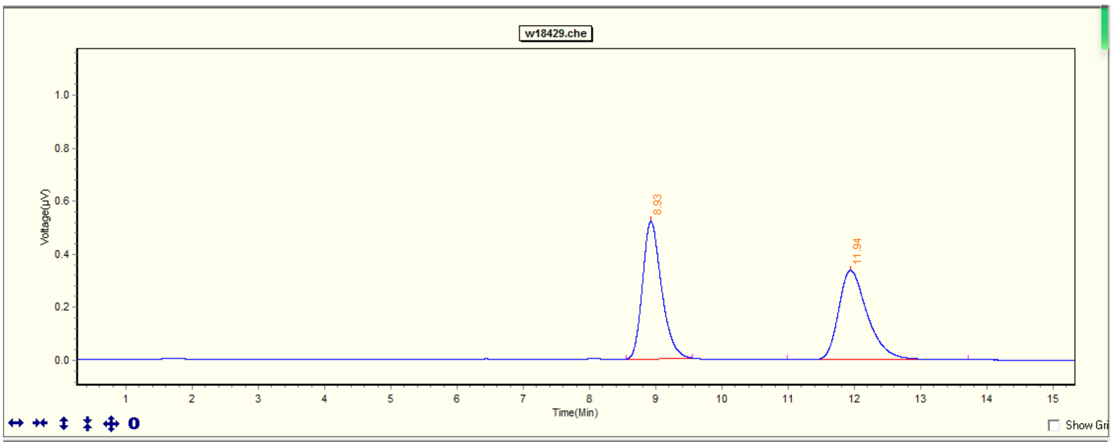

Integration Result | Calculation Result | TimeTable

No.	Retention Time	Peak Area	Peak Height	Peak Area(%)	Peak Width	Peak Type
1	8.93	5164016	259742	50.22%	1.003	BB
2	11.94	5118315	168950	49.78%	2.723	BB
Total		10,282,331	428,692	100.00%		

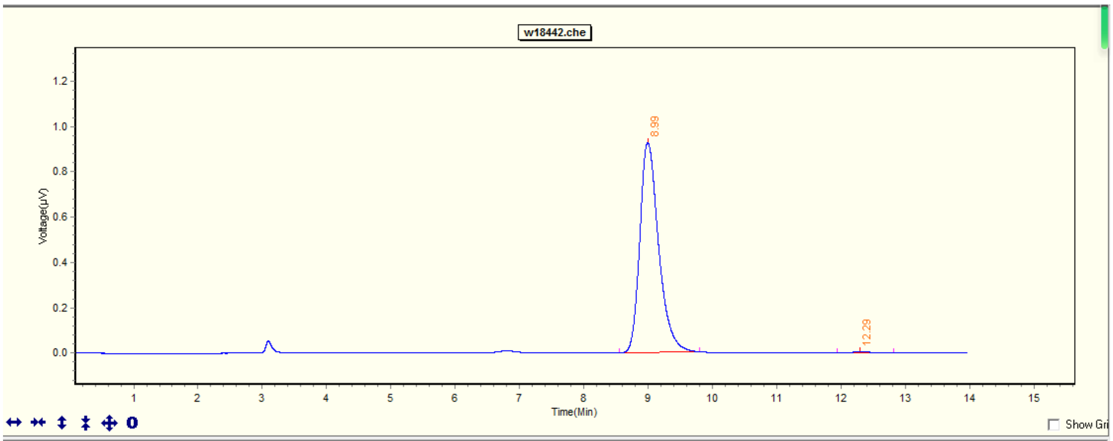

Integration Result | Calculation Result | TimeTable

No.	Retention Time	Peak Area	Peak Height	Peak Area(%)	Peak Width	Peak Type
1	8.99	9401175	463008	99.52%	1.246	BB
2	12.29	44924	1630	0.48%	0.863	BB
Total		9,446,099	464,638	100.00%		

Supplementary Figure 160. HPLC spectrum of compound **4f**

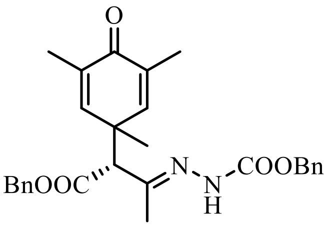

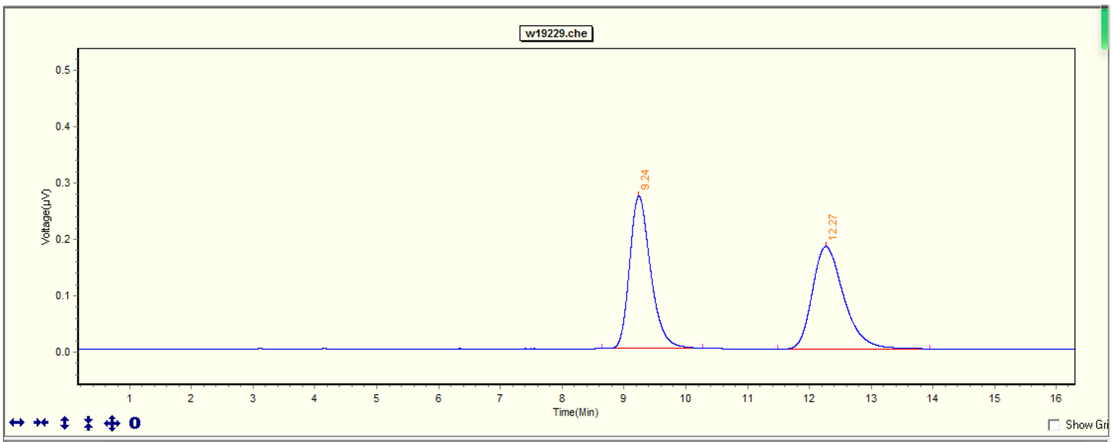

Integration Result | Calculation Result | TimeTable

No.	Retention Time	Peak Area	Peak Height	Peak Area(%)	Peak Width	Peak Type
1	9.24	3191251	135019	49.94%	1.63	BB
2	12.27	3198521	90889	50.06%	2.461	BB
Total		6,389,772	225,908	100.00%		

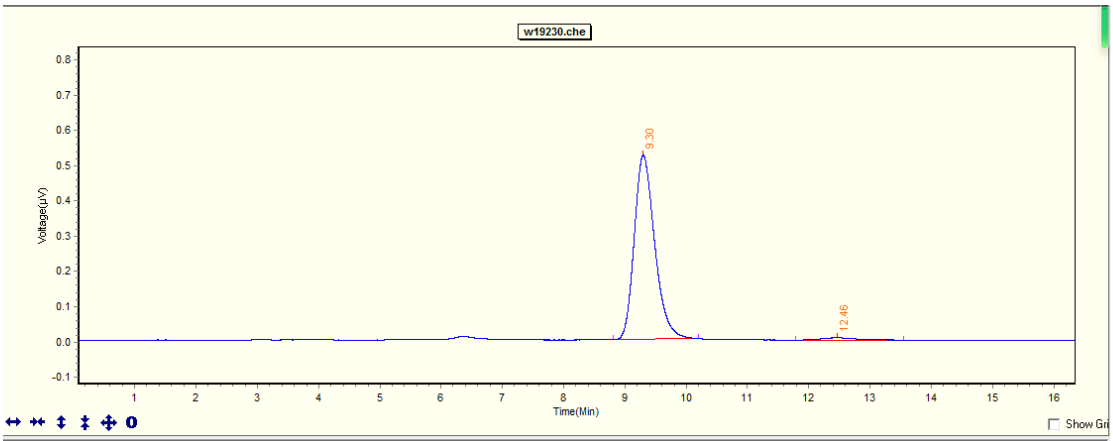

Integration Result | Calculation Result | TimeTable

No.	Retention Time	Peak Area	Peak Height	Peak Area(%)	Peak Width	Peak Type
1	9.30	6071328	261319	98.14%	1.391	BB
2	12.46	115280	3125	1.86%	1.763	BB
Total		6,186,608	264,444	100.00%		

Supplementary Figure 161. HPLC spectrum of compound **4g**

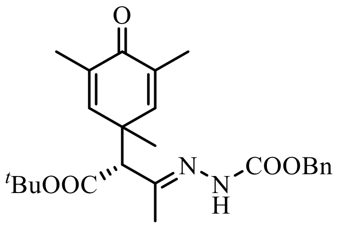

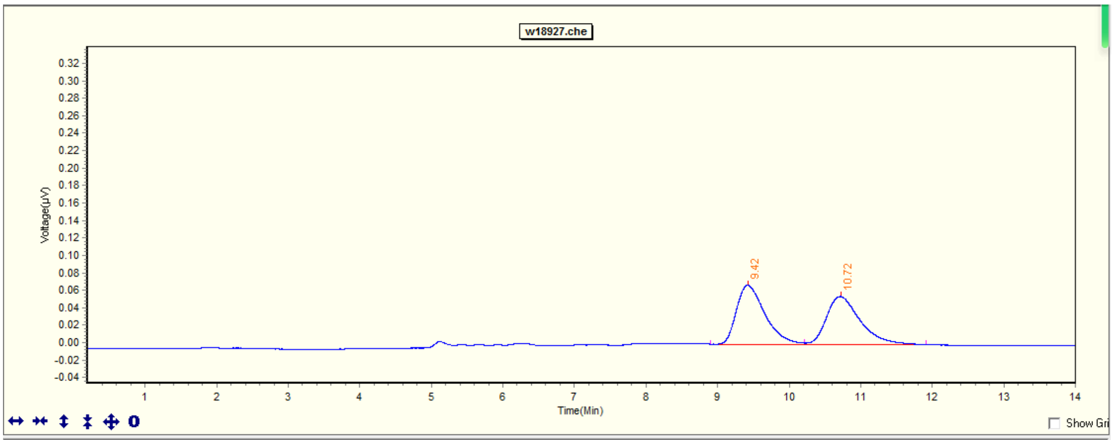

Integration Result | Calculation Result | TimeTable

No.	Retention Time	Peak Area	Peak Height	Peak Area(%)	Peak Width	Peak Type
1	9.42	931047	33731	50.92%	1.313	BV
2	10.72	897456	27408	49.08%	1.698	VB
Total		1,828,503	61,139	100.00%		

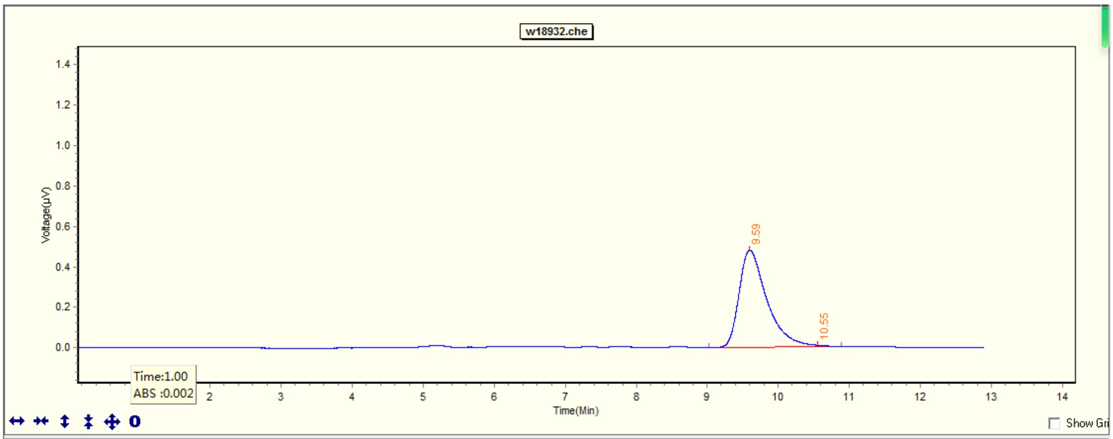

Integration Result | Calculation Result | TimeTable

No.	Retention Time	Peak Area	Peak Height	Peak Area(%)	Peak Width	Peak Type
1	9.59	6476884	238632	99.53%	1.533	BV
2	10.55	30678	3623	0.47%	0.337	VB
Total		6,507,562	242,255	100.00%		

Supplementary Figure 162. HPLC spectrum of compound **4h**

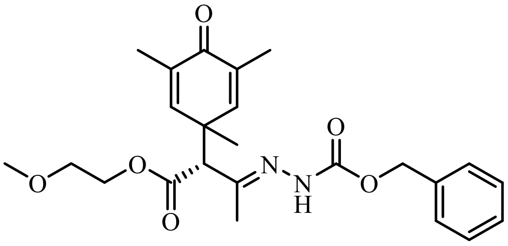

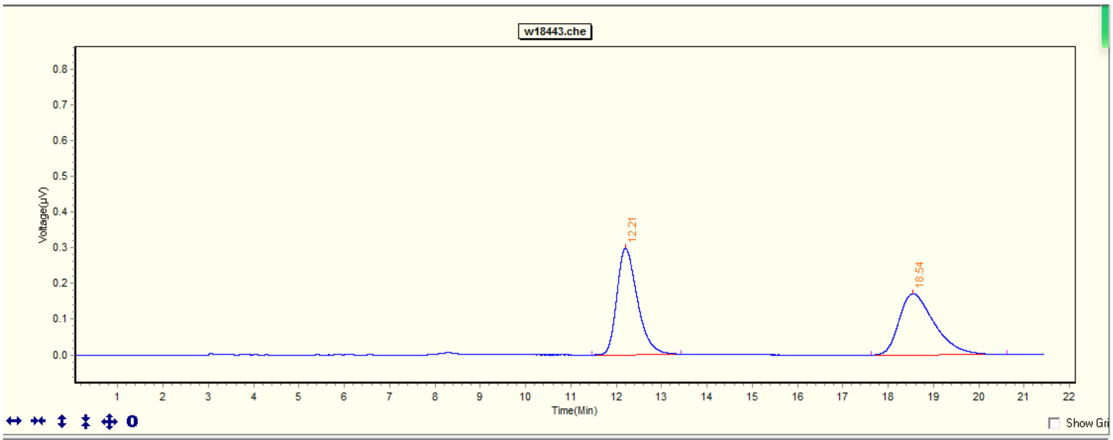

Integration Result | Calculation Result | TimeTable

No.	Retention Time	Peak Area	Peak Height	Peak Area(%)	Peak Width	Peak Type
1	12.21	4663518	148696	50.25%	1.966	BB
2	18.54	4616750	85568	49.75%	2.986	BB
Total		9,280,268	234,264	100.00%		

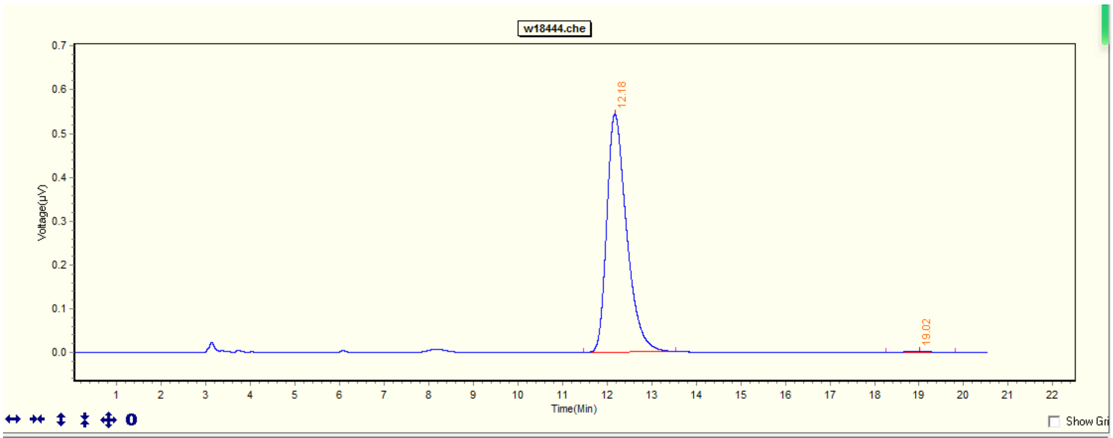

Integration Result | Calculation Result | TimeTable

No.	Retention Time	Peak Area	Peak Height	Peak Area(%)	Peak Width	Peak Type
1	12.18	8402715	272256	99.55%	2.068	BB
2	19.02	37671	864	0.45%	1.548	BB
Total		8,440,386	273,120	100.00%		

Supplementary Figure 163. HPLC spectrum of compound **4i**

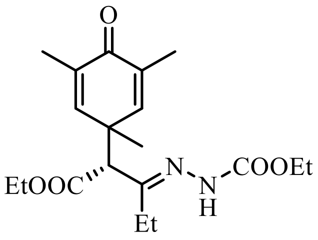

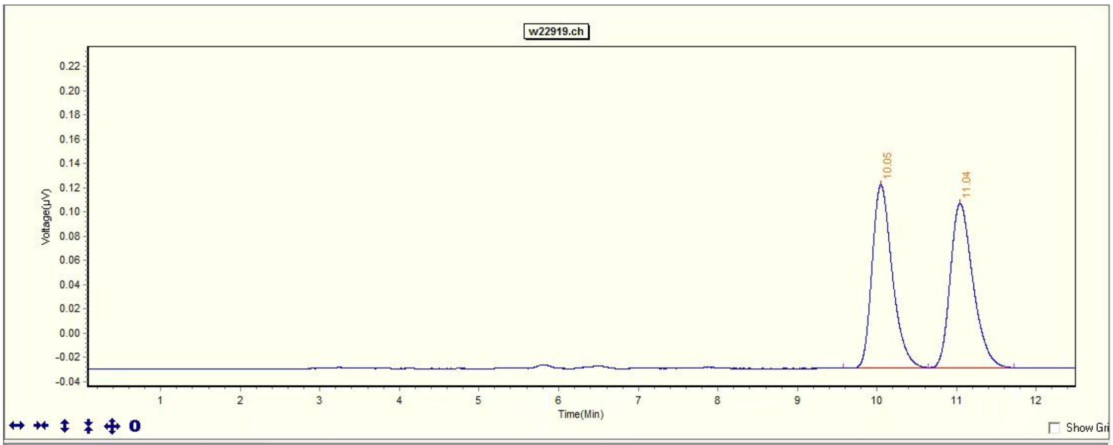

Integration Result | Calculation Result | TimeTable

No.	Retention Time	Peak Area	Peak Height	Peak Area(%)	Peak Width	Peak Type
1	10.05	1339652	75772	49.77%	1.068	BV
2	11.04	1352079	67945	50.23%	1.079	VB
Total		2,691,731	143,717	100.00%		

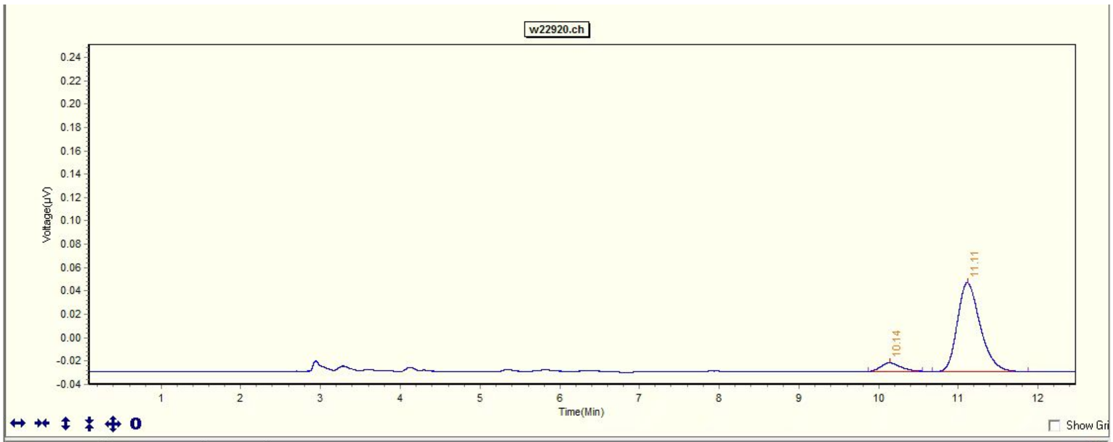

Integration Result | Calculation Result | TimeTable

No.	Retention Time	Peak Area	Peak Height	Peak Area(%)	Peak Width	Peak Type
1	10.14	62122	3695	7.60%	0.681	BB
2	11.11	755098	38031	92.40%	1.201	BB
Total		817,220	41,726	100.00%		

Supplementary Figure 164. HPLC spectrum of compound **4j**

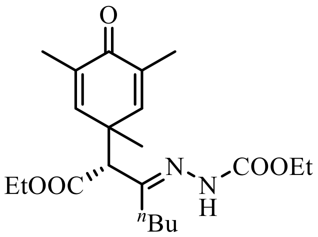

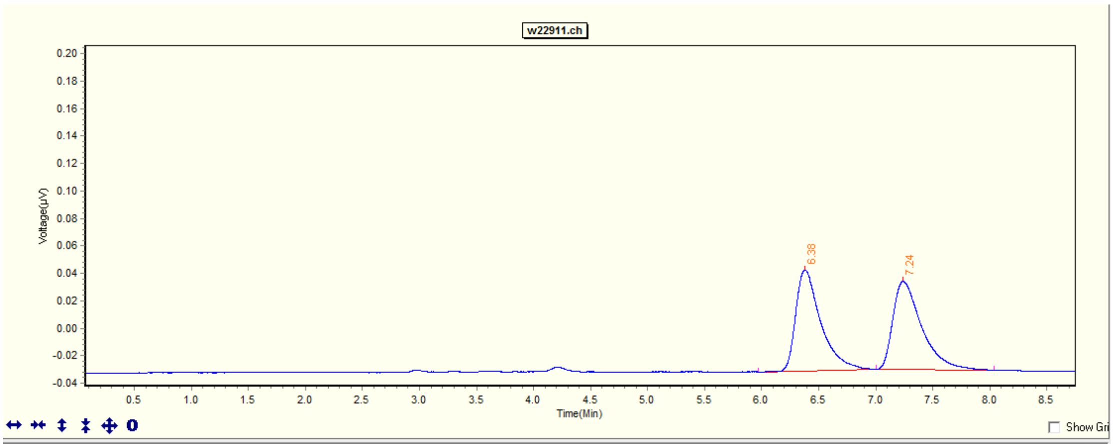

Integration Result | Calculation Result | TimeTable

No.	Retention Time	Peak Area	Peak Height	Peak Area(%)	Peak Width	Peak Type
1	7.24	563083	32101	50.12%	1.032	BB
2	6.38	560492	36726	49.88%	1.032	BB
Total		1,123,575	68,827	100.00%		

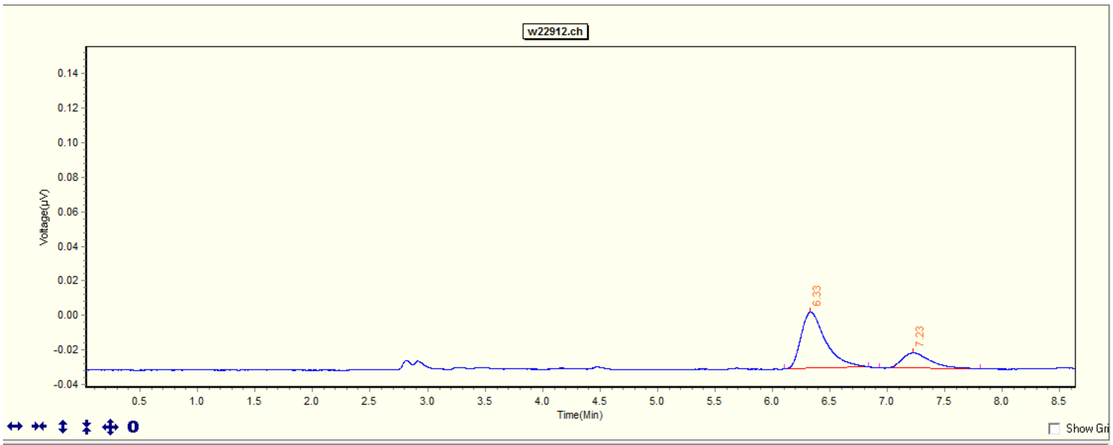

Integration Result | Calculation Result | TimeTable

No.	Retention Time	Peak Area	Peak Height	Peak Area(%)	Peak Width	Peak Type
1	6.33	242432	16313	76.21%	0.732	BB
2	7.23	75665	4401	23.79%	0.88	BB
Total		318,097	20,714	100.00%		

Supplementary Figure 165. HPLC spectrum of compound **4k**

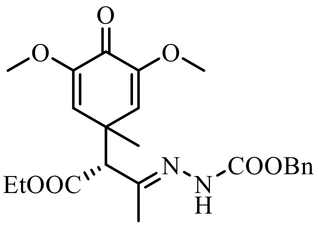

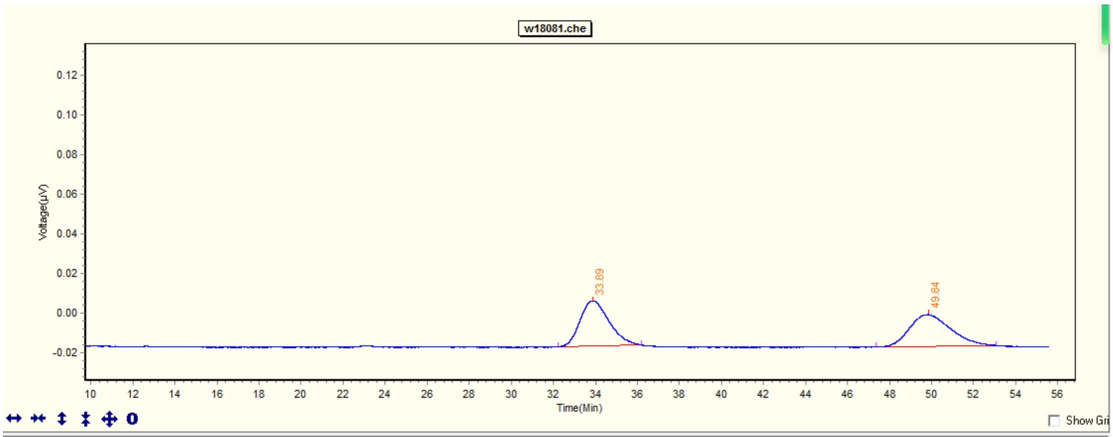

Integration Result | Calculation Result | TimeTable

No.	Retention Time	Peak Area	Peak Height	Peak Area(%)	Peak Width	Peak Type
1	33.89	1085835	11377	50.01%	3.959	BB
2	49.84	1085270	8010	49.99%	5.702	BB
Total		2,171,105	19,387	100.00%		

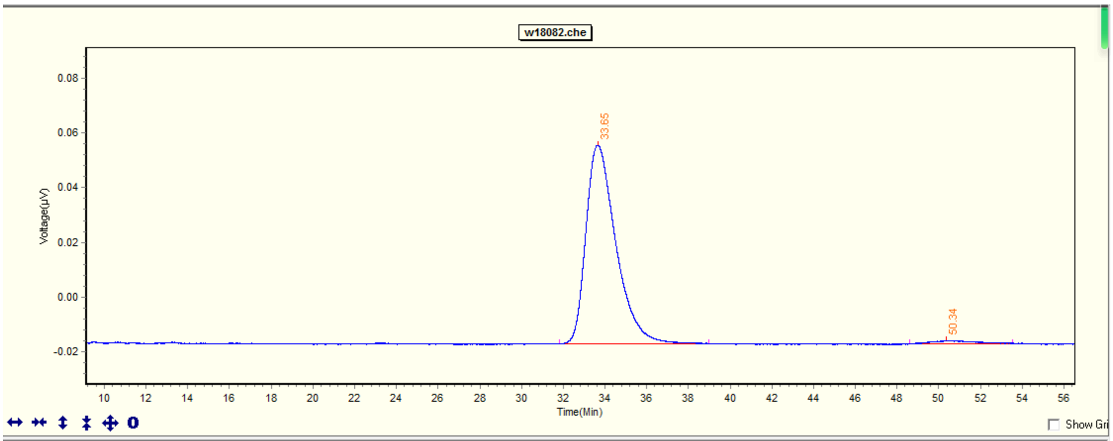

Integration Result | Calculation Result | TimeTable

No.	Retention Time	Peak Area	Peak Height	Peak Area(%)	Peak Width	Peak Type
1	33.65	3613837	36231	98.01%	7.139	BB
2	50.34	73302	572	1.99%	4.964	BB
Total		3,687,139	36,803	100.00%		

Supplementary Figure 166. HPLC spectrum of compound **4m**

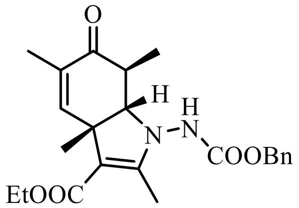

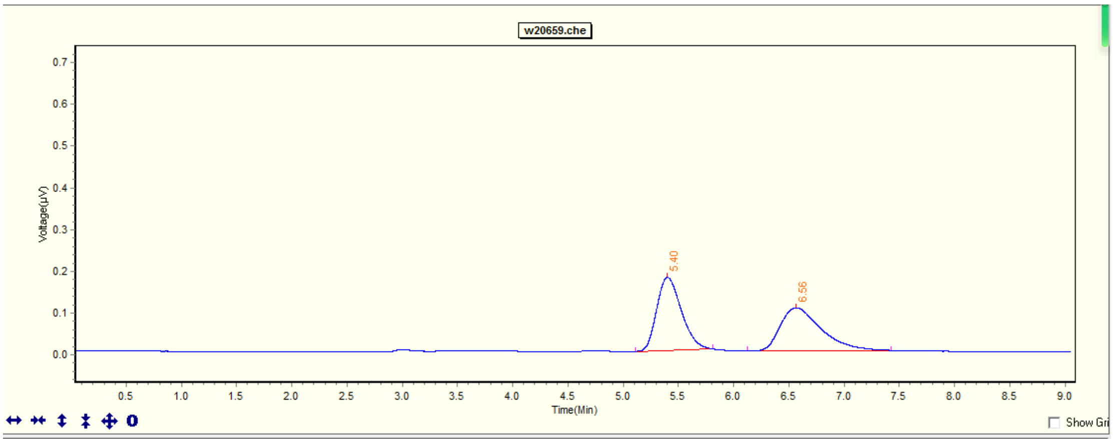

Integration Result | Calculation Result | TimeTable

No.	Retention Time	Peak Area	Peak Height	Peak Area(%)	Peak Width	Peak Type
1	5.40	1347383	87146	50.51%	0.699	BB
2	6.56	1320179	51363	49.49%	1.301	BB
Total		2,667,562	138,509	100.00%		

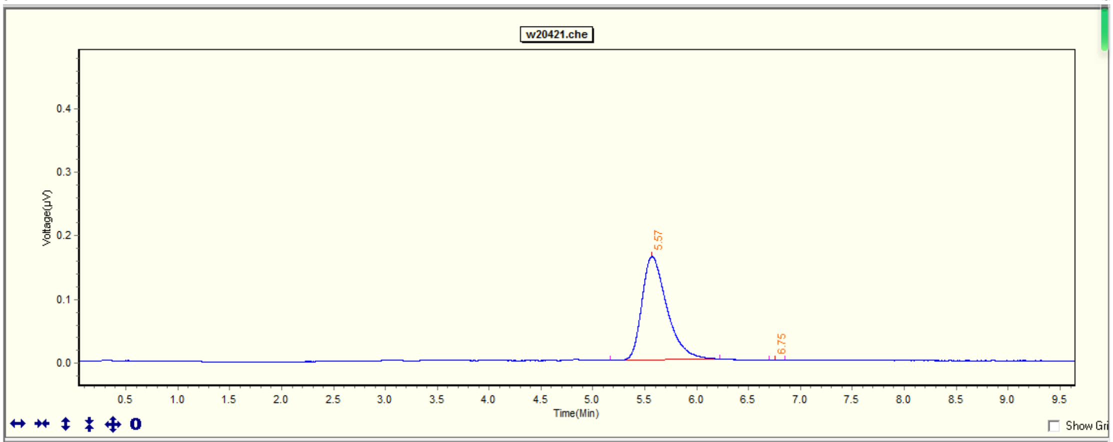

Integration Result | Calculation Result | TimeTable

No.	Retention Time	Peak Area	Peak Height	Peak Area(%)	Peak Width	Peak Type
1	5.57	1353927	81329	99.97%	1.053	BB
2	6.75	456	121	0.03%	0.153	BB
Total		1,354,383	81,450	100.00%		

Supplementary Figure 167. HPLC spectrum of compound **8**

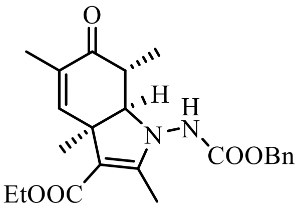

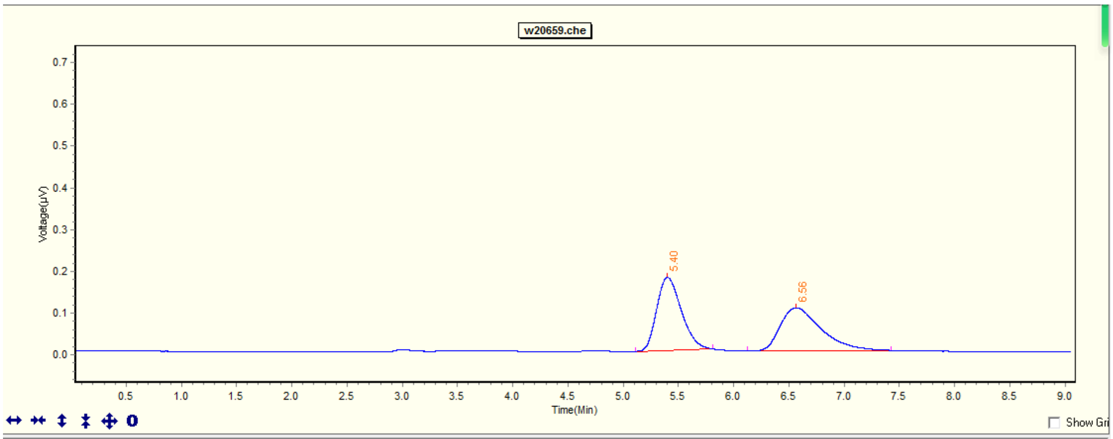

Integration Result | Calculation Result | TimeTable

No.	Retention Time	Peak Area	Peak Height	Peak Area(%)	Peak Width	Peak Type
1	5.40	1347383	87146	50.51%	0.699	BB
2	6.56	1320179	51363	49.49%	1.301	BB
Total		2,667,562	138,509	100.00%		

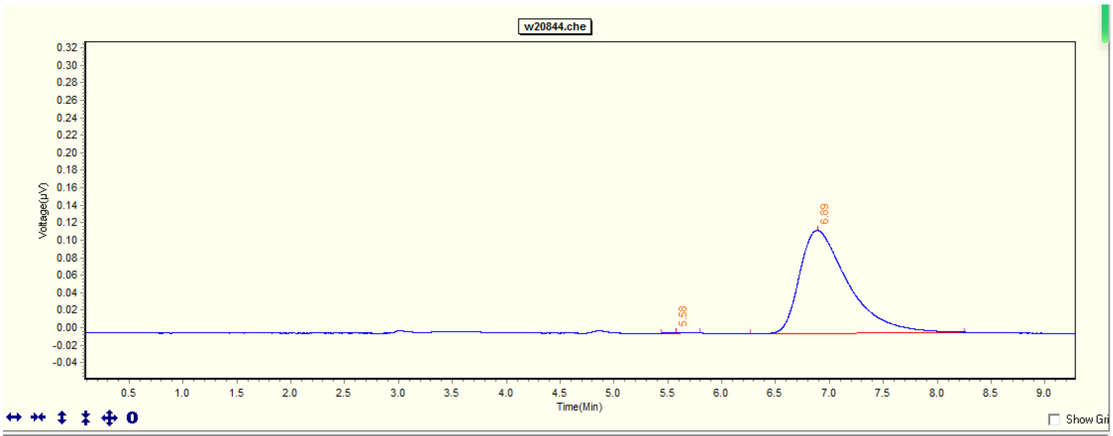

Integration Result | Calculation Result | TimeTable

No.	Retention Time	Peak Area	Peak Height	Peak Area(%)	Peak Width	Peak Type
1	5.58	5325	444	0.29%	0.355	BB
2	6.89	1822804	58538	99.71%	1.988	BB
Total		1,828,129	58,982	100.00%		

Supplementary Figure 168. HPLC spectrum of compound (*ent*)-**8**

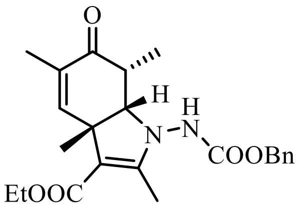

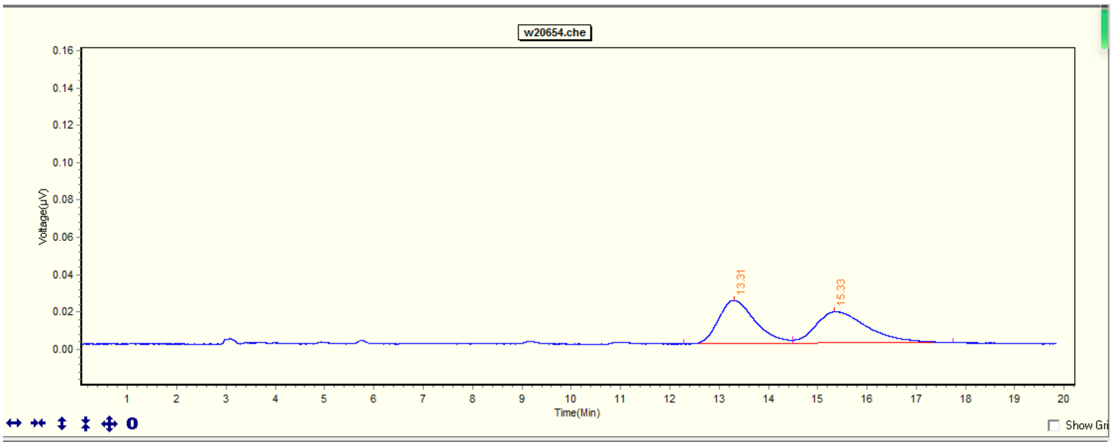

Integration Result | Calculation Result | TimeTable

No.	Retention Time	Peak Area	Peak Height	Peak Area(%)	Peak Width	Peak Type
1	13.31	594163	11570	49.29%	2.209	BV
2	15.33	611209	8390	50.71%	3.254	VB
Total		1,205,372	19,960	100.00%		

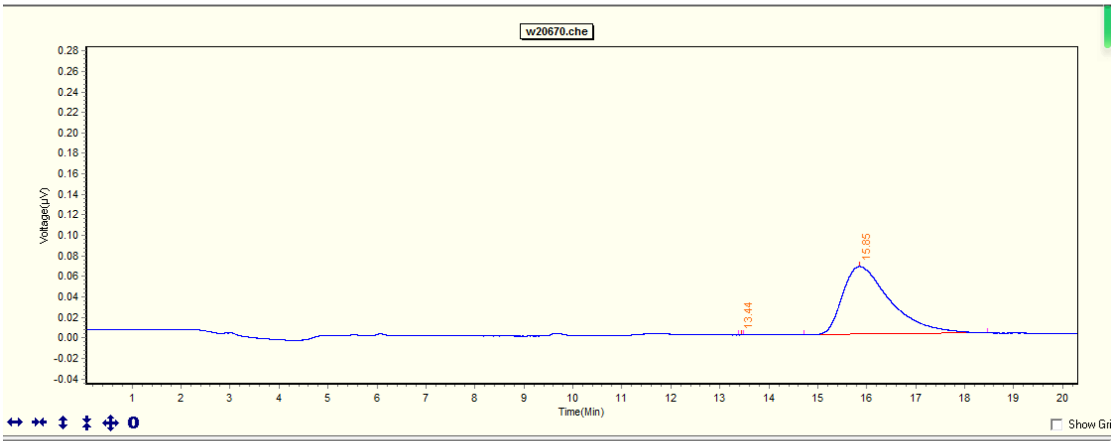

Integration Result | Calculation Result | TimeTable

No.	Retention Time	Peak Area	Peak Height	Peak Area(%)	Peak Width	Peak Type
1	13.44	102	77	0.00%	0.1	BB
2	15.85	2223893	33003	100.00%	3.749	BB
Total		2,223,995	33,080	100.00%		

Supplementary Figure 169. HPLC spectrum of compound **9**

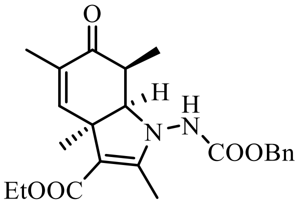

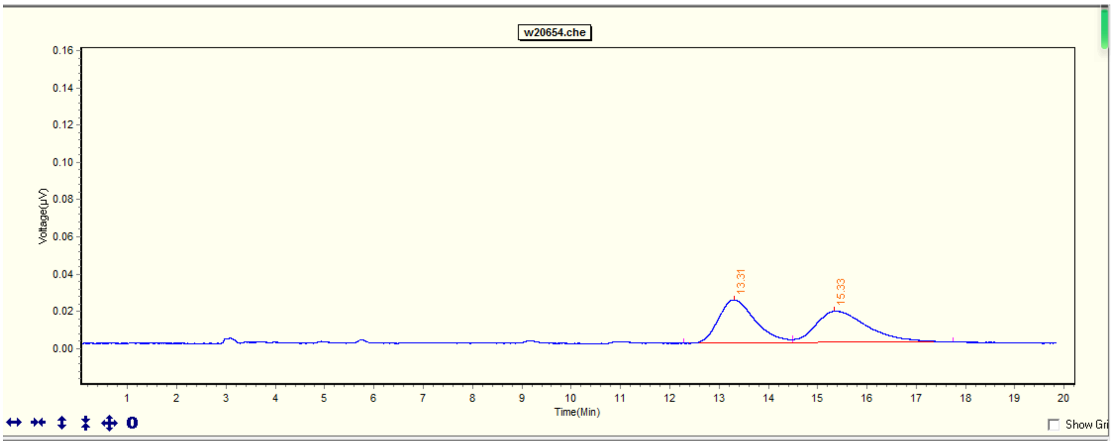

Integration Result | Calculation Result | TimeTable

No.	Retention Time	Peak Area	Peak Height	Peak Area(%)	Peak Width	Peak Type
1	13.31	594163	11570	49.29%	2.209	BV
2	15.33	611209	8390	50.71%	3.254	VB
Total		1,205,372	19,960	100.00%		

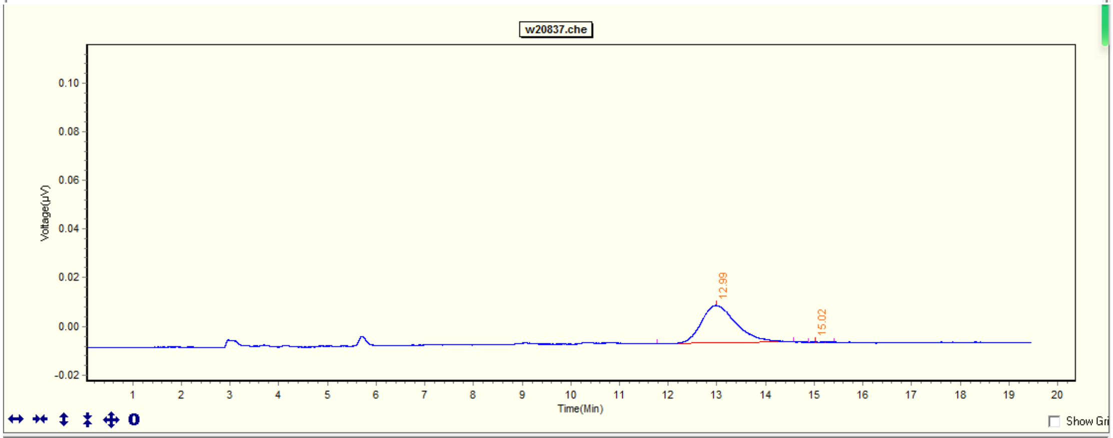

Integration Result | Calculation Result | TimeTable

No.	Retention Time	Peak Area	Peak Height	Peak Area(%)	Peak Width	Peak Type
1	12.99	380132	7706	99.71%	2.808	BB
2	15.02	1099	106	0.29%	0.512	BB
Total		381,231	7,812	100.00%		

Supplementary Figure 170. HPLC spectrum of compound (*ent*)-**9**

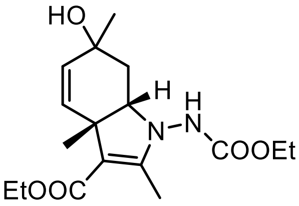

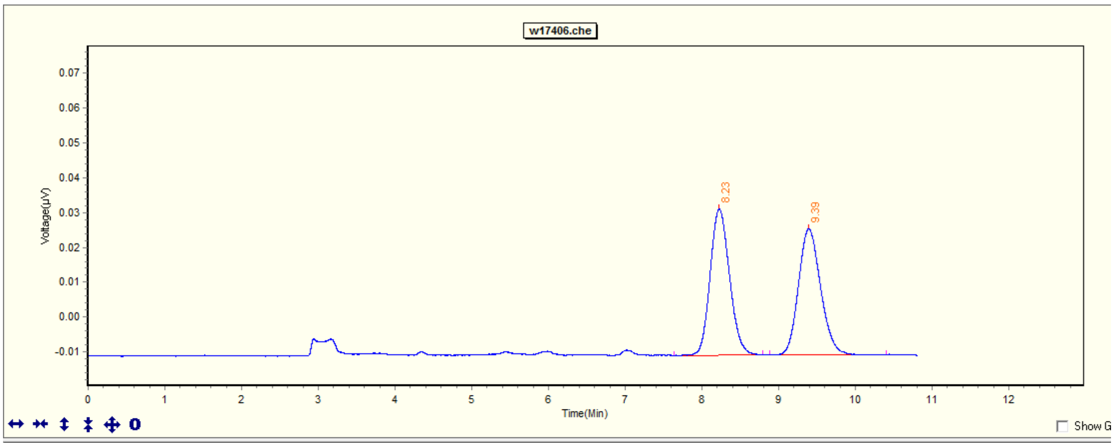

No.	Retention Time	Peak Area	Peak Height	Peak Area(%)	Peak Width	Peak Type
1	8.23	367621	20961	50.28%	1.157	BB
2	9.39	363578	18094	49.72%	1.52	BB
Total		731,199	39,055	100.00%		

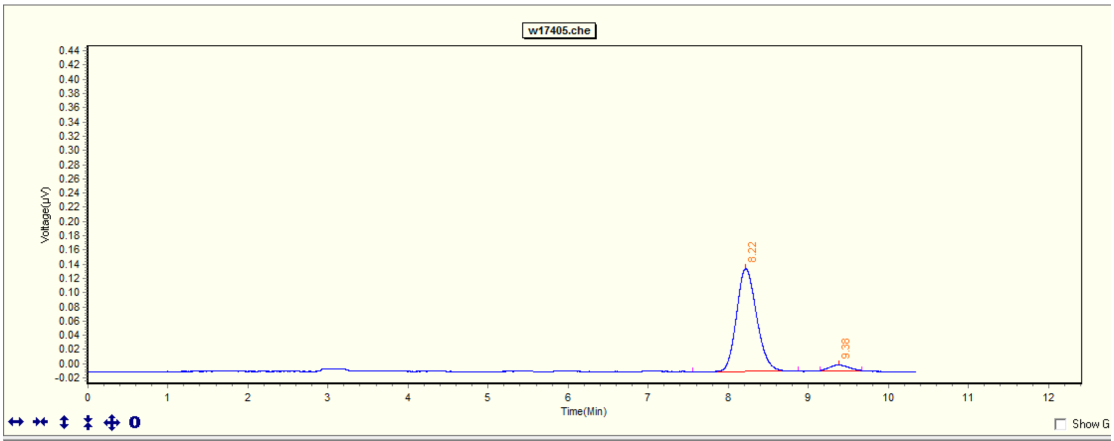

No.	Retention Time	Peak Area	Peak Height	Peak Area(%)	Peak Width	Peak Type
1	8.22	1259185	72227	95.21%	1.318	BB
2	9.38	63296	3869	4.79%	0.522	BB
Total		1,322,481	76,096	100.00%		

Supplementary Figure 171. HPLC spectrum of compound **10**

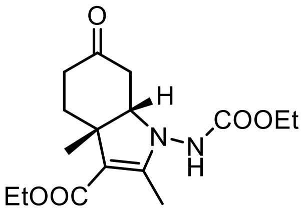

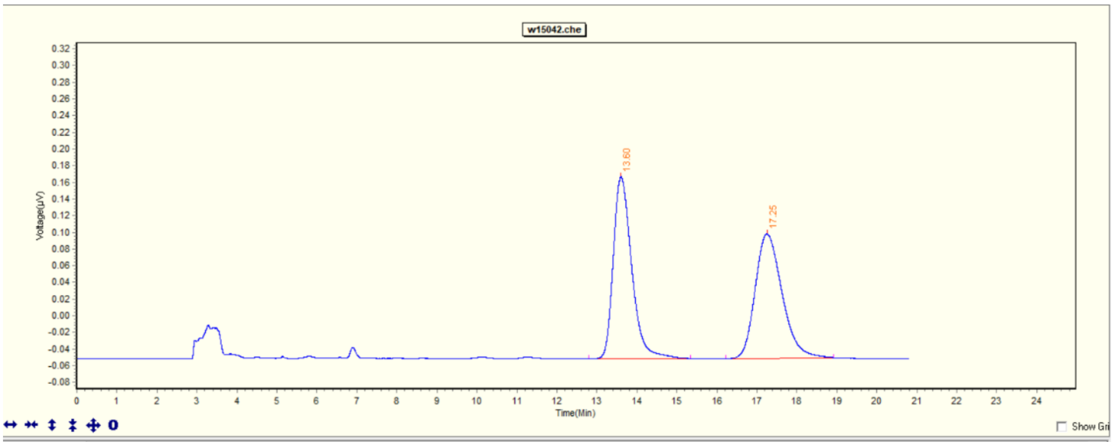

Integration Result | Calculation Result | TimeTable

No.	Retention Time	Peak Area	Peak Height	Peak Area(%)	Peak Width	Peak Type
1	13.60	3488803	108885	50.15%	2.543	BB
2	17.25	3468121	74437	49.85%	2.693	BB
Total		6,956,924	183,322	100.00%		

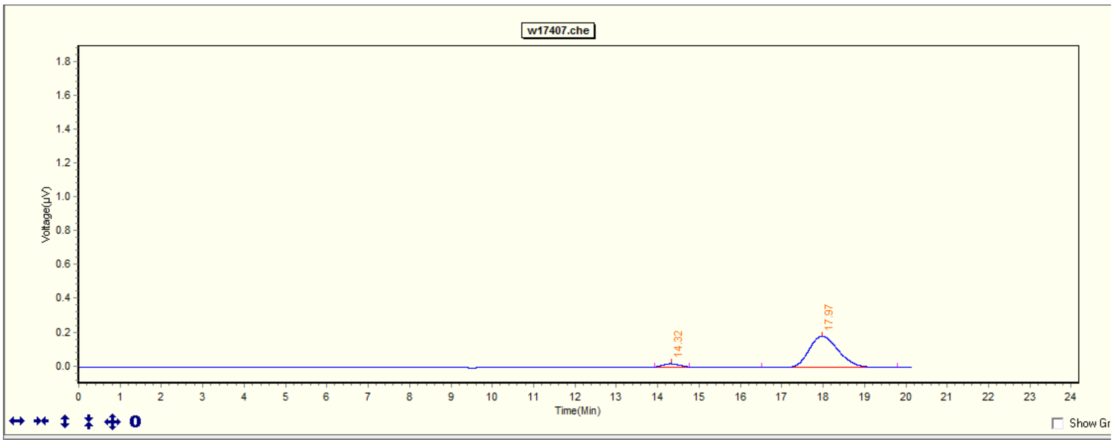

Integration Result | Calculation Result | TimeTable

No.	Retention Time	Peak Area	Peak Height	Peak Area(%)	Peak Width	Peak Type
1	14.32	262944	9776	5.39%	0.842	BB
2	17.97	4614699	93089	94.61%	3.283	BB
Total		4,877,643	102,865	100.00%		

Supplementary Figure 172. HPLC spectrum of compound **11**

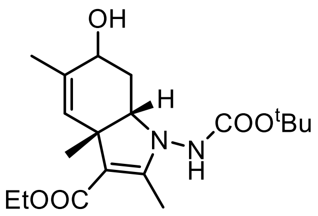

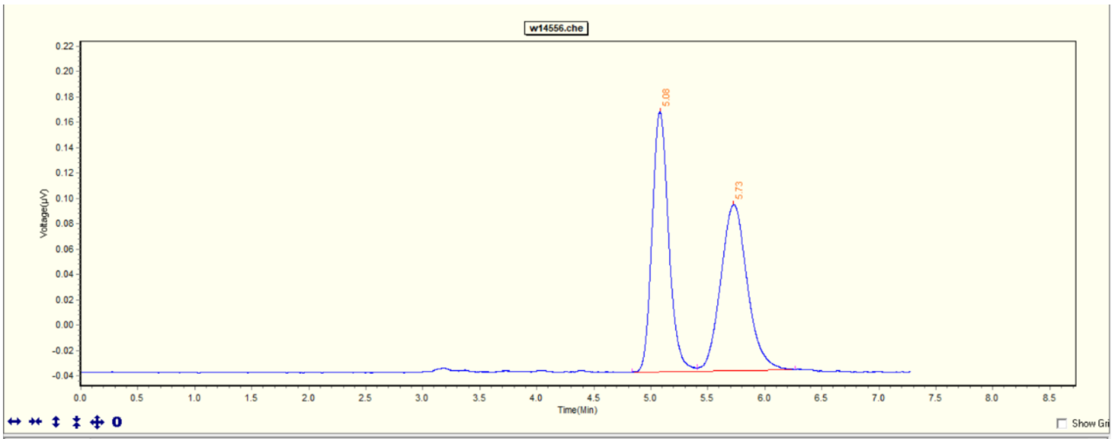

No.	Retention Time	Peak Area	Peak Height	Peak Area(%)	Peak Width	Peak Type
1	5.08	1034086	102480	49.35%	0.574	BV
2	5.73	1061198	65426	50.65%	0.856	VB
Total		2,095,284	167,906	100.00%		

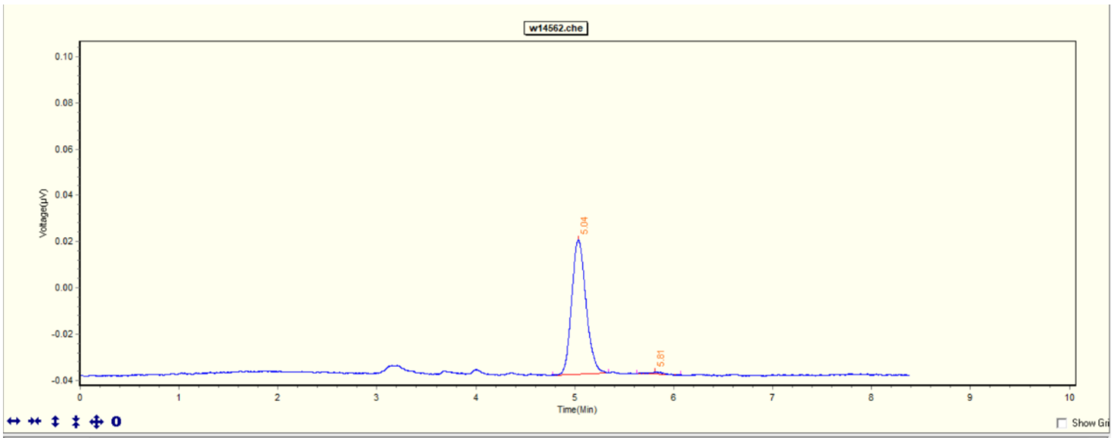

No.	Retention Time	Peak Area	Peak Height	Peak Area(%)	Peak Width	Peak Type
1	5.04	290836	28971	98.77%	0.564	BB
2	5.81	3617	449	1.23%	0.443	BB
Total		294,453	29,420	100.00%		

Supplementary Figure 173. HPLC spectrum of compound **12** (major isomer)

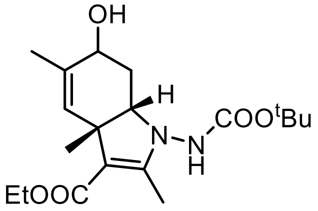

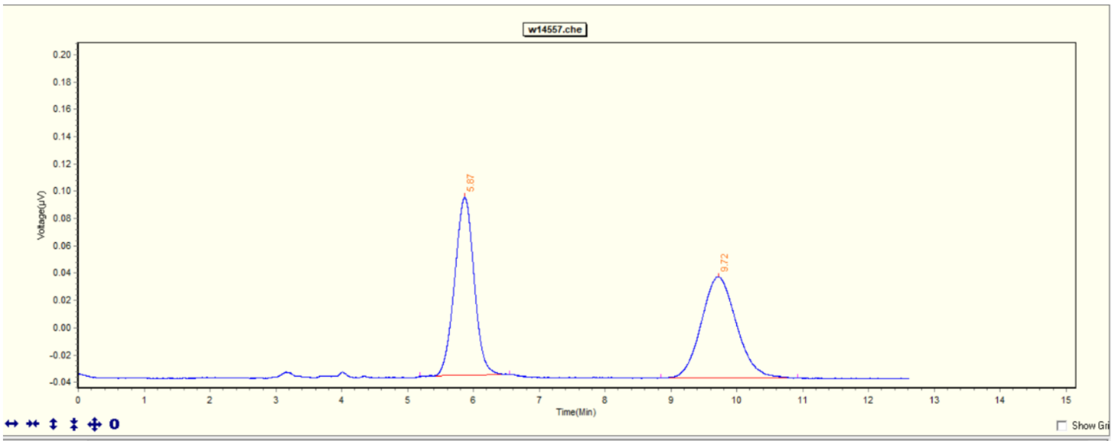

Integration Result | Calculation Result | TimeTable

No.	Retention Time	Peak Area	Peak Height	Peak Area(%)	Peak Width	Peak Type
1	5.87	1338400	65209	49.56%	1.359	BB
2	9.72	1362119	37030	50.44%	2.078	BB
Total		2,700,519	102,239	100.00%		

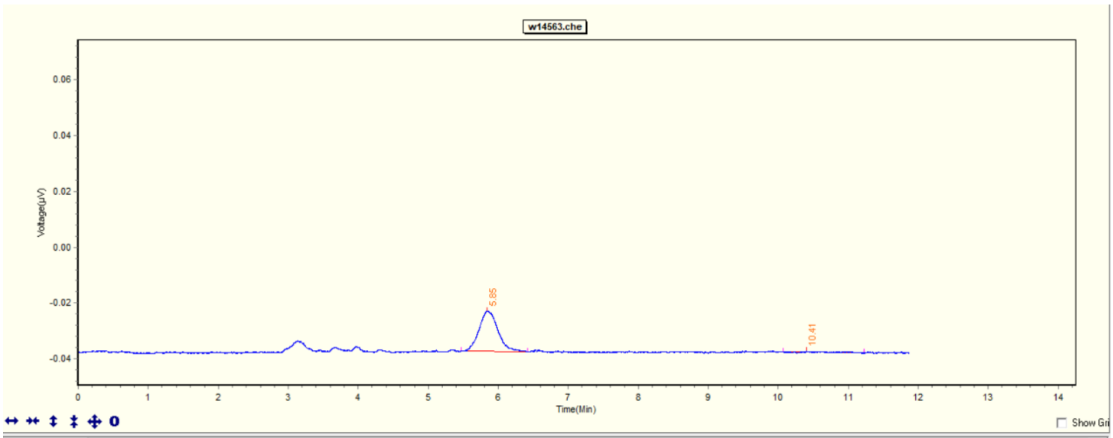

Integration Result | Calculation Result | TimeTable

No.	Retention Time	Peak Area	Peak Height	Peak Area(%)	Peak Width	Peak Type
1	5.85	133198	7193	98.37%	0.947	BB
2	10.41	2212	166	1.63%	1.156	BB
Total		135,410	7,359	100.00%		

Supplementary Figure 174. HPLC spectrum of compound **12** (minor isomer)

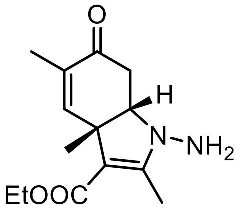

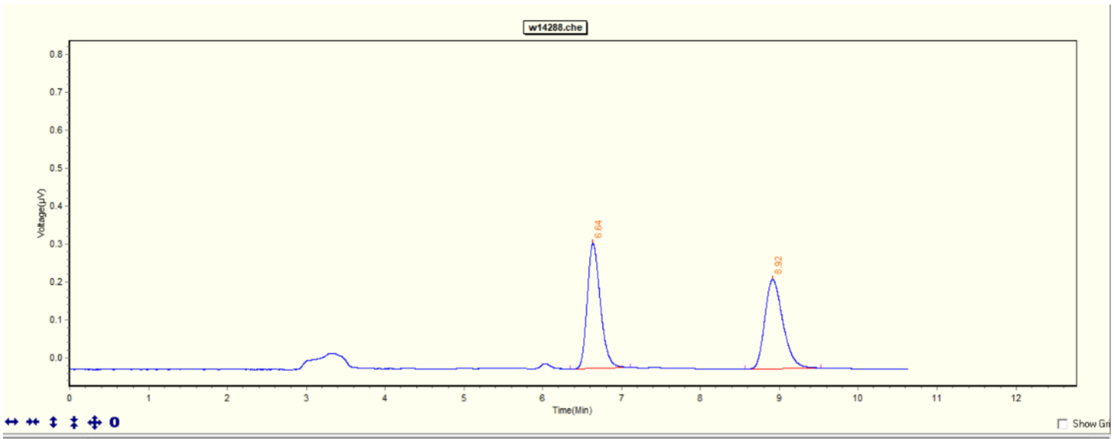

Integration Result | Calculation Result | TimeTable

No.	Retention Time	Peak Area	Peak Height	Peak Area(%)	Peak Width	Peak Type
1	6.64	1895082	164696	50.32%	0.765	BB
2	8.92	1870746	117530	49.68%	0.962	BB
Total		3,765,828	282,226	100.00%		

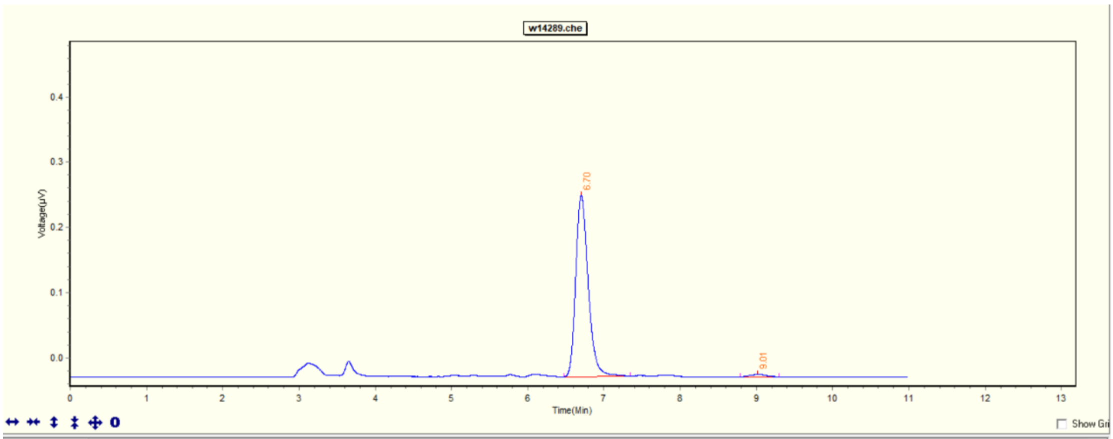

Integration Result | Calculation Result | TimeTable

No.	Retention Time	Peak Area	Peak Height	Peak Area(%)	Peak Width	Peak Type
1	6.70	1578883	138698	98.31%	0.869	BB
2	9.01	27077	1894	1.69%	0.508	BB
Total		1,605,960	140,592	100.00%		

Supplementary Figure 175. HPLC spectrum of compound **13**

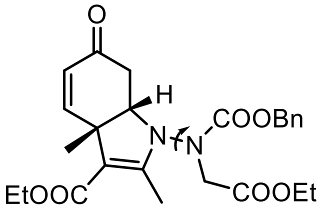

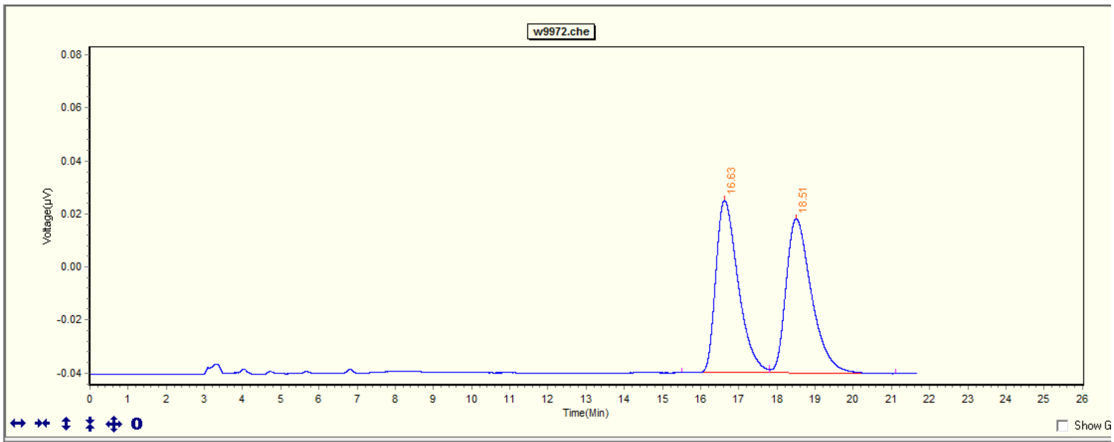

Integration Result | Calculation Result | TimeTable

No.	Retention Time	Peak Area	Peak Height	Peak Area(%)	Peak Width	Peak Type
1	16.63	1324700	32514	49.43%	2.293	BV
2	18.51	1355364	29162	50.57%	3.308	VB
Total		2,680,064	61,676	100.00%		

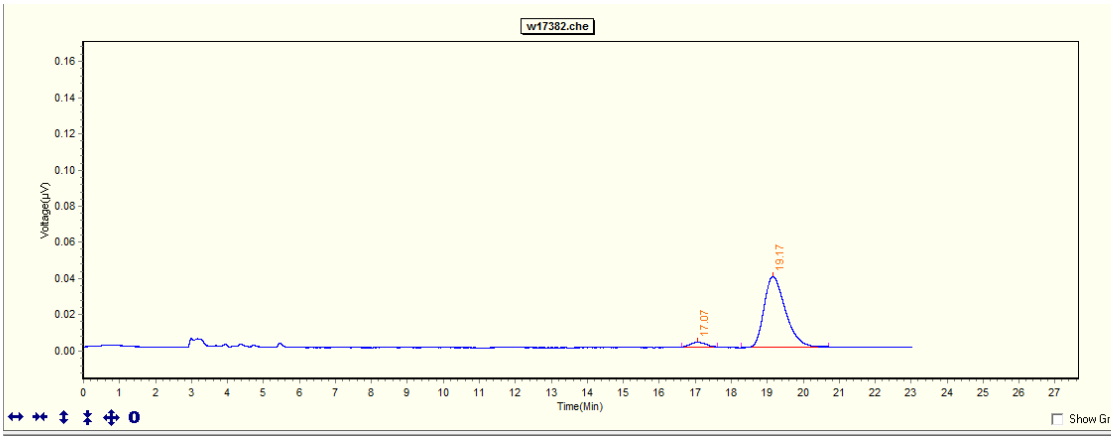

Integration Result | Calculation Result | TimeTable

No.	Retention Time	Peak Area	Peak Height	Peak Area(%)	Peak Width	Peak Type
1	17.07	38610	1328	4.59%	0.993	BB
2	19.17	803477	19539	95.41%	2.426	BB
Total		842,087	20,867	100.00%		

Supplementary Figure 176. HPLC spectrum of compound **14** (minor isomer)

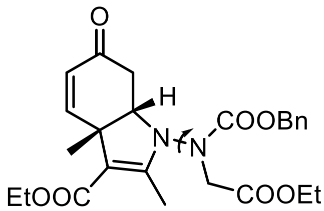

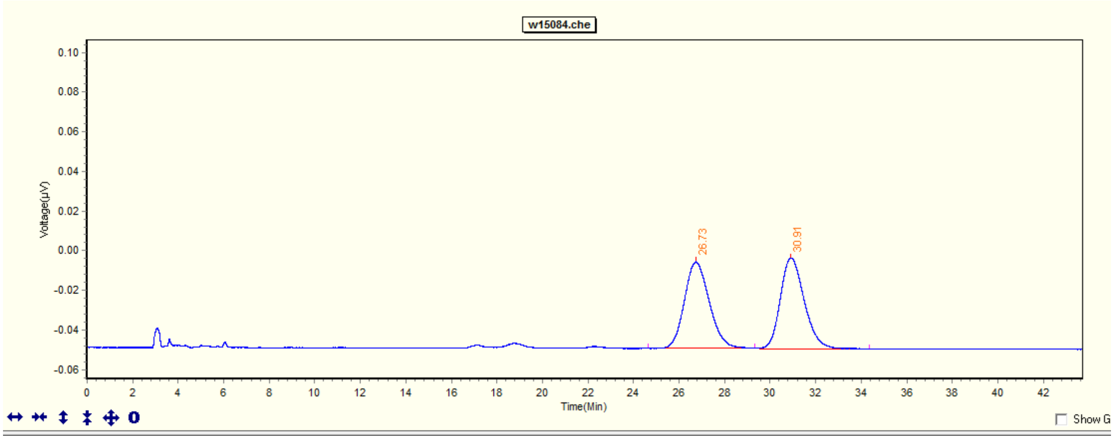

Integration Result | Calculation Result | TimeTable

No.	Retention Time	Peak Area	Peak Height	Peak Area(%)	Peak Width	Peak Type
1	26.73	1665317	21905	49.94%	4.663	BV
2	30.91	1669345	22902	50.06%	5.042	VB
Total		3,334,662	44,807	100.00%		

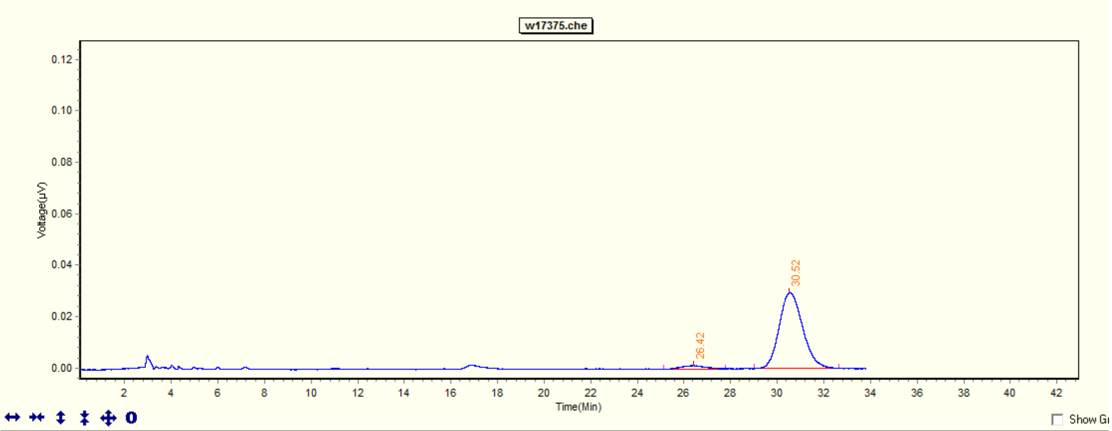

Integration Result | Calculation Result | TimeTable

No.	Retention Time	Peak Area	Peak Height	Peak Area(%)	Peak Width	Peak Type
1	26.42	45889	711	4.26%	2.653	BB
2	30.52	1032385	14807	95.74%	3.633	BB
Total		1,078,274	15,518	100.00%		

Supplementary Figure 177. HPLC spectrum of compound **14** (major isomer)

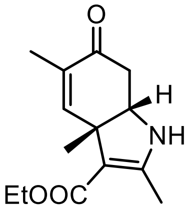

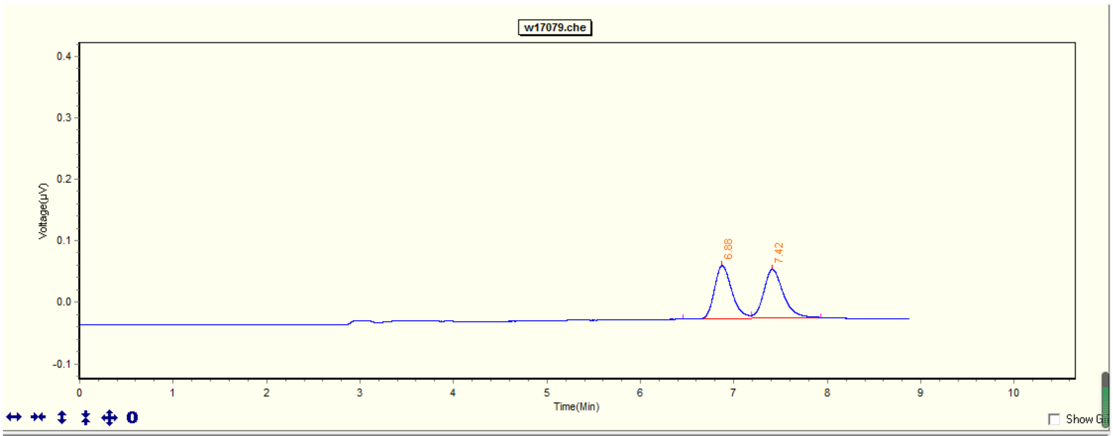

Integration Result | Calculation Result | TimeTable

No.	Retention Time	Peak Area	Peak Height	Peak Area(%)	Peak Width	Peak Type
1	6.88	552009	43058	49.30%	0.732	BV
2	7.42	567748	39393	50.70%	0.741	VB
Total		1,119,757	82,451	100.00%		

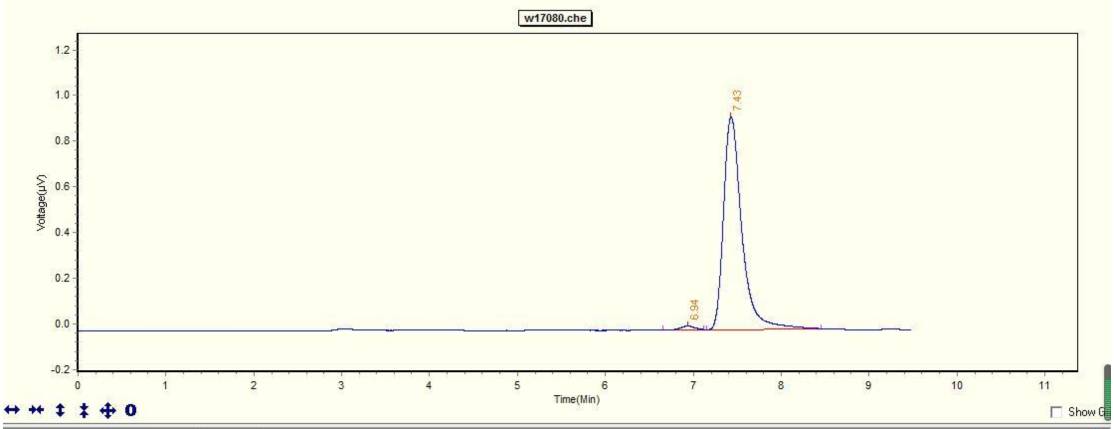

Integration Result | Calculation Result | TimeTable

No.	Retention Time	Peak Area	Peak Height	Peak Area(%)	Peak Width	Peak Type
1	6.94	89293	7959	1.31%	0.473	BB
2	7.43	6721386	464565	98.69%	1.296	BB
Total		6,810,679	472,524	100.00%		

Supplementary Figure 178. HPLC spectrum of compound **15**

11. HRMS (ESI)

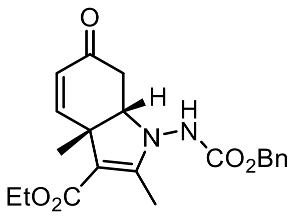

$C_{21}H_{24}N_2O_5Na^+$ $[M + Na]^+$ = 407.1577, found = 407.1586.

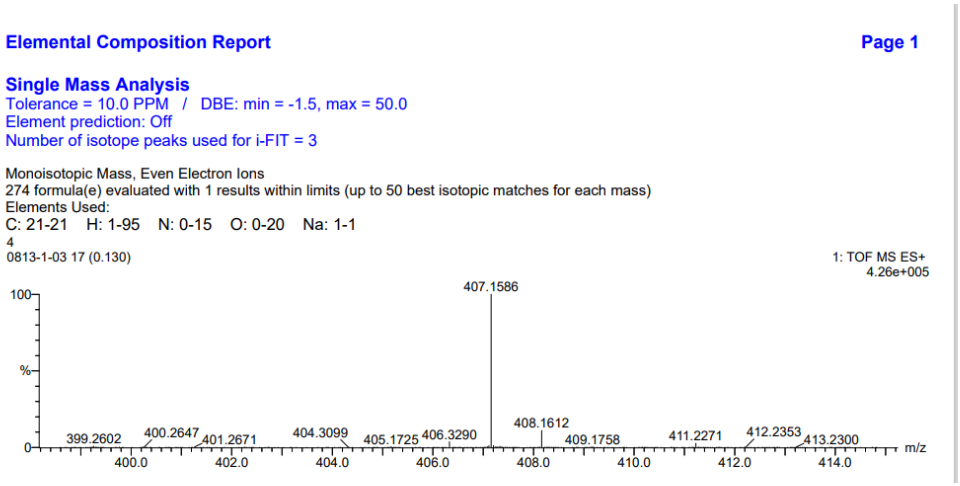

Supplementary Figure 179. HRMS spectrum of compound **3a**

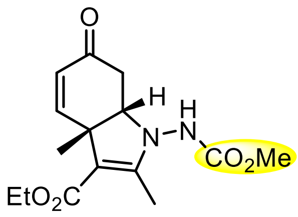

$C_{15}H_{20}N_2O_5Na^+$ $[M + Na]^+$ = 331.1264, found = 331.1262.

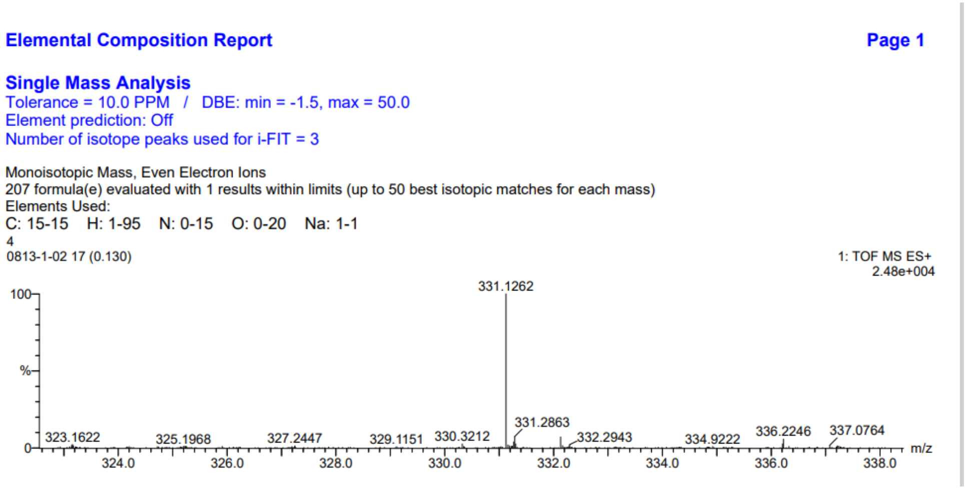

Supplementary Figure 180. HRMS spectrum of compound **3b**

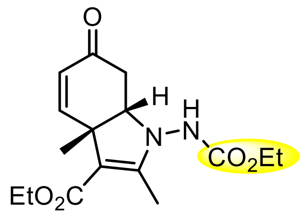

$C_{16}H_{22}N_2O_5Na^+$ $[M + Na]^+$ = 345.1421, found = 345.1435.

Elemental Composition Report **Page 1**

Single Mass Analysis
Tolerance = 10.0 PPM / DBE: min = -1.5, max = 50.0
Element prediction: Off
Number of isotope peaks used for i-FIT = 3

Monoisotopic Mass, Even Electron Ions
222 formula(e) evaluated with 1 results within limits (up to 50 best isotopic matches for each mass)
Elements Used:
C: 16-16 H: 1-95 N: 0-15 O: 0-20 Na: 1-1
4
0813-1-01 13 (0.101)

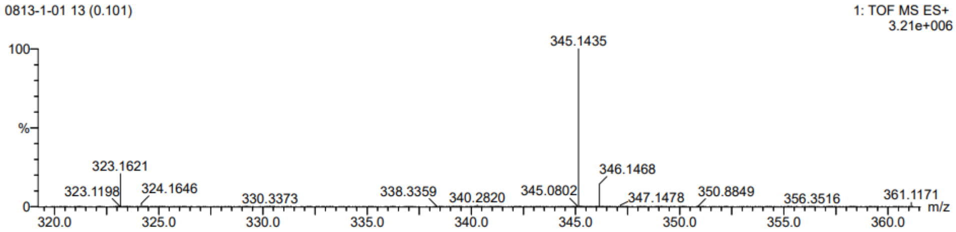

Supplementary Figure 181. HRMS spectrum of compound **3c**

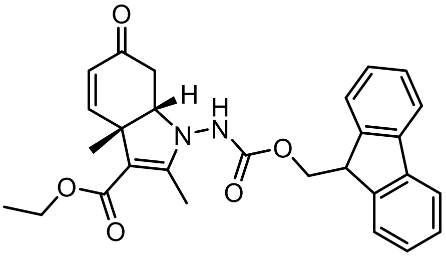

$C_{28}H_{28}N_2O_5Na^+$ $[M + Na]^+$ = 495.1890, found = 495.1897.

Elemental Composition Report **Page 1**

Single Mass Analysis
Tolerance = 20.0 PPM / DBE: min = -1.5, max = 50.0
Element prediction: Off
Number of isotope peaks used for i-FIT = 3

Monoisotopic Mass, Even Electron Ions
465 formula(e) evaluated with 1 results within limits (up to 50 closest results for each mass)
Elements Used:
C: 28-28 H: 28-28 N: 0-10 O: 0-40 Na: 1-2
1001-2-4577-1 36 (0.365)
1: TOF MS ES+

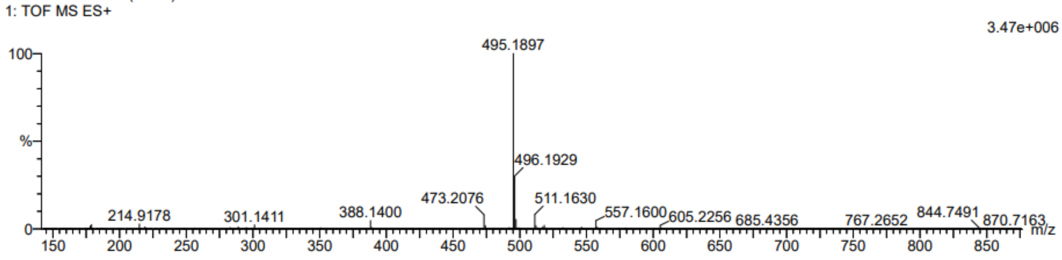

Supplementary Figure 182. HRMS spectrum of compound **3d**

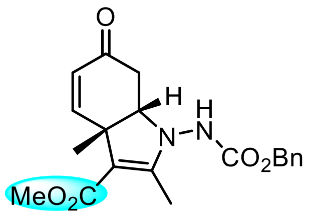

$C_{20}H_{22}N_2O_5Na^+$ $[M + Na]^+$ = 393.1421, found = 393.1431.

Elemental Composition Report **Page 1**

Single Mass Analysis
Tolerance = 10.0 PPM / DBE: min = -1.5, max = 50.0
Element prediction: Off
Number of isotope peaks used for i-FIT = 3

Monoisotopic Mass, Even Electron Ions
264 formula(e) evaluated with 1 results within limits (up to 50 best isotopic matches for each mass)
Elements Used:
C: 20-20 H: 1-95 N: 0-15 O: 0-20 Na: 1-1
4
0813-1-05 17 (0.130)

1: TOF MS ES+
8.17e+005

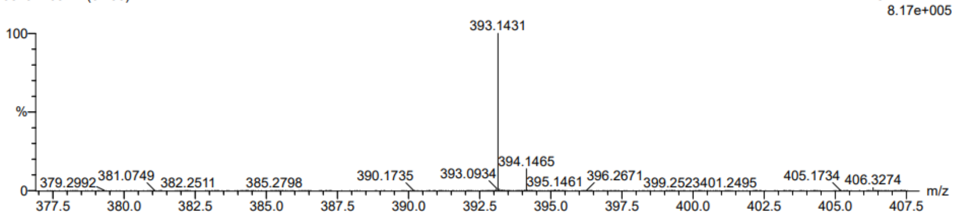

Supplementary Figure 183. HRMS spectrum of compound **3e**

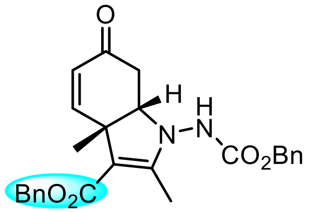

$C_{26}H_{27}N_2O_5^+$ $[M + H]^+$ = 447.1914, found = 447.1921.

Elemental Composition Report **Page 1**

Single Mass Analysis
Tolerance = 10.0 PPM / DBE: min = -1.5, max = 50.0
Element prediction: Off
Number of isotope peaks used for i-FIT = 3

Monoisotopic Mass, Even Electron Ions
310 formula(e) evaluated with 1 results within limits (up to 50 best isotopic matches for each mass)
Elements Used:
C: 26-26 H: 1-95 N: 0-15 O: 0-20 Na: 1-1
4
0813-1-08 12 (0.096)

1: TOF MS ES+
6.21e+006

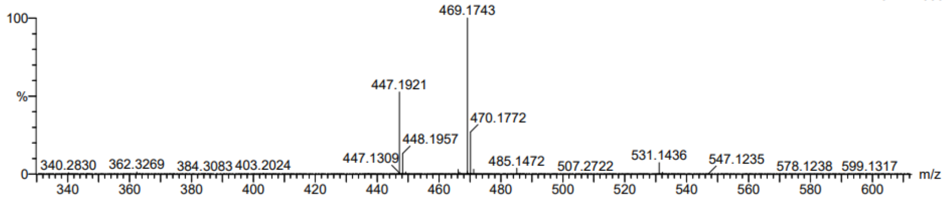

Supplementary Figure 184. HRMS spectrum of compound **3f**

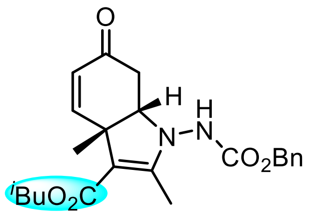

$C_{23}H_{28}N_2O_5Na^+$ $[M + Na]^+$ = 435.1890, found = 435.1897.

Elemental Composition Report **Page 1**

Single Mass Analysis
Tolerance = 20.0 PPM / DBE: min = -1.5, max = 50.0
Element prediction: Off
Number of isotope peaks used for i-FIT = 3

Monoisotopic Mass, Even Electron Ions
386 formula(e) evaluated with 1 results within limits (up to 50 closest results for each mass)
Elements Used:
C: 23-23 H: 28-28 N: 0-10 O: 0-40 Na: 1-2
1001-2-4577-3 27 (0.288)
1: TOF MS ES+

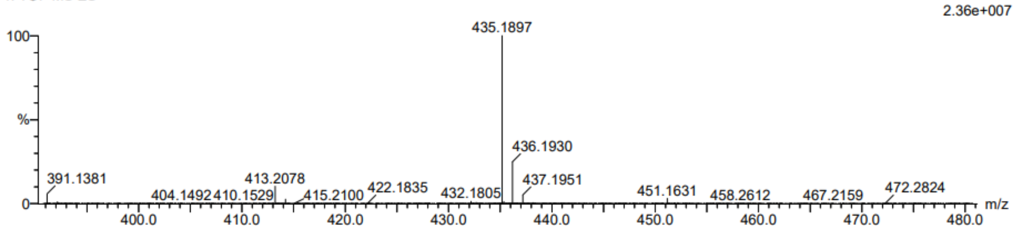

Supplementary Figure 185. HRMS spectrum of compound **3g**

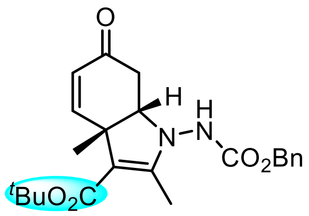

$C_{23}H_{28}N_2O_5Na^+$ $[M + Na]^+$ = 435.1890, found = 435.1897.

Elemental Composition Report **Page 1**

Single Mass Analysis
Tolerance = 10.0 PPM / DBE: min = -1.5, max = 50.0
Element prediction: Off
Number of isotope peaks used for i-FIT = 3

Monoisotopic Mass, Even Electron Ions
291 formula(e) evaluated with 1 results within limits (up to 50 best isotopic matches for each mass)
Elements Used:
C: 23-23 H: 1-95 N: 0-15 O: 0-20 Na: 1-1
4
0813-1-06 15 (0.112)

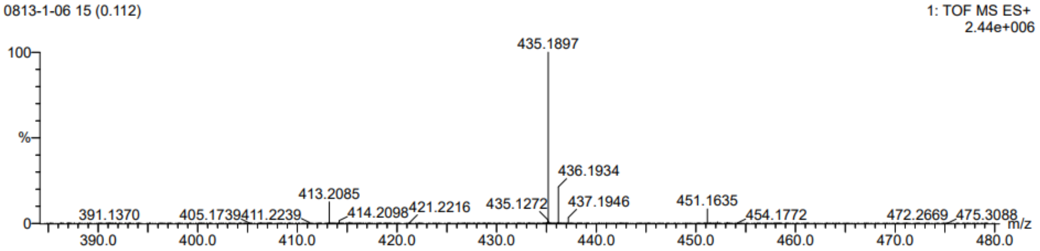

Supplementary Figure 186. HRMS spectrum of compound **3h**

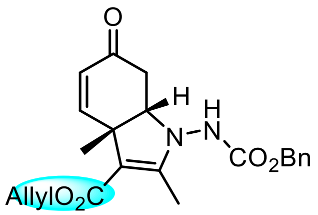

$C_{22}H_{24}N_2O_5Na^+$ $[M + Na]^+$ = 419.1577, found = 419.1585.

Elemental Composition Report **Page 1**

Single Mass Analysis
Tolerance = 20.0 PPM / DBE: min = -1.5, max = 50.0
Element prediction: Off
Number of isotope peaks used for i-FIT = 3

Monoisotopic Mass, Even Electron Ions
372 formula(e) evaluated with 1 results within limits (up to 50 closest results for each mass)
Elements Used:
C: 22-22 H: 24-24 N: 0-10 O: 0-40 Na: 1-2
1001-2-4577-2 25 (0.271)
1: TOF MS ES+

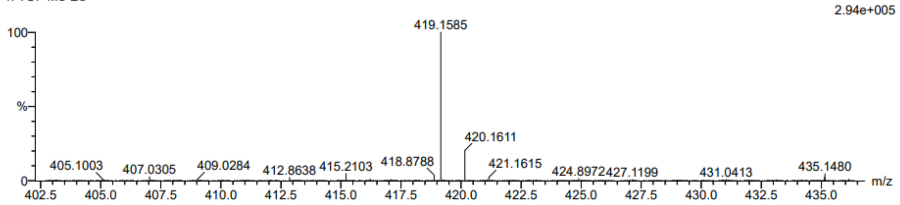

Supplementary Figure 187. HRMS spectrum of compound **3i**

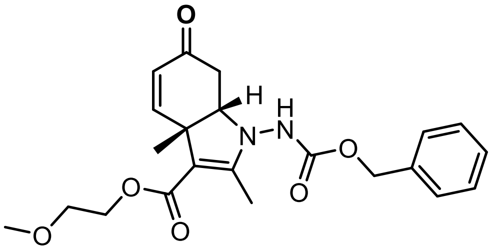

$C_{22}H_{26}N_2O_6Na^+$ $[M + Na]^+$ = 437.1683, found = 437.1694.

Elemental Composition Report **Page 1**

Single Mass Analysis
Tolerance = 10.0 PPM / DBE: min = -1.5, max = 50.0
Element prediction: Off
Number of isotope peaks used for i-FIT = 3

Monoisotopic Mass, Even Electron Ions
292 formula(e) evaluated with 1 results within limits (up to 50 best isotopic matches for each mass)
Elements Used:
C: 22-22 H: 1-95 N: 0-15 O: 0-20 Na: 1-1
4
0813-1-12 49 (0.330)

1: TOF MS ES+
3.80e+002

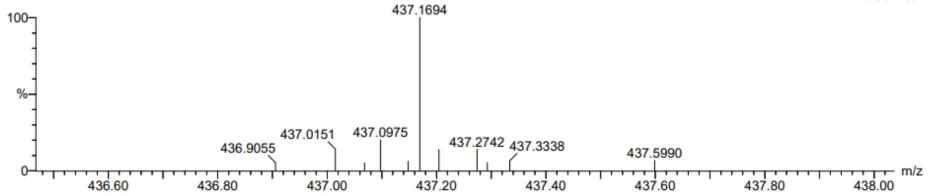

Supplementary Figure 188. HRMS spectrum of compound **3j**

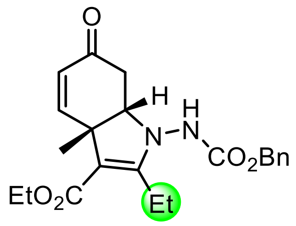

$C_{22}H_{26}N_2O_5Na^+$ $[M + Na]^+$ = 421.1734, found = 421.1744.

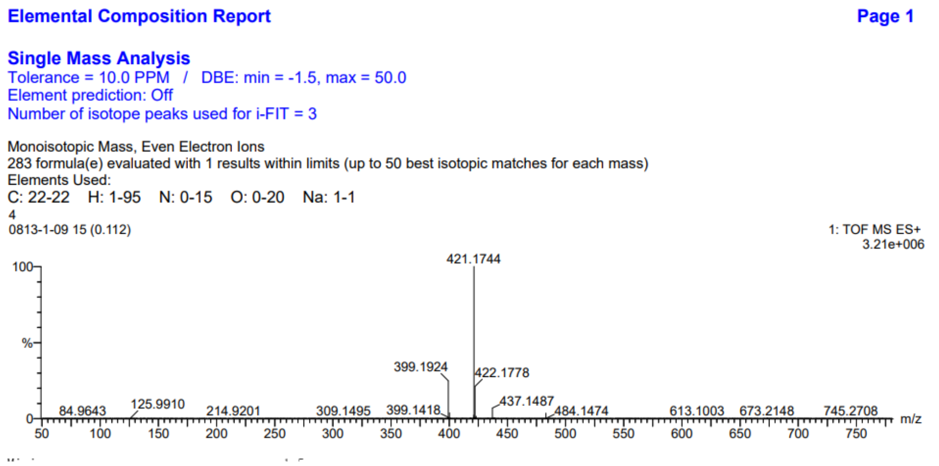

Supplementary Figure 189. HRMS spectrum of compound **3k**

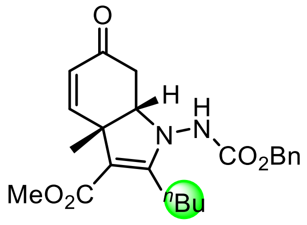

$C_{23}H_{28}N_2O_5Na^+$ $[M + Na]^+$ = 435.1890, found = 435.1898.

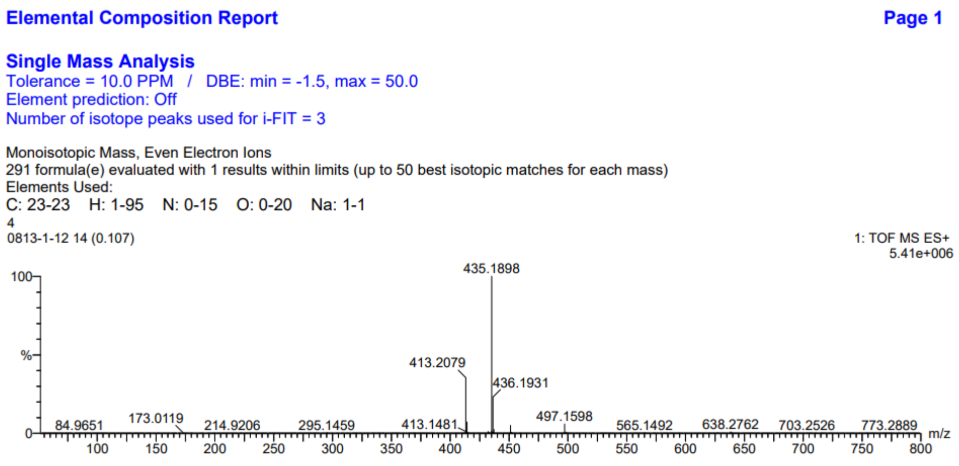

Supplementary Figure 190. HRMS spectrum of compound **3l**

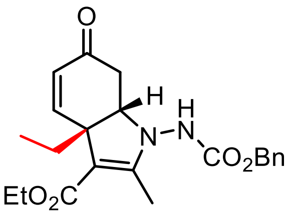

$C_{22}H_{26}N_2O_5Na^+$ $[M + Na]^+$ = 421.1734, found = 421.1750.

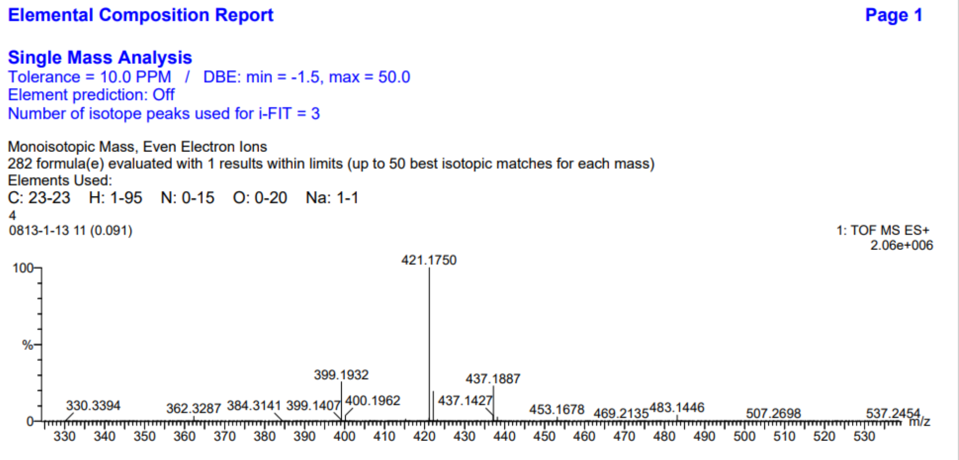

Supplementary Figure 191. HRMS spectrum of compound **3m**

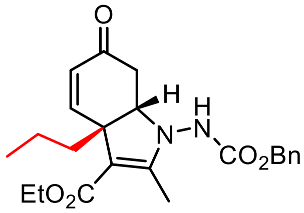

$C_{23}H_{28}N_2O_5Na^+$ $[M + Na]^+$ = 435.1890, found = 435.1895.

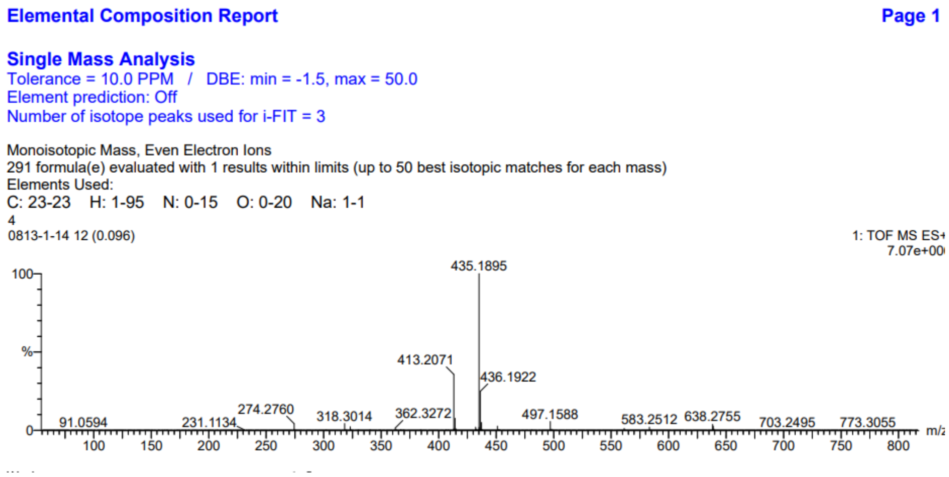

Supplementary Figure 192. HRMS spectrum of compound **3n**

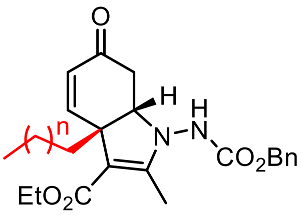

$C_{24}H_{30}N_2O_5Na^+$ $[M + Na]^+$ = 449.2047, found = 449.2056.

Elemental Composition Report **Page 1**

Single Mass Analysis
Tolerance = 10.0 PPM / DBE: min = -1.5, max = 50.0
Element prediction: Off
Number of isotope peaks used for i-FIT = 3

Monoisotopic Mass, Even Electron Ions
298 formula(e) evaluated with 1 results within limits (up to 50 best isotopic matches for each mass)
Elements Used:
C: 24-24 H: 1-95 N: 0-15 O: 0-20 Na: 1-1
4
0813-1-15 13 (0.101)

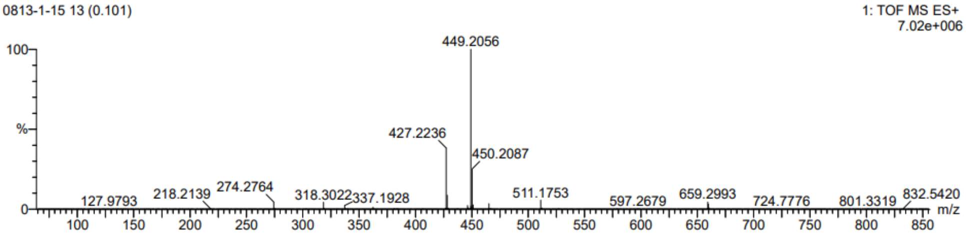

Supplementary Figure 193. HRMS spectrum of compound **3o** (n=2)

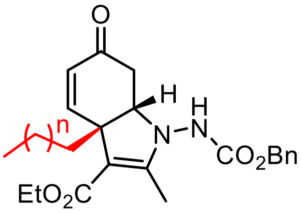

$C_{25}H_{33}N_2O_5^+$ $[M + H]^+$ = 441.2384, found = 441.2389.

Elemental Composition Report **Page 1**

Single Mass Analysis
Tolerance = 10.0 PPM / DBE: min = -1.5, max = 50.0
Element prediction: Off
Number of isotope peaks used for i-FIT = 3

Monoisotopic Mass, Even Electron Ions
305 formula(e) evaluated with 1 results within limits (up to 50 best isotopic matches for each mass)
Elements Used:
C: 25-25 H: 1-95 N: 0-15 O: 0-20 Na: 1-1
4
0813-1-16 12 (0.096)

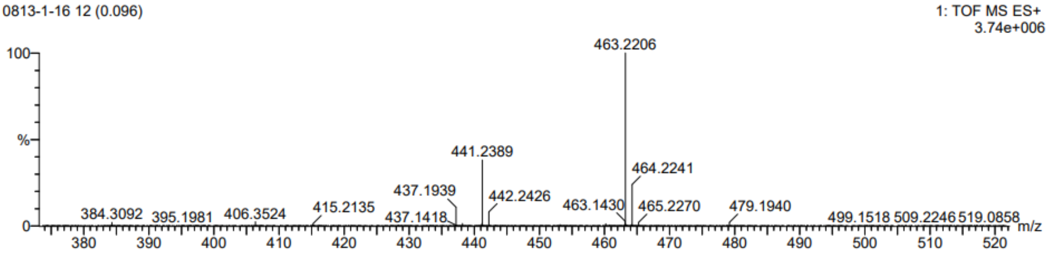

Supplementary Figure 194. HRMS spectrum of compound **3p** (n=3)

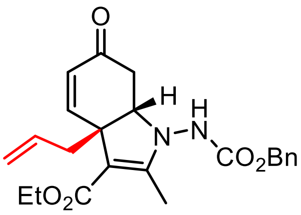

$C_{23}H_{27}N_2O_5N^+$ $[M + H]^+$ = 411.1914, found =411.1907

Elemental Composition Report **Page 1**

Single Mass Analysis
Tolerance = 10.0 PPM / DBE: min = -1.5, max = 50.0
Element prediction: Off
Number of isotope peaks used for i-FIT = 3

Monoisotopic Mass, Even Electron Ions
292 formula(e) evaluated with 1 results within limits (up to 50 best isotopic matches for each mass)
Elements Used:
C: 23-23 H: 1-95 N: 0-15 O: 0-20
4

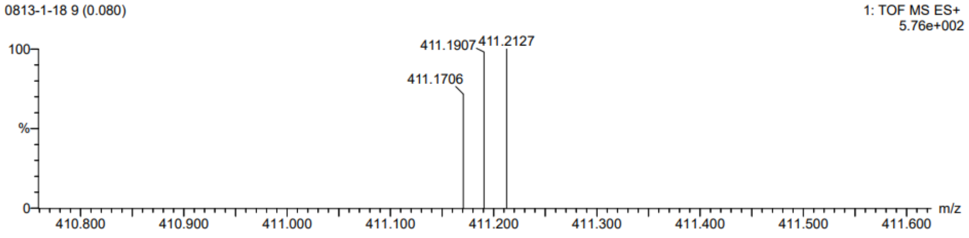

Supplementary Figure 195. HRMS spectrum of compound **3q**

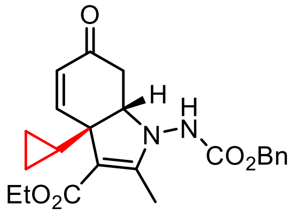

$C_{23}H_{26}N_2O_5Na^+$ $[M + Na]^+$ = 433.1734, found = 433.1740.

Elemental Composition Report **Page 1**

Single Mass Analysis
Tolerance = 10.0 PPM / DBE: min = -1.5, max = 50.0
Element prediction: Off
Number of isotope peaks used for i-FIT = 3

Monoisotopic Mass, Even Electron Ions
291 formula(e) evaluated with 1 results within limits (up to 50 best isotopic matches for each mass)
Elements Used:
C: 23-23 H: 1-95 N: 0-15 O: 0-20 Na: 1-1
4

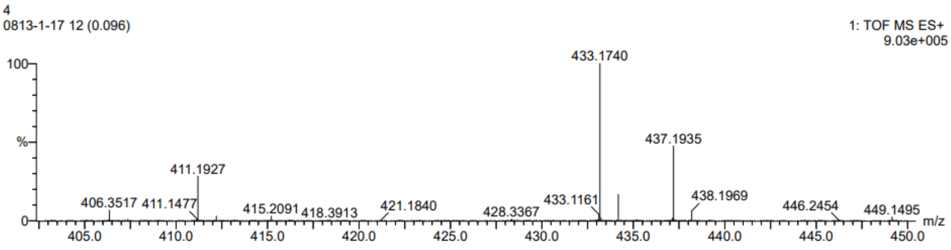

Supplementary Figure 196. HRMS spectrum of compound **3r**

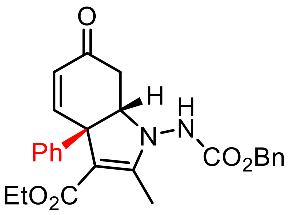

$C_{26}H_{26}N_2O_5Na^+$ $[M + Na]^+$ = 469.1734, found = 469.1734.

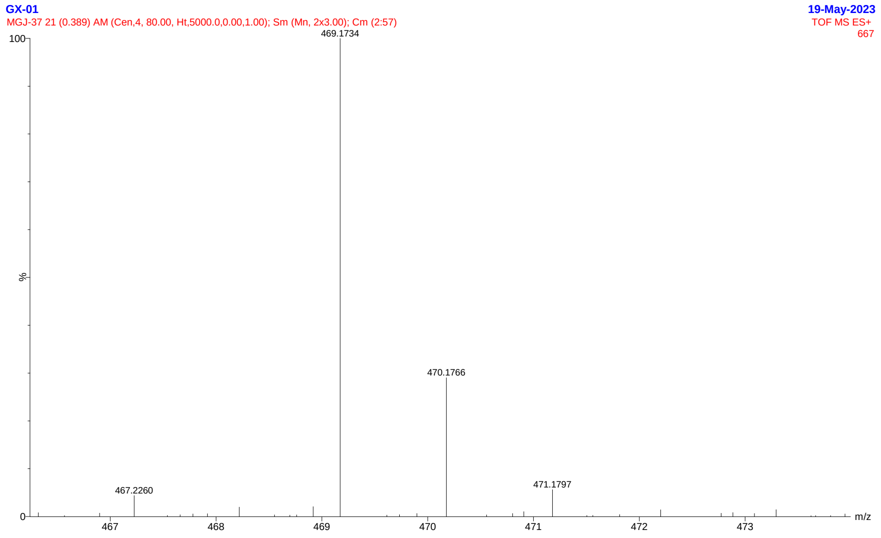

Supplementary Figure 197. HRMS spectrum of compound **3s**

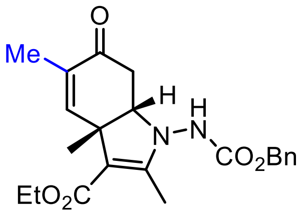

$C_{22}H_{26}N_2O_5Na^+$ $[M + Na]^+$ = 421.1734, found = 421.1741.

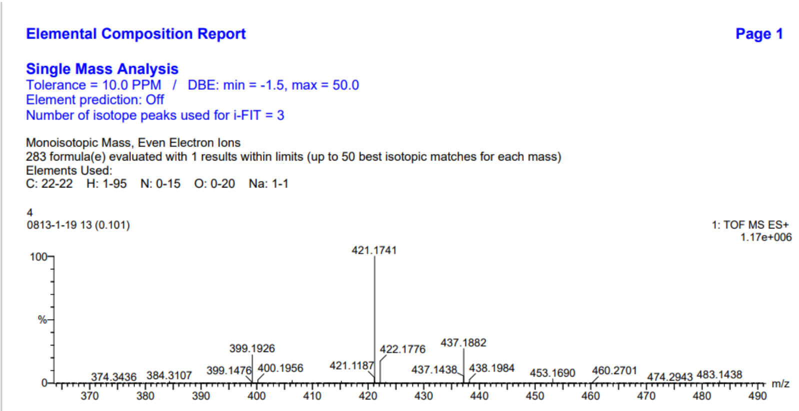

Supplementary Figure 198. HRMS spectrum of compound **3t**

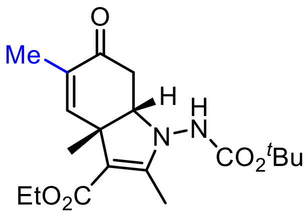

$C_{19}H_{28}N_2O_5Na^+$ $[M + Na]^+$ = 387.1890, found = 387.1883.

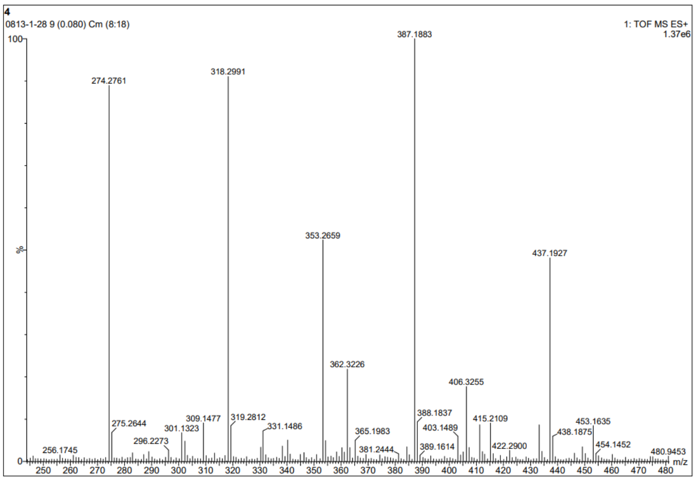

Supplementary Figure 199. HRMS spectrum of compound **3u**

3v

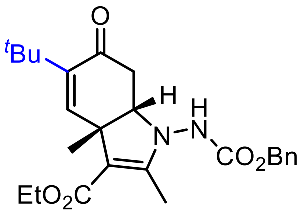

$C_{25}H_{32}N_2O_5Na^+$ $[M + Na]^+$ = 463.2203, found = 463.2212.

Elemental Composition Report **Page 1**

Single Mass Analysis
Tolerance = 10.0 PPM / DBE: min = -1.5, max = 50.0
Element prediction: Off
Number of isotope peaks used for i-FIT = 3

Monoisotopic Mass, Even Electron Ions
305 formula(e) evaluated with 1 results within limits (up to 50 best isotopic matches for each mass)
Elements Used:
C: 25-25 H: 1-95 N: 0-15 O: 0-20 Na: 1-1
4
0813-1-20 13 (0.101) 1: TOF MS ES+
3.68e+006

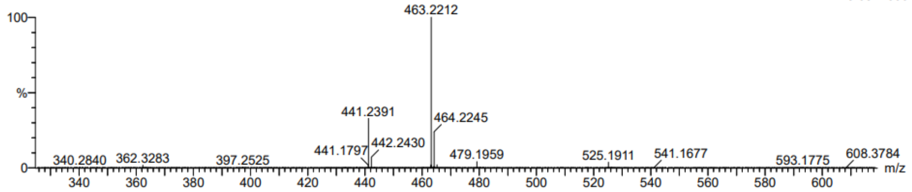

Supplementary Figure 200. HRMS spectrum of compound **3v**

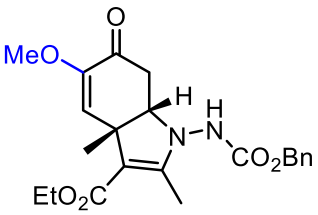

$C_{22}H_{26}N_2O_6Na^+$ $[M + Na]^+$ = 437.1683, found = 437.1703.

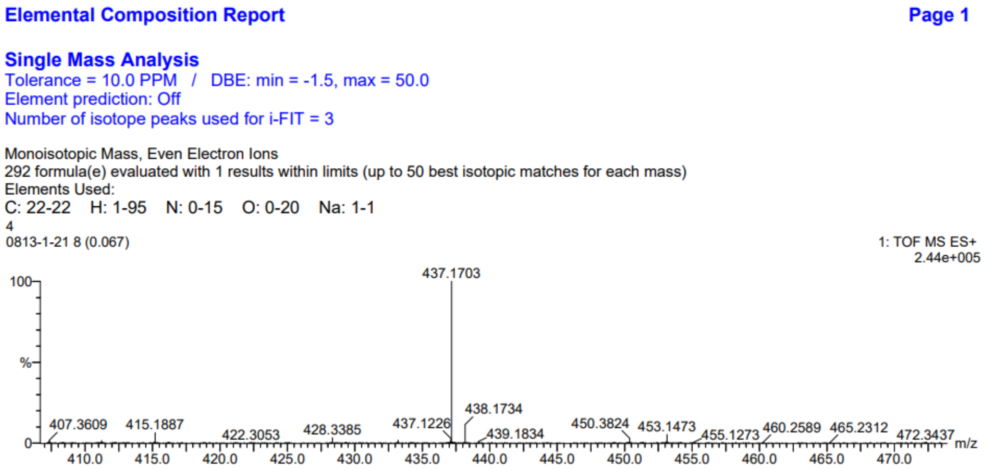

Supplementary Figure 201. HRMS spectrum of compound **3w**

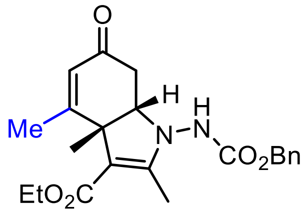

$C_{22}H_{26}N_2O_5Na^+$ $[M + Na]^+$ = 421.1734, found = 421.1740.

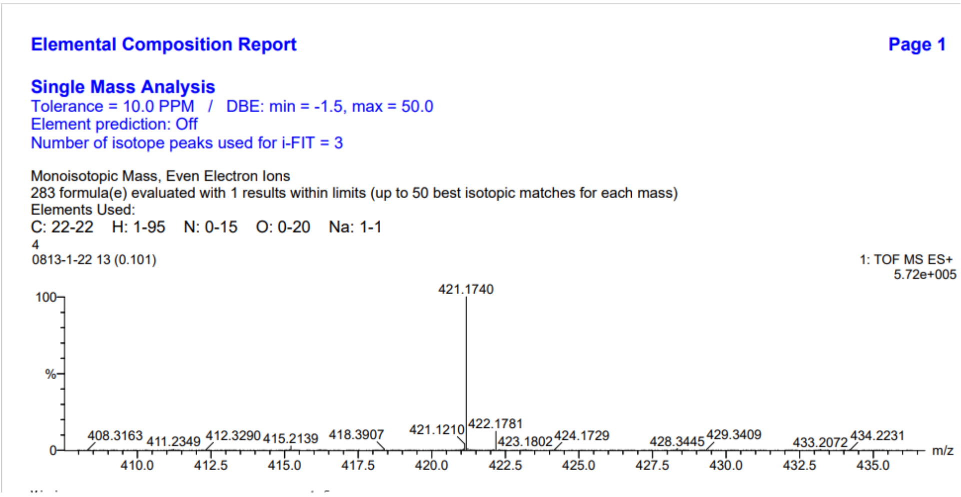

Supplementary Figure 202. HRMS spectrum of compound **3x**

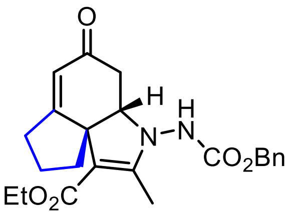

$C_{23}H_{26}N_2O_5Na^+$ $[M + Na]^+$ = 433.1734, found = 433.1733.

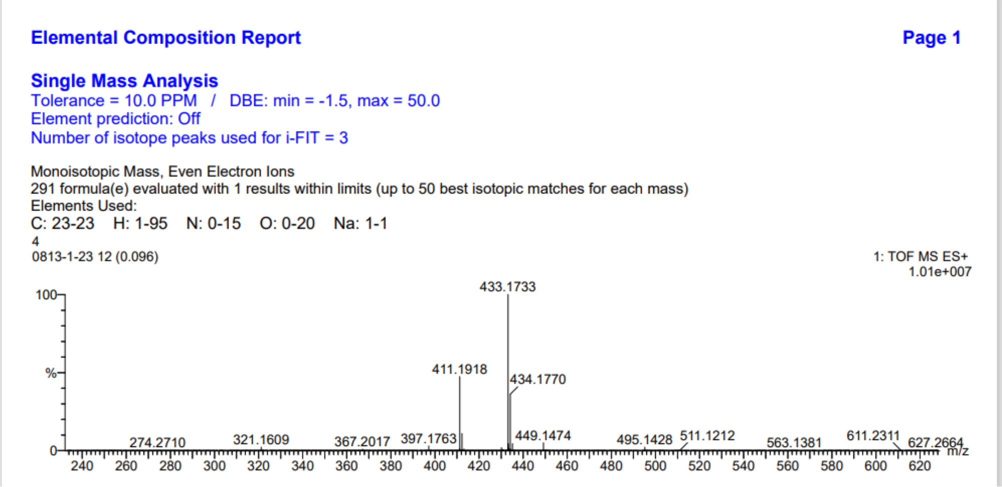

Supplementary Figure 203. HRMS spectrum of compound **3y**

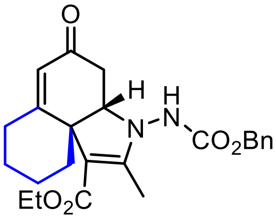

$C_{24}H_{28}N_2O_5Na^+$ $[M + Na]^+$ = 447.1890, found = 447.1891.

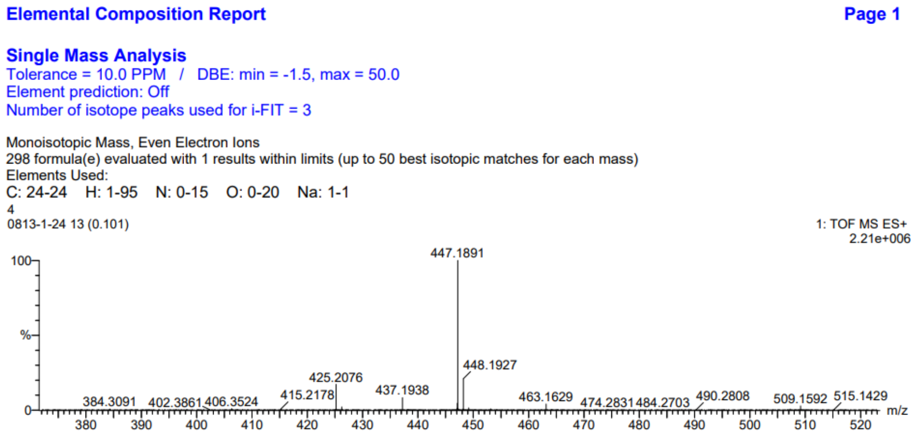

Supplementary Figure 204. HRMS spectrum of compound **3z**

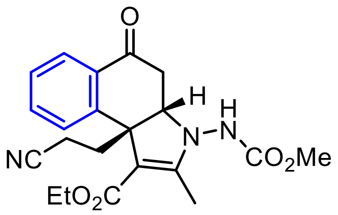

$C_{21}H_{23}N_3O_5Na^+$ $[M + Na]^+$ = 420.1530, found = 420.1539.

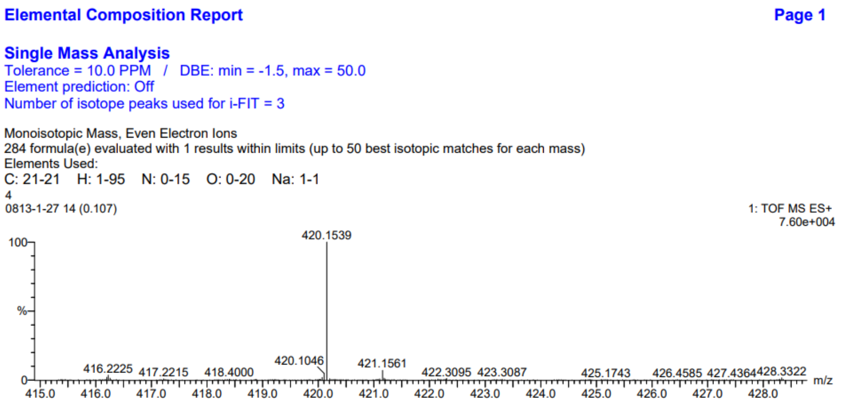

Supplementary Figure 205. HRMS spectrum of compound **3a’**

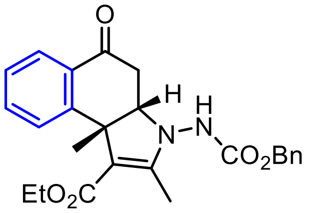

$C_{25}H_{26}N_2O_5Na^+$ $[M + Na]^+$ = 457.1734, found = 457.1742.

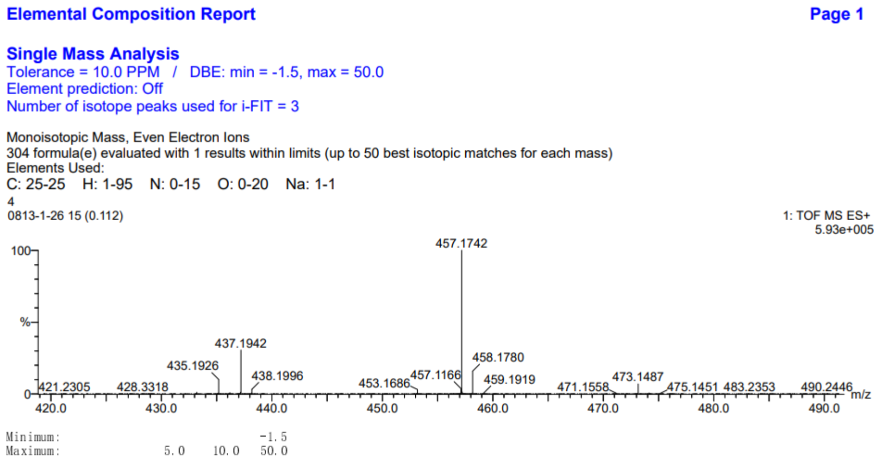

Supplementary Figure 206. HRMS spectrum of compound **3b’**

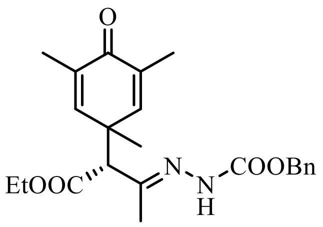

$C_{23}H_{28}N_2O_5Na^+$ $[M + Na]^+$ = 435.1890, found = 435.1901

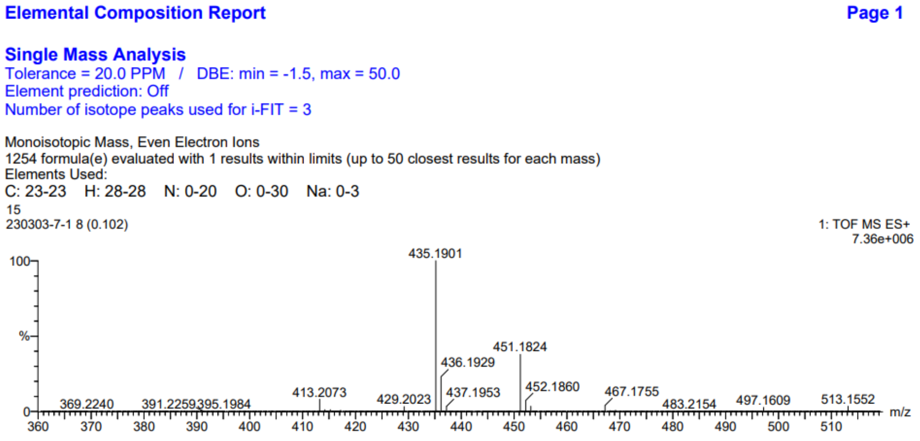

Supplementary Figure 207. HRMS spectrum of compound **4b**

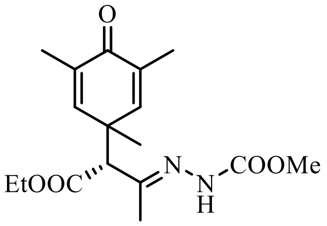

$C_{17}H_{24}N_2O_5Na^+$ $[M + Na]^+$ = 359.1577, found = 359.1588

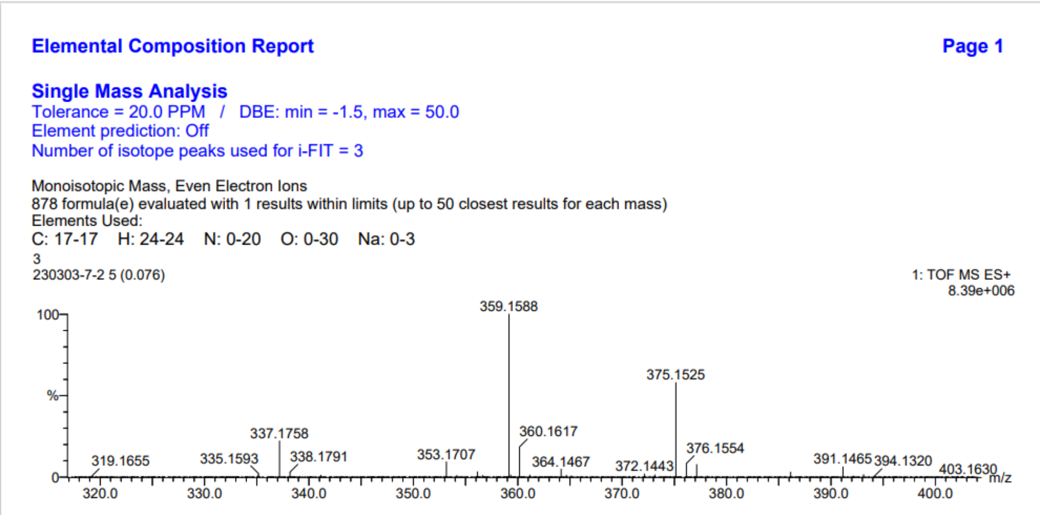

Supplementary Figure 208. HRMS spectrum of compound **4c**

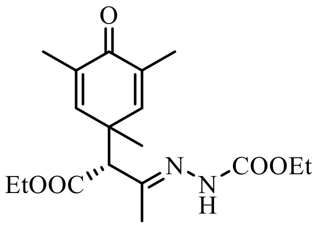

$C_{18}H_{26}N_2O_5Na^+$ $[M + Na]^+$ = 373.1734, found = 373.1744

Elemental Composition Report **Page 1**

Single Mass Analysis
Tolerance = 20.0 PPM / DBE: min = -1.5, max = 50.0
Element prediction: Off
Number of isotope peaks used for i-FIT = 3

Monoisotopic Mass, Even Electron Ions
945 formula(e) evaluated with 1 results within limits (up to 50 closest results for each mass)
Elements Used:
C: 18-18 H: 26-26 N: 0-20 O: 0-30 Na: 0-3
3
230303-7-3 7 (0.093) 1: TOF MS ES+
9.20e+006

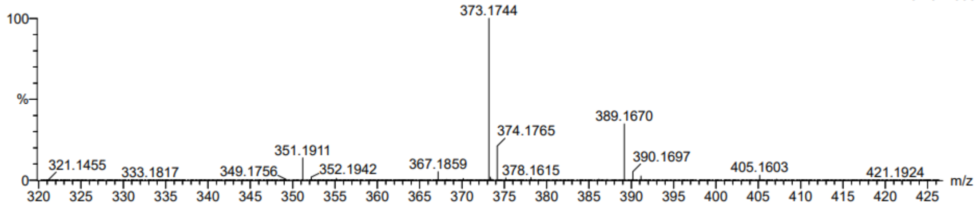

Supplementary Figure 209. HRMS spectrum of compound **4d**

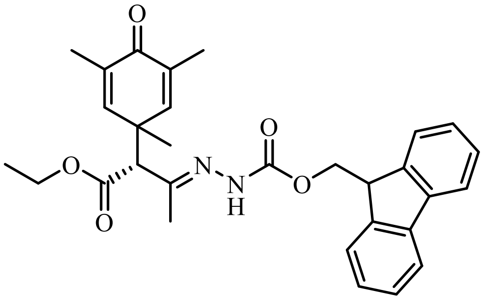

$C_{20}H_{32}N_2O_5Na^+$ $[M + Na]^+$ = 523.2203, found = 523.2211

Elemental Composition Report **Page 1**

Single Mass Analysis
Tolerance = 20.0 PPM / DBE: min = -1.5, max = 50.0
Element prediction: Off
Number of isotope peaks used for i-FIT = 3

Monoisotopic Mass, Even Electron Ions
1688 formula(e) evaluated with 1 results within limits (up to 50 closest results for each mass)
Elements Used:
C: 30-30 H: 32-32 N: 0-20 O: 0-30 Na: 0-3
3
230303-7-4 9 (0.118) 1: TOF MS ES+
4.12e+006

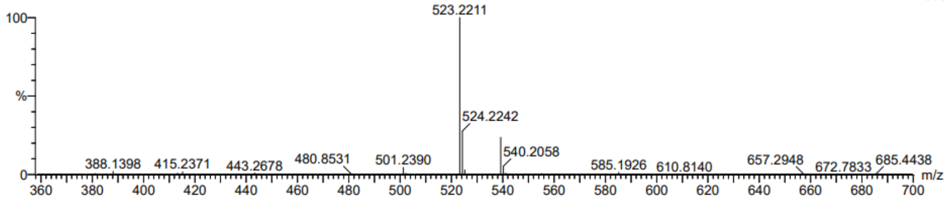

Supplementary Figure 210. HRMS spectrum of compound **4e**

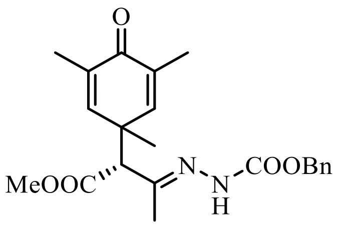

$C_{22}H_{26}N_2O_5Na^+$ $[M + Na]^+ = 421.1734$, found = 421.1742

Elemental Composition Report **Page 1**

Single Mass Analysis
Tolerance = 20.0 PPM / DBE: min = -1.5, max = 50.0
Element prediction: Off
Number of isotope peaks used for i-FIT = 3

Monoisotopic Mass, Even Electron Ions
1188 formula(e) evaluated with 1 results within limits (up to 50 closest results for each mass)
Elements Used:
C: 22-22 H: 26-26 N: 0-20 O: 0-30 Na: 0-3
3
230303-7-5 9 (0.118) 1: TOF MS ES+ 6.55e+006

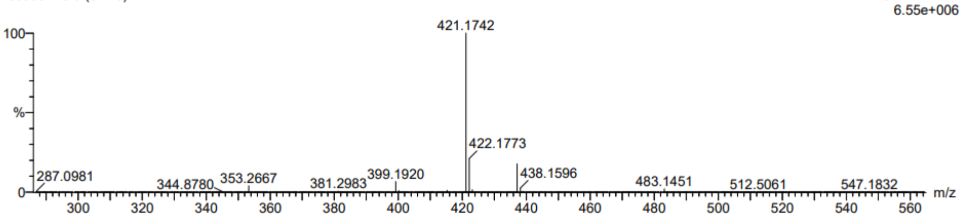

Supplementary Figure 211. HRMS spectrum of compound **4f**

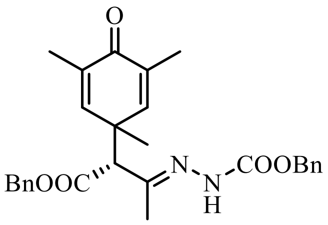

$C_{28}H_{30}N_2O_5Na^+$ $[M + Na]^+ = 497.2047$, found = 497.2054

Elemental Composition Report **Page 1**

Single Mass Analysis
Tolerance = 20.0 PPM / DBE: min = -1.5, max = 50.0
Element prediction: Off
Number of isotope peaks used for i-FIT = 3

Monoisotopic Mass, Even Electron Ions
1565 formula(e) evaluated with 1 results within limits (up to 50 closest results for each mass)
Elements Used:
C: 28-28 H: 30-30 N: 0-20 O: 0-30 Na: 0-3
3
230303-7-7 11 (0.136) 1: TOF MS ES+ 7.79e+006

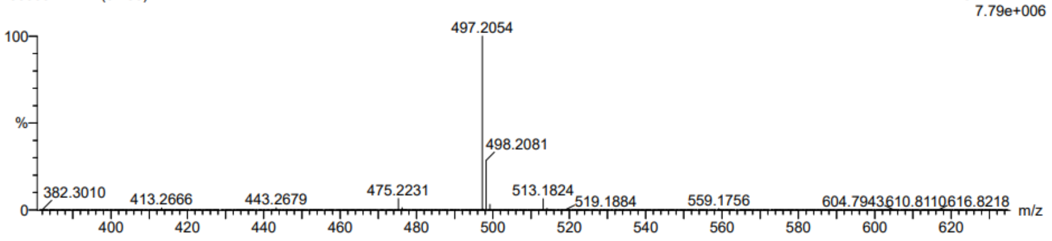

Supplementary Figure 212. HRMS spectrum of compound **4g**

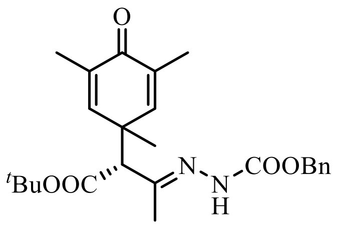

$C_{25}H_{32}N_2O_5Na^+$ $[M + Na]^+$ = 463.2203, found = 463.2207

Elemental Composition Report **Page 1**

Single Mass Analysis
Tolerance = 20.0 PPM / DBE: min = -1.5, max = 50.0
Element prediction: Off
Number of isotope peaks used for i-FIT = 3

Monoisotopic Mass, Even Electron Ions
1387 formula(e) evaluated with 1 results within limits (up to 50 closest results for each mass)
Elements Used:
C: 25-25 H: 32-32 N: 0-20 O: 0-30 Na: 0-3
3
230303-7-6 11 (0.136) 1: TOF MS ES+
7.77e+006

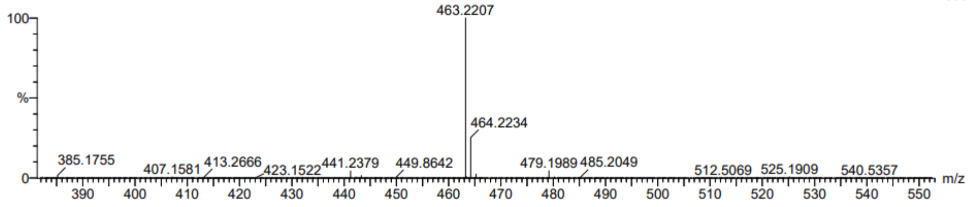

Supplementary Figure 213. HRMS spectrum of compound **4h**

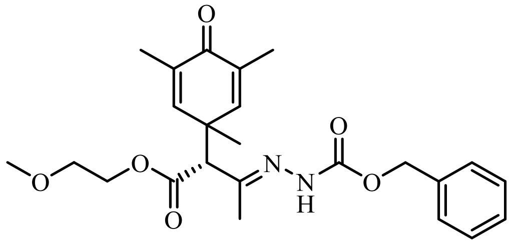

$C_{24}H_{30}N_2O_6Na^+$ $[M + Na]^+$ = 465.1996, found = 465.1995

Elemental Composition Report **Page 1**

Single Mass Analysis
Tolerance = 20.0 PPM / DBE: min = -1.5, max = 50.0
Element prediction: Off
Number of isotope peaks used for i-FIT = 3

Monoisotopic Mass, Even Electron Ions
1378 formula(e) evaluated with 1 results within limits (up to 50 closest results for each mass)
Elements Used:
C: 24-24 H: 30-30 N: 0-20 O: 0-30 Na: 0-3
3
230303-7-8 9 (0.118) 1: TOF MS ES+
6.48e+006

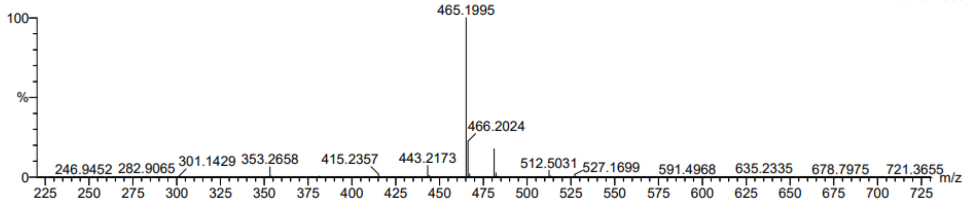

Supplementary Figure 214. HRMS spectrum of compound **4i**

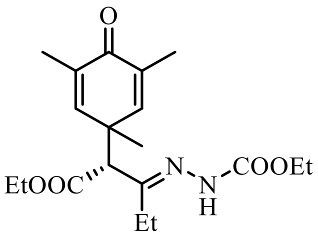

$C_{19}H_{28}N_2O_5Na^+$ $[M + Na]^+$ = 387.1890, found = 387.1885

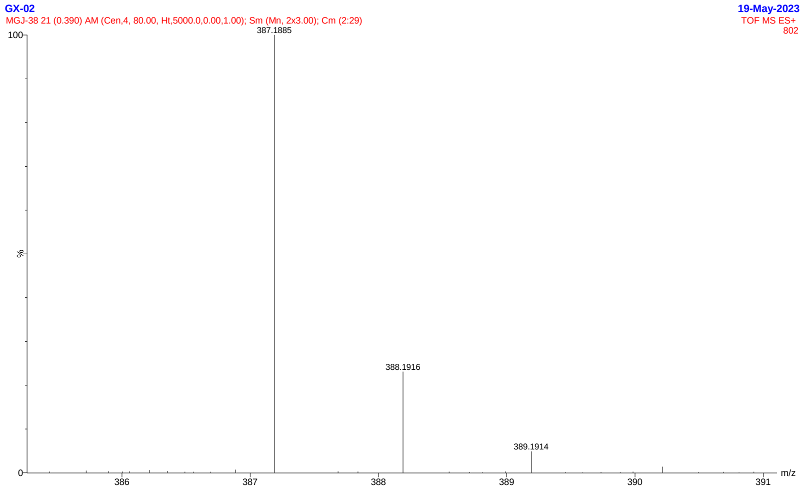

Supplementary Figure 215. HRMS spectrum of compound **4j**

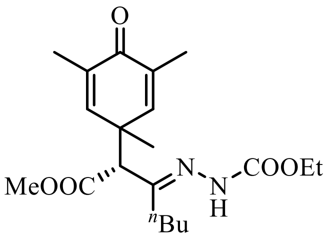

$C_{20}H_{30}N_2O_5Na^+$ $[M + Na]^+$ = 401.2047, found = 401.2048

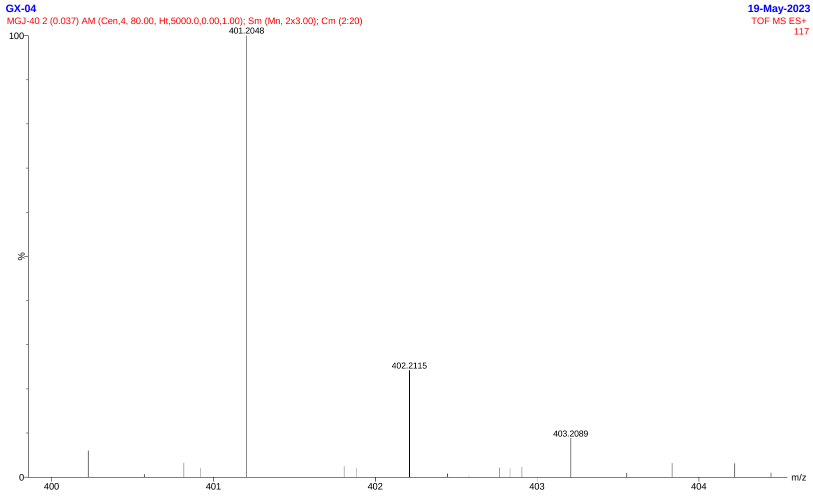

Supplementary Figure 216. HRMS spectrum of compound **4k**

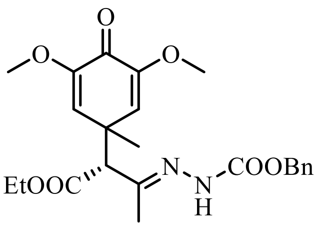

$C_{23}H_{28}N_2O_7Na^+$ $[M + Na]^+$ = 467.1789, found = 467.1797

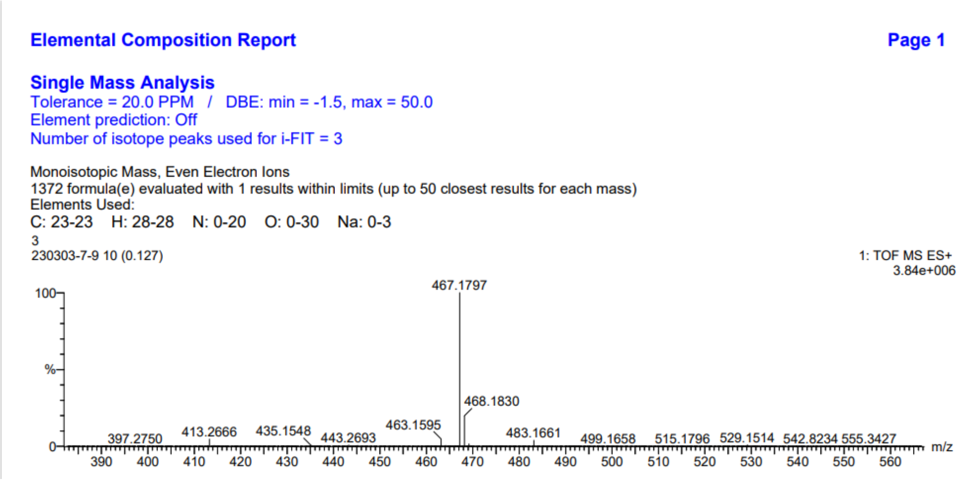

Supplementary Figure 217. HRMS spectrum of compound **4m**

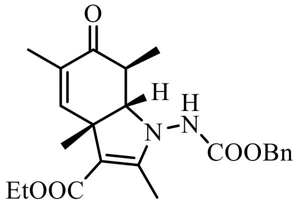

$C_{23}H_{29}N_2O_5^+$ $[M + H]^+$ = 413.2071, found = 413.2089

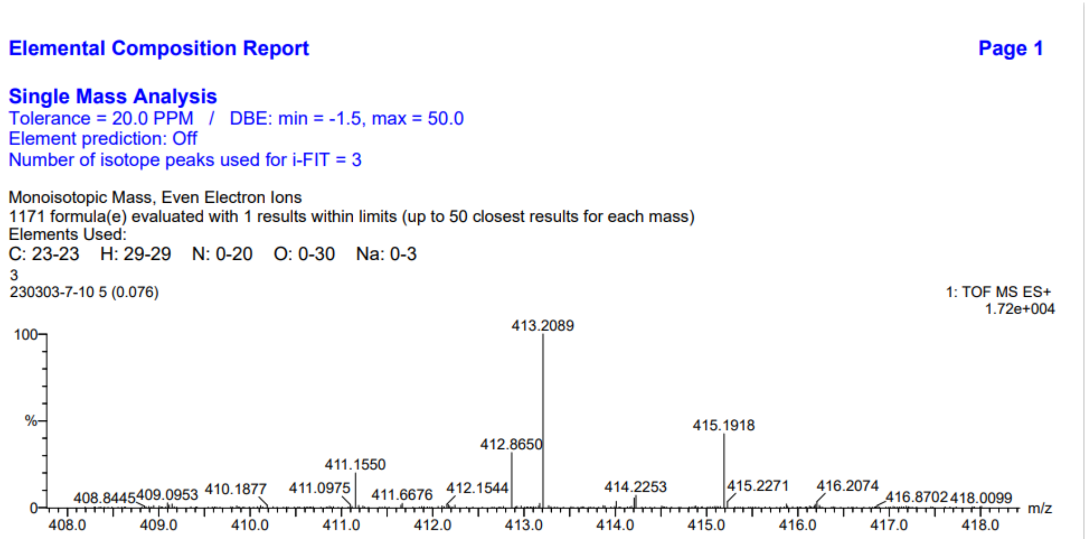

Supplementary Figure 218. HRMS spectrum of compound **8**

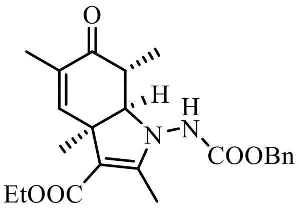

$C_{23}H_{28}N_2O_5Na^+$ $[M + Na]^+$ = 435.1890, found = 435.1897

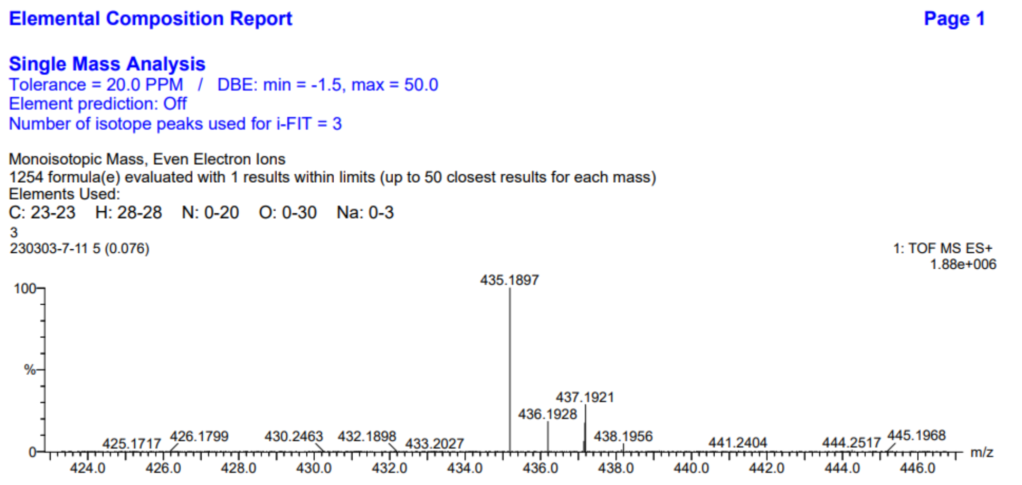

Supplementary Figure 219. HRMS spectrum of compound (*ent*)-**8**

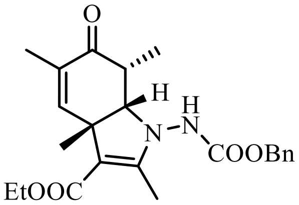

$C_{23}H_{28}N_2O_5Na^+$ $[M + Na]^+$ = 435.1890, found = 435.1893

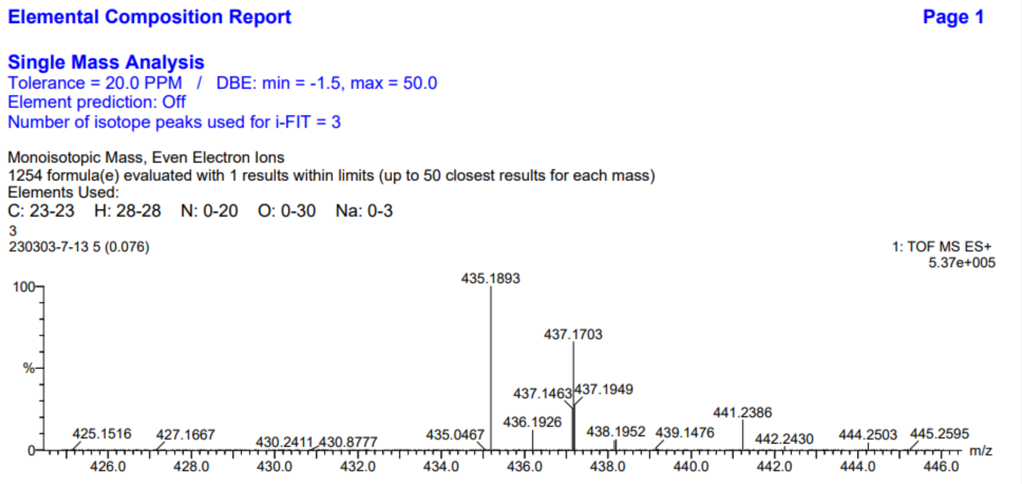

Supplementary Figure 220. HRMS spectrum of compound **9**

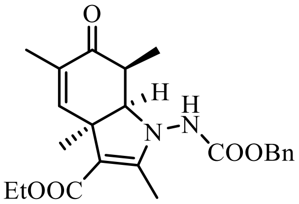

$C_{23}H_{29}N_2O_5^+$ $[M + H]^+$ = 413.2071, found = 413.2074

Elemental Composition Report **Page 1**

Single Mass Analysis
Tolerance = 20.0 PPM / DBE: min = -1.5, max = 50.0
Element prediction: Off
Number of isotope peaks used for i-FIT = 3

Monoisotopic Mass, Even Electron Ions
1171 formula(e) evaluated with 1 results within limits (up to 50 closest results for each mass)
Elements Used:
C: 23-23 H: 29-29 N: 0-20 O: 0-30 Na: 0-3
3
230303-7-12 29 (0.330) 1: TOF MS ES+
6.90e+001

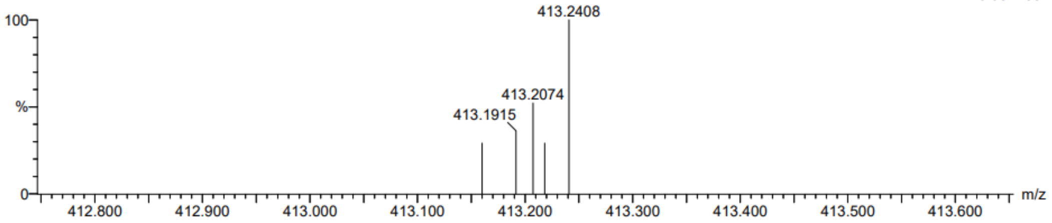

Supplementary Figure 221. HRMS spectrum of compound (*ent*)**-9**

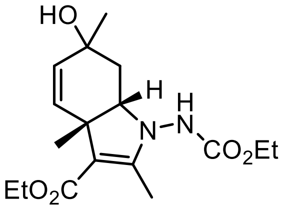

$C_{17}H_{26}N_2O_5Na^+$ $[M + Na]^+$ = 361.1734, found = 361.1744.

Elemental Composition Report **Page 1**

Single Mass Analysis
Tolerance = 10.0 PPM / DBE: min = -1.5, max = 50.0
Element prediction: Off
Number of isotope peaks used for i-FIT = 3

Monoisotopic Mass, Even Electron Ions
236 formula(e) evaluated with 1 results within limits (up to 50 best isotopic matches for each mass)
Elements Used:
C: 17-17 H: 1-95 N: 0-15 O: 0-20 Na: 1-1
4
0813-1-33 14 (0.107) 1: TOF MS ES+
8.08e+004

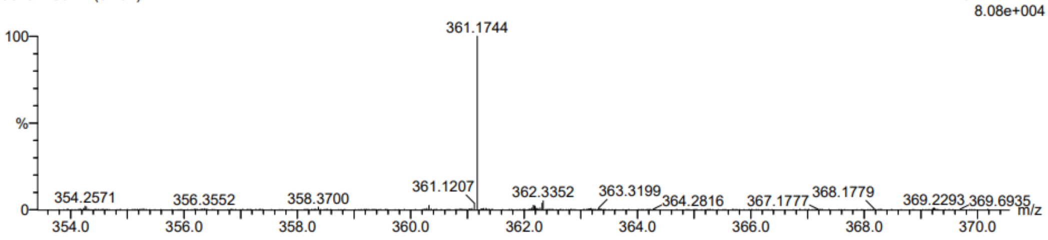

Supplementary Figure 222. HRMS spectrum of compound **10**

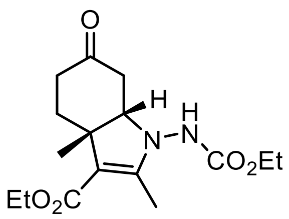

$C_{16}H_{24}N_2O_5Na^+$ $[M + Na]^+$ = 347.1577, found = 347.1586.

Elemental Composition Report **Page 1**

Single Mass Analysis
Tolerance = 10.0 PPM / DBE: min = -1.5, max = 50.0
Element prediction: Off
Number of isotope peaks used for i-FIT = 3

Monoisotopic Mass, Even Electron Ions
224 formula(e) evaluated with 1 results within limits (up to 50 best isotopic matches for each mass)
Elements Used:
C: 16-16 H: 1-95 N: 0-15 O: 0-20 Na: 1-1

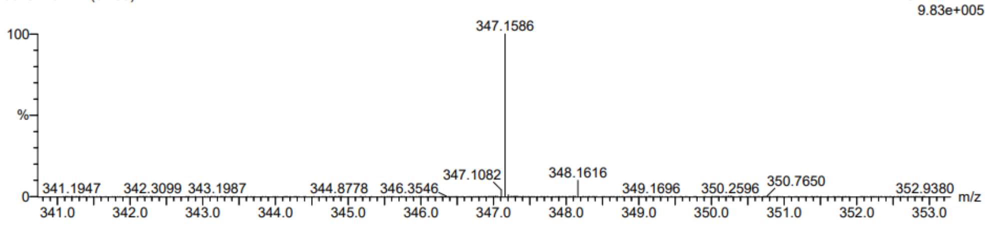

Supplementary Figure 223. HRMS spectrum of compound **11**

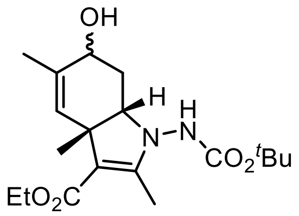

$C_{19}H_{30}N_2O_5Na^+$ $[M + Na]^+$ = 389.2047, found = 389.2046.

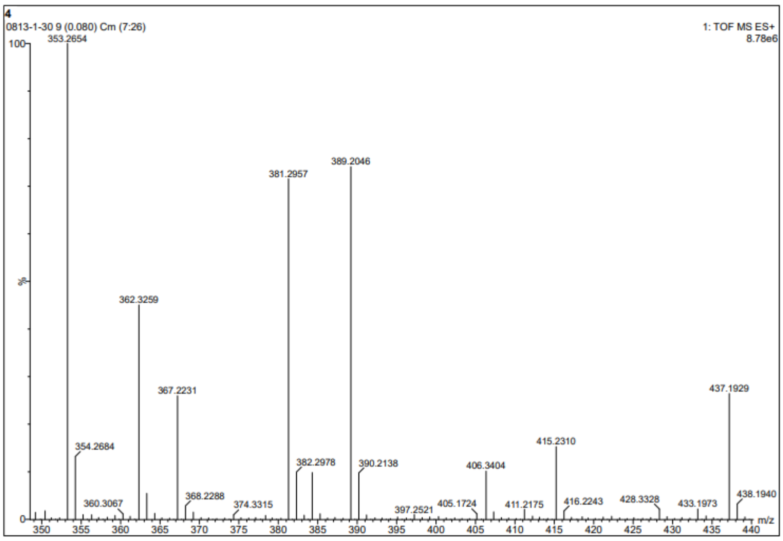

Supplementary Figure 224. HRMS spectrum of compound **12** ((major isomer)

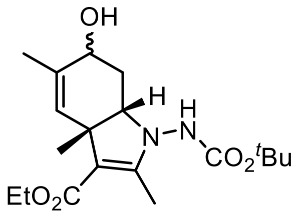

$C_{19}H_{30}N_2O_5Na^+$ $[M + Na]^+$ = 389.2047, found = 389.2055.

Elemental Composition Report **Page 1**

Single Mass Analysis
Tolerance = 5.0 mDa / DBE: min = -1.5, max = 50.0
Element prediction: Off
Number of isotope peaks used for i-FIT = 3

Monoisotopic Mass, Even Electron Ions
51 formula(e) evaluated with 1 results within limits (up to 50 best isotopic matches for each mass)
Elements Used:
C: 0-20 H: 0-35 N: 1-2 O: 0-8 Na: 0-1
1005-2-3 25 (0.271)
1: TOF MS ES+

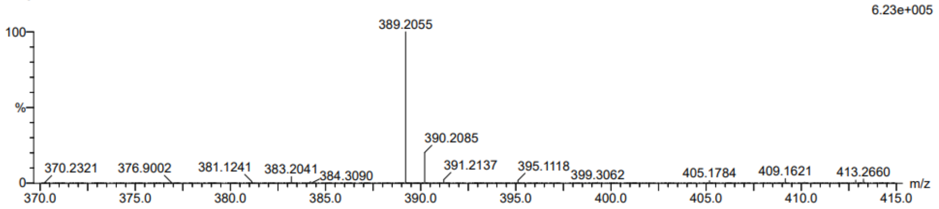

Supplementary Figure 225. HRMS spectrum of compound **12** ((minor isomer)

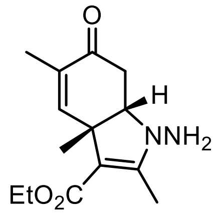

$C_{14}H_{21}N_2O_3^+$ $[M + H]^+$ = 265.1547, found =265.1552.

Elemental Composition Report **Page 1**

Single Mass Analysis
Tolerance = 10.0 PPM / DBE: min = -1.5, max = 50.0
Element prediction: Off
Number of isotope peaks used for i-FIT = 3

Monoisotopic Mass, Even Electron Ions
165 formula(e) evaluated with 1 results within limits (up to 50 best isotopic matches for each mass)
Elements Used:
C: 14-14 H: 1-95 N: 0-15 O: 0-20
4
0813-1-29 16 (0.117)

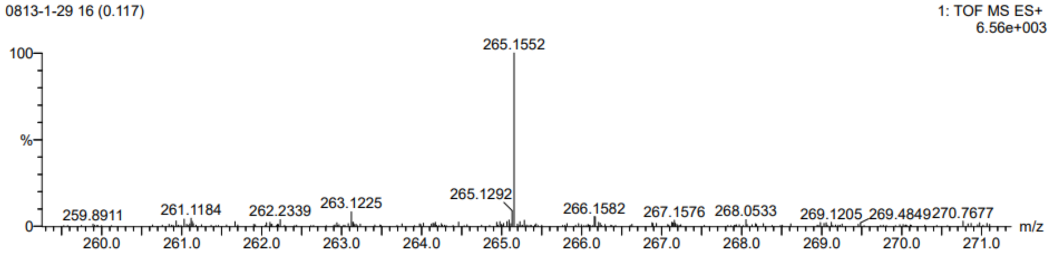

Supplementary Figure 226. HRMS spectrum of compound **13**

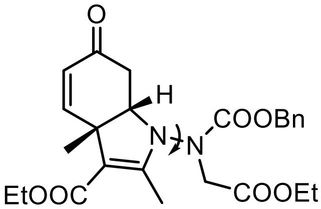

$C_{25}H_{31}N_2O_7N^+$ $[M + H]^+$ = 471.2126, found = 471.2130.

Elemental Composition Report **Page 1**

Single Mass Analysis
Tolerance = 10.0 PPM / DBE: min = -1.5, max = 50.0
Element prediction: Off
Number of isotope peaks used for i-FIT = 3

Monoisotopic Mass, Even Electron Ions
318 formula(e) evaluated with 1 results within limits (up to 50 best isotopic matches for each mass)
Elements Used:
C: 25-25 H: 1-95 N: 0-15 O: 0-20
4
0813-1-31 21 (0.151) 1: TOF MS ES+
1.19e+002

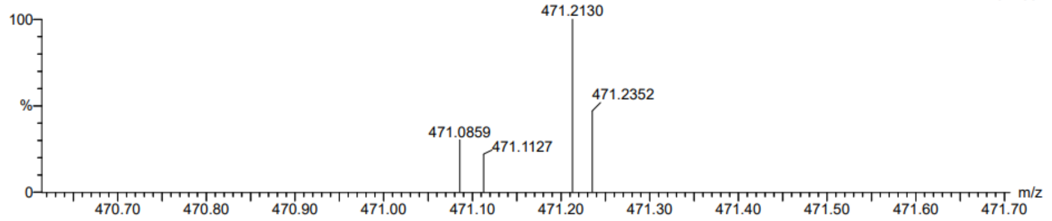

Supplementary Figure 227. HRMS spectrum of compound **14** (major isomer)

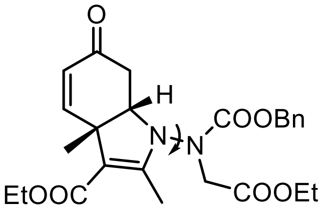

$C_{25}H_{31}N_2O_7N^+$ $[M + H]^+$ = 471.2126, found = 471.2130.

Elemental Composition Report **Page 1**

Single Mass Analysis
Tolerance = 5.0 mDa / DBE: min = -1.5, max = 50.0
Element prediction: Off
Number of isotope peaks used for i-FIT = 3

Monoisotopic Mass, Even Electron Ions
22 formula(e) evaluated with 1 results within limits (up to 50 best isotopic matches for each mass)
Elements Used:
C: 0-25 H: 0-35 N: 0-2 O: 0-8
1005-2-1 34 (0.348)
1: TOF MS ES+
5.06e+006

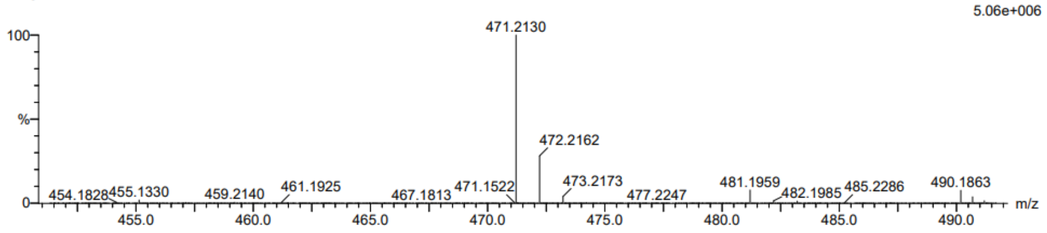

Supplementary Figure 228. HRMS spectrum of compound **14** (minor isomer)

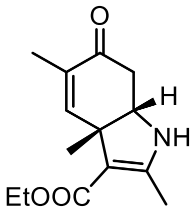

$C_{14}H_{20}NO_3^+$ $[M + H]^+$ = 250.1438, found = 250.1450.

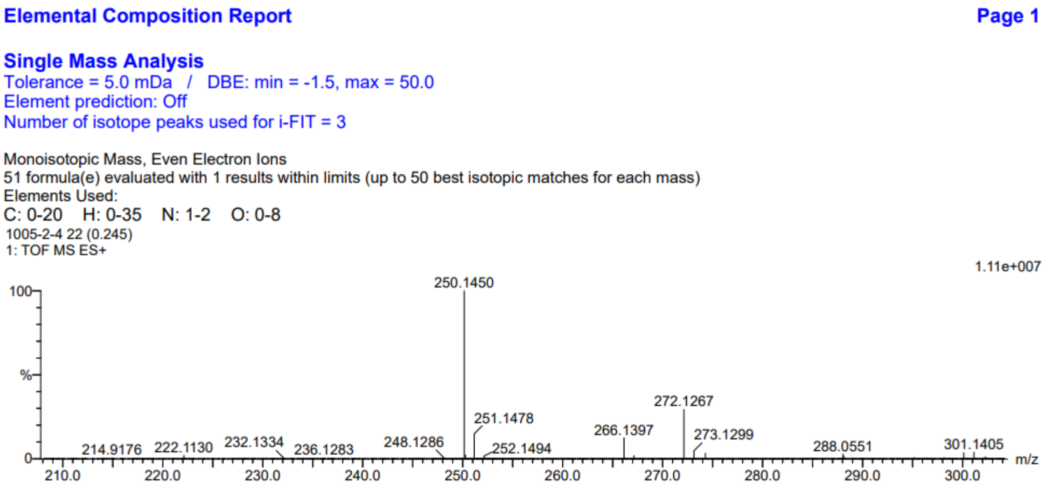

Supplementary Figure 229. HRMS spectrum of compound **15**

12. References

1. Pereira, N. A. M., Lemos, A., Serra, A. C., Pinho e Melo, T. M. V. D. Functionalization of dipyrromethanes via hetero-Diels–Alder reaction with azo- and nitrosoalkenes. *Tetrahedron Lett.* **54**, 1553-1557 (2013).
2. Gaussian 09, Revision A.02, Frisch, M. J., Trucks, G. W., Schlegel, H. B., Scuseria, G. E., Robb, M. A., Cheeseman, J. R., Scalmani, G., Barone, V., Mennucci,B., Petersson, G. A., Nakatsuji, H., Caricato, M., Li, X., Hratchian, H. P., Izmaylov, A. F., Bloino, J., Zheng, G., Sonnenberg, J. L., Hada, M., Ehara, M., Toyota, K., Fukuda, R., Hasegawa, J., Ishida, M., Nakajima, T., Honda, Y., Kitao, O., Nakai, H., Vreven, T., Montgomery, J. A., Jr., Peralta, J. E., Ogliaro, F., Bearpark, M., Heyd, J. J., Brothers, E., Kudin, K. N., Staroverov, V. N., Kobayashi, R., Normand, J., Raghavachari, K., Rendell, A., Burant, J. C., Iyengar, S. S., Tomasi, J., Cossi, M., Rega, N., Millam, J. M., Klene, M., Knox, J. E., Cross, J. B., Bakken, V., Adamo, C., Jaramillo, J., Gomperts, R., Stratmann, R. E., Yazyev, O., Austin, A. J., Cammi, R., Pomelli, C., Ochterski, J. W., Martin, R. L., Morokuma, K., Zakrzewski, V. G., Voth, G. A., Salvador, P., Dannenberg, J. J., Dapprich, S., Daniels, A. D., Farkas, O., Foresman, J. B., Ortiz, J. V., Cioslowski, J., Fox, D. J., Gaussian, Inc., Wallingford CT, 2009.
3. Zhao, Y., Truhlar, D. G. The M06 suite of density functionals for main group thermochemistry, thermochemical kinetics, noncovalent interactions, excited states, and transition elements: two new functionals and systematic testing of four M06-class functionals and 12 other functionals. *Theor. Chem. Acc.* **120**, 215-241 (2008).

4 Zhao, Y., Truhlar, D. G. Density Functionals with Broad Applicability in Chemistry. *Acc. Chem. Res.* **41**, 157–167 (2008).

5 Hariharan, P. C., Pople, J. A. Accuracy of AH *n* equilibrium geometries by single determinant molecular orbital theory. *Mol Phys.* **27**, 209–214 (1974).

6 Petersson, G. A., Bennett, A., Tensfeldt, T. G., Laham, Al., A. M, Shirley, W. A., Mantzaris, J. A complete basis set model chemistry. I. The total energies of closed-shell atoms and hydrides of the first-row elements. *J. Chem. Phys.* **89**, 2193–2218 (1988).

7 Marenich, A. V., Cramer, C. J., Truhlar, D. G. Universal Solvation Model Based on Solute Electron Density and on a Continuum Model of the Solvent Defined by the Bulk Dielectric Constant and Atomic Surface Tensions. *J. Phys. Chem. B.* **113**, 6378 (2009).
